# Supplementary material for: Discovery of a Gatekeeper Residue in the C-Terminal Tail of the Extracellular Signal-Regulated Protein Kinase 5 (ERK5)
Source: Int J Mol Sci. 2020 Jan 31;21(3):929. doi: 10.3390/ijms21030929 (PMC7037328; doi:10.3390/ijms21030929)
Supplement: Supplementary file 1 [file ijms-21-00929-s001.pdf]

Supplementary Table 1: ERK5 binding partners

id	Protein IDs	Protein na	Gene name	Razor + un	Sequence c	LFQ intens	LFQ intens	LFQ intens	LFQ intens	LFQ intens	LFQ intens	LFQ intens	LFQ intens	LFQ intens	normalizec	normalizec	normalizec	normalizec	normalizec	normalizec	normalizec	normalizec	normalizec	median -	mediam FL	median Mu	st dev -	st dev fl	st dev mut	cluster1	cluster2	cluster3
950	H7BXZ6	Mitochond	RHOT1	2	6.5				1.08E+08									26.77							26.77					+		
405	B7WPL0	Synembryr	RIC8B	2	6.1						9.55E+07									26.75					26.75					+		
780	F6RFD5	Destrin	DSTN	5	37.8						3.94E+07									25.49					25.49					+		
360	B1AN48	Small proli	SPRR3	2	10.1				3.65E+07									25.24							25.24					+		
1376	P02808	Statherin	STATH	2	54.8						3.05E+07									25.12					25.12					+		
196	A0A0A0MT	Elongation	ELOVL5	2	8.4					1.92E+07	1.52E+07								24.35	24.09					24.22			0.188066		+		
2157	Q13163-4	Dual specif	MAP2K5	6	18.7				9.04E+06	2.09E+07	1.04E+07	8.78E+06	1.28E+07	1.06E+07				23.11	24.49	23.53	23.01	23.4	22.9		23.53	23.01		0.704014	0.263334		+	
2702	Q96AB3-3	Isochorism	ISOC2	2	28.1					8.02E+06									23.1						23.1					+		
64	A0A087W\	DEP domai	DEPDC7	5	9.6				4.88E+06	7.56E+06	1.20E+07	3.13E+06						22.21	23.03	23.69	21.54				23.03	21.54		0.742829			+	
1673	P30837	Aldehyde c	ALDH1B1	5	13.7				4.26E+06	6.20E+06	6.14E+06	3.97E+06						22.04	22.78	22.81	21.87				22.78	21.87		0.438767			+	
798	F8VW96	Cysteine ar	CSRP2	2	10.3				6.58E+06									22.68							22.68					+		
1055	K7EMW4	Nicalin	NCLN	3	8.6					6.52E+06	5.08E+06								22.83	22.53					22.68			0.210696		+		
1343	O95983-2	Methyl-Cp	MBD3	2	9.3						5.15E+06									22.57					22.57							
3072	Q9NZN4	EH domain	EHD2	4	10.5						4.97E+06									22.5					22.5					+		
1888	P52435	DNA-direct	POLR2J	2	28.2						4.85E+06									22.46					22.46					+		
457	C9JF17	Apolipopro	APOD	2	9.8					4.38E+06									22.26						22.26					+		
1553	P18440	Arylamine	NAT1	3	12.4					4.21E+06	3.09E+06								22.18	21.86					22.02			0.227825		+		
2469	Q6ZVX7	F-box only	NCCRP1	3	18.9				8.06E+06	1.70E+06		2.29E+06						22.95	20.97		21.06				21.96	21.06		1.395383			+	
1535	P16401	Histone H1	HIST1H1B	2	11.9						3.27E+06									21.94					21.94					+		
2487	Q7RTV0	PHD finger	PHF5A	2	14.5						3.25E+06									21.93					21.93					+		
438	C9K0R9	Inosine-5-r	IMPDH1	2	11.1					2.63E+06	3.80E+06								21.56	22.15					21.85			0.416833		+		
56	A0A087W\	ATP-deper	DDX50	2	6.6					3.07E+06									21.77						21.77					+		
328	A6PVX1	Selenium-k	SELENBP1	2	6.4						2.72E+06	1.38E+06								21.69	20.26				21.69	20.26				+		
1644	X6R6D0	SHC-transf	SHC1	2	14.1				3.07E+06	2.84E+06	1.85E+06							21.57	21.68	21.14					21.57			0.285118		+		
2654	Q92506	Estradiol 1	HSD17B8	2	10.3						2.49E+06									21.56					21.56					+		
252	A0A0D9SG	Band 4.1-li	EPB41L1	3	6					2.41E+06									21.43						21.43					+		
1449	P09104-2	Gamma-er	ENO2	2	19.7						2.14E+06									21.36					21.36							
3026	Q9NTX5-3	Ethylmalor	ECHDC1	2	8.4						1.98E+06									21.24					21.24							
726	E9PNM1	Squalene s	FDFT1	2	5.1				2.40E+06									21.23							21.23						+	
416	C9JEL3	Eukaryotic	EIF4E2	3	21.1						1.86E+06									21.16					21.16						+	
2616	Q8TDL5	BPI fold-co	BPIFB1	2	6.2					1.97E+06									21.12						21.12					+		
2515	Q86WH2	Ras associa	RASSF3	2	8.4						1.78E+06									21.04					21.04					+		
3169	Q9UNN5	FAS-associa	FAF1	3	5.4					1.63E+06									20.89						20.89					+		
2210	Q15424-2	Scaffold at	SAFB	3	8				1.48E+06	1.59E+06	1.82E+06							20.47	20.83	21.11					20.83			0.31803		+		
2773	Q96QD9-2	UAP56-inte	FYTTD1	2	8.2						1.48E+06									20.78					20.78					+		
776	F5H702	39S riboso	MRPL48	2	21.2						1.45E+06									20.76					20.76						+	
2931	Q9H2W6	39S riboso	MRPL46	2	7.5						1.17E+06									20.55					20.55					+		
2658	Q92541	RNA polym	RTF1	4	6.1						1.15E+06									20.53					20.53					+		
2630	S5FZ81	PHD finger	PHF10	2	5.3					1.27E+06									20.52						20.52					+		
902	H0YJV2	Serine palr	SPTLC2	2	7.9						1.10E+06									20.48					20.48					+		
648	E7EQY4	Metastasis	MTA3	2	13.6						1.06E+06									20.46					20.46						+	
2041	P82932	28S riboso	MRPS6	2	12.8						9.82E+05									20.39					20.39					+		
646	E9PES6	High mobil	HMGB3	2	17						8.95E+05									20.27					20.27					+		
3121	Q9UH62	Armadillo r	ARMCX3	2	6.3					1.01E+06									20.24						20.24					+		
2291	Q15599-2	Na(+)/H(+)	SLC9A3R2	2	8						6.62E+05									20.03					20.03					+		
379	B4DLC8	Signal tran	STAT2	3	9.6				9.80E+05	8.66E+05	6.13E+05							19.98	20.01	19.95					19.98			0.030185				
1063	K7EQQ8	tRNA (guar	TRMT1	2	6.1						5.59E+05									19.8					19.8							
2872	Q9BWS9-3	Chitinase d	CHID1	2	6.4				6.89E+05									19.74							19.74						+	
663	E7EVU5	Nuclear re	NCOR1	2	5.2						4.66E+05									19.72					19.72						+	
934	H3BPH4	Phosphom	PMM2	2	15.5						4.53E+05									19.59					19.59							
624	E5RIL9	Probable tl	OSGEPL1	2	10.8						2.87E+05									19.33					19.33					+		
2831	Q9BRP8-2	Partner of	WIBG	2	14.3					5.02E+05									19.33						19.33							
1561	P19474	E3 ubiquiti	TRIM21	26	46.7	1.55E+08	7.67E+07	7.44E+08	4.85E+09	5.96E+09	4.09E+09	5.53E+09	1.17E+09	8.10E+09	27.26	26.45	29.97	32.17	32.59	32.26	32.02	29.97	32.02	27.26	32.26	32.02	1.846841	0.224012	1.182819		+	
1233	O60701	UDP-gluco	UGDH	15	35.8	1.96E+06	2.45E+06	6.14E+06	2.24E+07	7.98E+07	9.19E+07	2.02E+07	1.66E+06	3.42E+06	20.83	21.49	22.85	24.49	26.43	26.7	24.28	20.43	21.09	21.49	26.43	21.09	1.031141	1.206704	2.057839	+		
207	A0A0B4J23	Immunoglo	IGLL5	2	10.7			8.99E+07	2.81E+09	4.98E+09	1.25E+08	6.58E+09					26.77	31.41	32.25	27.26	32.43			26.77	31.41	32.43		2.669901				
1972	P62191	26S protea	PSMC1	10	29.5	2.46E+06	3.43E+06	4.24E+06	7.05E+06	8.77E+07	9.56E+07	5.80E+06			21.2	21.97	22.33	22.78	26.61	26.77	22.44			21.97	26.61	22.44	0.581541	2.255997		+		
2066	Q01518-2	Adenylyl cy	CAP1	14	34.6	1.15E+07	9.67E+06	3.36E+07	1.47E+08	1.75E+08	1.00E+08	2.07E+08	1.35E+07	4.04E+07	23.44	23.38	25.18	27.32	27.54	26.85	27.69	23.46	24.82	23.44	27.32	24.82	1.023728	0.350751	2.155749	+		
2158	Q13164	Mitogen-ac	MAPK7	47	44	1.55E+09	1.21E+09	1.18E+09	3.37E+10	3.47E+10	2.56E+10	1.10E+10	2.17E+10	4.92E+10	31.05	30.96	30.98	34.49	34.61	35.14	33.57	34.26	34.25	30.98	34.61	34.25	0.046764	0.347154	0.397241		+	
1615	P25705	ATP syntha	ATP5A1	24	52.8	4.17E+07	2.51E+07	4.43E+07	8.79E+07	4.68E+08	4.12E+08	8.31E+07	3.89E+07	7.26E+07	25.3	24.74	25.64	26.55	28.96	28.91	26.29	25.03	25.54	25.3	28.91	25.54	0.456841	1.377217	0.63815	+		
1784	P43490	Nicotinami	NAMPT	13	28.9	7.52E+06	3.77E+06	1.02E+07	2.62E+07	6.06E+07	6.18E+07	4.61E+07	1.79E+07	2.87E+07	22.77	22.14	23.55	24.71	26.06	26.1	25.41	23.88	24.3	22.77	26.06	24.3	0.707558	0.794333	0.788737	+		
871	H0Y4R1	Inosine-5-r	IMPDH2	14	35.5	3.44E+06	9.31E+06	9.31E+06	2.03E+07	8.64E+07	1.01E+08	8.06E+06			21.68	23.32	23.42	24.32	26.58	26.86	22.88			23.32	26.58	22.88	0.978356	1.390409		+		
914	H0YMV8	40S riboso	RPS27L	3	32			7.96E+06			5.72E+07						23.21			25.97				23.21	25.97							
1350	P00390-2	Glutathion	GSR	5	14.8		3.03E+06	2.45E+06	9.38E+06	2.40E+07	1.77E+07	9.43E+06				21.78	21.53	23.17	24.63	24.33	23.12			21.65	24.33	23.12	0.175361	0.772106		+		
1759	P41091	Eukaryotic	EIF2S3	15	47	8.15E+06	3.08E+06	2.75E+07	3.28E+07	4.44E+07	1.28E+08	2.64E+07	2.32E+06	1.31E+07	22.92	21.81	24.91	25.08	25.55	27.34	24.65	20.98	23.24	22.92	25.55	23.24	1.571962	1.192313	1.854249	+		
1635	P27797	Calreticulir	CALR	11	43.6	7.44E+06	3.16E+06	5.31E+07	2.78E+07	3.53E+07	8.04E+07	2.56E+07			22.76	21.85	25.94	24.78	25.24	26.53	24.62			22.76	25.24	24.62	2.14908	0.910104			+	
3135	Q9UI12-2	V-type pro	ATP6V1H	9	26.5	3.79E+06	4.23E+06	1.40E+07	1.91E+07	3.42E+07	2.33E+07	2.61E+07	8.46E+06	1.49E+07	21.83	22.28	23.96	24.23	25.18	24.69	24.65	22.79	23.44	22.28	24.69	23.44	1.123569	0.4735	0.940296	+		
2853	Q9BUA3	Uncharact	C11orf84	15	58.8	8.55E+07	5.17E+07	8.44E+07	2.65E+08	3.67E+08	2.98E+08	4.08E+08	1.81E+08	4.26E+08	26.36	25.82	26.66	28.07	28.56	28.61	28.54	27.24	27.93	26.36	28.56	27.93	0.425094	0.295866	0.654708		+	
2325	Q16763	Ubiquitin-c	UBE2S	2	8.6			9.47E+05			4.27E+06						20.2			22.3				20.2	22.3							
681	E9PDI2	Adenylyl cy	CAP2	16	42.4	6.87E+07	2.67E+07	6.19E+07	2.81E+08	2.52E+08	1.72E+08	3.38E+08	1.16E+08	4.48E+08	25.98	24.84	26.16	28.15	28.06	27.77	28.23	26.57	28.08	25.98	28.06	28.08	0.716431	0.197281	0.921684		+	
2381	Q5SWX8	Protein od	ODR4	21	41.6	1.07E+08	9.09E+07	1.18E+08	4.41E+08	4.19E+08	3.39E+08	3.96E+08	3.06E+08	4.14E+08	26.77	26.69	27.12	28.73	28.79	28.69	28.46	27.85	27.85	26.77	28.73	27.85	0.230928	0.054329	0.35191	+		
1501	P12273	Prolactin-ir	PIP	3	28.1	5.90E+06	3.60E+06	1.31E+07	2.06E+07	2.34E+07	1.65E+07	8.89E+06	4.38E+07		22.41	22.05	23.9	24.35	24.6	24.2	23.03	25.16		22.41	24.35	24.1	0.98035	0.201854	1.509257		+	
2477	Q71U36-2	Tubulin alp	TUBA1A	3	71.4		2.13E+07	5.52E+07	3.88E+07	1.43E+08	1.18E+08	1.86E+08	8.65E+07			24.49	26	25.32	27.24	27.15	27.5	26.17		25.25	27.15	26.84	1.069677	1.085712	0.941436		+	
3130	Q9UHL4	Dipeptidyl	DPP7	10	24.2	3.93E+07	3.72E+07	5.00E+07	8.18E+07	1.46E+08	1.22E+08	1.37E+08	2.82E+07	4.57E+07	25.22	25.34	25.88	26.41	27.31	27.22	27.07	24.57	24.97	25.34	27.22	24.97	0.350507	0.49626	1.341042	+		
791	F8W1G3	Bax inhibit	TMBIM6	2	93.8	2.40E+06	1.82E+06	2.15E+06	8.82E+06	4.35E+06	7.13E+06				21.14	21.1	21.36	23.06	22.24	23.02				21.14	23.02		0.138276	0.464376				
1525	P14868	Aspartate-	DARS	18	43.9	2.36E+07	1.94E+07	3.25E+07	4.22E+07	7.46E+07	7.60E+07	1.23E+07	5.57E+06	7.64E+06	24.46	24.37	25.13	25.45	26.34	26.43	23.46	22.19	22.39	24.46	26.34	22.39	0.417851	0.540164	0.683806			
1184	O43175	D-3-phospl	PHGDH	12	29.5	7.92E+06	1.08E+07	1.69E+07	1.34E+07	3.90E+07	3.89E+07	3.14E+07	6.52E+06	6.60E+06	22.85	23.55	24.24	23.7	25.37	25.47	24.89	22.38	22.15	23.55	25.37	22.38	0.692545	0.993617	1.517847	+		

3001	Q9NQ92	Coordinatc	COPRS	6	36.4	2.56E+08	1.74E+08	1.01E+08	7.31E+08	7.33E+08	1.66E+08	6.80E+08	5.90E+08	1.24E+09	27.98	27.78	26.96	29.43	29.61	27.74	29.17	28.72	29.55	27.78	29.43	29.17	0.542178	1.033483	0.418476		+	
1822	P49257	Protein ER	LMAN1	5	17.5			3.61E+06	9.42E+06	1.27E+07	1.41E+07	1.15E+07					22.11	23.18	23.75	23.96	23.37			22.11	23.75	23.37		0.406305			+	
1457	P09622-3	Dihydrolip	DLD	9	24.7	8.73E+06		1.11E+07	3.04E+07	3.27E+07	2.77E+07	2.00E+07	1.21E+07	1.59E+07	23.01		23.68	24.88	25.12	24.96	24.24	23.34	23.55	23.35	24.96	23.55	0.472352	0.1234	0.472521	+		
3074	Q9P035	Very-long-	HACD3	4	15.5	7.41E+06	4.45E+06	3.72E+06	1.55E+07	1.48E+07	9.09E+06	1.24E+07	1.11E+07	9.83E+06	22.74	22.33	22.16	23.91	23.97	23.36	23.48	23.17	22.76	22.33	23.91	23.17	0.298162	0.333237	0.362755	+		
2337	Q3BBV0	Neuroblast	NBPF1	4	6.8	1.48E+06	1.07E+06	9.35E+05	4.28E+06	3.52E+06	2.48E+06				20.42	20.4	20.17	22.04	21.94	21.55				20.4	21.94		0.135556	0.259703				
1104	O00268	Transcripti	TAF4	18	31.2	6.79E+07	5.03E+07	4.19E+07	1.40E+08	1.44E+08	8.18E+07	1.64E+08	2.01E+08	2.50E+08	25.97	25.77	25.55	27.27	27.27	26.56	27.26	27.33	27.13	25.77	27.27	27.26	0.207641	0.412284	0.099021		+	
482	C9JZI7	Nucleosom	NAP1L4	4	23.7	2.72E+06	3.56E+06	5.27E+06	4.26E+06	1.07E+07	1.24E+07	5.11E+06			21.33	22.03	22.65	22.04	23.51	23.78	22.24			22.03	23.51	22.24	0.658389	0.937756		+		
2180	Q13509	Tubulin be	TUBB3	8	53.8	2.97E+07	3.18E+07	5.42E+07	6.91E+07	9.91E+07	8.05E+07	1.37E+08	7.35E+07	9.86E+07	24.81	25.07	25.97	26.13	26.81	26.54	27.07	25.93	26.02	25.07	26.54	26.02	0.610992	0.345922	0.632985		+	
2016	P63167	Dynein ligh	DYNLL1	4	62.9		1.80E+06			2.48E+06	1.16E+07					21.08			21.47	23.65				21.08	22.56			1.542099				
2631	Q8WUH6	Transmem	TMEM263	6	68.1	9.89E+07	1.93E+08	8.70E+07	2.99E+08	2.63E+08	1.76E+08	2.17E+08	4.51E+08	5.10E+08	26.66	27.96	26.7	28.2	28.12	27.84	27.74	28.28	28.37	26.7	28.12	28.28	0.741158	0.187321	0.343555		+	
2392	Q5T9A4	ATPase fan	ATAD3B	2	16.8		1.45E+06	2.06E+06	5.75E+06		4.51E+06					20.74	21.3	22.47		22.38				21.02	22.43		0.399135	0.068008				
1751	P40429	60S ribosoi	RPL13A	3	14.8		1.01E+06				2.66E+06	1.22E+07				20.28				21.66	23.44			20.28	21.66	23.44						+
3054	Q9NY12-2	H/ACA ribc	GAR1	9	37.7	1.01E+08	6.68E+07	6.74E+07	2.09E+08	2.59E+08	1.23E+08	9.14E+07	1.83E+08	8.24E+07	26.7	26.24	26.35	27.72	28.1	27.24	26.45	27.24	25.76	26.35	27.72	26.45	0.239528	0.433013	0.744113	+		
1989	P62487	DNA-direct	POLR2G	10	71.5	7.78E+07	4.70E+07	3.39E+07	1.29E+08	1.14E+08	7.36E+07	1.32E+08	1.79E+08	1.54E+08	26.24	25.65	25.2	27.11	26.96	26.4	26.99	27.23	26.48	25.65	26.96	26.99	0.525584	0.374478	0.379598		+	
1410	P06702	Protein S1(	S100A9	4	31.6	1.57E+07	1.05E+07	1.72E+07	3.34E+07	2.51E+07	3.47E+07	1.97E+07	1.46E+07		23.85	23.5	24.26	25.11	24.73	25.3	24.22	23.61		23.85	25.11	23.91	0.380554	0.290161	0.43076	+		
2983	Q9HC07	Transmem	TMEM165	4	22.8	8.60E+06	6.36E+06	6.07E+06	2.02E+07	1.60E+07	9.95E+06	1.41E+07	2.33E+07	1.92E+07	22.99	22.79	22.83	24.32	24.09	23.48	23.7	24.29	23.78	22.83	24.09	23.78	0.106987	0.435701	0.318633		+	
2602	Q8NHQ1	Centrosom	CEP70	14	29.5	6.03E+07	3.52E+07	3.17E+07	8.72E+07	8.34E+07	5.34E+07	1.00E+08	1.17E+08	1.23E+08	25.79	25.27	25.09	26.53	26.51	25.9	26.58	26.59	26.24	25.27	26.51	26.58	0.362963	0.359159	0.198759		+	
29	A0A087WU	Magnesiur	MAGT1	6	16.6	3.54E+06	3.02E+06	4.31E+06	9.05E+06	7.37E+06	4.90E+06	6.34E+06	7.61E+06		21.71	21.77	22.36	23.12	23	22.49	22.55	22.64		21.77	23	22.6	0.360131	0.336392	0.060659		+	
1927	P55795	Heterogen	HNRNPH2	8	43.9	1.07E+08	1.06E+08	7.29E+07	2.46E+08	3.26E+08	1.88E+08	1.64E+08	2.10E+08	1.48E+08	26.77	26.92	26.49	28.01	28.39	27.94	27.26	27.4	26.45	26.77	28.01	27.26	0.220124	0.24613	0.512965	+		
1908	P53985	Monocarb	SLC16A1	2	6.4	1.16E+07	1.62E+07	2.37E+06	2.55E+07	2.51E+07	1.90E+07	1.61E+07			23.45	24.11	21.47	24.67	24.73	24.43	23.89			23.45	24.67	23.89	1.369646	0.158586		+		
779	I3L0H8	ATP-deper	DDX19A	4	10.3	1.09E+06	2.02E+06	3.52E+06	5.63E+06	5.48E+06	4.78E+06	3.53E+06	3.10E+06	3.55E+06	20.07	21.24	22.05	22.44	22.59	22.44	21.68	21.41	21.14	21.24	22.44	21.41	0.993879	0.090279	0.270195	+		
50	A0A087W\	C-Myc-binc	MYCBP	5	58.2	1.84E+07	1.27E+07	4.22E+07	3.88E+07	3.47E+07	3.74E+07	8.42E+06	2.79E+07	1.16E+07	24.12	23.79	25.55	25.32	25.21	25.42	22.94	24.55	23.05	24.12	25.32	23.05	0.93731	0.100446	0.89785			
1257	O75351	Vacuolar p	VPS4B	7	21.2		3.42E+06	4.68E+06	7.27E+06	9.92E+06	9.25E+06	8.29E+06		6.81E+06		21.97	22.46	22.83	23.4	23.39	22.91		22.19	22.22	23.39	22.55	0.349422	0.329714	0.514128	+		
2154	Q13151	Heterogen	HNRNPA0	6	29.8	3.21E+07	3.17E+07	3.98E+07	7.36E+07	6.93E+07	6.23E+07	3.46E+07	4.88E+07	3.06E+07	24.95	25.06	25.5	26.23	26.23	26.12	25.04	25.36	24.38	25.06	26.23	25.04	0.292256	0.067722	0.499284	+		
3238	V9GZ56	U6 snRNA-	LSM4	5	17.6	9.17E+06	1.23E+07	9.77E+06	2.55E+07	3.21E+07	1.11E+07	2.09E+07	2.49E+07	1.25E+07	23.1	23.75	23.5	24.67	25.1	23.6	24.33	24.4	23.16	23.5	24.67	24.33	0.328049	0.771976	0.698338		+	
1815	P48651-3	Phosphatic	PTDSS1	2	9			6.78E+06	1.17E+07	2.53E+07		1.30E+07		1.20E+07			22.96	23.48	24.77		23.55		23.1	22.96	24.12	23.33		0.910213	0.317012	+		
932	H3BPE7	RNA-bindir	FUS	9	12.1	7.26E+07	4.90E+07	5.12E+07	1.27E+08	1.34E+08	8.41E+07	7.01E+07	5.25E+07		26.12	25.7	25.92	27.08	27.17	26.59	25.98	25.47		25.92	27.08	25.73	0.207496	0.310497	0.359497	+		
1647	P29508	Serpin B3	SERPINB3	5	12.8	3.54E+06	3.20E+06	1.38E+06	1.07E+07	4.71E+06		4.91E+06	4.57E+06	1.08E+07	21.71	21.87	20.75	23.35	22.37		22.18	21.91	22.94	21.71	22.86	22.18	0.608495	0.692821	0.536339	+		
1498	P12235	ADP/ATP t	SLC25A4	3	47.3	5.66E+06	5.37E+06	4.53E+06	1.24E+07	1.56E+07	8.24E+06	3.07E+06	2.60E+06		22.35	22.57	22.42	23.57	24.05	23.23	21.5	21.13		22.42	23.57	21.31	0.109084	0.412801	0.257938			
1978	P62266	40S ribosoi	RPS23	5	48.3	6.26E+07	9.91E+07	6.35E+07	1.09E+08	1.56E+08	1.27E+08	1.84E+08	2.02E+08	1.78E+08	25.84	26.78	26.2	26.8	27.39	27.32	27.48	27.36	26.64	26.2	27.32	27.36	0.476696	0.3223	0.453736		+	
2101	Q06587-2	E3 ubiquiti	RING1	2	8			1.68E+06	3.30E+06	4.25E+06	3.77E+06	2.83E+06					21.01	21.68	22.19	22.13	21.37			21.01	22.13	21.37		0.280755		+		
2692	Q969L4	U7 snRNA-	LSM10	6	61	3.65E+07	1.89E+07	2.43E+07	7.12E+07	5.51E+07	3.03E+07	4.63E+07	4.77E+07	7.43E+07	25.09	24.32	24.78	26.16	25.89	25.1	25.42	25.32	25.61	24.78	25.89	25.42	0.384186	0.554208	0.147869	+		
2043	P83369	U7 snRNA-	LSM11	11	37.5	7.11E+07	5.46E+07	4.92E+07	1.25E+08	1.47E+08	8.68E+07	1.02E+08	1.15E+08	9.36E+07	26.1	25.93	25.86	27.04	27.32	26.62	26.6	26.53	25.99	25.93	27.04	26.53	0.120761	0.352734	0.329411	+		
3113	Q9UBW8	COP9 signa	COPS7A	2	7.3			8.50E+05			1.81E+06						19.98			21.09				19.98	21.09					+		
2356	X6R8Y7	Spindlin-4	SPIN4	6	37.7	1.65E+07	9.37E+06	1.31E+07	3.16E+07	3.58E+07	2.41E+07	1.33E+07	3.04E+07	1.80E+07	23.93	23.34	23.9	24.98	25.27	24.72	23.59	24.71	23.7	23.9	24.98	23.7	0.332896	0.271345	0.618046	+		
2015	P63151	Serine/thre	PPP2R2A	12	38.7	3.63E+07	1.47E+07	5.98E+07	7.00E+07	6.14E+07	6.77E+07	6.54E+07	2.17E+07	4.55E+07	25.07	23.97	26.1	26.14	26.07	26.26	25.88	24.18	24.96	25.07	26.14	24.96	1.063882	0.098538	0.849474	+		
3094	Q9UBB4	Ataxin-10	ATXN10	23	46.7	2.19E+08	1.87E+08	3.14E+08	5.26E+08	4.30E+08	4.29E+08	9.35E+08	5.86E+08	3.89E+08	27.81	27.93	28.83	29	28.85	29	29.75	28.7	27.74	27.93	29	28.7	0.558003	0.087186	1.008157		+	
2046	P84090	Enhancer c	ERH	8	62.5	2.63E+08	1.07E+08	2.19E+08	4.08E+08	4.86E+08	7.62E+08	6.43E+08	1.07E+09	1.13E+09	28.01	26.95	28.25	28.62	29.08	29.82	29.12	29.91	29.43	28.01	29.08	29.43	0.690385	0.606154	0.399247			
980	I3L1P8	Mitochond	SLC25A11	12	50	2.25E+07	1.95E+07	2.52E+07	3.60E+07	5.20E+07	3.81E+07	2.17E+07	3.22E+07	1.56E+07	24.36	24.37	24.82	25.21	25.82	25.43	24.37	24.76	23.52	24.37	25.43	24.37	0.26233	0.306049	0.636286	+		
647	E7EX53	Ribosomal	RPL15	3	22.6	2.96E+06		3.34E+06	7.04E+06	5.11E+06	6.47E+06				21.49		21.97	22.78	22.49	22.87				21.73	22.78		0.338731	0.199743				
1096	O00165-5	HCLS1-assc	HAX1	4	24.7	3.54E+06	4.28E+06	2.78E+06	6.94E+06	6.77E+06	5.30E+06	5.66E+06	5.27E+06	3.78E+06	21.71	22.29	21.7	22.75	22.9	22.61	22.41	22.08	21.19	21.71	22.75	22.08	0.339382	0.145391	0.628484	+		
2077	Q02543	60S ribosoi	RPL18A	7	39.2	1.11E+07	9.45E+06	1.56E+07		2.36E+07	1.68E+07				23.39	23.36	24.12		24.62	24.25				23.39	24.43		0.42912	0.263222				
1910	P54105	Methyloso	CLNS1A	10	56.1	1.97E+09	8.93E+08	6.68E+08	2.89E+09	2.90E+09	1.32E+09	3.68E+09	4.72E+09	5.55E+09	31.68	30.44	29.87	31.47	31.51	30.65	31.51	31.8	30.85	30.44	31.47	31.51	0.927249	0.488843	0.489458		+	
1182	O43167	Zinc finger	ZBTB24	7	12.9	2.78E+07	1.75E+07	2.02E+07	4.41E+07	4.55E+07	3.38E+07	5.77E+07	4.43E+07	3.01E+07	24.67	24.24	24.48	25.52	25.6	25.27	25.73	25.18	24.36	24.48	25.52	25.18	0.215158	0.173371	0.692651		+	
2935	Q9H3K6	BolA-like p	BOLA2	7	80.2	3.17E+08	1.12E+08	4.75E+07	1.21E+08	2.46E+08	4.22E+08	4.52E+08	9.38E+08	1.41E+09	28.3	27.01	25.78	26.98	28.04	28.98	28.71	29.66	29.61	27.01	28.04	29.61	1.262799	1.003189	0.537066			+
1277	O75688	Protein ph	PPM1B	18	41.5	9.73E+08	5.56E+08	4.43E+08	1.29E+09	1.32E+09	9.27E+08	9.76E+08	1.61E+09	2.07E+09	30.02	29.41	29.26	30.44	30.64	30.07	29.84	30.59	29.95	29.41	30.44	29.95	0.398099	0.285375	0.403809	+		
380	B4DLN1	Mitochond	SLC25A10	13	34.4	2.12E+07	2.24E+07	2.96E+07	3.78E+07	4.55E+07	4.59E+07	3.50E+07	3.41E+07	2.00E+07	24.29	24.58	25.03	25.28	25.6	25.7	25.06	24.86	23.86	24.58	25.6	24.86	0.371762	0.21964	0.645648	+		
2820	Q9BPZ2	Spindlin-2E	SPIN2B	6	53.9	2.59E+07	2.01E+07	1.76E+07	4.46E+07	4.04E+07	3.26E+07	4.23E+07	5.69E+07	7.36E+07	24.57	24.4	24.3	25.54	25.42	25.25	25.32	25.57	25.58	24.4	25.42	25.57	0.135433	0.147816	0.150108			
1367	P01625	Ig kappa ch	IGKV4-1	3	21.1	1.10E+07	6.66E+06	2.18E+07	2.10E+07	1.46E+07	2.93E+07				23.38	22.84	24.61	24.39	23.94	25.06				23.38	24.39		0.908582	0.559424				
2530	Q8IW41-2	MAP kinas	MAPKAPK5	9	21.9	7.55E+06	5.47E+06	1.14E+07	1.41E+07	1.23E+07	2.04E+07	1.89E+07		8.24E+06	22.77	22.59	23.72	23.78	23.7	24.51	24.16		22.49	22.77	23.78	23.33	0.60726	0.444416	1.18412	+		
2334	Q2PZI1	Probable C	DPY19L1	16	29.2	2.66E+08	1.36E+08	1.64E+08	3.76E+08	3.94E+08	4.83E+08	4.90E+08	7.54E+08	4.35E+08	28.04	27.38	27.65	28.44	28.65	29.21	28.86	29.15	28.04	27.65	28.65	28.86	0.329879	0.397035	0.571946			
2397	Q5TF85	Protein FA	FAM46A	20	31.4	7.55E+08	5.56E+08	4.60E+08	1.22E+09	1.43E+09	1.03E+09	1.65E+09	1.93E+09	1.48E+09	29.58	29.41	29.34	30.39	30.85	30.32	30.84	30.85	29.65	29.41	30.39	30.84	0.123609	0.287015	0.689365			
2339	Q3SY69	Mitochond	ALDH1L2	8	10.7	2.03E+06	1.48E+06	3.80E+06	2.64E+06	3.37E+06	3.07E+06	2.11E+06	3.18E+06	3.28E+06	20.87	20.77	22.2	21.39	21.89	21.85	20.87	21.44	21.04	20.87	21.85	21.04	0.798229	0.27623	0.296721	+		
1363	P01040	Cystatin-A	CSTA	2	30.6	2.91E+06	1.46E+06	2.93E+06	1.01E+07		2.45E+06	3.74E+06	5.75E+06	9.19E+06	21.45	20.76	21.79	23.27		21.52	21.79	22.22	22.67	21.45	22.4	22.22	0.523876	1.23616	0.436493		+	
2519	Q86X83-2	COMM dor	COMMD2	2	10.7			1.74E+06	4.26E+06								21.09	22.04						21.09	22.04							
2491	Q7Z353-2	Highly dive	HDX	6	10	1.66E+07	8.31E+06	8.54E+06	1.93E+07	2.51E+07	1.44E+07	1.67E+07	1.06E+07	1.38E+07	23.95	23.17	23.29	24.24	24.73	24	23.96	23.1	23.32	23.29	24.24	23.32	0.418523	0.372787	0.443826	+		
1986	P62318-2	Small nucle	SNRPD3	5	55.8	4.52E+07	3.48E+07	6.44E+07	8.12E+07	2.38E+08	5.08E+07	2.20E+08	3.75E+08	4.87E+08	25.42	25.25	26.24	26.37	28	25.82	27.77	28.04	28.27	25.42	26.37	28.04	0.531969	1.131556	0.251108			+
2894	Q9C040	Tripartite r	TRIM2	4	7.5	4.91E+06	3.91E+06	3.23E+06	8.94E+06	9.25E+06	6.48E+06	7.18E+06	4.93E+06		22.15	22.19	21.93	23.09	23.3	22.88	22.72	22.01		22.15	23.09	22.37	0.141158	0.213253	0.498631	+		
2481	Q7L1I2	Synaptic ve	SV2B	4	6.4	1.12E+07	4.76E+06	4.44E+06	1.08E+07	1.56E+07	6.97E+06	2.74E+07	2.31E+07	1.55E+07	23.41	22.43	22.39	23.36	24.05	23	24.7	24.27	23.51	22.43	23.36	24.27	0.57715	0.535352	0.604132			+
1053	K7EMM8	Putative ox	GLYR1	13	32.8	4.88E+07	3.89E+07	4.67E+07	8.93E+07	8.02E+07	6.92E+07	5.09E+07	5.88E+07	3.85E+07	25.51	25.42	25.76	26.57	26.43	26.28	25.59	25.63	24.73	25.51	26.43	25.59	0.174249	0.142161	0.503936	+		
1982	P62304	Small nucle	SNRPE	2	25	6.63E+07	9.01E+06	2.84E+06	4.96E+06	1.70E+07	1.04E+08		2.02E+08	2.44E+08	25.92	23.28	21.75	22.25	24.19	26.94		27.36	27.09	23.28	24.19	27.23	2.109737	2.35685	0.192003			+
2086	Q03393	6-pyruvoyl	PTS	11	57.9	5.86E+08	4.17E+08	2.64E+08	1.16E+09	9.16E+08	7.11E+08	1.28E+09	1.77E+09	4.24E+09	29.17	29.04	28.61	30.35	29.95	29.7	30.48	30.76	30.53	29.04	29.95	30.53	0.295863	0.329649	0.149983			
1550	P18085	ADP-ribosy	ARF4	7	64.4	6.65E+07	5.59E+07	7.89E+07	1.32E+08	1.06E+08	9.50E+07	9.61E+07	8.34E+07	8.61E+07	25.92	25.98	26.57	27.17	26.89	26.73	26.54	26.13	25.86	25.98	26.89	26.13	0.359502	0.222778	0.339035	+		
2452	Q6QNY1	Biogenesis	BLOC1S2	9	54.9	3.96E+08	2.70E+08	1.91E+08	7.11E+08	6.52E+08	4.06E+08	4.87E+08	7.14E+08	6.85E+08	28.6	28.45	27.98	29.36	29.41	28.89	28.83	28.98	28.62	28.45	29.36	28.83	0.32231	0.284109	0.180362	+		
2694	Q969R5	Lethal(3)m	L3MBTL2	6	10.2	6.51E+06	4.28E+06	3.40E+06	9.48E+06	7.31E+06	9.79E+06	7.72E+06	7.40E+06	5.07E+06	22.56	22.29	21.98	23.19	23	23.46	22.83	22.58	21.71	22.29	23.19	22.58	0.290014	0.234423	0.5856	+		
2824	Q9BQE4	Selenoprot	VIMP	2	11.1			1.88E+06			3.62E+06						21.19			22.08				21.19	22.08							
802	F8VXU5	Vacuolar p	VPS29	5	26.6	1.03E+07	8.20E+06	1.11E+07		1.16E+07	2.41E+07	1.08E+07	4.57E+06		23.28	23.15	23.68		23.61	24.72	23.29	21.91		23.28	24.17	22.6	0.279008	0.78688	0.976179			
884	H0Y818	DNA mism	MLH1	4	6.8		2.94E+06	2.77E+06		6.18E+06	4.71E+06					21.73	21.7		22.77	22.43				21.71	22.6		0.025505	0.236999				
1953	P84077	ADP-ribosy	ARF1	11	58.6	7.49E+07	8.72E+07	1.95E+08	9.30E+07	1.68E+08	1.37E+08	4.59E+07	7.55E+07	5.22E+07	26.17	26.59	28.02	26.61	27.47	27.47	25.41	25.98	25.15	26.59	27.47	25.41	0.970088	0.49321	0.426552			
1652	P30041	Peroxiredo	PRDX6	17	75	1.26E+08	1.01E+08	2.04E+08	1.84E+08	2.32E+08	1.74E+08	9.45E+07	9.90E+07	6.25E+07	26.95	26.82	28.14	27.54	27.96	27.81	26.51	26.36	25.36	26.95	27.81	26.36	0.724532	0.214945	0.626621	+		
1335	O95721	Synaptosoi	SNAP29	7	33.7	1.49E+07	1.68E+07	2.08E+07	3.24E+07	3.23E+07	1.60E+07	1.48E+07	7.84E+06	7.20E+06	23.78	24.18	24.54	25.04	25.11	24.16	23.78	22.69	22.28	24.18	25.04	22.69	0.380019	0.528849	0.776223			
256	A0A0G2JIS	Histone-lys	EHMT2	13	14.7	1.56E+07	6.62E+06	6.09E+06	1.34E+07	1.32E+07	7.44E+06	1.02E+07	7.97E+06		23.84	22.83	22.84	23.7	23.82	23.09	23.22	22.7		22.84	23.7	22.96	0.579514	0.392561	0.369106	+		
2640	Q8WVX9	Fatty acyl-(	FAR1	8	18.8	1.09E+07	3.47E+06	1.04E+07	1.80E+07	1.85E+07	1.66E+07	2.19E+07	1.00E+07	2.01E+07	23.36	21.99	23.57	24.16	24.29	24.22	24.39	23.03	23.86	23.36	24.22	23.86	0.856488	0.068357	0.68585	+		
2586	Q8ND56-3	Protein LSI	LSM14A	8	17.5	4.21E+07	2.47E+07	2.19E+07	4.51E+07	4.99E+07	2.70E+07	2.52E+07	2.66E+07	2.92E+07	25.32	24.71	24.62	25.56	25.75	24.91	24.59	24.48	24.33	24.71	25.56	24.48	0.384299	0.443434	0.130811	+		
1701	P34897-3	Serine hyd	SHMT2	17	41.4	2.79E+07	1.44E+07	5.97E+07	3.83E+07	4.34E+07	6.14E+07	4.69E+07	1.41E+07	1.67E+07	24.68	23.94	26.09	25.29	25.52	26.1	25.45	23.53	23.63	24.68	25.52	23.63	1.092548	0.415613	1.077827			
3202	Q9Y2W1	Thyroid ho	THRAP3	36	32.7	8.27E+08	5.30E+08	7.61E+08	1.43E+09	1.25E+09	1.14E+09	1.08E+09	1.49E+09	2.02E+09	29.77	29.32	29.99	30.63	30.6	30.43	30.07	30.5	29.89	29.77	30.6	30.07	0.3379	0.103299	0.312767	+		
1109	O00330-3	Pyruvate d	PDHX	4	9.5			3.36E+06	5.94E+06	6.30E+06	6.39E+06	6.39E+06					21.97	22.52	22.79	22.86	22.57			21.97	22.79	22.57		0.181987			+	

3182	Q9Y224	UPF0568 p	C14orf166	7	32	2.89E+07	2.21E+07	2.93E+07	2.52E+07	4.45E+07	4.33E+07	1.95E+07	2.33E+07	2.72E+07	24.75	24.56	25.02	24.65	25.56	25.63	24.21	24.29	24.21	24.75	25.56	24.21	0.2274	0.546919	0.04673	+		
466	C9JUE0	Protein TF(	TFG	2	24.8	5.52E+06		2.71E+06	7.09E+06						22.3		21.66	22.79						21.98	22.79		0.457426					
2045	P84085	ADP-ribosy	ARF5	6	58.9		4.43E+06	9.83E+06	5.65E+06	1.35E+07	1.22E+07					22.33	23.51	22.45	23.84	23.73				22.92	23.73		0.837959	0.772796				
347	B0QYK0	RNA-bindir	EWSR1	5	11.5	3.10E+07	2.57E+07	2.48E+07	5.28E+07	4.53E+07	3.71E+07	4.13E+07	5.28E+07		24.89	24.8	24.8	25.77	25.59	25.41	25.28	25.48		24.8	25.59	25.38	0.054227	0.182637	0.140113		+	
805	F8VYN9	ADP-ribosy	ARL1	5	27.8	3.09E+07	2.95E+07	2.64E+07	4.87E+07	5.73E+07	3.87E+07	2.98E+07	5.15E+07	4.35E+07	24.89	24.96	24.87	25.68	25.96	25.46	24.81	25.43	24.91	24.89	25.68	24.91	0.044439	0.248012	0.333866	+		
142	F1T0B3	ATP-deper	DDX1	24	43.1	7.64E+07	5.88E+07	6.55E+07	1.21E+08	1.20E+08	1.06E+08	8.68E+07	8.83E+07	6.12E+07	26.2	26.05	26.26	26.98	27.03	26.99	26.36	26.23	25.32	26.2	26.99	26.23	0.110248	0.028021	0.568461	+		
1900	P53350	Serine/thre	PLK1	8	19.7	9.45E+06	5.80E+06	6.21E+06	1.29E+07	1.65E+07	1.15E+07	5.58E+06	1.08E+07	9.97E+06	23.14	22.65	22.87	23.65	24.16	23.64	22.38	23.13	22.79	22.87	23.65	22.79	0.245206	0.297925	0.37396	+		
2822	Q9BQ67	Glutamate	GRWD1	4	11.2			2.51E+06	3.68E+06	4.56E+06	7.82E+06						21.55	21.81	22.33	23.16				21.55	22.33			0.681741				
786	F6VRR5	Polymeras	POLDIP3	6	22.6	8.88E+06	5.87E+06	4.87E+06	1.14E+07	1.14E+07	9.52E+06			7.54E+06	23.05	22.66	22.52	23.43	23.59	23.42			22.37	22.66	23.43	22.37	0.272274	0.090795		+		
3163	Q9UN37	Vacuolar p	VPS4A	2	11			2.04E+06			3.58E+06						21.29			22.06				21.29	22.06							
2329	Q66S35	6-phospho	PFKFB4	8	27.9	6.95E+06	2.28E+06	8.04E+06	1.15E+07	6.19E+06	9.48E+06	4.57E+06			22.65	21.38	23.23	23.45	22.77	23.42	22.09			22.65	23.42	22.09	0.946046	0.385143				
612	E5RFP0	NudC dom	NUDCD2	3	29.5	5.70E+06	8.11E+06	3.63E+06		5.77E+06	1.09E+07				22.36	23.13	22.12		22.66	23.58				22.36	23.12		0.528228	0.646857				
2026	P68366-2	Tubulin alp	TUBA4A	4	62.4	1.17E+08	5.94E+07	1.20E+08	1.96E+08	2.18E+08	1.54E+08	2.62E+08	2.26E+08	2.16E+08	26.9	26.07	27.19	27.63	27.85	27.65	27.93	27.52	26.95	26.9	27.65	27.52	0.581776	0.117977	0.489055		+	
3061	Q9NZ01	Very-long-(	TECR	13	34.1	8.14E+07	4.83E+07	9.30E+07	8.76E+07	1.68E+08	1.10E+08	5.78E+07	4.71E+07		26.29	25.68	26.84	26.54	27.47	27.05	25.74	25.3		26.29	27.05	25.52	0.582172	0.46302	0.314934			
2521	Q86XN7	Proline anc	PROSER1	10	9.7	5.42E+07	3.33E+07	2.90E+07	5.91E+07	7.48E+07	5.17E+07	7.88E+07	8.11E+07	4.67E+07	25.67	25.17	25	25.92	26.35	25.86	26.17	26.08	25	25.17	25.92	26.08	0.34848	0.264406	0.651786			
2370	Q5JVZ5	Engulfmen	ELMO2	27	44.4	2.83E+08	1.93E+08	1.80E+08	4.40E+08	4.47E+08	2.39E+08	7.30E+08	7.38E+08	7.29E+08	28.11	27.96	27.87	28.71	28.93	28.24	29.33	29.06	28.82	27.96	28.71	29.06	0.123056	0.35274	0.252731			
841	G3V529	ATP-deper	DDX24	7	10.2	3.36E+06	2.88E+06	2.48E+06	3.16E+06	6.05E+06	4.60E+06	1.78E+06	2.09E+06		21.65	21.7	21.54	21.62	22.73	22.4	20.66	20.86		21.65	22.4	20.76	0.077913	0.570757	0.141946			
1255	O75340	Programm	PDCD6	6	28.3	2.31E+07	2.57E+07	2.17E+07	4.61E+07	3.83E+07	2.05E+07	1.25E+07	2.04E+07	1.41E+07	24.41	24.8	24.61	25.6	25.35	24.51	23.49	24.12	23.36	24.61	25.35	23.49	0.19539	0.570359	0.405227			
469	C9JME2	FERM, Rho	FARP1	17	19.5	2.33E+07	2.56E+07	2.25E+07	4.09E+07	4.27E+07	3.09E+07	1.92E+07	2.19E+07	1.33E+07	24.42	24.79	24.66	25.39	25.49	25.15	24.19	24.19	23.27	24.66	25.39	24.19	0.184421	0.177099	0.530549	+		
1019	J3KT68	Transmem	TMEM97	2	25		5.42E+06	5.78E+06		1.50E+07	6.27E+06					22.58	22.79		23.99	22.84				22.68	23.41		0.148902	0.813796				
1582	P22087	rRNA 2-O-r	FBL	15	44.2	1.06E+08	1.19E+08	1.01E+08	1.86E+08	1.97E+08	1.60E+08	8.65E+07	1.63E+08	1.41E+08	26.75	27.14	26.96	27.55	27.68	27.68	26.36	27.06	26.41	26.96	27.68	26.41	0.194837	0.07486	0.388575			
1689	P31947-2	14-3-3 prot	SFN	7	43.5	1.30E+08	1.05E+08	6.45E+07	1.33E+08	2.10E+08	1.50E+08	3.35E+08	2.77E+08		26.99	26.9	26.25	27.18	27.78	27.62	28.22	27.69		26.9	27.62	27.95	0.404956	0.307636	0.373548			
476	C9JWU9	Mesoderm	MEST	3	17.8			2.59E+06		4.77E+06	4.08E+06						21.61		22.39	22.25				21.61	22.32			0.101834				
2198	Q13885	Tubulin be	TUBB2A	5	63.8	3.18E+07	3.03E+07	5.03E+07	4.90E+07	4.63E+07	5.00E+07	8.27E+07	4.87E+07	3.30E+07	24.94	24.99	25.89	25.7	25.64	25.78	26.28	25.36	24.55	24.99	25.7	25.36	0.535125	0.070398	0.865445	+		
2354	Q53GT1	Kelch-like p	KLHL22	18	31.1	4.93E+07	2.50E+07	2.78E+07	4.90E+07	4.60E+07	3.20E+07	1.04E+08	5.79E+07	7.32E+07	25.52	24.73	24.92	25.7	25.63	25.19	26.64	25.61	25.57	24.92	25.63	25.61	0.416878	0.274631	0.610556		+	
2908	Q9H079	KATNB1-lik	KATNBL1	5	25		1.35E+07	1.30E+07		2.47E+07	1.97E+07					23.86	23.89		24.69	24.47				23.87	24.58		0.017513	0.156392				
2647	Q8WXD5	Gem-assoc	GEMIN6	3	21.6		3.63E+06	3.75E+06	7.35E+06	3.88E+06	6.15E+06		8.07E+06			22.05	22.17	22.85	22.09	22.81		22.73		22.11	22.81	22.73	0.081917	0.42865			+	
1656	P30049	ATP syntha	ATP5D	2	13.7			4.36E+06			7.37E+06						22.37			23.08				22.37	23.08							
3150	Q9UKK6	NTF2-relat	NXT1	4	30.7	9.90E+06	1.97E+07	2.20E+07	1.36E+07	3.62E+07	2.98E+07	1.36E+07	1.45E+07		23.21	24.38	24.63	23.72	25.28	25.08	23.64	23.6		24.38	25.08	23.62	0.755332	0.847784	0.031214			
3175	Q9UPQ9-1	Trinucleoti	TNRC6B	15	13.8	2.50E+07	1.71E+07	1.50E+07	3.91E+07	2.78E+07	2.30E+07	3.53E+07	1.81E+07	3.16E+07	24.52	24.22	24.06	25.34	24.91	24.67	25.08	23.91	24.44	24.22	24.91	24.44	0.234607	0.337567	0.586271	+		
2835	Q9BRX9	WD repeat	WDR83	5	21.9	7.94E+06	5.14E+06	3.92E+06	9.55E+06	6.00E+06	8.09E+06	6.03E+06	1.08E+07		22.86	22.51	22.24	23.19	22.71	23.21	22.5	23.13		22.51	23.19	22.81	0.313233	0.28388	0.449435	+		
2473	Q70IA6	MOB kinas	MOB2	12	50.6	1.63E+09	1.07E+09	8.99E+08	2.33E+09	2.24E+09	1.78E+09	1.20E+09	2.72E+09	2.90E+09	31.21	30.64	30.56	31.25	31.33	31.51	30.25	31.07	30.22	30.64	31.33	30.25	0.352521	0.13134	0.481454	+		
2289	Q15555-4	Microtubu	MAPRE2	2	13.5	3.36E+06	3.89E+06	3.11E+06	6.09E+06	6.75E+06	4.31E+06	4.90E+06	6.56E+06		21.65	22.18	21.89	22.57	22.89	22.31	22.17	22.4		21.89	22.57	22.28	0.266941	0.289139	0.159838	+		
1166	Q5T8U5	Surfeit locu	SURF4	3	22	8.76E+06	5.58E+06	1.89E+07	1.34E+07	3.49E+07	1.02E+07	9.70E+06	5.21E+07		23.02	22.62	24.39	23.7	25.22	23.5	23.15	25.45		23.02	23.7	24.3	0.929925	0.940482	1.621147			+
1984	P62314	Small nucle	SNRPD1	5	54.6	1.01E+09	7.53E+08	5.06E+08	1.46E+09	1.37E+09	1.19E+09	2.87E+09	2.89E+09	4.18E+09	30.19	29.99	29.45	30.67	30.71	30.49	31.3	31.15	30.48	29.99	30.67	31.15	0.381387	0.114767	0.438117			
1691	P31949	Protein S1(	S100A11	2	25.7	9.01E+07	6.81E+06	3.57E+06			1.07E+07	3.24E+06			26.5	22.88	22.08			23.56	21.58			22.88	23.56	21.58	2.352373					
314	A6NHQ2	rRNA/tRNA	FBLL1	2	6.9		1.52E+07	1.44E+07		2.62E+07	2.15E+07					24.03	24		24.8	24.57				24.01	24.69		0.023365	0.162721				
468	C9JLU1	DNA-direct	POLR2H	6	57.7	1.78E+07	1.84E+07	1.78E+07	3.24E+07	2.39E+07	2.77E+07	4.02E+07	3.88E+07		24.05	24.29	24.33	25.04	24.63	24.96	25.25	25.02		24.29	24.96	25.14	0.149259	0.22017	0.160915			
2214	Q14204	Cytoplasmi	DYNC1H1	75	20.4	2.46E+07	3.17E+07	4.60E+07	5.66E+07	4.92E+07	3.71E+07	2.06E+08	2.41E+08	1.27E+08	24.5	25.06	25.71	25.86	25.72	25.41	27.67	27.59	26.3	25.06	25.72	27.59	0.603587	0.233079	0.767553			+
1317	O95295	SNARE-ass	SNAPIN	4	41.9	7.43E+06	4.02E+06	2.99E+06	6.01E+06	6.90E+06	6.51E+06	4.11E+06	7.21E+06		22.75	22.23	21.82	22.56	22.93	22.89	21.94	22.55		22.23	22.89	22.25	0.465274	0.20415	0.42772	+		
1365	P01605	Ig kappa ch	IGKV3-15	2	25	2.50E+07	1.26E+07	1.25E+07	3.68E+07	1.86E+07	1.98E+07				24.52	23.78	23.82	25.26	24.3	24.48				23.82	24.48		0.415533	0.512315				
421	B9EGQ5	Protein Wi	WIZ	10	12.6	1.42E+07	7.20E+06	6.60E+06	1.44E+07	1.18E+07	9.18E+06	2.32E+07	1.41E+07		23.7	22.98	22.92	23.81	23.63	23.38	24.47	23.53		22.98	23.63	24	0.436376	0.214002	0.662947			
3140	Q9UJS0	Calcium-bi	SLC25A13	13	21.9	2.93E+07	2.03E+07	1.97E+07	3.09E+07	3.55E+07	3.03E+07	2.41E+07	1.98E+07	2.63E+07	24.78	24.41	24.44	24.93	25.25	25.1	24.52	24.07	24.17	24.44	25.1	24.17	0.203191	0.1583	0.236126	+		
708	E9PS17	N-terminal	SCYL1	6	10.3	1.05E+06			1.63E+06	1.75E+06	8.40E+05				19.95			20.61	21	20.21				19.95	20.61			0.396647		+		
1301	O94887-3	FERM, Rho	FARP2	2	6.3	3.63E+06	1.74E+06	1.25E+06	3.54E+06	2.71E+06		1.84E+06			21.75	21.02	20.64	21.74	21.61		20.71			21.02	21.68	20.71	0.5631	0.091598		+		
254	J3KNX4	PWWP dor	MUM1	6	11.4	6.41E+06	4.69E+06	4.45E+06		8.81E+06	6.52E+06	5.24E+06			22.55	22.4	22.39		23.22	22.89	22.28			22.4	23.05	22.28	0.08659	0.231816		+		
2502	Q7Z7G8-2	Vacuolar p	VPS13B	17	5.6	5.92E+06	7.13E+06	6.77E+06	1.18E+07	1.15E+07	1.11E+07	2.12E+07	3.09E+07	6.31E+06	22.42	22.96	22.95	23.49	23.6	23.6	24.35	24.74	22.09	22.95	23.6	24.35	0.310205	0.061468	1.432456			+
3251	Q9Y5Y2	Cytosolic F	NUBP2	6	31.7	8.24E+06	8.50E+06	1.11E+07	1.32E+07	1.60E+07	1.29E+07	1.20E+07	9.55E+06		22.94	23.19	23.68	23.68	24.09	23.84	23.42	22.96		23.19	23.84	23.19	0.378403	0.206185	0.324694	+		
2306	Q15942	Zyxin	ZYX	11	34.4	3.74E+07	3.31E+07	3.79E+07	5.39E+07	5.98E+07	4.13E+07	7.87E+07	4.80E+07	5.94E+07	25.12	25.15	25.43	25.79	26.02	25.56	26.16	25.33	25.29	25.15	25.79	25.33	0.170713	0.230047	0.491401	+		
1573	P20700	Lamin-B1	LMNB1	22	39.1	3.66E+07	2.87E+07	3.76E+07	5.07E+07	7.14E+07	4.33E+07	2.30E+07	1.48E+07	5.49E+06	25.1	24.93	25.42	25.74	26.26	25.63	24.46	23.63	21.83	25.1	25.74	23.63	0.250745	0.335678	1.342511			
2409	Q5VWN6-2	Protein FAI	FAM208B	17	15.1	3.55E+07	2.10E+07	2.27E+07	4.85E+07	2.68E+07	3.49E+07	4.81E+07	4.47E+07	3.11E+07	25.06	24.46	24.67	25.66	24.85	25.31	25.49	25.2	24.42	24.67	25.31	25.2	0.301846	0.403899	0.554344		+	
2883	Q9BYC9	39S riboso	MRPL20	2	12.8			3.54E+06	5.16E+06	5.93E+06	6.01E+06			4.43E+06			22.06	22.31	22.69	22.78			21.43	22.06	22.69	21.43		0.252388				
267	B0QYV1	RNA bindir	RBFOX2	4	14.3	8.73E+06	6.16E+06		1.19E+07	1.53E+07	9.23E+06	8.44E+06	8.67E+06	9.04E+06	23.01	22.74		23.5	24.01	23.39	22.94	22.83	22.62	22.88	23.5	22.83	0.192635	0.328362	0.163481	+		
1874	P51571	Translocon	SSR4	3	24.9	1.95E+07	5.04E+07	6.64E+07	6.17E+07	1.08E+08	7.45E+07	4.69E+07	5.37E+07	5.65E+07	24.18	25.79	26.3	25.96	26.91	26.41	25.45	25.51	25.22	25.79	26.41	25.45	1.104812	0.478396	0.154248	+		
2328	Q5W015	6-phospho	PFKFB3	25	58.1	2.38E+08	2.63E+08	2.22E+08	5.65E+08	4.21E+08	4.09E+08	2.32E+08	4.71E+08	4.51E+08	27.93	28.42	28.28	29.13	28.81	28.9	27.79	28.35	28.12	28.28	28.9	28.12	0.251183	0.165213	0.281175	+		
3095	Q9UBB5	Methyl-Cp(	MBD2	6	15.8	1.65E+07	1.11E+07	9.79E+06	1.89E+07	1.86E+07	1.42E+07	1.72E+07	2.81E+07	8.62E+06	23.93	23.59	23.51	24.22	24.3	23.97	24.02	24.56	22.55	23.59	24.22	24.02	0.226571	0.172867	1.039376		+	
66	A0A087WV	Rho GTPas	ARHGAP21	21	18.3	7.02E+07	7.07E+07	5.79E+07	1.01E+08	9.17E+07	7.05E+07	1.24E+08	1.46E+08	1.02E+08	26.05	26.33	26.06	26.71	26.68	26.32	26.91	26.91	26.06	26.06	26.68	26.91	0.159798	0.214463	0.490005			
1388	P04792	Heat shock	HSPB1	11	59.5	1.48E+08	1.46E+08	1.99E+08	2.43E+08	2.74E+08	2.21E+08	1.59E+08	2.50E+08	1.83E+08	27.2	27.49	28.1	27.96	28.2	28.1	27.24	27.6	26.69	27.49	28.1	27.24	0.458475	0.119914	0.460798	+		
2684	V9GYM8	Rho guanir	ARHGEF2	6	7.8	3.10E+06	2.52E+06	3.55E+06	2.59E+06	4.43E+06	3.80E+06		2.31E+06		21.54	21.53	22.07	21.36	22.28	22.15		20.96		21.54	22.15	20.96	0.311521	0.494738				
644	E7EQS8	Methylcytc	TET2	37	22.5	2.82E+08	1.54E+08	1.36E+08	2.93E+08	2.97E+08	1.85E+08	3.34E+08	2.21E+08	2.86E+08	28.1	27.57	27.42	28.18	28.3	27.9	28.2	27.48	27.27	27.57	28.18	27.48	0.356647	0.203813	0.491531	+		
2886	Q9BYE7	Polycomb g	PCGF6	2	6.9	1.81E+06	1.20E+06	8.72E+05	2.25E+06	1.99E+06	1.79E+06	2.21E+06			20.7	20.52	20.06	21.12	21.15	21.06	20.98			20.52	21.12	20.98	0.32874	0.044621			+	
1776	P42766	60S riboso	RPL35	4	29.3	3.61E+07	4.04E+07	6.81E+07	2.96E+07	6.12E+07	7.93E+07	3.88E+07	5.55E+07		25.07	25.46	26.37	24.85	26.06	26.5	25.22	25.54		25.46	26.06	25.38	0.666933	0.857406	0.232977	+		
1674	P31025	Lipocalin-1	LCN1	2	12.5	2.77E+06		1.16E+06	3.14E+06	2.51E+06		1.55E+06	2.31E+06		21.36		20.53	21.61	21.47		20.51	20.96		20.94	21.54	20.73	0.589182	0.094478	0.321685	+		
1820	P49189	4-trimethy	ALDH9A1	4	8.7		1.39E+06	2.47E+06		2.37E+06	3.44E+06	2.47E+06				20.68	21.54		21.4	22.02	21.15			21.11	21.71	21.15	0.606623	0.438569		+		
3153	Q9UL15	BAG family	BAG5	4	13.6	3.19E+06	3.70E+06	3.65E+06	3.13E+06	6.10E+06	5.57E+06	4.16E+06	4.67E+06		21.57	22.09	22.13	21.6	22.74	22.68	21.96	21.94		22.09	22.68	21.95	0.311629	0.643158	0.009641	+		
2506	Q86U86-5	Protein pol	PBRM1	23	18.6	2.59E+07	1.57E+07	1.46E+07	3.17E+07	2.05E+07	2.29E+07	9.11E+06	1.02E+07	7.80E+06	24.57	24.07	24.02	25	24.46	24.66	23.06	23.05	22.42	24.07	24.66	23.05	0.305431	0.272893	0.367196			
2643	Q8WWC4	Uncharacte	C2orf47	3	12		2.61E+06	2.33E+06		5.15E+06	2.73E+06					21.57	21.46		22.5	21.7				21.51	22.1		0.08138	0.5662				
1280	O75746	Calcium-bi	SLC25A12	3	8.7	1.75E+06	1.21E+06	1.34E+06	2.18E+06	2.54E+06	1.97E+06		1.76E+06		20.64	20.55	20.71	21.06	21.49	21.23		20.56		20.64	21.23	20.56	0.076336	0.218442		+		
3254	Q9Y657	Spindlin-1	SPIN1	16	74.8	1.95E+09	1.21E+09	9.58E+08	3.03E+09	3.63E+09	1.84E+09	2.84E+09	3.83E+09	3.53E+09	31.54	30.96	30.63	31.52	31.66	31.54	31.21	31.36	30.32	30.96	31.54	31.21	0.458379	0.072157	0.562202	+		
2990	Q9HD45	Transmem	TM9SF3	5	8.5	1.42E+07	9.86E+06	8.69E+06	1.88E+07	1.51E+07	1.27E+07	1.69E+07	1.38E+07	1.75E+07	23.7	23.42	23.31	24.21	24	23.81	23.98	23.5	23.67	23.42	24	23.67	0.203043	0.197264	0.244623	+		
1238	O60825	6-phospho	PFKFB2	20	50.1	9.08E+07	7.52E+07	7.39E+07	1.67E+08	1.21E+08	1.13E+08	7.48E+07	1.22E+08	4.92E+07	26.51	26.41	26.51	27.41	27.05	27.09	26.09	26.66	25.09	26.51	27.09	26.09	0.057583	0.196639	0.798814			
356	H0Y6Y8	39S riboso	MRPL43	5	30.8	6.93E+06	6.35E+06	6.36E+06	1.16E+07	6.77E+06	9.04E+06		8.35E+06	1.27E+07	22.65	22.78	22.89	23.46	22.9	23.36		22.77	23.18	22.78	23.36	22.98	0.122981	0.300938	0.287417	+		
3031	Q9NUQ8-2	ATP-bindin	ABCF3	8	15.1	1.08E+07	8.47E+06	7.77E+06	1.40E+07	1.32E+07	1.13E+07	9.57E+06	9.78E+06	1.23E+07	23.34	23.19	23.17	23.76	23.82	23.61	23.13	23	23.13	23.19	23.76	23.13	0.093943	0.104438	0.074698	+		
1470	P10599	Thioredoxi	TXN	7	51.4	1.00E+08	8.43E+07	1.20E+08	1.70E+08	1.43E+08	1.20E+08	1.46E+08	1.78E+08	2.28E+08	26.67	26.55	27.19	27.43	27.24	27.18	27.15	27.21	27.02	26.67	27.24	27.15	0.338801	0.128091	0.096496		+	
1316	O95292	Vesicle-ass	VAPB	4	22.6	9.99E+08	7.53E+08	5.61E+08	1.42E+09	1.23E+09	1.32E+09	9.19E+08	1.21E+09		30.14	29.99	29.63	30.55	30.55	30.65	29.69	30		29.99	30.55	29.85	0.259646	0.053571	0.214902	+		
1143	O14925	Mitochond	TIMM23	2	12.9		4.75E+06			7.26E+06		6.96E+06	7.32E+06			22.42			22.98		22.68	22.57		22.42	22.98	22.62			0.078631	+		
3016	Q9NS86	LanC-like p	LANCL2	4	13.3	6.72E+06	5.76E+06	5.77E+06		8.67E+06	8.00E+06	8.56E+06	1.20E+07		22.6	22.64	22.79		23.2	23.2	22.97	23.32		22.64	23.2	23.15	0.096493	0.004785	0.249347		+	

999	J3KNN5	Probable A	DDX41	14	25.2	3.21E+07	2.11E+07	1.70E+07	3.39E+07	2.54E+07	2.90E+07	2.86E+07	1.61E+07	3.08E+07	24.95	24.47	24.25	25.14	24.77	25.03	24.77	23.74	24.4	24.47	25.03	24.4	0.358366	0.185871	0.524298	+		
1810	P48507	Glutamate	GCLM	5	25.9	1.10E+07	8.52E+06	5.59E+06	1.29E+07	2.08E+07	1.23E+07	9.47E+06	1.37E+07		23.38	23.2	22.75	23.65	24.48	23.75	23.12	23.49		23.2	23.75	23.31	0.322941	0.45516	0.259707	+		
3077	Q9P0L0	Vesicle-ass	VAPA	5	22.1	1.42E+07	1.51E+07	1.65E+07	2.41E+07	2.47E+07	1.92E+07	1.07E+07	1.94E+07	2.10E+07	23.7	24.02	24.2	24.56	24.69	24.44	23.27	24.02	23.92	24.02	24.56	23.92	0.252696	0.124647	0.406306	+		
1829	P49419-2	Alpha-amir	ALDH7A1	11	29.9	6.20E+06	4.43E+06	2.36E+07	7.36E+06	7.58E+06	1.65E+07	1.88E+07	4.98E+06	8.40E+06	22.49	22.33	24.74	22.85	23.04	24.2	24.15	22.02	22.53	22.49	23.04	22.53	1.344169	0.732417	1.109699	+		
2025	P68363	Tubulin alp	TUBA1B	27	65.9	3.73E+09	2.27E+09	4.57E+09	6.00E+09	8.55E+09	7.13E+09	9.15E+09	6.31E+09	6.65E+09	32.64	32.21	33.24	32.6	33.48	33.18	32.96	32.06	31.47	32.64	33.18	32.06	0.52042	0.447602	0.749927			
1264	O75419-2	Cell divisio	CDC45	4	8.1		4.44E+06	3.04E+06		5.80E+06	5.22E+06					22.33	21.85		22.67	22.59				22.09	22.63		0.342646	0.060343				
1245	O75027	ATP-bindin	ABCB7	8	13.3	7.27E+06	5.60E+06	6.84E+06	1.00E+07	8.09E+06	8.47E+06	6.97E+06	1.13E+07	1.29E+07	22.72	22.62	22.97	23.26	23.1	23.26	22.68	23.2	23.21	22.72	23.26	23.2	0.181307	0.089998	0.29906		+	
1560	P19387	DNA-direct	POLR2C	8	39.3	2.73E+07	2.83E+07	2.29E+07	3.06E+07	5.05E+07	3.23E+07	7.30E+07	8.16E+07	4.38E+07	24.64	24.91	24.68	24.91	25.78	25.22	26.06	26.09	24.92	24.68	25.22	26.06	0.144676	0.441309	0.666026			+
1443	P08670	Vimentin	VIM	39	67.2	7.41E+08	8.12E+08	8.12E+08	1.03E+09	1.37E+09	1.55E+09	6.94E+08	6.35E+08	4.72E+08	29.5	30.18	30.17	30.1	30.71	31.18	29.21	28.8	28.19	30.17	30.71	28.8	0.391931	0.542302	0.511822			
3068	Q9NZJ5	Eukaryotic	EIF2AK3	39	49	7.12E+08	4.91E+08	4.45E+08	9.33E+08	8.55E+08	5.98E+08	9.39E+08	9.58E+08	6.69E+08	29.46	29.21	29.28	30.01	29.81	29.45	29.8	29.69	28.6	29.28	29.81	29.69	0.128459	0.285092	0.663109		+	
1726	P36542	ATP syntha	ATP5C1	9	28.5	1.02E+08	1.09E+08	8.63E+07	1.46E+08	1.43E+08	1.23E+08	1.00E+08	1.21E+08	1.53E+08	26.72	26.99	26.68	27.31	27.24	27.24	26.58	26.65	26.47	26.72	27.24	26.58	0.167402	0.039607	0.089099	+		
899	H0YHS6	Tyrosine--t	YARS2	2	11.3	2.12E+06			2.35E+06	2.68E+06	2.36E+06				20.95			21.2	21.59	21.48				20.95	21.48			0.202259				
2823	Q9BQA1	Methyloso	WDR77	21	83	3.16E+10	2.35E+10	1.89E+10	4.20E+10	3.72E+10	2.19E+10	5.78E+10	7.26E+10	6.56E+10	35.05	35.79	35.18	35.7	35.74	34.71	35.66	35.58	35.15	35.18	35.7	35.58	0.39768	0.582693	0.274691		+	
3199	Q9Y2T2	AP-3 comp	AP3M1	12	40.9	2.86E+07	2.21E+07	2.03E+07	3.63E+07	3.19E+07	2.64E+07	4.35E+07	4.67E+07	4.59E+07	24.72	24.56	24.49	25.23	25.08	24.85	25.33	25.29	24.98	24.56	25.08	25.29	0.117599	0.19291	0.189119			
228	A0A0C4DG	Probable d	DIMT1	4	19.3	7.10E+06	6.44E+06	5.06E+06	9.61E+06	8.84E+06	6.89E+06	9.11E+06	1.11E+07	9.34E+06	22.69	22.8	22.57	23.21	23.22	22.97	23.06	23.17	22.68	22.69	23.21	23.06	0.116698	0.14015	0.260056		+	
2310	Q16401-2	26S protea	PSMD5	16	44.9	9.68E+07	9.64E+07	1.19E+08	1.23E+08	1.44E+08	1.43E+08	1.35E+08	1.35E+08	8.65E+07	26.64	26.75	27.16	27.01	27.27	27.52	27.01	26.77	25.87	26.75	27.27	26.77	0.271384	0.255166	0.601071	+		
2637	Q8WV22	Non-struct	NSMCE1	11	39.1	1.62E+08	1.41E+08	1.68E+08	1.63E+08	2.29E+08	1.95E+08	1.36E+08	1.76E+08	6.58E+07	27.32	27.43	27.73	27.39	27.94	28	27.03	27.19	25.43	27.43	27.94	27.03	0.210313	0.335389	0.975449			
1269	O75489	NADH deh	NDUFS3	3	16.3		3.32E+06				4.78E+06					21.93				22.44				21.93	22.44							
2511	Q86V81	THO comp	ALYREF	6	40.1	8.43E+07	7.39E+07	7.34E+07	1.16E+08	1.24E+08	7.45E+07	7.84E+07	1.02E+08	8.40E+07	26.34	26.38	26.5	26.89	27.08	26.41	26.15	26.39	25.77	26.38	26.89	26.15	0.078727	0.344133	0.313137	+		
150	A0A0A0M(	Pyrroline-5	PYCRL	8	33.2	1.20E+07	1.08E+07	1.37E+07	1.69E+07	1.06E+07	1.67E+07	1.34E+07			23.49	23.55	23.93	24.06	23.5	24.23	23.59			23.55	24.06	23.59	0.241009	0.383927		+		
2326	Q16795	NADH deh	NDUFA9	6	17.2	5.33E+06	4.05E+06	5.24E+06	5.97E+06	8.61E+06	5.84E+06	3.87E+06	5.46E+06		22.26	22.24	22.64	22.54	23.19	22.75	21.84	22.14		22.26	22.75	21.99	0.225879	0.331062	0.217876	+		
2982	Q9HBH0	Rho-relate	RHOF	2	13.3		8.61E+05			1.21E+06						19.95			20.44					19.95	20.44							
2854	Q9BUF5	Tubulin be	TUBB6	9	56.7	8.95E+07	8.25E+07	9.84E+07	1.24E+08	1.56E+08	1.05E+08	1.81E+08	1.70E+08	1.85E+08	26.49	26.53	26.92	27.03	27.39	26.96	27.46	27.11	26.71	26.53	27.03	27.11	0.235472	0.232864	0.374957			
1454	P09496-2	Clathrin lig	CLTA	5	22	2.42E+07	1.69E+07	1.27E+07	2.36E+07	3.06E+07	2.34E+07	1.35E+07	1.92E+07	1.19E+07	24.49	24.2	23.85	24.54	25.03	24.69	23.61	24.01	23.09	24.2	24.69	23.61	0.318591	0.250807	0.461927			
296	A2A2Q9	Protein AA	AAR2	4	12.1		2.31E+06	2.90E+06		3.73E+06	3.76E+06		5.10E+06	6.16E+06		21.4	21.77		22.02	22.13		22.05	22.04	21.58	22.07	22.05	0.256543	0.077094	0.004365		+	
2725	Q96EE3	Nucleopor	SEH1L	8	24.7	1.17E+07	1.02E+07	1.03E+07	1.58E+07	1.46E+07	1.43E+07	1.18E+07	1.41E+07		23.46	23.47	23.56	23.96	23.94	23.98	23.39	23.53		23.47	23.96	23.46	0.053533	0.020294	0.097465	+		
2489	Q7Z2W9	39S ribosor	MRPL21	5	30.2	5.95E+06	4.55E+06	5.06E+06	7.44E+06	7.31E+06	6.67E+06	9.14E+06	8.41E+06	9.34E+06	22.43	22.37	22.57	22.86	23	22.91	23.07	22.78	22.68	22.43	22.91	22.78	0.104083	0.067404	0.203011		+	
3185	Q9Y265	RuvB-like 1	RUVBL1	17	54.2	7.54E+07	7.41E+07	8.99E+07	8.82E+07	1.04E+08	1.11E+08	9.59E+07	5.77E+07	6.46E+07	26.18	26.4	26.77	26.56	26.88	27.07	26.53	25.6	25.39	26.4	26.88	25.6	0.301424	0.259167	0.605278			
1499	P12236	ADP/ATP t	SLC25A6	18	53.7	1.32E+09	1.10E+09	8.71E+08	1.83E+09	1.97E+09	1.46E+09	1.20E+09	1.21E+09	1.16E+09	30.59	30.7	30.52	31.07	31.16	30.94	30.25	30	29.51	30.59	31.07	30	0.088617	0.112261	0.374542			
1384	P04350	Tubulin be	TUBB4A	2	58.6			4.81E+06			6.96E+06						22.51			22.99				22.51	22.99							
2065	Q01469	Fatty acid-l	FABP5	3	21.5		7.97E+06	8.61E+06			1.19E+07					23.1	23.31			23.68				23.21	23.68		0.144986					
340	A8MYQ9	Ras-relatec	RAB34	5	21.9	6.80E+06	6.08E+06	5.74E+06	7.23E+06	9.22E+06	7.90E+06	3.69E+06	5.50E+06		22.63	22.71	22.78	22.81	23.29	23.18	21.77	22.16		22.71	23.18	21.96	0.07552	0.250942	0.278984			
2561	Q8N4Q0	Zinc-bindir	ZADH2	2	6.4		4.40E+06	3.00E+06	5.98E+06	5.81E+06	3.71E+06	7.76E+06				22.32	21.83	22.55	22.68	22.11	22.83			22.07	22.55	22.83	0.349569	0.294881				
1387	P04746	Pancreatic	AMY2A	6	15.5	1.72E+07	1.69E+07	1.53E+07	2.44E+07	2.24E+07	1.99E+07	3.74E+07	1.90E+07	1.20E+07	24.01	24.2	24.09	24.6	24.56	24.49	25.15	23.98	23.1	24.09	24.56	23.98	0.096399	0.056873	1.029383	+		
364	H7BXF4	Sphingomy	SMPD4	5	6.6	3.21E+06	4.40E+06	4.28E+06	5.04E+06	6.51E+06	6.04E+06	3.22E+06	4.35E+06		21.59	22.32	22.35	22.27	22.83	22.79	21.57	21.84		22.32	22.79	21.71	0.428932	0.310622	0.188091			
849	G3XAN4	Translocati	TRAM1	2	7.3	5.13E+06	6.00E+06	5.39E+06	9.21E+06	9.70E+06	7.69E+06		1.06E+07		22.2	22.69	22.69	23.14	23.36	23.15		23.1		22.69	23.15	23.1	0.281542	0.122255			+	
103	A0A087W2	Pleckstrin l	PLEKHA2	4	14.1	6.99E+06	5.32E+06	4.48E+06	6.21E+06	9.38E+06	7.03E+06	5.50E+06	6.82E+06	6.94E+06	22.67	22.55	22.41	22.6	23.32	23.01	22.36	22.46	22.22	22.55	23.01	22.36	0.131101	0.363283	0.118747	+		
861	G8JLB6	Heterogen	HNRNPH1	22	54	3.34E+09	2.67E+09	1.84E+09	6.51E+09	6.06E+09	4.30E+09	6.21E+09	8.64E+09	7.74E+09	32.26	32.28	31.96	32.74	32.72	32.46	32.18	32.65	31.91	32.26	32.72	32.18	0.179642	0.156091	0.373984	+		
1254	O75330-4	Hyaluronar	HMMR	3	6.4	1.94E+06	1.38E+06	1.36E+06		1.91E+06	2.01E+06	2.06E+06			20.8	20.66	20.72		21.09	21.27	20.82			20.72	21.18	20.82	0.072319	0.126969		+		
2055	Q00535	Cyclin-dep	CDK5	6	26.7	9.32E+06	8.36E+06	7.95E+06	1.28E+07	1.37E+07	8.82E+06	1.32E+07	1.34E+07	1.46E+07	23.12	23.18	23.21	23.63	23.86	23.31	23.57	23.46	23.41	23.18	23.63	23.46	0.044832	0.275276	0.084529		+	
1606	P24666	Low molec	ACP1	9	53.8	2.78E+07	2.97E+07	2.40E+07	3.62E+07	2.52E+07	3.55E+07	3.44E+07	3.62E+07	4.68E+07	24.67	24.97	24.76	25.22	24.75	25.33	25.03	24.92	25.01	24.76	25.22	25.01	0.149569	0.309913	0.057836	+		
3109	Q9UBU9	Nuclear RN	NXF1	24	48.8	8.15E+08	6.56E+08	6.80E+08	9.31E+08	1.03E+09	9.84E+08	7.91E+08	9.17E+08	3.18E+08	29.7	29.63	29.89	29.99	30.17	30.15	29.41	29.61	27.39	29.7	30.15	29.41	0.136154	0.097423	1.232168	+		
1371	P02545	Prelamin-A	LMNA	36	53	2.23E+08	1.93E+08	2.29E+08	2.46E+08	3.27E+08	2.80E+08	1.69E+08	1.14E+08	5.34E+07	27.84	27.96	28.35	28.01	28.41	28.43	27.31	26.5	25.19	27.96	28.41	26.5	0.262272	0.239492	1.067582			
16	K7ERP4	Glutathion	GPX4	3	23.9	5.41E+06	4.45E+06	5.37E+06	6.33E+06	6.21E+06	6.24E+06	3.90E+06	5.82E+06	7.02E+06	22.28	22.33	22.69	22.62	22.78	22.83	21.85	22.24	22.24	22.33	22.78	22.24	0.221078	0.110342	0.227008	+		
2237	Q14739	Lamin-B re	LBR	4	7.3	1.78E+07	1.21E+07	1.00E+07	1.90E+07	1.68E+07	1.52E+07	1.14E+07	1.43E+07	1.24E+07	24.05	23.73	23.53	24.23	24.18	24.09	23.35	23.57	23.14	23.73	24.18	23.35	0.263284	0.071243	0.214087			
1291	O75955-2	Flotillin-1	FLOT1	3	9.2		1.71E+06	1.36E+06			2.07E+06					20.98	20.72			21.3				20.85	21.3		0.182264					
2009	P62979	Ubiquitin-4	RPS27A	9	48.7	9.70E+08	7.01E+08	8.67E+08	1.30E+09	1.14E+09	1.02E+09	1.44E+09	1.30E+09	2.09E+09	29.98	29.79	30.5	30.49	30.42	30.24	30.71	30.11	30	29.98	30.42	30.11	0.3662	0.128154	0.383412	+		
2755	Q96KP4	Cytosolic n	CNDP2	4	12.4	4.00E+06	3.29E+06	7.55E+06	4.40E+06	4.66E+06	6.09E+06	5.49E+06			21.89	21.91	23.14	22.08	22.36	22.8	22.36			21.91	22.36	22.36	0.713257	0.365415			+	
1708	P35251-2	Replicatior	RFC1	8	9.7	6.27E+06	5.44E+06	6.39E+06	6.20E+06	7.45E+06	7.38E+06	9.63E+06	8.62E+06		22.51	22.58	22.9	22.59	23.02	23.08	23.15	22.82		22.58	23.02	22.98	0.20829	0.266677	0.228861		+	
1593	P23258	Tubulin ga	TUBG1	14	43.5	3.91E+07	5.09E+07	3.50E+07	4.44E+07	5.98E+07	4.64E+07	5.51E+07	5.30E+07	5.09E+07	25.21	25.8	25.28	25.53	26.02	25.72	25.69	25.49	25.12	25.28	25.72	25.49	0.322956	0.245512	0.28946	+		
1700	P34896-2	Serine hyd	SHMT1	7	24.3	9.44E+06	8.99E+06	1.13E+07	1.24E+07	1.24E+07	1.36E+07	1.01E+07	1.04E+07	7.73E+06	23.13	23.28	23.7	23.57	23.71	23.91	23.21	23.08	22.41	23.28	23.71	23.08	0.296768	0.168424	0.42751	+		
1218	O60331-4	Phosphatic	PIP5K1C	20	44.5	5.74E+08	3.05E+08	3.43E+08	6.88E+08	7.82E+08	5.92E+08	4.40E+08	7.35E+08	9.91E+08	29.13	28.66	28.98	29.33	29.71	29.42	28.64	29.03	29.26	28.98	29.42	29.03	0.238569	0.197475	0.311861	+		
2116	Q08554-2	Desmocoll	DSC1	8	11	4.26E+06	7.61E+06	3.34E+07	1.25E+07	9.85E+06	9.97E+06	2.86E+06	1.22E+07		21.98	23.04	25.17	23.58	23.39	23.48	21.4	23.35		23.04	23.48	22.37	1.622105	0.096895	1.378011			
1398	P05141	ADP/ATP t	SLC25A5	6	45	4.31E+08	2.98E+08	2.24E+08	5.36E+08	5.36E+08	3.78E+08	2.54E+08	3.70E+08	2.81E+08	28.73	28.61	28.29	29.04	29.22	28.85	27.91	28.01	27.22	28.61	29.04	27.91	0.228342	0.18817	0.429571			
2410	Q5VXN0	Ribosome	RPF2	2	11.7		2.52E+06	2.88E+06		3.96E+06	3.56E+06					21.53	21.76		22.11	22.05				21.64	22.08		0.167007	0.039156				
2837	Q9BSD7	Cancer-rel	NTPCR	6	38.4	7.91E+06	8.35E+06	8.43E+06	1.27E+07	1.22E+07	8.80E+06	8.60E+06	7.83E+06	9.82E+06	22.85	23.17	23.28	23.61	23.69	23.3	22.98	22.68	22.75	23.17	23.61	22.75	0.225491	0.207106	0.154435			
2579	X6R5E5	Ubiquitin-a	UBAC2	3	14.7	4.05E+06	3.40E+06	2.54E+06	5.27E+06			3.88E+06		4.03E+06	21.91	21.96	21.58	22.34			21.84		21.29	21.91	22.34	21.56	0.206148		0.38898			
620	E5RHG8	Transcripti	TCEB1	2	36		2.47E+06	2.80E+06			3.51E+06					21.5	21.71			22.04				21.61	22.04		0.14908					
1111	O00411	DNA-direct	POLRMT	5	7	5.27E+06	3.58E+06	2.75E+06	4.16E+06	5.33E+06	4.87E+06				22.24	22.04	21.69	21.99	22.56	22.47				22.04	22.47		0.279855	0.305303				
1932	P57740	Nuclear po	NUP107	25	31	5.84E+07	8.09E+07	5.43E+07	9.22E+07	7.90E+07	5.77E+07	3.26E+07	2.51E+07	4.72E+06	25.77	26.52	25.98	26.6	26.41	25.99	24.95	24.4	21.58	25.98	26.41	24.4	0.383843	0.314657	1.808244			
843	G3V5T9	Cyclin-dep	CDK2	16	57.2	1.93E+08	2.05E+08	1.67E+08	2.71E+08	2.03E+08	2.35E+08	2.41E+08	2.70E+08	1.79E+08	27.66	28.03	27.69	28.12	27.74	28.21	27.84	27.66	26.67	27.69	28.12	27.66	0.205971	0.24644	0.628422	+		
2660	Q92572	AP-3 comp	AP3S1	7	43.5	1.06E+07	1.01E+07	7.82E+06	1.38E+07	9.77E+06	1.24E+07	2.03E+07	2.61E+07		23.31	23.45	23.18	23.74	23.36	23.78	24.29	24.46		23.31	23.74	24.38	0.134468	0.229434	0.116966			+
1139	O14828	Secretory c	SCAMP3	4	23.1	5.16E+06	5.25E+06	3.80E+06	6.38E+06	4.47E+06	6.03E+06	5.64E+06	7.46E+06	9.56E+06	22.21	22.53	22.2	22.64	22.29	22.79	22.41	22.6	22.72	22.21	22.64	22.6	0.188653	0.252986	0.15898		+	
3083	Q9P227-2	Rho GTPas	ARHGAP23	6	8.6	6.32E+06	3.30E+06	3.22E+06	5.58E+06	4.65E+06	2.71E+06				22.52	21.92	21.92	22.43	22.35	21.68				21.92	22.35		0.347333	0.409438				
2864	Q9BVJ7	Dual specif	DUSP23	2	13.3	8.83E+05	6.43E+05	7.85E+05	1.03E+06	8.40E+05	8.57E+05	1.18E+06			19.6	19.72	19.86	20.14	19.97	20.26	20.04			19.72	20.14	20.04	0.12894	0.144266			+	
1686	P31942-2	Heterogen	HNRNPH3	3	13.3	1.47E+07	1.17E+07	9.83E+06		1.61E+07					23.76	23.68	23.51		24.1					23.68	24.1		0.124104					
3138	Q9UII4	E3 ISG15--|	HERC5	7	7.1	3.56E+06	2.52E+06	2.02E+06	3.14E+06	3.84E+06	3.29E+06				21.72	21.53	21.28	21.61	22.07	21.95				21.53	21.95		0.221963	0.241577				
1692	P32119	Peroxiredo	PRDX2	14	59.6	5.89E+08	6.29E+08	6.41E+08	9.20E+08	1.09E+09	7.34E+08	1.12E+09	8.40E+08	9.09E+08	29.21	29.56	29.73	29.98	30.36	29.8	30.12	29.4	29.1	29.56	29.98	29.4	0.267062	0.286048	0.524854	+		
1436	P08240	Signal reco	SRPR	8	14.9	1.56E+07	9.56E+06	9.10E+06	1.43E+07	1.48E+07	1.11E+07	1.00E+07	1.04E+07		23.84	23.38	23.38	23.8	23.97	23.6	23.2	23.08		23.38	23.8	23.14	0.267234	0.184662	0.083504	+		
1340	O95881	Thioredoxi	TXNDC12	6	40.1	2.62E+08	1.82E+08	1.89E+08	3.50E+08	3.04E+08	3.05E+08	2.71E+08	4.56E+08	3.59E+08	28	27.9	27.96	28.37	28.32	28.62	27.95	28.3	27.64	27.96	28.37	27.95	0.054083	0.16057	0.331169	+		
1909	P53999	Activated F	SUB1	9	50.4	3.51E+08	2.42E+08	1.84E+08	4.35E+08	3.41E+08	3.60E+08	4.26E+08	5.74E+08	4.24E+08	28.49	28.27	27.9	28.69	28.48	28.75	28.61	28.68	27.88	28.27	28.69	28.61	0.299405	0.144429	0.440518		+	
2201	Q14011	Cold-induc	CIRBP	7	45.3	9.64E+07	9.97E+07	1.85E+08	1.36E+08	4.38E+08	6.81E+07	5.41E+07	1.18E+08	2.27E+07	26.63	26.8	27.92	27.21	28.91	26.28	25.66	26.62	24	26.8	27.21	25.66	0.702293	1.334375	1.325365			
610	E3W994	CLIP-assoc	CLASP2	7	7.1	3.42E+06	2.84E+06	4.12E+06	3.17E+06	4.44E+06	3.62E+06	3.58E+06	3.55E+06		21.67	21.66	22.29	21.62	22.29	22.08	21.71	21.57		21.67	22.08	21.64	0.361258	0.339037	0.097518	+		
1339	O95831-3	Apoptosis-	AIFM1	12	25.1	3.07E+07	3.22E+07	3.30E+07	4.38E+07	4.55E+07	3.71E+07	4.90E+07	5.35E+07	3.09E+07	24.87	25.09	25.15	25.5	25.6	25.41	25.53	25.5	24.41	25.09	25.5	25.5	0.146506	0.09672	0.638118		+	
2761	Q96N66-3	Lysophosp	MBOAT7	3	11.6	6.35E+06	2.77E+06	2.97E+06	5.18E+06	4.30E+06	3.39E+06	5.50E+06	4.67E+06	4.81E+06	22.53	21.63	21.81	22.31	22.22	21.99	22.36	21.94	21.61	21.81	22.22	21.94	0.478908	0.163155	0.376219	+		
3134	Q9UI09	NADH deh	NDUFA12	2	17.2			1.05E+06			1.45E+06						20.35			20.76				20.35	20.76							
2174	Q13423	NAD(P) tra	NNT	7	7.8	3.48E+06	2.32E+06	5.48E+06	3.74E+06	3.92E+06	4.35E+06	4.23E+06	1.45E+06		21.69	21.41	22.73	21.84	22.1	22.33	21.99	20.25		21.69	22.1	21.12	0.694798	0.244273	1.230351			

2398	Q5TFE4-2	5-nucleotic	NT5DC1	2	6.7		1.64E+06				2.10E+06					20.93				21.33				20.93	21.33							
2749	Q96J02-2	E3 ubiquiti	ITCH	6	8			1.78E+06		2.65E+06	2.42E+06						21.13		21.57	21.5				21.13	21.54			0.044406				
1854	P50402	Emerin	EMD	5	31.1	6.98E+06	1.28E+07	7.48E+06	1.21E+07	1.22E+07	9.41E+06	9.38E+06	1.20E+07		22.66	23.8	23.13	23.54	23.69	23.41	23.1	23.32		23.13	23.54	23.21	0.571263	0.141011	0.156826	+		
2084	Q03135	Caveolin-1	CAV1	8	47.8	4.07E+08	2.78E+08	5.13E+08	5.37E+08	4.22E+08	4.59E+08	4.88E+08	7.24E+08	7.94E+08	28.66	28.49	29.48	29.06	28.83	29.07	28.84	29.01	28.94	28.66	29.06	28.94	0.531285	0.138593	0.084022		+	
2533	Q8IWI9-4	MAX gene-	MGA	47	19.9	1.96E+08	1.27E+08	1.26E+08	2.01E+08	1.97E+08	1.60E+08	1.84E+08	1.64E+08	1.64E+08	27.68	27.27	27.27	27.67	27.68	27.68	27.48	27.07	26.54	27.27	27.68	27.07	0.234485	0.008224	0.470621	+		
2783	Q96S66	Chloride ch	CLCC1	13	24.5	4.96E+07	3.91E+07	3.55E+07	3.64E+07	6.04E+07	5.12E+07	9.11E+07	7.77E+07	5.27E+07	25.53	25.43	25.31	25.24	26.05	25.83	26.44	26.03	25.16	25.43	25.83	26.03	0.108535	0.420489	0.64975			
1468	P10398	Serine/thre	ARAF	7	12.9	1.09E+07	7.81E+06	6.92E+06	1.27E+07	1.05E+07	9.77E+06	1.51E+07	1.24E+07		23.36	23.08	22.99	23.61	23.48	23.46	23.82	23.36		23.08	23.48	23.59	0.1931	0.080118	0.325455			
3224	Q9Y3U8	60S riboso	RPL36	2	21			9.94E+06			1.37E+07						23.53			23.93				23.53	23.93							
3257	Q9Y679-3	Ancient ub	AUP1	3	12.1		2.50E+06	2.21E+06	2.38E+06	3.30E+06	3.41E+06	5.94E+06	6.08E+06	5.02E+06		21.51	21.4	21.21	21.86	22	22.47	22.31	21.68	21.46	21.86	22.31	0.076451	0.420615	0.415721			+
1415	Q16778	Histone H2	HIST2H2BE	2	52.4	1.93E+07	1.26E+07	1.53E+07	2.24E+07	1.48E+07	2.17E+07	4.65E+07			24.18	23.78	24.09	24.49	23.97	24.58	25.43			24.09	24.49	25.43	0.210388	0.332272				+
2179	Q13472-2	DNA topois	TOP3A	7	7.9	4.76E+06	1.92E+06	3.30E+06	4.12E+06	7.95E+06	4.45E+06	2.26E+06	3.45E+06	2.61E+06	22.1	21.19	21.96	21.98	23.09	22.36	21.04	21.53	20.73	21.96	22.36	21.04	0.492021	0.56444	0.405261			
3245	Q9Y5L0-3	Transporti	TNPO3	8	11.4	5.96E+06	1.06E+07	4.40E+06	9.50E+06	5.80E+06	6.28E+06	1.55E+07			22.44	23.52	22.38	23.19	22.67	22.84	23.84			22.44	22.84	23.84	0.639453	0.264362				+
429	H7C1T3	Choline-ph	PCYT1A	3	24.4	3.02E+06	3.32E+06	2.19E+06		2.93E+06	3.71E+06		2.58E+06		21.52	21.93	21.39		21.72	22.11		21.12		21.52	21.92	21.12	0.281931	0.28147				
623	E5RIY4	Transcripti	MTERF3	2	16.2		2.09E+06	1.81E+06	3.17E+06	2.90E+06	1.74E+06		2.98E+06			21.3	21.15	21.62	21.7	21.01		21.36		21.23	21.62	21.36	0.109322	0.380648		+		
2845	Q9BT67	NEDD4 fan	NDFIP1	6	31.2	2.96E+07	3.42E+07	4.60E+07	4.36E+07	6.68E+07	4.31E+07	5.53E+07	6.21E+07		24.8	25.22	25.71	25.49	26.18	25.61	25.7	25.69		25.22	25.61	25.7	0.455576	0.367916	0.002485			
784	F6SS63	TGF-beta-a	TAB3	6	15.6	2.07E+07	1.17E+07	1.27E+07	1.77E+07	2.49E+07	1.68E+07	2.51E+07	2.46E+07	1.97E+07	24.25	23.68	23.85	24.11	24.71	24.25	24.58	24.38	23.82	23.85	24.25	24.38	0.294072	0.3156	0.391148			
2893	Q9BZX2	Uridine-cyt	UCK2	5	21.8	9.92E+06	9.73E+06	7.81E+06	1.54E+07	1.16E+07	8.81E+06	5.14E+06	4.51E+06		23.22	23.39	23.18	23.9	23.61	23.31	22.25	21.89		23.22	23.61	22.07	0.114426	0.296028	0.252281			
1294	O76031	ATP-depen	CLPX	8	14.4	1.28E+07	7.48E+06	8.50E+06	1.46E+07	1.21E+07	9.80E+06	1.35E+07	1.39E+07	1.18E+07	23.58	23.02	23.29	23.83	23.68	23.47	23.61	23.51	23.08	23.29	23.68	23.51	0.278066	0.184972	0.283906	+		
1827	P49406	39S riboso	MRPL19	5	12.3	4.61E+06	4.08E+06	4.62E+06	6.42E+06	5.68E+06	4.22E+06	3.58E+06	5.01E+06		22.07	22.25	22.45	22.65	22.64	22.29	21.71	22.03		22.25	22.64	21.87	0.192538	0.206487	0.228567			
2238	Q14766	Latent-trar	LTBP1	21	16.9	1.99E+07	1.28E+07	1.01E+07	2.42E+07	1.68E+07	1.64E+07	1.94E+07	1.72E+07		24.22	23.8	23.54	24.58	24.18	24.19	24.2	23.82		23.8	24.19	24.01	0.342456	0.227247	0.268935	+		
1028	J3QQY2	Transmem	TMCO1	2	22.1		1.46E+06	6.28E+06	1.76E+06	4.41E+06	3.98E+06					20.76	22.88	20.73	22.26	22.21				21.82	22.21		1.500801	0.867815				
2220	Q14331	Protein FR	FRG1	2	9.7		4.55E+06	3.91E+06	6.60E+06	6.00E+06	5.21E+06					22.37	22.23	22.68	22.71	22.58				22.3	22.68		0.096109	0.071622				
1401	P05387	60S acidic r	RPLP2	5	73	5.62E+07	5.26E+07	6.40E+07	9.41E+07	7.12E+07	4.91E+07	6.83E+07	7.60E+07	8.11E+07	25.72	25.87	26.22	26.63	26.25	25.75	25.93	25.99	25.73	25.87	26.25	25.93	0.258476	0.439685	0.133228	+		
2436	Q6P1M0	Long-chain	SLC27A4	7	13.5	6.56E+06	5.02E+06	5.28E+06	8.12E+06	7.25E+06	5.79E+06	3.49E+06	8.58E+06	7.01E+06	22.57	22.49	22.66	22.96	22.98	22.74	21.67	22.81	22.23	22.57	22.96	22.23	0.084341	0.131377	0.572315			
2969	Q9H9A6	Leucine-ric	LRRC40	13	27.1	1.70E+07	1.71E+07	1.45E+07	2.10E+07	1.99E+07	1.54E+07	2.44E+07	2.22E+07	2.45E+07	23.98	24.22	24.01	24.39	24.41	24.11	24.54	24.22	24.09	24.01	24.39	24.22	0.129338	0.172029	0.228716	+		
2456	Q6UN15-4	Pre-mRNA	FIP1L1	4	16.7	5.80E+06	4.91E+06	5.57E+06	6.43E+06	6.57E+06	6.39E+06				22.39	22.46	22.74	22.66	22.84	22.86				22.46	22.84		0.186448	0.113786				
706	H7C5A7	Acyl-coenz	ACOT8	2	12			2.74E+06			3.59E+06			8.61E+06			21.68			22.06			22.55	21.68	22.06	22.55						+
1926	P55735	Protein SE	SEC13	7	34.8	2.87E+07	2.74E+07	2.18E+07	2.60E+07	3.31E+07	3.05E+07	2.03E+07	3.00E+07	2.13E+07	24.73	24.88	24.61	24.69	25.15	25.12	24.29	24.67	23.95	24.73	25.12	24.29	0.131294	0.254243	0.363075			
373	K7EMQ8	SWI/SNF-r	SMARCE1	3	22.6	8.41E+06	1.05E+07	1.45E+07	1.84E+07	1.26E+07	1.34E+07	1.10E+07			22.96	23.5	24.01	24.19	23.74	23.89	23.3			23.5	23.89	23.3	0.525201	0.228315		+		
2052	Q00325-2	Phosphate	SLC25A3	11	33.5	1.14E+08	1.45E+08	1.28E+08	1.09E+08	2.01E+08	1.55E+08	1.05E+08	1.27E+08		26.87	27.47	27.28	26.8	27.71	27.66	26.68	26.71		27.28	27.66	26.69	0.311542	0.512648	0.016885			
1480	P11172	Uridine 5-r	UMPS	11	34.2	1.01E+07	1.07E+07	1.26E+07	1.44E+07	1.71E+07	1.36E+07	1.69E+07	1.16E+07	1.17E+07	23.25	23.53	23.84	23.81	24.2	23.91	23.98	23.24	23.07	23.53	23.91	23.24	0.29559	0.203691	0.487729			
1699	P33993	DNA replic	MCM7	34	53.1	1.79E+08	1.55E+08	1.70E+08	2.23E+08	2.35E+08	2.07E+08	1.43E+08	1.61E+08	1.10E+08	27.51	27.6	27.77	27.79	27.97	28.06	27.11	27.04	26.14	27.6	27.97	27.04	0.136212	0.137357	0.541289			
2031	P78346	Ribonuclea	RPP30	7	34	1.65E+07	1.91E+07	1.76E+07	2.56E+07	2.71E+07	1.89E+07	2.46E+07	2.73E+07	2.51E+07	23.93	24.35	24.3	24.68	24.88	24.42	24.55	24.5	24.13	24.3	24.68	24.5	0.22606	0.229857	0.228932	+		
474	F8WF90	PRA1 famil	ARL6IP5	2	33.9	3.30E+06	2.42E+06				3.24E+06			5.07E+06	21.63	21.47				21.92			21.71	21.55	21.92	21.71	0.110871			+		
868	Q5QPK2	Dolichol-pl	DPM1	9	38	2.00E+07	2.34E+07	1.94E+07	2.52E+07	3.13E+07	2.59E+07	1.63E+07	2.36E+07	2.02E+07	24.22	24.63	24.43	24.65	25.04	24.8	23.93	24.32	23.88	24.43	24.8	23.93	0.20557	0.198225	0.238876			
3007	Q9NR12	PDZ and LI	PDLIM7	3	7.4		2.83E+06	2.85E+06	4.02E+06	3.85E+06	4.65E+06					21.66	21.75	21.94	22.08	22.41				21.7	22.08		0.068347	0.239121				
1911	P54132	Bloom syn	BLM	64	51.8	1.51E+09	9.59E+08	1.05E+09	1.43E+09	1.96E+09	1.50E+09	1.18E+09	1.68E+09	1.52E+09	30.89	30.51	30.71	30.63	31.09	31.1	30.17	30.67	29.72	30.71	31.09	30.17	0.191728	0.270088	0.476846			
2766	Q96PK6	RNA-bindir	RBM14	5	8.7		2.34E+06	2.39E+06			3.04E+06					21.42	21.49			21.83				21.45	21.83		0.049246					
1464	P0DN76	Splicing fac	U2AF1	5	26.7	5.57E+07	4.74E+07	4.72E+07	4.66E+07	8.22E+07	6.08E+07	8.14E+07	9.55E+07	6.61E+07	25.7	25.65	25.77	25.61	26.48	26.08	26.22	26.3	25.44	25.7	26.08	26.22	0.058642	0.434078	0.479034			
1050	K7ELQ0	39S riboso	MRPL4	4	26.4		3.83E+06	3.65E+06	5.56E+06	5.51E+06	5.02E+06	4.87E+06		5.14E+06		22.16	22.13	22.42	22.6	22.52	22.16		21.74	22.14	22.52	21.95	0.019704	0.092656	0.295412	+		
601	D6REM4	Casein kina	CSNK1A1	2	9.7			2.31E+06			3.03E+06		2.56E+06	2.35E+06			21.45			21.82		21.1	20.53	21.45	21.82	20.82			0.404076			
1539	P16989-2	Y-box-bind	YBX3	3	45.2		3.13E+06	2.09E+06		3.01E+06	3.81E+06	2.41E+06	2.69E+06			21.83	21.32		21.74	22.15	21.12	21.2		21.57	21.94	21.16	0.358224	0.288908	0.057839			
2271	Q15233	Non-POU c	NONO	15	41.6	2.37E+08	1.14E+08	1.36E+08	2.23E+08	2.15E+08	1.39E+08	1.54E+08	1.37E+08	3.54E+08	27.92	27.04	27.42	27.79	27.83	27.48	27.21	26.8	27.54	27.42	27.79	27.21	0.438897	0.190538	0.37202	+		
247	A0A0D9SFI	ATP-depen	DDX3X	26	49.1	1.80E+08	1.52E+08	1.78E+08	2.40E+08	2.14E+08	2.11E+08	1.72E+08	1.78E+08	1.36E+08	27.52	27.56	27.83	27.93	27.82	28.07	27.34	27.21	26.36	27.56	27.93	27.21	0.167375	0.126938	0.531979			
1722	P36404	ADP-ribosy	ARL2	8	54.9	2.28E+07	2.15E+07	2.16E+07	3.04E+07	3.57E+07	2.66E+07	1.36E+07	1.85E+07	2.05E+07	24.39	24.51	24.6	24.88	25.26	24.87	23.64	23.95	23.9	24.51	24.88	23.9	0.106187	0.223571	0.164217			
2199	Q13895	Bystin	BYSL	3	8.2		1.70E+06	1.54E+06			2.03E+06					20.97	20.87			21.28				20.92	21.28		0.074847					+
1242	O60884	DnaJ homo	DNAJA2	6	14.3	1.26E+07	1.68E+07	1.66E+07	1.87E+07	2.78E+07	2.08E+07	2.49E+07	2.30E+07	2.10E+07	23.56	24.18	24.22	24.2	24.91	24.54	24.57	24.26	23.92	24.18	24.54	24.26	0.369373	0.35321	0.326898	+		
728	E9PP21	Cysteine ar	CSRP1	2	23.1	6.88E+06	3.69E+06	3.08E+06	5.36E+06	5.21E+06					22.64	22.08	21.87	22.36	22.53					22.08	22.44		0.399191	0.119501				
1092	O00148	ATP-depen	DDX39A	17	45	1.87E+08	1.74E+08	1.66E+08	2.11E+08	2.66E+08	2.02E+08	1.65E+08	2.11E+08	1.74E+08	27.61	27.78	27.68	27.73	28.13	28.04	27.28	27.42	26.61	27.68	28.04	27.28	0.084638	0.210067	0.430305			
3021	Q9NSP4-4	Centromer	CENPM	3	27.1	2.04E+06	1.49E+06	1.66E+06	2.31E+06	2.13E+06	2.10E+06				20.88	20.79	20.98	21.15	21.24	21.33				20.88	21.24		0.094502	0.092155				
401	E7ESU0	Ubiquitin c	USP19	6	7.3	1.05E+06	8.93E+05	1.56E+06	1.06E+06	1.16E+06	1.64E+06				19.95	20.03	20.88	20.2	20.39	20.93				20.03	20.39		0.511638	0.378485				
2764	Q96P63	Serpin B12	SERPINB12	3	8.6	3.79E+06	4.27E+06	4.56E+06	1.14E+07		3.07E+06	3.66E+06	3.01E+06		21.83	22.29	22.43	23.43		21.85	21.75	21.37		22.29	22.64	21.56	0.315944	1.122956	0.263961			
697	E9PH82	Protein FA	FAM98A	4	20.8	8.43E+06	4.80E+06	6.17E+06	9.65E+06	6.10E+06	1.44E+07	6.04E+06	9.70E+06		22.96	22.44	22.86	23.22	22.74	24	22.5	22.98		22.86	23.22	22.74	0.277689	0.635637	0.341117	+		
1034	J3QTA2	BAG family	BAG1	4	15.7		2.70E+06	2.69E+06	2.08E+06	3.61E+06	4.10E+06					21.59	21.65	20.99	21.97	22.25				21.62	21.97		0.040992	0.663051				
1224	O60506-4	Heterogen	SYNCRIP	21	38.7	7.07E+07	8.63E+07	1.22E+08	7.50E+07	1.09E+08	1.34E+08	9.04E+07	9.06E+07	2.35E+07	26.08	26.57	27.23	26.26	26.93	27.42	26.42	26.25	24.04	26.57	26.93	26.25	0.576911	0.579494	1.3278			
2280	Q3LIE7	Delta(24)-s	Nbla03646	2	5.2			5.30E+06			7.08E+06	8.56E+06	5.02E+06	7.27E+06			22.67			23.02	22.97	22.03	22.3	22.67	23.02	22.3			0.482739			
3220	Q9Y3I0	tRNA-splici	RTCB	3	7.5			2.01E+06			2.58E+06						21.27			21.62				21.27	21.62							
1005	J3KPX7	Prohibitin-	PHB2	14	54.7	4.55E+08	3.57E+08	2.67E+08	4.89E+08	5.23E+08	4.81E+08	4.15E+08	4.76E+08	3.34E+08	28.83	28.85	28.62	28.82	29.19	29.17	28.58	28.38	27.48	28.83	29.17	28.38	0.126342	0.212182	0.585098			
365	B2R4S9	Histone H2	HIST1H2BI	8	52.4	5.36E+08	3.78E+08	3.49E+08	7.12E+08	6.03E+08	4.32E+08	1.43E+09	1.39E+09	1.03E+09	28.98	28.95	29.02	29.38	29.32	29.02	30.66	30.29	29.3	28.98	29.32	30.29	0.032193	0.19575	0.704736			+
2583	Q8NC51-4	Plasminoge	SERBP1	7	23.8	4.24E+07	6.28E+07	7.06E+07	6.15E+07	9.93E+07	7.90E+07	4.89E+07	5.60E+07	3.34E+07	25.35	26.15	26.43	25.95	26.83	26.5	25.52	25.56	24.56	26.15	26.5	25.52	0.563752	0.447634	0.564288			
2383	Q5T160	Probable a	RARS2	4	8		2.07E+06	2.04E+06		2.61E+06	2.78E+06	2.22E+06	2.98E+06			21.29	21.29		21.54	21.73	21	21.36		21.29	21.63	21.18	0.001084	0.131854	0.253581	+		
2611	Q8TCJ2	Dolichyl-di	STT3B	7	9.4	1.23E+07	1.01E+07	9.15E+06	1.92E+07	1.30E+07	1.21E+07	1.38E+07	1.03E+07	8.43E+06	23.52	23.45	23.4	24.24	23.79	23.71	23.68	23.07	22.53	23.45	23.79	23.07	0.06356	0.282629	0.573172			
2295	Q15654	Thyroid rec	TRIP6	9	32.4	7.39E+06	8.65E+06	6.26E+06	1.37E+07	8.57E+06	8.12E+06	8.94E+06	1.28E+07	8.29E+06	22.74	23.22	22.88	23.73	23.18	23.22	23.04	23.4	22.5	22.88	23.22	23.04	0.246992	0.303865	0.452597	+		
1368	P01876	Ig alpha-1 c	IGHA1	3	10.2	9.50E+06	2.06E+06	2.90E+06	9.55E+06	3.95E+06	2.51E+06	4.08E+06	2.81E+06	5.68E+06	23.15	21.28	21.77	23.19	22.11	21.57	21.93	21.29	21.88	21.77	22.11	21.88	0.968275	0.826564	0.359521	+		
1020	J3KTA4	Probable A	DDX5	22	38.6	1.61E+08	1.21E+08	1.45E+08	1.93E+08	1.95E+08	1.70E+08	1.24E+08	1.16E+08	8.24E+07	27.31	27.21	27.52	27.6	27.65	27.76	26.91	26.57	25.76	27.31	27.65	26.57	0.159209	0.082724	0.591206			
2934	Q9H3G5	Probable s	CPVL	4	9.5		3.11E+06	2.22E+06	3.75E+06	3.55E+06	3.73E+06					21.82	21.42	21.85	21.96	22.12				21.62	21.96		0.286213	0.137899				
1379	P04080	Cystatin-B	CSTB	4	53.1	1.62E+07	1.11E+07	1.27E+07	1.90E+07	1.25E+07	1.64E+07	6.87E+06	1.08E+07		23.91	23.59	23.85	24.23	23.72	24.19	22.66	23.13		23.85	24.19	22.9	0.169463	0.281512	0.33325			
2187	Q13618-2	Cullin-3	CUL3	4	7.4			1.69E+06		2.33E+06							21.04		21.37					21.04	21.37							
1030	J3QRG6	Cyclin-dep	CDKN2A	5	50.7	9.27E+06	1.13E+07	1.55E+07	1.13E+07	1.47E+07	1.44E+07	6.89E+06	9.13E+06		23.11	23.62	24.11	23.42	23.96	24	22.67	22.89		23.62	23.96	22.78	0.49875	0.324067	0.158749			
1885	P52294	Importin su	KPNA1	5	11.9	4.22E+06	3.73E+06	4.90E+06	5.06E+06	4.97E+06	6.01E+06	3.66E+06	4.16E+06		21.97	22.11	22.54	22.28	22.44	22.78	21.75	21.8		22.11	22.44	21.77	0.295128	0.254912	0.03581			
1150	O15013-7	Rho guanir	ARHGEF10	25	24.5	4.79E+07	2.50E+07	3.37E+07	4.46E+07	4.19E+07	4.03E+07	2.66E+07	1.97E+07	1.63E+07	25.47	24.73	25.19	25.54	25.48	25.52	24.66	24.05	23.59	25.19	25.52	24.05	0.377339	0.032845	0.535056			
229	E9PIY1	Protein kin	PACSIN3	3	11.6		1.19E+06	1.26E+06	1.84E+06	1.64E+06	2.13E+06	1.93E+06				20.49	20.66	20.82	20.91	21.35	20.78			20.58	20.91	20.78	0.123838	0.28846		+		
2411	Q5VYS8	Terminal u	ZCCHC6	38	28.8	9.08E+07	5.78E+07	6.33E+07	8.57E+07	8.39E+07	7.95E+07	3.52E+07	5.46E+07	2.32E+07	26.51	26.04	26.18	26.5	26.52	26.52	25.07	25.52	24.02	26.18	26.52	25.07	0.242915	0.011088	0.770618			
762	F5H1S9	tRNA pseu	PUS1	5	14.2	6.79E+06	5.63E+06	5.64E+06	3.91E+06	7.27E+06	6.83E+06	3.12E+06	5.59E+06		22.62	22.62	22.77	21.9	22.98	22.95	21.52	22.19		22.62	22.95	21.86	0.086517	0.615728	0.474659			
2320	Q16649	Nuclear fac	NFIL3	2	6.1	3.20E+06	2.05E+06	1.99E+06	3.00E+06	2.70E+06	2.58E+06	3.27E+06			21.58	21.27	21.26	21.54	21.6	21.62	21.6			21.27	21.6	21.6	0.183609	0.04074			+	
3116	Q9UG63-2	ATP-bindin	ABCF2	12	21.3	2.86E+07	2.52E+07	2.36E+07	3.20E+07	3.88E+07	2.94E+07	1.80E+07	1.58E+07	1.65E+07	24.72	24.75	24.74	25.02	25.36	25.06	24.08	23.72	23.61	24.74	25.06	23.72	0.013897	0.183602	0.243053			
3207	Q9Y305-2	Acyl-coenz	ACOT9	12	25.1	2.91E+07	2.71E+07	2.37E+07	3.86E+07	3.20E+07	2.68E+07	3.85E+07	4.03E+07	3.36E+07	24.76	24.86	24.75	25.29	25.09	24.88	25.19	25.06	24.57	24.76	25.09	25.06	0.059727	0.207098	0.324551		+	

947	H7BXK9	ATP-bindin	ABCB6	5	10		1.74E+06	2.82E+06	3.19E+06	3.60E+06	2.74E+06	5.31E+06	4.61E+06	5.69E+06		21.02	21.73	21.63	21.97	21.71	22.31	21.92	21.9	21.38	21.71	21.92	0.502928	0.176605	0.232474			
2936	Q9H3M7-2	Thioredoxi	TXNIP	2	8	2.25E+06	1.25E+06	1.90E+06	2.38E+06	3.05E+06	2.12E+06	2.27E+06	2.52E+06	2.18E+06	21.02	20.59	21.21	21.21	21.76	21.35	21.04	21.08	20.39	21.02	21.35	21.04	0.320142	0.286277	0.38789	+		
1208	O43913	Origin recc	ORC5	5	12.2	5.02E+06	4.77E+06	4.94E+06	5.19E+06	6.94E+06	5.88E+06	8.71E+06	7.48E+06	4.24E+06	22.18	22.43	22.55	22.31	22.93	22.76	23	22.62	21.35	22.43	22.76	22.62	0.186845	0.319086	0.860048	+		
1489	Q5HY81	Ubiquitin-l	UBL4A	2	10			1.57E+06			1.93E+06						20.89			21.21				20.89	21.21							
1915	P54652	Heat shock	HSPA2	7	30	1.30E+07	1.01E+07	9.27E+06	1.41E+07	1.04E+07	1.34E+07	1.17E+07	9.16E+06	1.33E+07	23.6	23.45	23.41	23.78	23.47	23.89	23.39	22.9	23.27	23.45	23.78	23.27	0.095634	0.218014	0.257013	+		
1579	P21980	Protein-glu	TGM2	3	6.6	2.94E+06	2.73E+06	3.59E+06	3.77E+06	3.50E+06	3.25E+06	4.44E+06	4.28E+06		21.47	21.61	22.09	21.85	21.93	21.93	22.05	21.83		21.61	21.93	21.94	0.326075	0.044972	0.155912		+	
2351	Q52LJ0	Protein FAI	FAM98B	6	23.6	1.37E+07	1.05E+07	1.14E+07	1.59E+07	1.14E+07	1.44E+07	1.05E+07	1.07E+07		23.65	23.5	23.72	23.97	23.59	24	23.25	23.12		23.65	23.97	23.18	0.110033	0.230215	0.092712			
275	A0A0R4J2E	Matrin-3	MATR3	12	21.6	2.32E+07	2.18E+07	3.03E+07	1.48E+07	2.69E+07	2.92E+07	3.42E+07	9.42E+06		24.41	24.54	25.06	23.85	24.86	25.04	25.01	22.93		24.54	24.86	23.97	0.343011	0.641393	1.471537			
1072	M0QXS5	Heterogen	HNRNPL	14	44.2	5.43E+07	8.17E+07	9.14E+07	6.35E+07	1.02E+08	1.35E+08	7.75E+07	1.33E+08	1.30E+07	25.67	26.52	26.81	26.01	26.84	27.43	26.12	26.76	23.22	26.52	26.84	26.12	0.592487	0.716652	1.883752			
2233	Q14692	Ribosome	BMS1	21	21	3.02E+07	2.97E+07	4.54E+07	2.17E+07	3.98E+07	3.39E+07	2.19E+07	1.73E+07	1.05E+07	24.85	24.97	25.69	24.44	25.39	25.28	24.39	23.83	22.89	24.97	25.28	23.83	0.456931	0.517724	0.759743			
626	E5RJR5	S-phase kir	SKP1	4	16.6	1.63E+07	1.65E+07	2.03E+07	2.16E+07	2.41E+07	9.85E+06	1.69E+07	2.23E+07	1.07E+07	23.92	24.12	24.49	24.44	24.64	23.47	23.98	24.23	22.92	24.12	24.44	23.98	0.289546	0.624192	0.695628	+		
2443	Q6PCE3	Glucose 1,(	PGM2L1	6	12.7	4.76E+06	4.70E+06	5.75E+06	5.57E+06	6.20E+06	5.76E+06	1.27E+07			22.1	22.41	22.78	22.42	22.78	22.73	23.52			22.41	22.73	23.52	0.340671	0.19205				+
166	F8W8M4	Actin-bindi	ABLIM1	7	14.3	9.81E+06	8.32E+06	8.02E+06	1.19E+07	1.35E+07	1.02E+07	8.21E+06	5.45E+06		23.19	23.17	23.23	23.5	23.84	23.5	22.9	22.14		23.19	23.5	22.52	0.029775	0.193309	0.544006			
2592	Q8NEK8	Protein FAI	FAM46D	2	6.9	2.23E+06	1.76E+06	1.58E+06	2.52E+06	2.25E+06	1.98E+06	2.55E+06			21	21.04	20.9	21.31	21.31	21.24	21.2			21	21.31	21.2	0.074202	0.041649			+	
2790	Q96SZ6-5	CDK5 regul	CDK5RAP1	2	6.6			1.65E+06			2.19E+06						20.97			21.28				20.97	21.28							
2706	Q96AV8-3	Transcripti	E2F7	33	48.3	2.90E+08	2.05E+08	1.61E+08	3.40E+08	3.39E+08	2.27E+08	3.91E+08	3.87E+08	3.99E+08	28.12	28.03	27.63	28.34	28.46	28.12	28.43	28.09	27.76	28.03	28.34	28.09	0.262666	0.168634	0.335899	+		
1768	P42167	Lamina-ass	TMPO	5	41.2	1.28E+07	6.32E+06	6.77E+06	1.01E+07	9.07E+06	6.90E+06	5.77E+06	4.44E+06		23.58	22.77	22.95	23.27	23.26	22.98	22.43	21.87		22.95	23.26	22.15	0.423691	0.165551	0.396035			
1297	O94776	Metastasis	MTA2	18	29.8	9.05E+07	5.49E+07	5.91E+07	8.15E+07	8.18E+07	7.00E+07	8.25E+07	8.19E+07	4.90E+07	26.5	25.96	26.08	26.39	26.47	26.31	26.26	26.1	25.07	26.08	26.39	26.1	0.283877	0.083418	0.644806	+		
1190	O43318-2	Mitogen-ac	MAP3K7	16	30.1	7.90E+07	6.73E+07	6.76E+07	8.15E+07	1.08E+08	8.23E+07	8.03E+07	6.99E+07	3.90E+07	26.26	26.26	26.35	26.39	26.91	26.57	26.21	25.85	24.75	26.26	26.57	25.85	0.053392	0.266631	0.763525			
168	A0A0A0MF	Kynurenine	CCBL2	2	9.2	1.60E+06	1.97E+06	2.12E+06			2.45E+06				20.51	21.22	21.34			21.52				21.22	21.52		0.45119					
906	S4R329	ER membra	EMC4	3	54.2		9.07E+05	1.36E+06			1.36E+06					20.07	20.72			20.7				20.4	20.7		0.458462			+		
3012	Q9NRF9	DNA polym	POLE3	2	18.4	2.42E+06	3.08E+06				2.91E+06		2.02E+06		21.16	21.81				21.79		20.81		21.49	21.79	20.81	0.455648					
1714	P35610	Sterol O-ac	SOAT1	3	7.5	4.76E+06	7.61E+06	7.38E+06	6.36E+06	1.05E+07	9.00E+06	3.26E+06	7.45E+06		22.1	23.04	23.09	22.63	23.48	23.34	21.59	22.59		23.04	23.34	22.09	0.558683	0.454886	0.711128			
1144	O14929	Histone ac	HAT1	6	24.6	1.38E+07	1.11E+07	1.25E+07	8.94E+06	1.47E+07	1.51E+07	1.13E+07	1.44E+07		23.66	23.59	23.82	23.09	23.96	24.07	23.34	23.58		23.66	23.96	23.46	0.118323	0.538831	0.173322	+		
3137	Q9UID3-2	Vacuolar p	VPS51	4	7.6		3.31E+06	2.92E+06	4.52E+06	4.79E+06	3.82E+06	5.05E+06	5.46E+06	5.99E+06		21.93	21.78	22.1	22.4	22.15	22.22	22.14	21.96	21.85	22.15	22.14	0.102726	0.157316	0.132872		+	
944	H3BU53	MAX gene-	MGA	3	27.7	7.84E+06	1.05E+07	7.88E+06	1.18E+07	8.62E+06	1.16E+07	5.30E+06	8.97E+06		22.83	23.5	23.19	23.49	23.19	23.65	22.3	22.87		23.19	23.49	22.58	0.338624	0.232362	0.398452	+		
3205	Q9Y2X3	Nucleolar p	NOP58	6	16.3		3.11E+06	3.27E+06	4.76E+06	4.17E+06	4.50E+06	4.33E+06	3.70E+06			21.82	21.94	22.18	22.16	22.37	22.02	21.63		21.88	22.18	21.82	0.087588	0.11853	0.277048	+		
1282	O75794	Cell divisio	CDC123	7	23.2	1.03E+07	9.63E+06	7.13E+06	1.15E+07	1.19E+07	1.09E+07	8.90E+06	1.39E+07		23.28	23.38	23.03	23.45	23.64	23.58	23.03	23.51		23.28	23.58	23.27	0.182325	0.098568	0.337824	+		
2938	Q9H3P7	Golgi resid	ACBD3	5	10.6	3.19E+06	3.19E+06	4.56E+06	2.73E+06	4.18E+06	4.15E+06				21.57	21.87	22.43	21.41	22.17	22.27				21.87	22.17		0.437539	0.470066				
48	A0A087X0	NF-kappa-I	IKBKG	5	15.1	4.79E+06	2.83E+06	2.92E+06		4.51E+06	3.06E+06	3.93E+06			22.11	21.66	21.78		22.32	21.84	21.86			21.78	22.08	21.86	0.235429	0.336764		+		
1630	P27105	Erythrocyt	STOM	7	31.9	2.99E+07	2.10E+07	1.74E+07	2.73E+07	2.30E+07	2.74E+07	4.00E+07	4.46E+07	4.49E+07	24.82	24.46	24.28	24.76	24.59	24.94	25.25	25.19	24.94	24.46	24.76	25.19	0.27372	0.176617	0.165391			+
1075	M0R0N4	AP-2 comp	AP2S1	4	27.1	1.22E+07	9.84E+06	1.19E+07	9.76E+06	1.31E+07	1.26E+07	1.10E+07	9.33E+06		23.51	23.41	23.75	23.22	23.81	23.81	23.3	22.92		23.51	23.81	23.11	0.175556	0.335426	0.26527			
1325	O95433	Activator o	AHSA1	13	48.5	9.28E+07	7.21E+07	6.73E+07	9.58E+07	9.76E+07	7.72E+07	9.07E+07	1.07E+08	1.06E+08	26.57	26.35	26.34	26.65	26.78	26.48	26.43	26.43	26.1	26.35	26.65	26.43	0.12843	0.151771	0.191538	+		
3043	Q9NVV4-2	Poly(A) RN	MTPAP	6	11.2	8.11E+06	7.64E+06	7.17E+06	8.74E+06	9.84E+06	8.99E+06	1.20E+07	1.10E+07	1.62E+07	22.91	23.05	23.04	23.05	23.38	23.34	23.42	23.15	23.58	23.04	23.34	23.42	0.080083	0.179006	0.215711			
309	A6NEM5	GPI-anchor	PIGK	4	14.8		6.73E+06	6.67E+06	6.65E+06	1.05E+07	7.93E+06	1.45E+07				22.86	22.93	22.69	23.48	23.19	23.76			22.9	23.19	23.76	0.051267	0.400114				+
1991	P62699	Protein yip	YPEL5	3	29.8	1.50E+07	1.15E+07	1.09E+07	1.79E+07	8.38E+06	1.38E+07	1.11E+07	1.61E+07	1.29E+07	23.79	23.64	23.64	24.14	23.16	23.94	23.31	23.74	23.21	23.64	23.94	23.31	0.085175	0.513998	0.280474			
2529	Q8IVH2-2	Forkhead b	FOXP4	23	53.8	3.09E+08	2.48E+08	2.52E+08	4.03E+08	3.84E+08	2.74E+08	4.53E+08	3.52E+08	2.25E+08	28.21	28.3	28.56	28.59	28.61	28.37	28.72	27.96	26.99	28.3	28.59	27.96	0.184317	0.136261	0.866891			
2136	Q12800-2	Alpha-glob	TFCP2	2	6.7	1.68E+06	1.49E+06	1.95E+06	2.20E+06	1.76E+06	3.20E+06				20.56	20.79	21.24	21.08	21.02	21.91				20.79	21.08		0.34931	0.499857				
1833	P49590-2	Probable h	HARS2	4	9.1	1.82E+06	1.67E+06	3.10E+06		1.88E+06	2.21E+06	2.54E+06			20.72	20.95	21.88		21.08	21.41	21.19			20.95	21.24	21.19	0.614386	0.228444			+	
3087	Q9P270	SLAIN moti	SLAIN2	15	39.6	1.24E+08	1.17E+08	1.12E+08	1.50E+08	1.75E+08	1.27E+08	1.73E+08	1.40E+08	5.49E+07	26.93	27.11	27.06	27.35	27.54	27.32	27.36	26.84	25.2	27.06	27.35	26.84	0.093959	0.118049	1.127434			
1482	P11182	Lipoamide	DBT	6	15.1	7.43E+06	7.02E+06	5.37E+06	8.66E+06	7.45E+06	7.22E+06	9.12E+06	8.28E+06		22.75	22.94	22.69	23.04	23.02	23.04	23.07	22.77		22.75	23.04	22.92	0.132702	0.01065	0.212146	+		
2648	Q8WXF1	Paraspeckl	PSPC1	14	32.3	3.50E+08	2.24E+08	2.51E+08	4.88E+08	4.15E+08	2.88E+08	5.06E+08	5.33E+08	4.53E+08	28.47	28.18	28.54	28.79	28.75	28.54	28.93	28.58	28.13	28.47	28.75	28.58	0.194219	0.133882	0.399821	+		
2343	Q3ZCQ8	Mitochond	TIMM50	5	21	3.00E+07	2.43E+07	2.14E+07	3.15E+07	4.30E+07	2.75E+07	3.14E+07	4.45E+07	4.09E+07	24.83	24.68	24.59	24.97	25.51	24.95	24.89	25.19	24.83	24.68	24.97	24.89	0.123153	0.317348	0.192079		+	
3055	Q9NYF8-2	Bcl-2-assoc	BCLAF1	38	36.9	1.39E+09	7.38E+08	1.32E+09	1.56E+09	1.54E+09	1.68E+09	1.25E+09	1.31E+09	2.34E+09	30.69	29.95	31.08	30.81	30.98	31.41	30.38	30.16	30.06	30.69	30.98	30.16	0.574096	0.305287	0.166539			
2368	Q5JUX0	Spindlin-3	SPIN3	5	43	2.60E+07	1.18E+07	1.22E+07	2.53E+07	1.57E+07	1.45E+07	1.91E+07	3.40E+07	5.90E+07	24.58	23.69	23.78	24.66	24.06	24.02	24.18	24.85	25.28	23.78	24.06	24.85	0.489495	0.358331	0.552942			+
2023	P68036-2	Ubiquitin-c	UBE2L3	4	39.3	5.77E+06	1.96E+07	2.90E+07	8.90E+06	2.44E+07	2.65E+07	4.07E+06			22.38	24.38	25	23.08	24.66	24.86	21.92			24.38	24.66	21.92	1.367406	0.972854				
3204	Q9Y2X0-2	Mediator c	MED16	4	5.8	4.59E+06	2.84E+06	3.22E+06	4.15E+06	4.26E+06	4.30E+06	4.00E+06			22.06	21.66	21.92	21.98	22.2	22.31	21.88			21.92	22.2	21.88	0.202437	0.164024		+		
171	A0A0A0MS	Sideroflexi	SFXN3	5	21.2	6.41E+06	3.48E+06	4.59E+06	6.80E+06	5.66E+06	6.01E+06	6.35E+06	9.94E+06	1.03E+07	22.55	22	22.44	22.72	22.63	22.78	22.56	23.02	22.85	22.44	22.72	22.85	0.290066	0.078171	0.233093			
1696	P33897	ATP-bindin	ABCD1	9	14.9	1.43E+07	7.40E+06	8.24E+06	1.21E+07	1.13E+07	8.92E+06	1.24E+07	1.21E+07	1.28E+07	23.72	23.01	23.26	23.54	23.57	23.32	23.48	23.34	23.19	23.26	23.54	23.34	0.359343	0.135191	0.144221	+		
2917	Q9H0S4-2	Probable A	DDX47	5	13.5	6.81E+06	5.20E+06	5.30E+06	7.77E+06	7.86E+06	5.36E+06	6.17E+06	6.94E+06	6.43E+06	22.63	22.51	22.67	22.91	23.08	22.62	22.53	22.49	22.12	22.63	22.91	22.49	0.080157	0.229283	0.224732	+		
2293	Q15643	Thyroid rec	TRIP11	29	18	9.14E+07	1.58E+07	1.39E+07	2.07E+07	2.62E+07	1.37E+07	5.26E+07	2.11E+07	4.76E+07	26.55	24.09	23.95	24.36	24.8	23.93	25.61	24.15	25.04	24.09	24.36	25.04	1.461427	0.436606	0.733115			+
1917	P54886-2	Delta-1-pyr	ALDH18A1	23	32.3	5.80E+07	4.95E+07	6.01E+07	5.57E+07	6.01E+07	7.23E+07	5.32E+07	5.31E+07	4.03E+07	25.76	25.72	26.12	25.82	26.03	26.37	25.62	25.49	24.81	25.76	26.03	25.49	0.217419	0.276482	0.438274			
2301	Q15758	Neutral am	SLC1A5	8	18.7	2.44E+07	2.15E+07	2.34E+07	2.70E+07	3.15E+07	2.56E+07	2.56E+07	2.20E+07	3.20E+07	24.5	24.51	24.73	24.74	25.06	24.79	24.62	24.2	24.46	24.51	24.79	24.46	0.126454	0.170056	0.210068	+		
1681	P31749	RAC-alpha	AKT1	4	10.6	3.63E+06	3.03E+06	3.08E+06	3.70E+06	4.32E+06	3.53E+06	3.68E+06	3.72E+06		21.75	21.78	21.87	21.83	22.23	22.05	21.75	21.63		21.78	22.05	21.69	0.060601	0.200039	0.084453	+		
1698	P33992	DNA replic	MCM5	34	50.5	1.63E+08	1.16E+08	1.24E+08	1.83E+08	1.80E+08	1.42E+08	1.19E+08	1.27E+08	1.25E+08	27.34	27.09	27.25	27.52	27.58	27.5	26.85	26.71	26.28	27.25	27.52	26.71	0.127175	0.044575	0.29461			
45	E7ETK0	40S riboso	RPS24	5	35.9	3.00E+07	3.35E+07	3.70E+07	3.54E+07	4.18E+07	3.86E+07	5.69E+07		3.36E+07	24.83	25.18	25.4	25.19	25.47	25.45	25.72		24.57	25.18	25.45	25.15	0.289489	0.158414	0.810205	+		
2425	Q6FI81-3	Anamorsin	CIAPIN1	4	21.1	5.95E+06	5.78E+06	4.70E+06			5.80E+06		4.72E+06	4.37E+06	22.43	22.64	22.47			22.74		21.96	21.4	22.47	22.74	21.68	0.112445		0.393643			
1065	K7ERI7	60S riboso	RPL22	5	44.2	4.44E+07	5.27E+07	3.97E+07	5.86E+07	3.70E+07	4.92E+07	2.18E+07	5.55E+07		25.4	25.88	25.49	25.9	25.33	25.76	24.38	25.54		25.49	25.76	24.96	0.252392	0.299597	0.825594			
305	A6ND22	28S riboso	MRPS16	2	21.2		2.30E+06				2.68E+06					21.4				21.66				21.4	21.66							
2037	P81605	Dermcidin	DCD	6	53.6	2.04E+08	8.79E+07	4.09E+07	2.44E+08	1.07E+08	2.19E+07	1.05E+08	5.50E+08	2.25E+08	27.74	26.63	25.54	27.98	26.9	24.59	26.68	28.65	26.99	26.63	26.9	26.99	1.100882	1.729791	1.057117			
3039	Q9NVI7-2	ATPase far	ATAD3A	15	27	5.81E+07	4.04E+07	4.97E+07	6.48E+07	5.84E+07	6.66E+07	4.59E+07	3.75E+07	2.35E+07	25.77	25.46	25.87	26.03	25.98	26.23	25.41	24.98	24.04	25.77	26.03	24.98	0.210629	0.1321	0.696697			
2420	Q68CQ7	Glycosyltra	GLT8D1	3	10	2.07E+06	2.25E+06	1.89E+06	2.19E+06	2.47E+06	2.49E+06	5.30E+06	3.78E+06	3.79E+06	20.91	21.37	21.2	21.07	21.46	21.56	22.3	21.66	21.2	21.2	21.46	21.66	0.235676	0.261998	0.552823			
2751	Q96K37	Solute carr	SLC35E1	3	11	3.27E+06	2.42E+06	2.80E+06	4.22E+06	3.33E+06	2.95E+06	4.47E+06		6.15E+06	21.61	21.47	21.71	22.02	21.87	21.8	22.06		22.03	21.61	21.87	22.05	0.123492	0.113846	0.019521			
2260	Q15054-3	DNA polym	POLD3	3	10.8	4.18E+06	3.87E+06	3.97E+06	5.61E+06	4.42E+06	5.70E+06		3.60E+06		21.95	22.17	22.25	22.43	22.27	22.71		21.59		22.17	22.43	21.59	0.153086	0.220736				
731	E9PQM0	Phosphatic	PTPMT1	2	14.9		9.10E+06	9.42E+06	8.28E+06	1.40E+07	1.14E+07	1.31E+07	1.41E+07	1.09E+07		23.29	23.44	22.97	23.89	23.62	23.56	23.53	22.96	23.36	23.62	23.53	0.106365	0.471086	0.337694		+	
858	G5EA29	Gem-assoc	GEMIN2	3	18.1	7.79E+06	3.55E+06	4.31E+06	7.10E+06	3.77E+06	5.34E+06				22.81	22.03	22.36	22.79	22.04	22.62				22.36	22.62		0.394772	0.395037				
704	E9PJH7	Mitochond	SLC25A22	5	17.3	6.50E+06	2.95E+06	2.80E+06	5.25E+06	3.42E+06	3.40E+06	2.96E+06	3.09E+06	2.66E+06	22.56	21.74	21.71	22.34	21.91	22	21.46	21.4	20.81	21.74	22	21.4	0.481749	0.224557	0.358798			
2338	Q3SXM5-2	Inactive hy	HSDL1	6	21.5	3.80E+07	3.17E+07	2.83E+07	3.66E+07	3.68E+07	3.58E+07	3.30E+07	4.79E+07	3.17E+07	25.15	25.06	24.95	25.25	25.32	25.36	24.95	25.33	24.44	25.06	25.32	24.95	0.099262	0.058277	0.442904	+		
2681	Q92922	SWI/SNF cc	SMARCC1	19	22.9	2.99E+07	2.87E+07	3.35E+07	3.53E+07	2.87E+07	4.05E+07	1.69E+07	2.53E+07		24.82	24.93	25.18	25.18	24.96	25.53	23.98	24.41		24.93	25.18	24.2	0.182062	0.287964	0.302261			
2663	Q92600-3	Cell differe	RQCD1	6	33.3	9.05E+06	1.04E+07	8.02E+06	1.02E+07	1.05E+07	1.03E+07				23.07	23.49	23.23	23.29	23.48	23.52				23.23	23.48		0.211609	0.12258				
2441	Q6P3X3	Tetratricop	TTC27	6	8.7	5.52E+06	6.45E+06	5.64E+06	7.65E+06	8.27E+06	7.15E+06	9.75E+06	7.12E+06		22.3	22.81	22.77	22.89	23.13	23.03	23.17	22.52		22.77	23.03	22.85	0.280909	0.121895	0.453257	+		
2536	Q8IXB1	DnaJ homc	DNAJC10	18	20.9	1.03E+08	9.72E+07	8.85E+07	1.38E+08	1.15E+08	1.06E+08	1.64E+08	1.52E+08	1.20E+08	26.74	26.76	26.74	27.25	26.97	26.99	27.26	26.94	26.22	26.74	26.99	26.94	0.013829	0.154512	0.529959		+	
1210	O60216	Double-str	RAD21	4	7.8	3.58E+06	3.45E+06	2.63E+06		4.37E+06	2.75E+06				21.73	21.98	21.63		22.25	21.71				21.73	21.98		0.184298	0.383061				
2606	Q8TBM8	DnaJ homc	DNAJB14	2	5	1.74E+06	1.37E+06	1.49E+06	2.22E+06		1.36E+06	2.83E+06			20.62	20.65	20.83	21.09		20.7	21.37			20.65	20.89	21.37	0.115269	0.276428				+
2613	Q8TCT9-5	Minor hist	HM13	4	14.9	5.87E+06	7.42E+06	5.76E+06	8.62E+06	9.86E+06	6.05E+06	6.42E+06	8.60E+06	9.70E+06	22.4	23.01	22.78	23.03	23.39	22.8	22.58	22.82	22.73	22.78	23.03	22.73	0.307239	0.300851	0.12096	+		
1931	Q5SQP8	C-terminal-	CTBP2	4	13.5	8.10E+06	1.13E+07	7.97E+06	8.56E+06	1.11E+07	9.75E+06	6.77E+06	1.02E+07		22.9	23.62	23.21	23.02	23.54	23.46	22.65	23.05		23.21	23.46	22.85	0.360395	0.278404	0.286571			
2973	Q9H9J2	39S riboso	MRPL44	5	14.2	8.43E+06	6.86E+06	5.58E+06	1.01E+07	7.48E+06	7.62E+06	6.52E+06	7.87E+06		22.96	22.89	22.75	23.27	23.03	23.13	22.61	22.69		22.89	23.13	22.65	0.109955	0.12336	0.055016			

692	E9PFT7	Adenomat	APC	8	8.3	3.25E+06	3.14E+06	2.84E+06	4.16E+06	3.98E+06	2.95E+06		3.82E+06	4.88E+06	21.6	21.84	21.75	21.99	22.12	21.8		21.68	21.64	21.75	21.99	21.66	0.117838	0.161848	0.031093	+		
2550	Q8N1B4-2	Vacuolar p	VPS52	7	11.5		3.91E+06	4.63E+06	6.09E+06	6.50E+06	4.91E+06	4.74E+06	6.09E+06			22.19	22.46	22.57	22.83	22.49	22.12	22.31		22.32	22.57	22.21	0.18797	0.175832	0.132822	+		
2071	Q01804	OTU doma	OTUD4	10	13.4	1.70E+07	8.25E+06	7.83E+06	1.22E+07	1.01E+07	7.51E+06	1.35E+07	1.14E+07		23.98	23.15	23.19	23.55	23.43	23.1	23.61	23.21		23.19	23.43	23.41	0.469794	0.230842	0.287096		+	
618	E5RGS4	Prefoldin s	PFDN1	3	23.9	4.05E+06	4.27E+06	3.21E+06	4.47E+06		4.02E+06				21.91	22.29	21.92	22.09		22.22				21.92	22.16		0.217091	0.091339				
2915	Q9H0H5	Rac GTPas	RACGAP1	3	5.2	2.97E+06	1.44E+06	1.69E+06	2.08E+06	2.19E+06	2.21E+06	2.14E+06			21.49	20.72	21.04	20.99	21.28	21.41	20.91			21.04	21.28	20.91	0.387312	0.212693		+		
3005	Q9NQS1	Cell death	AVEN	6	26.5	1.82E+07	1.34E+07	9.34E+06	9.56E+06	1.81E+07	1.52E+07	1.53E+07	1.18E+07	9.73E+06	24.1	23.85	23.43	23.2	24.28	24.09	23.84	23.28	22.74	23.85	24.09	23.28	0.341424	0.574014	0.550073			
2282	Q15404-2	Ras suppre	RSU1	5	26.8	5.19E+06	6.25E+06	6.57E+06	8.45E+06	1.18E+07	5.12E+06	6.13E+06	8.14E+06		22.22	22.76	22.92	23	23.63	22.55	22.52	22.74		22.76	23	22.63	0.366062	0.542201	0.151518	+		
2737	Q96GM8-2	Target of E	TOE1	3	9.5		3.47E+06	4.00E+06		4.74E+06	4.41E+06		4.00E+06			21.99	22.26		22.38	22.34		21.74		22.13	22.36	21.74	0.185355	0.02913				
2855	Q9BUK6-7	Protein mi	MSTO1	2	6.5	2.74E+06	1.80E+06	1.62E+06	2.53E+06	2.02E+06	2.30E+06				21.35	21.08	20.93	21.32	21.16	21.46				21.08	21.32		0.212989	0.147045				
2736	Q96GM5	SWI/SNF-r	SMARCD1	8	16.9	9.56E+06	3.91E+06	5.01E+06	7.46E+06	6.34E+06	6.06E+06				23.16	22.19	22.56	22.87	22.8	22.8				22.56	22.8		0.488437	0.039514				
2552	Q8N1G2	Cap-specifi	CMTR1	4	5.4			1.26E+06			1.62E+06						20.66			20.9				20.66	20.9							
418	B8ZZN6	Small ubiq	SUMO1	2	13	4.92E+06	7.65E+06	7.15E+06	4.97E+06	1.16E+07	8.56E+06	6.29E+06			22.16	23.05	23.04	22.25	23.61	23.27	22.54			23.04	23.27	22.54	0.51315	0.707219				
1983	P62310	U6 snRNA-	LSM3	2	32.4	3.11E+06	3.02E+06	3.20E+06	3.65E+06	3.68E+06	3.41E+06	4.01E+06			21.54	21.77	21.91	21.79	22	22	21.89			21.77	22	21.89	0.186744	0.121492		+		
1396	P05089	Arginase-1	ARG1	7	24.8		2.27E+06	5.39E+06	7.27E+06	4.42E+06	1.50E+06	2.22E+06		2.96E+07		21.38	22.69	22.83	22.27	20.79	21		24.33	22.04	22.27	22.67	0.929191	1.0512	2.358669			+
594	D6RAW3	Cyclin-G-as	GAK	2	18.1	2.56E+06	2.42E+06	1.85E+06	2.87E+06	2.96E+06	2.01E+06	2.18E+06			21.26	21.47	21.17	21.49	21.72	21.27	20.93			21.26	21.49	20.93	0.151389	0.227962				
2278	Q15382	GTP-bindir	RHEB	5	30.4	8.29E+06	8.55E+06	7.27E+06	8.08E+06	9.32E+06	9.12E+06	5.72E+06	6.69E+06		22.94	23.2	23.08	22.95	23.31	23.37	22.42	22.42		23.08	23.31	22.42	0.129048	0.224219	0.004684			
965	H7C3G9	N-acetyl-D	NAGK	3	10.6	2.87E+06	1.97E+06	1.68E+06	2.83E+06	1.99E+06	2.29E+06				21.42	21.22	21.01	21.46	21.15	21.45				21.22	21.45		0.203924	0.178915				
419	H7C463	MICOS con	IMMT	4	10	4.15E+06		2.71E+06	4.65E+06	3.50E+06	3.46E+06	3.27E+06	2.61E+06	2.44E+06	21.94		21.66	22.16	21.93	22.03	21.6	21.14	20.61	21.8	22.03	21.14	0.202221	0.113552	0.491498			
649	E7EQZ4	Survival m	SMN1	6	21.4	2.13E+07	1.44E+07	1.15E+07	1.81E+07	2.47E+07	1.46E+07	1.49E+07	2.02E+07	1.53E+07	24.3	23.94	23.74	24.17	24.69	24.02	23.79	24.1	23.48	23.94	24.17	23.79	0.283895	0.350175	0.310154	+		
2887	Q9BZ29-4	Dedicator	DOCK9	46	28.1	1.24E+08	7.92E+07	7.27E+07	1.01E+08	8.85E+07	1.02E+08	9.02E+07	8.23E+07	9.12E+07	26.93	26.48	26.48	26.71	26.62	26.9	26.42	26.11	25.97	26.48	26.71	26.11	0.257567	0.141077	0.228508			
2270	Q15208	Serine/thr	STK38	22	62.6	1.69E+09	1.57E+09	1.77E+09	3.82E+09	4.16E+09	3.13E+09	3.24E+09	3.56E+09	2.10E+10	31.38	31.56	31.77	31.79	31.75	32.01	31.39	31.26	32.86	31.56	31.79	31.39	0.198115	0.138323	0.890373			
432	C9J0J7	Profilin	PFN2	7	67	5.57E+07	6.10E+07	5.31E+07	6.72E+07	8.63E+07	6.34E+07	5.85E+07	5.20E+07	3.97E+07	25.7	26.1	25.94	26.1	26.57	26.17	25.78	25.44	24.78	25.94	26.17	25.44	0.202958	0.252982	0.509874			
1808	P48147	Prolyl endo	PREP	6	12.8		1.06E+07	1.05E+07		1.28E+07						23.52	23.58		23.77					23.55	23.77		0.046014					
998	J3KNN3	Phosphory	PHKG2	3	8		1.86E+06	1.89E+06	2.24E+06	2.35E+06	2.22E+06	2.09E+06				21.13	21.2	21.11	21.38	21.42	20.83			21.16	21.38	20.83	0.047084	0.171773				
2551	Q8N1F7	Nuclear po	NUP93	24	34.6	7.73E+07	6.71E+07	7.09E+07	8.16E+07	9.39E+07	7.72E+07	8.26E+07	6.07E+07	7.20E+07	26.24	26.26	26.45	26.4	26.73	26.48	26.27	25.66	25.53	26.26	26.48	25.66	0.120035	0.176048	0.394772			
3198	Q9Y2S7	Polymeras	POLDIP2	11	33.7	2.66E+07	2.32E+07	2.01E+07	3.01E+07	2.65E+07	2.20E+07	3.86E+07	3.96E+07	3.41E+07	24.61	24.61	24.47	24.86	24.83	24.6	25.2	25.06	24.59	24.61	24.83	25.06	0.082367	0.141932	0.315315			+
435	C9J2Y9	DNA-direct	POLR2B	38	35.5	8.12E+07	7.87E+07	6.33E+07	8.59E+07	8.69E+07	6.64E+07	1.32E+08	1.31E+08	6.14E+07	26.28	26.47	26.18	26.5	26.59	26.22	26.99	26.73	25.33	26.28	26.5	26.73	0.146283	0.19587	0.895225			+
2089	Q04837	Single-stra	SSBP1	8	58.1	2.62E+07	3.71E+07	4.42E+07	4.50E+07	5.02E+07	3.94E+07	6.48E+07	6.33E+07		24.59	25.33	25.63	25.55	25.77	25.49	25.87	25.73		25.33	25.55	25.8	0.536651	0.145442	0.10443			+
1680	P31689	DnaJ homo	DNAJA1	15	45.1	4.98E+07	7.87E+07	7.90E+07	8.87E+07	9.43E+07	9.01E+07	1.24E+08	1.12E+08	7.27E+07	25.55	26.47	26.58	26.56	26.75	26.69	26.91	26.48	25.55	26.47	26.69	26.48	0.567251	0.094704	0.696708	+		
3154	Q9UL63	Muskelin	MKLN1	5	9.9	5.52E+06	5.20E+06	4.98E+06	7.78E+06	6.07E+06	4.99E+06	7.09E+06	7.50E+06	5.56E+06	22.3	22.51	22.56	22.91	22.73	22.51	22.71	22.62	21.86	22.51	22.73	22.62	0.135692	0.20299	0.466221	+		
23	A0A087W1	Far upstrea	KHSRP	14	24.9	6.21E+07	5.04E+07	6.25E+07	6.48E+07	5.86E+07	6.95E+07	5.81E+07	5.22E+07	3.24E+07	25.82	25.79	26.16	26.03	25.99	26.3	25.75	25.45	24.52	25.82	26.03	25.45	0.209925	0.16724	0.643437			
2569	Q8N7H5-3	RNA polym	PAF1	4	10.3	3.41E+06	3.60E+06	4.50E+06	3.08E+06	4.48E+06	4.12E+06	3.05E+06	2.77E+06		21.66	22.05	22.41	21.57	22.3	22.26	21.49	21.26		22.05	22.26	21.38	0.374054	0.408114	0.165345			
2861	Q9BVA0	Katanin p8	KATNB1	20	44	4.27E+07	2.61E+07	2.80E+07	3.40E+07	4.22E+07	2.74E+07	1.69E+07	2.61E+07	1.38E+07	25.36	24.82	24.93	25.14	25.48	24.94	23.98	24.46	23.32	24.93	25.14	23.98	0.285405	0.274132	0.572135			
1103	O00267-2	Transcripti	SUPT5H	16	18.7	1.15E+07	1.38E+07	1.56E+07	1.76E+07	1.86E+07	1.30E+07	1.04E+07	1.44E+07	1.03E+07	23.44	23.89	24.12	24.1	24.3	23.85	23.24	23.58	22.85	23.89	24.1	23.24	0.345582	0.226907	0.369158			
749	H3BRR1	DNA-bindi	IGHMBP2	2	20.9	1.30E+06	9.22E+05	9.65E+05	1.19E+06	1.29E+06	1.01E+06		1.27E+06		20.21	20.1	20.23	20.27	20.57	20.42		20.11		20.21	20.42	20.11	0.068486	0.146177		+		
2595	Q8NEZ5	F-box only	FBXO22	5	15.9	8.36E+06	7.82E+06	7.83E+06	1.02E+07	9.54E+06	6.86E+06	1.07E+07	1.20E+07	1.17E+07	22.95	23.08	23.19	23.29	23.34	22.96	23.27	23.32	23.07	23.08	23.29	23.27	0.119029	0.205609	0.136431		+	
1791	P46109	Crk-like pr	CRKL	6	32.7	1.09E+07	8.23E+06	8.16E+06	1.16E+07	1.16E+07	9.18E+06	9.42E+06	7.09E+06	1.06E+07	23.36	23.15	23.25	23.46	23.61	23.38	23.11	22.52	22.9	23.25	23.46	22.9	0.104763	0.115708	0.298752			
2467	Q6ZQX7-4	Protein LIA	LIAT1	2	21.7		2.76E+06	2.46E+06		2.93E+06	3.09E+06		2.57E+06			21.62	21.53		21.72	21.86		21.11		21.58	21.79	21.11	0.061464	0.100677				
182	A0A0A0MS	Protein arg	PRMT3	3	7.9	2.42E+06	2.85E+06	3.05E+06	2.88E+06	3.49E+06	3.13E+06	3.14E+06		3.47E+06	21.16	21.67	21.85	21.49	21.93	21.88	21.54		21.12	21.67	21.88	21.33	0.356918	0.237969	0.298891			
2890	Q9BZF1-3	Oxysterol-l	OSBPL8	4	7.4	1.83E+06		2.43E+06	2.33E+06	2.28E+06	2.15E+06		2.98E+06	2.05E+06	20.74		21.52	21.18	21.34	21.37		21.36	20.23	21.13	21.34	20.79	0.546506	0.099315	0.795934			
282	A0A0U1RR	Transcripti	GATAD2B	4	11.8	5.51E+06	3.55E+06	3.94E+06	5.65E+06	4.77E+06	4.80E+06	4.38E+06			22.3	22.03	22.24	22.45	22.39	22.45	22.03			22.24	22.45	22.03	0.142928	0.032483				
91	A0A087WY	Ig gamma-	IGHG1	4	9.9	1.29E+07	8.97E+06	1.57E+07	1.04E+07	1.30E+07	1.66E+07	3.30E+07		1.89E+07	23.59	23.27	24.12	23.33	23.79	24.22	24.95		23.76	23.59	23.79	24.36	0.43224	0.445314	0.845798			+
2500	Q7Z5K2	Wings apa	WAPAL	3	5.2	1.25E+06	9.95E+05	1.41E+06	1.47E+06	1.43E+06	9.80E+05	1.58E+06			20.16	20.25	20.76	20.46	20.71	20.38	20.56			20.25	20.46	20.56	0.321331	0.170357				
2568	Q8N766-4	ER membr	EMC1	5	6.2	3.33E+06	2.61E+06	3.56E+06	3.28E+06	3.27E+06	3.40E+06		1.89E+06		21.64	21.57	22.08	21.66	21.84	22		20.7		21.64	21.84	20.7	0.275952	0.16699				
1987	P62330	ADP-ribos	ARF6	3	24.6	8.94E+06	1.17E+07	1.29E+07	1.51E+07	1.65E+07	1.19E+07	1.39E+07	1.80E+07		23.06	23.68	23.88	23.88	24.16	23.68	23.69	23.9		23.68	23.88	23.79	0.426525	0.238807	0.14871	+		
2730	Q96EV8-2	Dysbindin	DTNBP1	2	7.6	2.27E+06	2.39E+06	1.78E+06	2.58E+06	2.27E+06	1.96E+06				21.06	21.45	21.13	21.35	21.33	21.22				21.13	21.33		0.207527	0.07251				
159	A0A0A0MF	RNA-bindi	RBM10	64	61	9.01E+09	5.45E+09	5.04E+09	9.36E+09	1.06E+10	7.39E+09	9.25E+09	1.00E+10	1.33E+10	33.65	33.38	33.43	33.63	33.72	33.57	33.06	32.86	32.48	33.43	33.63	32.86	0.145091	0.074049	0.296598			
758	F5H008	Vacuolar p	VPS33B	7	11.9	5.78E+06	4.06E+06	5.23E+06	5.09E+06	6.31E+06	5.22E+06	6.09E+06	6.68E+06	5.48E+06	22.38	22.24	22.63	22.3	22.8	22.59	22.51	22.42	21.83	22.38	22.59	22.42	0.196606	0.250661	0.372501	+		
1958	P61353	60S riboso	RPL27	9	49.3	2.32E+08	1.64E+08	1.69E+08	3.09E+08	1.70E+08	1.89E+08	2.16E+08	2.00E+08	2.32E+08	27.91	27.69	27.76	28.25	27.5	27.96	27.73	27.32	27.03	27.76	27.96	27.32	0.111954	0.378528	0.349313			
1289	O75935-3	Dynactin su	DCTN3	5	28.5	9.54E+06	9.96E+06	9.09E+06	1.31E+07	8.36E+06	1.09E+07	7.01E+06	1.29E+07	1.40E+07	23.15	23.43	23.38	23.67	23.15	23.58	22.69	23.41	23.34	23.38	23.58	23.34	0.145836	0.274893	0.394425	+		
2001	P62873	Guanine nu	GNB1	6	26.2	1.20E+07	1.19E+07	1.37E+07	1.13E+07	1.42E+07	1.37E+07	7.19E+06	1.62E+07		23.49	23.7	23.93	23.42	23.9	23.93	22.72	23.75		23.7	23.9	23.24	0.223001	0.287849	0.72757			
1029	J3QRD1	Fatty aldeh	ALDH3A2	5	16	3.20E+06	3.41E+06	2.74E+06			3.13E+06		3.18E+06		21.58	21.97	21.68			21.88		21.44		21.68	21.88	21.44	0.200569					
2440	Q6P3W7	SCY1-like p	SCYL2	54	57.3	1.25E+10	8.94E+09	6.57E+09	1.85E+10	2.12E+10	1.32E+10	1.58E+10	1.68E+10	2.45E+10	34.53	33.86	33.92	33.99	34.12	34.24	34.39	33.74	33.05	33.92	34.12	33.74	0.367901	0.126445	0.673242			
2268	Q15149-4	Plectin	PLEC	104	28.7	1.01E+08	8.78E+07	9.71E+07	1.28E+08	8.19E+07	1.02E+08	9.19E+07	1.15E+08	3.30E+07	26.7	26.63	26.89	27.09	26.48	26.9	26.47	26.53	24.55	26.7	26.9	26.47	0.139377	0.312085	1.125876			
3226	Q9Y3Z3-4	Deoxynucl	SAMHD1	11	21.8	2.09E+07	1.47E+07	1.37E+07	1.59E+07	2.18E+07	1.61E+07	1.30E+07	1.17E+07	1.46E+07	24.27	23.97	23.93	23.97	24.52	24.17	23.55	23.26	23.41	23.97	24.17	23.41	0.185188	0.277363	0.145526			
1147	O14965	Aurora kin	AURKA	5	15.4	9.54E+06	7.12E+06	5.12E+06	8.92E+06	1.32E+07	7.77E+06	8.85E+06	1.22E+07	9.89E+06	23.15	22.96	22.59	23.09	23.82	23.16	23.03	23.35	22.77	22.96	23.16	23.03	0.285902	0.403321	0.2894	+		
2626	Q8WTT2	Nucleolar	NOC3L	3	5	1.08E+06	3.10E+06	8.96E+05			9.42E+05				20.02	21.81	20.12			20.32				20.12	20.32		1.005729					
2277	Q15370	Transcripti	TCEB2	7	59.3	9.14E+06	1.34E+07	1.77E+07	1.67E+07	1.46E+07	2.00E+07	1.73E+07	1.05E+07	2.21E+07	23.09	23.85	24.31	24.05	23.94	24.49	24.03	23.1	23.98	23.85	24.05	23.98	0.619841	0.290969	0.526328		+	
1422	Q5JP53	Tubulin be	TUBB	7	66.4	1.21E+09	1.38E+09	1.34E+09	1.76E+09	2.49E+09	1.60E+09	2.21E+09	2.15E+09	1.87E+09	30.55	31.42	31.14	30.95	31.41	31.34	30.98	30.92	29.84	31.14	31.34	30.92	0.448581	0.244826	0.64304			
2449	Q6PKG0	La-related	LARP1	30	35.6	1.06E+08	6.18E+07	6.38E+07	8.27E+07	7.56E+07	7.36E+07	6.35E+07	4.58E+07	2.57E+07	26.75	26.12	26.21	26.44	26.37	26.4	25.86	25.24	24.15	26.21	26.4	25.24	0.345433	0.03593	0.865946			
2227	Q14573	Inositol 1,4	ITPR3	56	24.1	7.92E+07	7.45E+07	7.93E+07	7.41E+07	8.74E+07	1.03E+08	2.90E+07	6.27E+07	7.16E+06	26.27	26.4	26.59	26.25	26.6	26.92	24.78	25.7	22.27	26.4	26.6	24.78	0.162792	0.336865	1.777198			
1351	P00403	Cytochrom	MT-CO2	5	27.8		1.90E+07	2.94E+07	2.72E+07	2.98E+07		3.61E+07	3.02E+07	4.14E+07		24.34	25.02	24.75	25		25.12	24.7	24.85	24.68	24.87	24.85	0.484594	0.175218	0.212637		+	
2597	Q8NFH3	Nucleopor	NUP43	4	14.7	8.80E+06	8.31E+06	7.50E+06	1.14E+07	9.42E+06	7.34E+06	7.14E+06	6.63E+06	4.81E+06	23.03	23.17	23.13	23.43	23.33	23.07	22.71	22.4	21.61	23.13	23.33	22.4	0.070492	0.186317	0.568409			
1974	P62241	40S riboso	RPS8	10	54.8	5.15E+07	4.11E+07	4.78E+07	5.32E+07	3.55E+07	5.31E+07	2.30E+07	3.13E+07	1.78E+07	25.59	25.51	25.8	25.78	25.25	25.9	24.46	24.75	23.69	25.59	25.78	24.46	0.147125	0.347281	0.549604			
2164	Q13257	Mitotic spi	MAD2L1	3	12.7	1.79E+07	1.40E+07	1.08E+07	3.46E+07	7.79E+06	1.54E+07	1.44E+07	1.56E+07		24.06	23.91	23.63	25.17	23.07	24.11	23.74	23.7		23.91	24.11	23.72	0.219282	1.054351	0.031617			
3100	Q9UBI1	COMM dor	COMMD3	4	28.2	2.29E+06	3.67E+06	6.88E+06	3.29E+06	5.57E+06	4.12E+06	3.29E+06	3.11E+06		21.07	22.07	22.98	21.67	22.61	22.26	21.61	21.42		22.07	22.26	21.51	0.955493	0.471763	0.134229			
2345	Q49AG3	Zinc finger	ZBED5	13	20.2	2.50E+07	2.02E+07	1.76E+07	2.48E+07	2.33E+07	1.74E+07	2.94E+07	2.85E+07	1.61E+07	24.52	24.4	24.3	24.62	24.6	24.29	24.79	24.58	23.57	24.4	24.6	24.58	0.108053	0.183955	0.65296		+	
41	A0A087WU	Proteasom	PSMB3	4	37.9	1.36E+07	1.08E+07	1.02E+07	1.38E+07	2.34E+07	7.58E+06	1.35E+07	1.16E+07	1.39E+07	23.64	23.55	23.55	23.74	24.6	23.13	23.61	23.24	23.33	23.55	23.74	23.33	0.051025	0.740254	0.194539			
3151	Q9UKN8	General tra	GTF3C4	6	9.5	6.54E+06	6.37E+06	4.92E+06	6.95E+06	6.71E+06	4.36E+06	5.53E+06	5.09E+06		22.57	22.79	22.55	22.76	22.88	22.33	22.37	22.04		22.57	22.76	22.21	0.133572	0.287786	0.229778			
43	A0A087WU	SRA stem-l	SLIRP	4	51.1	3.63E+06	5.02E+06	8.93E+06	6.56E+06	5.36E+06	6.74E+06	8.39E+06	7.68E+06		21.75	22.49	23.35	22.68	22.56	22.94	22.93	22.65		22.49	22.68	22.79	0.797621	0.191282	0.199088			
2955	Q9H6W3	Bifunctiona	NO66	5	13.6	7.23E+06	3.73E+06	1.91E+06		5.64E+06	3.37E+06	3.32E+06	4.45E+06	3.88E+06	22.72	22.11	21.22		22.62	21.99	21.61	21.88	21.24	22.11	22.3	21.61	0.752698	0.445064	0.322411			
2833	Q9BRS2	Serine/thre	RIOK1	34	48.2	2.85E+09	1.67E+09	1.67E+09	3.77E+09	4.34E+09	3.10E+09	4.34E+09	4.51E+09	3.79E+09	32.04	31.65	31.52	31.69	31.84	31.96	31.68	31.56	30.39	31.65	31.84	31.56	0.2724	0.134524	0.710352	+		
2731	Q96EY1	DnaJ homo	DNAJA3	5	12.3		4.55E+06	3.44E+06		4.82E+06	4.38E+06	4.90E+06	5.56E+06	5.25E+06		22.37	22		22.41	22.33	22.17	22.18	21.77	22.18	22.37	22.17	0.260537	0.051851	0.237398	+		
2402	Q5VT06	Centrosom	CEP350	24	11.5	1.65E+07	1.65E+07	1.41E+07	1.80E+07	2.02E+07	1.55E+07	1.51E+07	1.79E+07	4.02E+06	23.93	24.12	23.97	24.16	24.44	24.11	23.82	23.88	21.28	23.97	24.16	23.82	0.101988	0.17946	1.48717			
2130	Q12765-3	Secernin-1	SCRN1	2	8.4		2.45E+06	2.40E+06			2.70E+06					21.49	21.49			21.68				21.49	21.68		0.002697					
2884	Q9BYD1	39S riboso	MRPL13	5	34.8	7.58E+06	5.53E+06	6.05E+06	6.80E+06	8.40E+06	6.88E+06	4.96E+06	3.40E+06		22.78	22.6	22.83	22.72	23.17	22.97	22.19	21.52		22.78	22.97	21.85	0.120444	0.223167	0.477625			
3213	Q9Y3B7	39S riboso	MRPL11	6	40.1	6.77E+06	8.32E+06	7.23E+06	8.35E+06	9.18E+06	8.40E+06	8.78E+06	8.24E+06		22.61	23.17	23.07	22.99	23.27	23.25	23.01	22.75		23.07	23.25	22.88	0.295953	0.158826	0.180191			

2781	Q96S55-2	ATPase WI	WRNIP1	11	19.1	1.60E+07	1.00E+07	1.10E+07	1.49E+07	1.36E+07	1.14E+07	1.80E+07	1.44E+07	9.16E+06	23.89	23.44	23.66	23.87	23.85	23.62	24.08	23.58	22.65	23.66	23.85	23.58	0.227102	0.136065	0.725418	+		
1047	K7EK42	Tubulin-fol	TBCB	4	24.1		1.00E+07	8.23E+06		1.63E+07	6.82E+06	1.77E+07				23.44	23.26		24.12	22.95	24.06			23.35	23.53	24.06	0.124742	0.825665				+
770	F5H5D3	Tubulin alp	TUBA1C	2	53.2	3.41E+07	7.42E+06	1.07E+07	1.28E+07	1.30E+07	1.79E+07	1.92E+07		1.59E+07	25.01	23.01	23.61	23.63	23.79	24.36	24.19		23.55	23.61	23.79	23.87	1.025441	0.381465	0.449629			
1892	P52756	RNA-bindir	RBM5	21	27.9	4.55E+07	6.22E+07	5.02E+07	6.63E+07	6.27E+07	5.19E+07	8.33E+07	2.56E+07	1.08E+07	25.44	26.13	25.88	26.07	26.1	25.87	26.3	24.43	22.94	25.88	26.07	24.43	0.352833	0.127531	1.682537			
2867	Q9BVS4-2	Serine/thre	RIOK2	2	5.1		2.46E+06	2.43E+06			2.72E+06					21.5	21.52			21.69				21.51	21.69		0.014156					
1812	P48634-2	Protein PR	PRRC2A	8	6.6	4.23E+06	3.39E+06	3.70E+06	4.16E+06	4.16E+06	3.90E+06				21.97	21.96	22.15	21.99	22.16	22.18				21.97	22.16		0.108496	0.101416				
698	E9PHA2	Condensin	NCAPH	7	13.3	9.05E+06	1.09E+07	1.01E+07	1.36E+07	1.26E+07	5.15E+06	1.24E+07	8.66E+06		23.07	23.57	23.54	23.72	23.74	22.57	23.48	22.83		23.54	23.72	23.16	0.277549	0.672328	0.458941			
2419	Q68CP9-3	AT-rich inte	ARID2	9	6.3	7.05E+06	8.05E+06	8.04E+06	9.80E+06	9.23E+06	1.18E+07	3.69E+06	2.62E+06		22.68	23.11	23.23	23.23	23.3	23.67	21.77	21.15		23.11	23.3	21.46	0.291252	0.236929	0.436935			
1145	O14950	Myosin reg	MYL12B	12	80.2	2.79E+08	2.55E+08	2.32E+08	3.93E+08	3.83E+08	1.52E+08	2.02E+08	3.50E+08	4.35E+08	28.09	28.36	28.38	28.54	28.6	27.64	27.62	27.95	28.04	28.36	28.54	27.95	0.159772	0.539236	0.222008			
86	A0A087WY	Vacuolar p	VTA1	3	15.7	3.55E+06	5.32E+06	3.85E+06			4.59E+06				21.71	22.55	22.21			22.39				22.21	22.39		0.419088					
2524	Q8IUR0	Trafficking	TRAPPC5	3	14.4	2.60E+06	3.07E+06	2.75E+06		3.32E+06		2.55E+06			21.27	21.8	21.69		21.87		21.2			21.69	21.87	21.2	0.278631					
465	C9JJ19	28S riboso	MRPS34	3	23.6	2.64E+06	2.58E+06	4.35E+06	2.67E+06	3.37E+06	2.78E+06		3.28E+06		21.29	21.55	22.37	21.4	21.89	21.73		21.47		21.55	21.73	21.47	0.566359	0.24933		+		
257	A0A0G2JIV	Heat shock	HSPA1B	28	54.8	7.55E+08	6.15E+08	6.26E+08	8.04E+08	8.84E+08	7.18E+08	7.50E+08	6.65E+08	7.22E+08	29.58	29.51	29.71	29.65	29.91	29.76	29.39	28.87	28.79	29.58	29.76	28.87	0.099659	0.130225	0.323413			
156	A0A0A0MI	Voltage-de	VDAC2	8	41.1	1.54E+07	1.51E+07	1.32E+07	1.69E+07	1.60E+07	1.79E+07	1.16E+07	1.30E+07		23.82	24.02	23.91	24.06	24.09	24.36	23.38	23.42		23.91	24.09	23.4	0.098912	0.165137	0.029373			
678	E9PDF2	2-oxogluta	OGDH	17	19	1.43E+07	1.22E+07	1.99E+07	1.48E+07	1.43E+07	1.58E+07	6.39E+06	1.16E+07	4.67E+06	23.72	23.74	24.46	23.85	23.92	24.13	22.57	23.24	21.55	23.74	23.92	22.57	0.42156	0.145065	0.849519			
3060	Q9NYY8-2	FAST kinas	FASTKD2	12	24.4	1.76E+07	2.07E+07	1.73E+07	2.19E+07	2.66E+07	1.93E+07	2.04E+07	2.08E+07	1.04E+07	24.04	24.44	24.27	24.45	24.84	24.44	24.31	24.14	22.87	24.27	24.45	24.14	0.204528	0.225782	0.787276			
1155	O15084-2	Serine/thre	ANKRD28	7	11	3.96E+06	4.60E+06	5.19E+06	4.65E+06	5.35E+06	5.68E+06		7.34E+06	4.37E+06	21.88	22.38	22.61	22.16	22.56	22.7		22.57	21.4	22.38	22.56	21.99	0.376446	0.28254	0.825245			
1782	P43355	Melanoma	MAGEA1	3	8.7	3.27E+06	3.48E+06	2.69E+06	4.25E+06	3.21E+06	2.85E+06	4.27E+06	3.73E+06	3.54E+06	21.61	22	21.65	22.03	21.83	21.76	22	21.64	21.13	21.65	21.83	21.64	0.215331	0.137765	0.434843	+		
1395	V9GYR2	Sodium/pc	ATP1B1	2	19.2	4.71E+06	4.05E+06	4.14E+06	4.15E+06	4.94E+06	4.65E+06	4.78E+06	5.50E+06	4.33E+06	22.09	22.24	22.3	21.98	22.44	22.41	22.13	22.16	21.39	22.24	22.41	22.13	0.108374	0.253029	0.434785	+		
1248	O75143-2	Autophagy	ATG13	10	27.1	1.74E+07	1.09E+07	9.95E+06	1.53E+07	1.26E+07	1.07E+07	9.17E+06	1.20E+07	1.11E+07	24.03	23.57	23.53	23.89	23.74	23.56	23.07	23.32	22.98	23.57	23.74	23.07	0.278999	0.167099	0.177444			
2054	Q00534	Cyclin-dep	CDK6	5	23	9.17E+06	9.12E+06	9.44E+06	1.16E+07	8.34E+06	1.15E+07	7.98E+06	8.16E+06		23.1	23.3	23.44	23.46	23.15	23.64	22.86	22.74		23.3	23.46	22.8	0.172867	0.245958	0.086941			
2745	Q96I24	Far upstrea	FUBP3	6	17.5	1.15E+07	6.88E+06	7.47E+06	1.02E+07	1.05E+07	8.35E+06	6.92E+06	6.66E+06		23.44	22.9	23.12	23.29	23.48	23.25	22.67	22.41		23.12	23.29	22.54	0.27227	0.124699	0.184326			
921	H3BQF2	COMM dor	COMMD4	2	20.5	2.75E+06	2.94E+06	3.52E+06	3.64E+06		3.43E+06	3.24E+06			21.35	21.73	22.05	21.78		22.01	21.58			21.73	21.9	21.58	0.346805	0.160345				
920	H3BLZ8	Probable A	DDX17	17	34.9	5.05E+07	3.99E+07	4.42E+07	4.77E+07	4.95E+07	4.67E+07	3.72E+07	3.59E+07	1.97E+07	25.56	25.45	25.63	25.63	25.73	25.72	25.15	24.9	23.82	25.56	25.72	24.9	0.091426	0.054823	0.704945			
2335	Q32MZ4-3	Leucine-ric	LRRFIP1	2	5.1		9.80E+05	1.06E+06			1.04E+06					20.21	20.37			20.45				20.29	20.45		0.113213					
1240	O60832-2	H/ACA ribc	DKC1	3	8.3	1.62E+06	1.64E+06	2.18E+06	1.89E+06	2.08E+06	1.81E+06				20.53	20.93	21.38	20.87	21.21	21.09				20.93	21.09		0.425789	0.172431				
38	A0A087WU	Mannose-1	GMPPA	3	8.8	5.78E+06	5.71E+06	3.22E+06	5.72E+06	5.30E+06	5.31E+06	6.03E+06	6.54E+06	7.91E+06	22.38	22.63	21.92	22.46	22.55	22.61	22.5	22.39	22.45	22.38	22.55	22.45	0.361169	0.073518	0.054421	+		
1266	O75449	Katanin p6	KATNA1	19	38.3	2.19E+08	1.50E+08	1.49E+08	1.96E+08	2.23E+08	1.63E+08	1.04E+08	1.57E+08	7.37E+07	27.81	27.52	27.55	27.63	27.89	27.71	26.64	27	25.59	27.55	27.71	26.64	0.161335	0.132358	0.734426			
285	A0A140T9	HLA class I	HLA-C	3	31.3	3.92E+06	4.00E+06	3.89E+06	5.07E+06	4.86E+06	4.53E+06	3.75E+06	5.35E+06	4.89E+06	21.87	22.22	22.22	22.29	22.42	22.38	21.8	22.1	21.65	22.22	22.38	21.8	0.20411	0.067422	0.232349			
316	A6NIW2	Dedicator	DOCK11	31	19.9	3.43E+07	2.85E+07	2.99E+07	3.50E+07	3.11E+07	4.10E+07	3.18E+07	4.22E+07	2.63E+07	25.02	24.92	25.04	25.18	25.04	25.55	24.92	25.13	24.17	25.02	25.18	24.92	0.063325	0.262825	0.502439	+		
1168	O15294	UDP-N-ace	OGT	48	47.9	8.14E+08	5.61E+08	4.97E+08	7.79E+08	9.05E+08	6.71E+08	8.45E+08	8.80E+08	1.07E+09	29.69	29.44	29.43	29.53	29.93	29.6	29.54	29.47	29.35	29.44	29.6	29.47	0.142558	0.214889	0.095815	+		
3017	Q9NS87-2	Kinesin-like	KIF15	7	6.9	2.40E+06	1.63E+06	2.34E+06	2.40E+06	2.23E+06	2.47E+06	2.11E+06	2.08E+06		21.14	20.91	21.46	21.23	21.3	21.54	20.87	20.85		21.14	21.3	20.86	0.279213	0.161093	0.009406			
2539	Q8IXW5-2	Putative RI	RPAP2	4	7.4	5.58E+06	4.69E+06	3.66E+06	5.63E+06	5.34E+06	4.88E+06	9.09E+06	1.18E+07	3.13E+06	22.32	22.4	22.14	22.44	22.56	22.48	23.05	23.28	21.01	22.32	22.48	23.05	0.136131	0.062252	1.253672			+
3040	Q9NVJ2	ADP-ribosy	ARL8B	4	22	4.09E+06	3.48E+06	3.87E+06	4.94E+06	3.35E+06	3.86E+06	4.86E+06			21.92	22	22.22	22.24	21.88	22.16	22.16			22	22.16	22.16	0.153919	0.187919			+	
32	A0A087WU	EH domain	EHD4	12	25.4	6.79E+06	6.06E+06	5.90E+06	7.42E+06	6.54E+06	6.68E+06	8.36E+06	6.30E+06	1.01E+07	22.62	22.7	22.81	22.86	22.83	22.92	22.93	22.34	22.81	22.7	22.86	22.81	0.093846	0.043281	0.309576		+	
392	B5MC59	Replicatior	RPA3	3	52.4		2.31E+06	4.28E+06	3.47E+06		4.44E+06	3.38E+06	3.26E+06			21.4	22.35	21.72		22.35	21.64	21.47		21.87	22.03	21.55	0.666841	0.447297	0.120845			
181	A0A0A0MS	Torsin-1A-i	TOR1AIP1	10	27.1	1.20E+07	1.70E+07	1.40E+07	1.41E+07	1.63E+07	1.58E+07	1.25E+07	1.25E+07	7.51E+06	23.49	24.21	23.96	23.78	24.12	24.13	23.49	23.37	22.35	23.96	24.12	23.37	0.368743	0.200532	0.627381			
2414	Q5XPI4	E3 ubiquiti	RNF123	6	5.3	2.13E+06	2.74E+06	1.74E+06	2.41E+06	2.22E+06	1.88E+06	1.71E+06	2.02E+06		20.96	21.61	21.09	21.24	21.29	21.17	20.62	20.81		21.09	21.24	20.72	0.343904	0.059835	0.134959			
1959	P61586	Transformi	RHOA	5	24.4	3.90E+06	3.13E+06	5.25E+06		3.28E+06	3.92E+06	3.72E+06			21.86	21.83	22.64		21.85	22.19	21.78			21.86	22.02	21.78	0.4623	0.240364		+		
2115	Q08380	Galectin-3-	LGALS3BP	9	20.3	2.30E+07	2.09E+07	2.22E+07	2.42E+07	2.55E+07	2.21E+07	3.32E+07	2.98E+07	3.69E+07	24.4	24.45	24.65	24.58	24.78	24.61	24.97	24.66	24.69	24.45	24.61	24.69	0.129419	0.10947	0.169856			
320	F8WC89	SAC3 doma	SAC3D1	2	5.3	2.41E+06	2.36E+06	2.76E+06	3.10E+06	2.68E+06	1.90E+06	2.77E+06	3.31E+06		21.15	21.43	21.69	21.59	21.59	21.19	21.33	21.49		21.43	21.59	21.41	0.270958	0.230882	0.111527	+		
1998	P62829	60S riboso	RPL23	5	40	1.38E+08	1.21E+08	8.40E+07	2.02E+08	1.43E+08	1.21E+08	1.31E+08	1.61E+08	1.67E+08	27.09	27.21	26.65	27.68	27.24	27.21	26.99	27.04	26.58	27.09	27.24	26.99	0.291019	0.262071	0.253286	+		
1035	J9JID7	Lamin-B2	LMNB2	7	11.6	2.32E+06	2.62E+06	3.49E+06	3.09E+06	2.97E+06	3.05E+06				21.09	21.57	22.02	21.58	21.73	21.83				21.57	21.73		0.462969	0.129485				
2968	Q9H974-2	Queuine tF	QTRTD1	2	10.6			3.06E+06		3.45E+06	3.66E+06						21.86		21.92	22.11				21.86	22.01			0.135193				
2453	Q6R327	Rapamycin	RICTOR	8	5.1	5.37E+06	5.92E+06	4.06E+06	5.06E+06	5.67E+06	4.69E+06	4.96E+06	4.81E+06		22.27	22.67	22.27	22.28	22.63	22.42	22.19	21.99		22.27	22.42	22.09	0.232423	0.177042	0.145027			
1329	O95573	Long-chain	ACSL3	12	21.9	2.00E+07	1.81E+07	2.10E+07	2.14E+07	2.27E+07	1.82E+07	2.16E+07	2.15E+07	1.92E+07	24.22	24.27	24.56	24.42	24.58	24.39	24.37	24.17	23.78	24.27	24.42	24.17	0.183162	0.099462	0.299283	+		
1078	M0QZR4	Rho guanir	ARHGEF1	6	6.7	4.14E+06	2.81E+06	2.94E+06	3.82E+06	4.13E+06	3.27E+06	2.71E+06	2.71E+06	2.44E+06	21.94	21.64	21.79	21.87	22.15	21.94	21.3	21.22	20.61	21.79	21.94	21.22	0.146584	0.145214	0.374688			
186	Q9HBD4	Transcripti	SMARCA4	29	18.5	3.85E+07	4.33E+07	3.64E+07	4.34E+07	4.28E+07	5.02E+07	2.99E+07	2.95E+07	1.29E+07	25.17	25.57	25.35	25.48	25.5	25.79	24.82	24.63	23.21	25.35	25.5	24.63	0.203594	0.169013	0.883587			
1739	P38435	Vitamin K-c	GGCX	3	6.3	5.38E+06	4.92E+06	5.01E+06	6.32E+06	5.98E+06	3.95E+06	8.94E+06	8.89E+06		22.27	22.47	22.56	22.62	22.71	22.2	23.04	22.86		22.47	22.62	22.95	0.150071	0.27019	0.125943			+
1848	P49903	Selenide, w	SEPHS1	5	16.1		8.45E+06	4.49E+06		6.17E+06	7.62E+06	5.32E+06				23.18	22.41		22.76	23.13	22.32			22.8	22.95	22.32	0.546413	0.262687				
2247	Q14CX7	N-alpha-ac	NAA25	21	26	2.89E+07	3.13E+07	3.29E+07	2.69E+07	3.43E+07	3.40E+07	3.09E+07	4.10E+07	1.58E+07	24.75	25.04	25.14	24.73	25.19	25.29	24.85	25.09	23.54	25.04	25.19	24.85	0.203864	0.295863	0.835346			
1320	O95347	Structural	SMC2	45	41.4	1.15E+08	1.10E+08	9.38E+07	1.25E+08	1.20E+08	9.73E+07	1.15E+08	7.28E+07	1.45E+08	26.88	27	26.85	27.04	27.03	26.8	26.79	25.92	26.44	26.88	27.03	26.44	0.079105	0.137899	0.439266			
1230	O60645-2	Exocyst co	EXOC3	8	15.1	7.74E+06	7.44E+06	5.95E+06	7.77E+06	7.22E+06	7.37E+06	7.09E+06	8.01E+06	6.51E+06	22.81	23.02	22.82	22.91	22.97	23.08	22.71	22.71	22.14	22.82	22.97	22.71	0.11801	0.085153	0.329421			
238	A0A0C4DG	DNA-direct	POLR2A	42	26.5	7.37E+07	6.55E+07	5.39E+07	8.70E+07	7.28E+07	6.03E+07	1.36E+08	1.00E+08	1.07E+08	26.15	26.22	25.97	26.53	26.3	26.06	27.03	26.37	26.11	26.15	26.3	26.37	0.129431	0.233405	0.473364			
2662	Q92598-2	Heat shock	HSPH1	21	31.8	6.25E+07	8.02E+07	1.03E+08	5.58E+07	9.09E+07	8.79E+07	4.77E+07	6.49E+07	6.83E+06	25.83	26.5	26.99	25.83	26.65	26.66	25.47	25.77	22.19	26.5	26.65	25.47	0.58573	0.47837	1.984578			
1310	O95140	Mitofusin-2	MFN2	5	7.9		1.91E+06	1.85E+06	2.81E+06	1.79E+06	2.09E+06		2.64E+06	2.65E+06		21.17	21.17	21.44	21.04	21.32		21.16	20.79	21.17	21.32	20.97	0.005288	0.205886	0.264898			
1641	P28288	ATP-bindin	ABCD3	18	27	5.69E+07	5.65E+07	5.61E+07	7.32E+07	5.46E+07	6.32E+07	6.67E+07	7.32E+07	3.72E+07	25.75	26.01	26.02	26.22	25.86	26.15	25.9	25.92	24.71	26.01	26.15	25.9	0.155083	0.192422	0.694029			
1819	P48960-2	CD97 antig	CD97	4	7.1	4.14E+06	2.96E+06	3.62E+06			3.62E+06	4.15E+06	3.82E+06		21.94	21.75	22.12			22.08	21.95	21.68		21.94	22.08	21.82	0.183745		0.193766			
1192	O43390	Heterogen	HNRNPR	12	28.4	1.71E+07	2.24E+07	2.51E+07	2.07E+07	2.50E+07	3.12E+07	2.54E+07	3.23E+07		23.99	24.58	24.81	24.36	24.72	25.16	24.61	24.77		24.58	24.72	24.69	0.419199	0.40213	0.115934		+	
2763	Q96P48-3	Arf-GAP wi	ARAP1	14	14	1.09E+07	1.05E+07	1.13E+07	1.29E+07	1.17E+07	1.60E+07	7.05E+06	7.29E+06	3.64E+06	23.36	23.5	23.7	23.65	23.62	24.16	22.7	22.56	21.17	23.5	23.65	22.56	0.172477	0.302843	0.846576			
3107	Q9UBS4	DnaJ homo	DNAJB11	9	31.3	3.11E+07	2.67E+07	2.19E+07	3.16E+07	3.19E+07	2.67E+07	2.41E+07	3.09E+07	2.74E+07	24.9	24.84	24.62	24.98	25.08	24.87	24.52	24.74	24.25	24.84	24.98	24.52	0.147236	0.106625	0.246244			
2382	Q5SY16	Polynucleo	NOL9	3	6.8		3.79E+06	3.79E+06	4.06E+06	4.50E+06	4.31E+06					22.14	22.19	21.96	22.31	22.31				22.17	22.31		0.035011	0.203219				
1598	P23458	Tyrosine-p	JAK1	52	45.6	6.65E+08	4.27E+08	3.95E+08	6.53E+08	5.17E+08	5.04E+08	7.22E+08	7.76E+08	1.15E+09	29.42	29.1	29.09	29.24	29.16	29.28	29.3	29.23	29.49	29.1	29.24	29.3	0.188623	0.057441	0.136852			
617	E5RGS2	Receptor e	REEP4	2	13.1		1.87E+06			2.02E+06	2.18E+06					21.14			21.16	21.39				21.14	21.27			0.157026				
1704	P35232	Prohibitin	PHB	12	52.9	2.94E+08	2.39E+08	3.01E+08	3.27E+08	3.25E+08	3.13E+08	2.17E+08	3.47E+08	1.87E+08	28.13	28.24	28.8	28.29	28.38	28.66	27.74	27.92	26.74	28.24	28.38	27.74	0.358079	0.194774	0.633728			
1898	P53004	Biliverdin r	BLVRA	11	39.5	4.18E+07	3.56E+07	3.67E+07	4.21E+07	4.11E+07	4.01E+07	3.69E+07	4.59E+07	4.36E+07	25.31	25.28	25.37	25.44	25.44	25.52	25.14	25.25	24.92	25.31	25.44	25.14	0.044025	0.045131	0.168923			
2578	Q8NBJ5	Procollage	COLGALT1	11	15.9	1.83E+07	1.64E+07	1.74E+07	1.83E+07	1.79E+07	1.78E+07	1.99E+07	1.83E+07	1.77E+07	24.11	24.12	24.28	24.18	24.25	24.34	24.23	23.93	23.68	24.12	24.25	23.93	0.0953	0.080198	0.277057			
2435	Q6P1L8	39S riboso	MRPL14	2	13.1	8.01E+06	7.16E+06	6.25E+06	7.11E+06	8.32E+06	7.03E+06				22.87	22.97	22.87	22.79	23.15	23.01				22.87	23.01		0.054701	0.178308				
1795	P46821	Microtubu	MAP1B	47	30.8	1.37E+08	1.18E+08	1.05E+08	1.41E+08	1.39E+08	9.85E+07	1.80E+08	1.40E+08	4.89E+07	27.07	27.13	27.03	27.29	27.2	26.84	27.43	26.84	25.07	27.07	27.2	26.84	0.04775	0.240617	1.230117			
234	A0A0C4DG	Dolichyl-di	DDOST	14	50.3	7.14E+07	6.75E+07	5.77E+07	6.26E+07	7.47E+07	6.69E+07	7.27E+07	7.86E+07	4.73E+07	26.11	26.27	26.06	25.98	26.34	26.24	26.05	26.04	25.02	26.11	26.24	26.04	0.110803	0.184321	0.592006	+		
2618	Q8TDX7	Serine/thre	NEK7	5	19.9	6.16E+06	6.92E+06	5.63E+06	7.33E+06	7.90E+06	6.55E+06	5.07E+06	6.05E+06	5.41E+06	22.48	22.92	22.77	22.84	23.08	22.9	22.23	22.29	21.81	22.77	22.9	22.23	0.220785	0.123977	0.262257			
1228	O60568	Procollage	PLOD3	15	24.4	2.42E+07	2.78E+07	2.96E+07	2.68E+07	3.07E+07	2.90E+07	4.18E+07	3.93E+07		24.49	24.9	25.03	24.73	25.03	25.03	25.31	25.04		24.9	25.03	25.17	0.282538	0.173843	0.190912			+
1176	O15427	Monocarb	SLC16A3	4	8.6		6.94E+06	9.73E+06	1.20E+07	9.53E+06	8.69E+06	6.34E+06				22.93	23.49	23.52	23.34	23.29	22.55			23.21	23.34	22.55	0.391326	0.122803				
353	B0YIW6	Coatomer	ARCN1	10	22.5	3.27E+07	3.31E+07	3.00E+07	3.27E+07	4.03E+07	3.18E+07	2.97E+07	2.97E+07	3.53E+07	24.97	25.15	25.04	25.07	25.41	25.17	24.8	24.65	24.65	25.04	25.17	24.65	0.090726	0.175317	0.087414			
2525	Q8IUR7-7	Armadillo	ARMC8	9	15.9	1.83E+07	1.20E+07	1.10E+07	1.89E+07	1.13E+07	1.30E+07	1.83E+07	1.60E+07	1.65E+07	24.11	23.72	23.66	24.22	23.57	23.85	24.1	23.73	23.61	23.72	23.85	23.73	0.245684	0.324132	0.256809	+		
946	H7BXK7	Serpin B8	SERPINB8	2	23.3	1.41E+06	1.31E+06	1.18E+06	1.63E+06	1.41E+06	1.40E+06	1.67E+06			20.35	20.62	20.55	20.61	20.68	20.73	20.59			20.55	20.68	20.59	0.139394	0.063169		+		
1733	P36954	DNA-direct	POLR2I	2	27.2			9.47E+06	7.15E+06	1.93E+07		1.04E+07					23.45	22.8	24.36		23.24			23.45	23.58	23.24		1.105655				

1412	P06748-2	Nucleopho	NPM1	8	37.4	1.73E+08	1.14E+08	2.05E+08	1.19E+08	1.87E+08	1.74E+08	4.93E+07	1.54E+08	6.80E+07	27.48	27.04	28.17	26.93	27.61	27.81	25.54	26.96	25.48	27.48	27.61	25.54	0.567744	0.464745	0.837401			
2465	Q6YN16-2	Hydroxyste	HSDL2	6	28.4	1.39E+07	1.30E+07	1.14E+07	9.07E+06	1.69E+07	1.30E+07	2.19E+07	1.92E+07		23.67	23.82	23.72	23.12	24.19	23.85	24.39	24.01		23.72	23.85	24.2	0.07718	0.544614	0.269649			+
2294	Q15645	Pachytene	TRIP13	20	46.3	2.56E+08	2.41E+08	1.97E+08	2.92E+08	2.82E+08	2.29E+08	4.03E+08	4.17E+08	4.26E+08	27.98	28.26	28.06	28.17	28.22	28.18	28.51	28.21	27.93	28.06	28.18	28.21	0.146868	0.030099	0.286388			
2369	Q5JVF3-3	PCI domair	PCID2	11	32.7	1.98E+07	1.93E+07	1.90E+07	2.23E+07	2.19E+07	1.76E+07	2.53E+07	2.06E+07	2.49E+07	24.21	24.36	24.4	24.48	24.52	24.31	24.6	24.13	24.12	24.36	24.48	24.13	0.09748	0.114026	0.27285			
2446	Q6PGP7	Tetratricop	TTC37	12	10.4	7.71E+06	6.65E+06	7.15E+06	8.21E+06	8.31E+06	6.55E+06	1.19E+07	9.74E+06	7.07E+06	22.8	22.84	23.04	22.96	23.14	22.9	23.41	22.99	22.24	22.84	22.96	22.99	0.125731	0.126416	0.589847			
1717	P35659	Protein DE	DEK	7	21.1	8.24E+06	6.62E+06	9.25E+06	8.81E+06	5.47E+06	7.86E+06	6.37E+06	5.85E+06		22.94	22.83	23.41	23.06	22.59	23.17	22.56	22.25		22.94	23.06	22.41	0.306989	0.307597	0.223587			
2901	Q9GZL7	Ribosome	WDR12	3	9.9			3.08E+06		3.70E+06	3.34E+06	3.81E+06					21.87		22.01	21.97	21.81			21.87	21.99	21.81		0.027168		+		
3147	Q9UKD2	mRNA turr	MRTO4	8	40.2	1.15E+07	1.51E+07	1.54E+07	1.57E+07	1.64E+07	1.77E+07	1.57E+07	1.58E+07	8.12E+06	23.44	24.02	24.1	23.94	24.14	24.33	23.85	23.72	22.47	24.02	24.14	23.72	0.360283	0.192459	0.763567			
2000	P62854	40S ribosoi	RPS26	3	27	3.08E+07	6.89E+07	6.28E+07	4.04E+07	7.26E+07	7.18E+07	9.27E+07	1.24E+08	4.14E+07	24.88	26.3	26.17	25.37	26.29	26.36	26.48	26.67	24.85	26.17	26.29	26.48	0.785104	0.552739	0.999762			+
771	H0Y8R1	G-rich sequ	GRSF1	4	11.3	2.48E+06	3.35E+06	2.59E+06			2.78E+06				21.2	21.94	21.61			21.73				21.61	21.73		0.370108					
754	F5GXX5	Dolichyl-di	DAD1	3	37.6	1.64E+07	7.04E+06	4.92E+06	8.82E+06	6.83E+06	1.74E+07	1.20E+07	2.17E+07	3.46E+07	23.92	22.94	22.55	23.06	22.91	24.29	23.42	24.18	24.62	22.94	23.06	24.18	0.708523	0.756852	0.60471			
3217	Q9Y3D0	Mitotic spi	FAM96B	4	43.6	7.43E+06	1.12E+07	1.03E+07	1.32E+07	1.36E+07	1.00E+07	1.61E+07	1.31E+07		22.75	23.61	23.56	23.68	23.85	23.49	23.89	23.43		23.56	23.68	23.66	0.480886	0.18127	0.323166		+	
1258	O75367-2	Core histor	H2AFY	5	18.4	5.63E+06	3.76E+06	3.50E+06	4.97E+06	5.01E+06	3.80E+06	8.88E+06	7.37E+06	4.93E+06	22.34	22.13	22.03	22.25	22.46	22.15	23.03	22.58	21.67	22.13	22.25	22.58	0.158848	0.161577	0.692296			+
3241	Q9Y5B6	PAX3- and	PAXBP1	13	18.4	2.35E+07	2.39E+07	2.03E+07	2.57E+07	1.45E+07	2.21E+07	1.36E+07	3.24E+07	1.41E+07	24.45	24.65	24.49	24.69	23.93	24.61	23.64	24.78	23.36	24.49	24.61	23.64	0.106911	0.415599	0.750783			
1419	P07339	Cathepsin	CTSD	8	23.3	9.22E+06	1.28E+07	1.25E+07	2.42E+07	1.43E+07	1.29E+07				23.11	23.8	23.82	24.58	23.92	23.84				23.8	23.92		0.406802	0.406738				
358	B1AMA6	Target of r	MAPKAP1	2	21.3			1.59E+06		1.83E+06	1.73E+06	2.29E+06	2.72E+06				20.91		21.05	21	21.06	21.23		20.91	21.03	21.14		0.0376	0.124851			+
3227	Q9Y446	Plakophilin	PKP3	4	5.1	3.46E+06	3.14E+06	2.91E+06	3.88E+06	3.73E+06	2.79E+06	3.69E+06	3.52E+06		21.68	21.84	21.77	21.89	22.02	21.73	21.77	21.55		21.77	21.89	21.66	0.077536	0.142728	0.149097			
1357	P00747	Plasminoge	PLG	3	5.6	6.77E+07	3.13E+07	1.24E+07	3.89E+07	2.95E+07		3.44E+07			25.95	25.04	23.81	25.33	24.98		25.03			25.04	25.15	25.03	1.076705	0.248598		+		
1518	P13995	Bifunctiona	MTHFD2	6	30.3	1.55E+07	1.22E+07	1.22E+07	1.89E+07	1.41E+07	1.34E+07	1.07E+07	1.62E+07	1.02E+07	23.83	23.74	23.78	24.22	23.9	23.89	23.27	23.75	22.83	23.78	23.9	23.27	0.046337	0.188279	0.460462			
1196	O43592	Exportin-T	XPOT	26	32.2	8.79E+07	1.07E+08	6.77E+07	8.95E+07	9.27E+07	7.16E+07	6.81E+07	8.07E+07	5.29E+07	26.46	26.95	26.36	26.57	26.69	26.36	25.92	26.06	25.17	26.46	26.57	25.92	0.316094	0.17032	0.478501			
2929	Q9H2U1-3	ATP-depen	DHX36	14	18.3	1.44E+07	1.72E+07	2.34E+07	1.19E+07	1.90E+07	2.27E+07	1.27E+07	3.01E+07		23.72	24.23	24.73	23.5	24.34	24.65	23.52	24.68		24.23	24.34	24.1	0.500335	0.590875	0.821839			
2132	Q12769	Nuclear po	NUP160	30	27	5.45E+07	4.85E+07	6.18E+07	4.15E+07	5.12E+07	6.11E+07	2.66E+07	2.88E+07	7.64E+06	25.68	25.69	26.15	25.42	25.8	26.09	24.66	24.61	22.39	25.69	25.8	24.61	0.27093	0.337411	1.296885			
332	A8MT02	Small nucle	SNRPB	7	20.5	5.58E+07	5.03E+07	4.78E+07	6.37E+07	4.45E+07	5.27E+07	4.75E+07	5.12E+07	5.25E+07	25.71	25.77	25.8	26.02	25.56	25.88	25.46	25.41	25.16	25.77	25.88	25.41	0.041967	0.234455	0.163903			
3114	Q9UDY4	DnaJ homc	DNAJB4	4	19.6	4.96E+06	4.46E+06	3.44E+06	4.22E+06	4.44E+06	4.32E+06	4.08E+06	4.66E+06	4.42E+06	22.17	22.34	22	22.02	22.29	22.32	21.93	21.94	21.43	22.17	22.29	21.93	0.168665	0.163788	0.292466			
827	H7C072	THO comp	THOC5	2	9.5			2.37E+06			2.54E+06						21.47			21.59				21.47	21.59							
1348	P00374-2	Dihydrofol	DHFR	3	30.4		1.08E+07	8.76E+06	1.26E+07	1.12E+07	9.80E+06					23.55	23.33	23.59	23.55	23.47				23.44	23.55		0.156057	0.064941				
3148	Q9UKF6	Cleavage a	CPSF3	5	10.1	1.05E+07	7.88E+06	6.87E+06	9.81E+06	8.53E+06	8.04E+06	7.59E+06	7.80E+06		23.3	23.09	22.98	23.24	23.18	23.21	22.78	22.68		23.09	23.21	22.73	0.163329	0.028094	0.072449			
1612	P25325	3-mercapto	MPST	3	12.8			2.68E+06		2.26E+06	3.93E+06						21.65		21.32	22.19				21.65	21.76			0.616003				
3219	Q9Y3F4	Serine-thre	STRAP	15	50.9	4.71E+07	5.58E+07	4.38E+07	4.97E+07	4.91E+07	4.36E+07	4.89E+07	4.73E+07	5.20E+07	25.46	25.98	25.6	25.72	25.71	25.65	25.52	25.31	25.14	25.6	25.71	25.31	0.26562	0.042264	0.191644			
2153	Q13131	5-AMP-act	PRKAA1	6	14.1	8.98E+06	7.34E+06	7.11E+06	9.14E+06	7.71E+06	9.36E+06	7.72E+06			23.07	23	23.02	23.14	23.06	23.41	22.83			23.02	23.14	22.83	0.034286	0.184448				
1305	Q4G104	F-box only	FBXO21	3	9.5	3.46E+06	3.16E+06	3.01E+06	4.56E+06	3.34E+06	3.26E+06	4.01E+06			21.68	21.85	21.83	22.12	21.88	21.94	21.89			21.83	21.94	21.89	0.090922	0.129365		+		
635	E7EPW2	28S riboso	MRPS25	3	30.9	2.53E+06	1.95E+06	4.10E+06	2.16E+06	2.79E+06	2.11E+06				21.23	21.2	22.28	21.05	21.65	21.34				21.23	21.34		0.615447	0.301595				
2219	Q14318	Peptidyl-pr	FKBP8	3	11.4	4.30E+06	5.35E+06	4.27E+06	3.64E+06	4.98E+06	4.99E+06				21.99	22.56	22.34	21.78	22.45	22.51				22.34	22.45		0.287041	0.401722				
1225	O60518	Ran-bindin	RANBP6	3	5.1	1.48E+06	1.12E+06	1.08E+06	1.03E+06	1.27E+06	1.16E+06				20.42	20.43	20.39	20.14	20.52	20.54				20.42	20.52		0.017602	0.223125				
2693	Q969N2-5	GPI transa	PIGT	7	13.4	5.80E+06	5.08E+06	5.38E+06	6.10E+06	5.55E+06	6.36E+06	5.04E+06			22.39	22.5	22.69	22.57	22.6	22.85	22.22			22.5	22.6	22.22	0.152309	0.152964				
2862	Q9BVA6	Adenosine	FICD	5	11.4	2.55E+06	3.74E+06	2.64E+06		3.21E+06	2.63E+06		2.41E+06		21.25	22.12	21.63		21.83	21.64		21.03		21.63	21.73	21.03	0.436931	0.132827				
2580	Q8NBN3-3	Transmem	TMEM87A	3	8.5			1.79E+06	2.91E+06	2.15E+06	1.81E+06	2.35E+06	2.85E+06				21.14	21.51	21.24	21.09	21.09	21.29		21.14	21.24	21.19		0.211705	0.146212	+		
1112	O00425	Insulin-like	IGF2BP3	11	24.9	1.13E+07	1.07E+07	1.09E+07	1.24E+07	1.18E+07	1.20E+07	1.63E+07	1.75E+07	1.95E+07	23.41	23.53	23.64	23.57	23.63	23.69	23.93	23.86	23.81	23.53	23.63	23.86	0.114401	0.060447	0.060631			+
1345	O96019-2	Actin-like p	ACTL6A	10	34.9	2.74E+07	2.50E+07	2.30E+07	3.05E+07	2.11E+07	2.57E+07	2.38E+07	2.25E+07	1.99E+07	24.65	24.73	24.69	24.89	24.49	24.79	24.49	24.24	23.85	24.69	24.79	24.24	0.0369	0.208111	0.322053			
1925	P55265-5	Double-str	ADAR	12	15.8	9.46E+06	9.45E+06	9.11E+06	5.82E+06	1.03E+07	1.15E+07	4.07E+06	1.66E+06		23.14	23.36	23.39	22.49	23.46	23.64	21.92	20.43		23.36	23.46	21.17	0.133439	0.618457	1.059182			
760	F5H0F9	Anaphase-	ANAPC5	4	8		3.04E+06	2.97E+06	3.55E+06	3.42E+06	3.15E+06	3.12E+06	4.02E+06			21.78	21.81	21.75	21.91	21.9	21.52	21.74		21.8	21.9	21.63	0.016997	0.091478	0.153551			
943	H3BSS5	BTB/POZ d	KCTD5	3	15	5.64E+06	4.56E+06	4.33E+06	3.87E+06		7.26E+06	2.47E+06			22.34	22.37	22.37	21.89		23.05	21.15			22.37	22.47	21.15	0.016	0.826445				
2787	Q96ST3	Paired amp	SIN3A	5	5.2	2.94E+06	1.82E+06	1.89E+06	2.50E+06	2.57E+06	1.81E+06	1.92E+06			21.47	21.1	21.2	21.3	21.5	21.09	20.76			21.2	21.3	20.76	0.193294	0.206486				
1734	P36957	Dihydrolipo	DLST	9	23.4	3.75E+07	3.65E+07	3.48E+07	4.28E+07	3.05E+07	3.58E+07	4.42E+07	4.51E+07	2.85E+07	25.13	25.31	25.27	25.46	25.02	25.36	25.34	25.22	24.29	25.27	25.36	25.22	0.09264	0.232564	0.576357	+		
178	A0A0A0MS	Chromodo	CHD1L	8	10.5	3.93E+06		4.08E+06	4.31E+06	4.20E+06	4.40E+06	2.60E+06	4.06E+06		21.87		22.28	22.05	22.17	22.34	21.22	21.77		22.08	22.17	21.49	0.288434	0.143497	0.385288			
58	A0A087W\	Adenylate	TAF9	2	16.5		1.71E+06	1.20E+06			1.58E+06					20.98	20.57			20.87				20.77	20.87		0.292227					
2340	Q3V6T2-4	Girdin	CCDC88A	83	43.4	5.08E+08	5.36E+08	4.05E+08	6.81E+08	5.48E+08	4.67E+08	3.74E+08	2.83E+08	8.56E+07	28.9	29.37	29.14	29.27	29.24	29.13	28.37	27.72	25.84	29.14	29.24	27.72	0.235961	0.074068	1.312786			
2106	Q07666	KH domain	KHDRBS1	6	13.5	1.02E+07	1.86E+07	1.77E+07	9.55E+06	2.00E+07	1.84E+07	8.71E+06	7.13E+06		23.27	24.3	24.31	23.19	24.43	24.4	23	22.53		24.3	24.4	22.76	0.601975	0.703807	0.332085			
1311	O95155-3	Ubiquitin c	UBE4B	4	5.3	1.82E+06	2.09E+06	1.56E+06	1.36E+06	1.71E+06	1.69E+06				20.72	21.3	20.88	20.39	20.99	20.97				20.88	20.97		0.302594	0.339248				
2051	P98179	RNA-bindir	RBM3	2	21		1.26E+07	1.79E+07	1.80E+07	1.10E+07	1.76E+07					23.78	24.35	24.16	23.53	24.31				24.06	24.16		0.402269	0.414299				
3066	Q9NZI8	Insulin-like	IGF2BP1	5	16.1	1.14E+07	1.15E+07	1.27E+07	1.05E+07	1.26E+07	1.37E+07	1.46E+07	1.82E+07	1.06E+07	23.42	23.64	23.85	23.33	23.74	23.93	23.77	23.92	22.9	23.64	23.74	23.77	0.213995	0.303881	0.551941			
2866	Q9BVP2-2	Guanine nu	GNL3	9	20.1	1.35E+07	8.14E+06	8.70E+06	9.82E+06	9.98E+06	9.64E+06	1.57E+07	1.18E+07	5.61E+06	23.63	23.14	23.32	23.24	23.41	23.45	23.85	23.28	21.87	23.32	23.41	23.28	0.249799	0.111726	1.020564	+		
2802	Q99570	Phosphoin	PIK3R4	7	6.8	4.56E+06	3.71E+06	3.72E+06	4.55E+06	4.25E+06	4.47E+06	4.78E+06	5.81E+06		22.05	22.1	22.16	22.12	22.19	22.36	22.13	22.24		22.1	22.19	22.19	0.057532	0.124688	0.076617		+	
2038	P82664	28S riboso	MRPS10	2	16.9	2.03E+06	2.63E+06	3.14E+06	2.50E+06	3.80E+06	2.69E+06	2.63E+06	2.27E+06		20.87	21.58	21.89	21.3	22.05	21.67	21.23	20.94		21.58	21.67	21.09	0.526031	0.377486	0.201865			
795	F8VYH9	Cyclin-dep	CDK4	5	36.5	5.92E+06	1.05E+07	6.99E+06	1.02E+07	6.42E+06	7.46E+06	5.56E+06	4.96E+06		22.42	23.5	23	23.29	22.81	23.09	22.38	22.02		23	23.09	22.2	0.543709	0.240464	0.253612			
2182	Q13547	Histone de	HDAC1	11	31.5	7.83E+07	8.60E+07	8.85E+07	9.76E+07	7.52E+07	1.03E+08	6.84E+07	8.13E+07	4.26E+07	26.25	26.57	26.74	26.66	26.36	26.92	25.94	26.09	24.89	26.57	26.66	25.94	0.244946	0.281222	0.650904			
662	E7EVJ5	Cytoplasm	CYFIP2	2	11.2		7.31E+05	9.76E+05			8.24E+05					19.87	20.26			20.16				20.07	20.16		0.275206			+		
89	A0A087W\	Ubiquitin-c	UBE2D3	3	24.3	2.93E+07	3.35E+07	1.74E+07		2.70E+07					24.78	25.18	24.28		24.87					24.78	24.87		0.451258					
1252	O75306-2	NADH deh	NDUFS2	3	6.1		1.64E+06	1.49E+06			1.69E+06					20.93	20.83			20.97				20.88	20.97		0.064082					
2806	Q99661-2	Kinesin-like	KIF2C	2	6.1	2.65E+06	1.96E+06	2.78E+06		2.46E+06	2.09E+06	2.03E+06			21.3	21.21	21.7		21.45	21.32	20.81			21.3	21.38	20.81	0.262153	0.096985				
1899	P53007	Tricarboxyl	SLC25A1	5	14.1	5.39E+06	3.43E+06	3.07E+06		3.79E+06	3.62E+06				22.27	21.97	21.86		22.04	22.08				21.97	22.06		0.211617	0.026453				
1723	P36405	ADP-ribosy	ARL3	6	46.7	1.30E+07	1.49E+07	1.63E+07	1.57E+07	1.87E+07	1.52E+07	9.61E+06	9.80E+06		23.6	24	24.18	23.94	24.32	24.09	23.14	23.01		24	24.09	23.08	0.298505	0.188332	0.095571			
288	A0A140T9	Antigen pe	TAP1	4	10.5		2.57E+06	4.08E+06	3.39E+06	3.67E+06	3.48E+06	3.72E+06	3.32E+06			21.54	22.28	21.7	22	22.03	21.78	21.49		21.91	22	21.64	0.521272	0.186465	0.206151			
3216	Q9Y3C8	Ubiquitin-f	UFC1	3	16.2	7.62E+06	6.96E+06	7.20E+06	1.00E+07	6.73E+06	7.15E+06	8.26E+06	6.24E+06	8.39E+06	22.79	22.94	23.06	23.26	22.89	23.03	22.91	22.33	22.52	22.94	23.03	22.52	0.137841	0.190274	0.294898			
2148	Q13042-3	Cell divisio	CDC16	6	12.5	5.46E+06	3.89E+06	4.70E+06	4.01E+06	4.72E+06	5.22E+06		5.26E+06		22.29	22.18	22.47	21.94	22.38	22.59		22.07		22.29	22.38	22.07	0.148178	0.329768				
237	E7ESL0	39S riboso	MRPL22	3	15.1	2.73E+06	3.48E+06	3.83E+06	6.84E+06		2.27E+06		3.98E+06		21.35	22	22.21	22.74		21.44		21.73		22	22.09	21.73	0.449628	0.920379				
1565	P20020-6	Plasma me	ATP2B1	10	12.3	9.67E+06	8.13E+06	5.83E+06	9.73E+06	9.03E+06	6.41E+06	9.78E+06			23.18	23.13	22.8	23.22	23.25	22.87	23.17			23.13	23.22	23.17	0.205933	0.212007		+		
931	H3BPE1	Microtubu	MACF1	48	8.3	2.50E+07	2.89E+07	3.67E+07	2.78E+07	3.11E+07	3.22E+07	4.80E+07	1.02E+08	1.63E+07	24.52	24.95	25.37	24.78	25.04	25.21	25.48	26.39	23.59	24.95	25.04	25.48	0.424398	0.214929	1.429015			+
1756	P40937-2	Replicatior	RFC5	10	42.6	1.84E+07	2.02E+07	1.61E+07	1.76E+07	1.78E+07	1.80E+07	1.70E+07	1.83E+07		24.12	24.4	24.16	24.1	24.24	24.37	24	23.93		24.16	24.24	23.97	0.153538	0.136021	0.046871			
2732	Q96EY7	Pentatrico	PTCD3	4	6.8	4.40E+06	2.89E+06	4.45E+06	5.01E+06	3.88E+06	3.64E+06	3.45E+06	3.81E+06		22.01	21.71	22.39	22.26	22.09	22.1	21.66	21.67		22.01	22.1	21.66	0.344146	0.099071	0.009586			
1913	P54577	Tyrosine--t	YARS	19	41.7	2.94E+07	4.40E+07	4.41E+07	3.13E+07	4.83E+07	4.56E+07	2.90E+07	2.88E+07	1.02E+07	24.79	25.61	25.62	24.95	25.7	25.69	24.78	24.61	22.83	25.61	25.69	24.61	0.474912	0.433466	1.079215			
604	D6RFH4	Cytochrom	CYB5B	3	42	7.22E+06		9.11E+06			7.62E+06				22.72		23.39			23.13				23.05	23.13		0.47309					
689	Q504U8	Receptor p	EGFR	9	10.2	8.90E+06	7.81E+06	9.77E+06	8.01E+06	8.37E+06	8.39E+06	6.34E+06	8.43E+06		23.05	23.08	23.5	22.93	23.16	23.25	22.55	22.79		23.08	23.16	22.67	0.251485	0.16387	0.166554			
2059	Q00688	Peptidyl-pr	FKBP3	7	44.2	3.85E+06	5.69E+06	4.60E+06			5.08E+06	2.94E+06			21.85	22.63	22.45			22.53	21.44			22.45	22.53	21.44	0.407658					
3	A0A024R5	EH domain	EHD1	11	29.9	9.85E+06	1.30E+07	1.15E+07	1.03E+07	1.32E+07	1.50E+07	1.42E+07	1.04E+07	1.34E+07	23.2	23.82	23.74	23.32	23.82	24.06	23.72	23.08	23.28	23.74	23.82	23.28	0.335419	0.379185	0.325535			
1879	P51812	Ribosomal	RPS6KA3	16	27	2.08E+07	1.74E+07	1.63E+07	1.85E+07	1.87E+07	2.03E+07	1.67E+07	9.74E+06		24.26	24.24	24.18	24.2	24.32	24.5	23.96	22.99		24.24	24.32	23.47	0.042133	0.15502	0.682527			
3023	Q9NT62-2	Ubiquitin-l	ATG3	4	13.8	2.38E+06	3.63E+06	1.79E+06			1.96E+06	2.25E+06	1.82E+06		21.12	22.05	21.14			21.22	21.03	20.62		21.14	21.22	20.83	0.533801		0.290274			

1866	P51114-2	Fragile X m	FXR1	11	28.4	3.86E+07	3.57E+07	3.47E+07	3.89E+07	4.37E+07	3.19E+07	3.09E+07	2.01E+07	1.66E+07	25.17	25.28	25.25	25.33	25.54	25.18	24.85	24.09	23.62	25.25	25.33	24.09	0.056177	0.180569	0.622444			
1873	P51570	Galactokin	GALK1	7	24	1.37E+07	8.96E+06	7.09E+06	1.06E+07	1.02E+07	6.92E+06	1.13E+07	1.65E+07	1.53E+07	23.65	23.26	23.02	23.34	23.45	22.98	23.34	23.78	23.48	23.26	23.34	23.48	0.31745	0.242986	0.222665			+
3014	Q9NRW3	DNA dC->d	APOBEC3C	2	13.7	3.72E+06	3.57E+06	3.60E+06	3.67E+06		4.71E+06				21.79	22.04	22.1	21.8		22.43				22.04	22.12		0.162577	0.444424				
997	J3KNL6	Protein tra	SEC16A	9	5.4	1.13E+07	7.22E+06	7.83E+06	9.59E+06	1.42E+07	8.54E+06	5.11E+06	5.92E+06		23.41	22.98	23.19	23.21	23.9	23.27	22.24	22.26		23.19	23.27	22.25	0.217837	0.387753	0.0185			
2914	Q9H0F7	ADP-ribosy	ARL6	4	33.3	5.27E+06	6.14E+06	6.38E+06	6.05E+06	6.39E+06	6.30E+06	4.11E+06	4.87E+06		22.24	22.73	22.9	22.56	22.81	22.85	21.94	22		22.73	22.81	21.97	0.3413	0.156352	0.040159			
3086	Q9P265	Disco-inter	DIP2B	7	5.5	3.72E+06	3.03E+06	3.34E+06	2.76E+06	3.32E+06	3.43E+06	3.13E+06			21.79	21.78	21.97	21.42	21.87	22.01	21.54			21.79	21.87	21.54	0.1054	0.307305				
3045	Q9NWU2	Glucose-in	GID8	4	23.7	6.90E+06	6.46E+06	6.67E+06	9.44E+06	6.74E+06	5.56E+06	4.96E+06	6.87E+06	7.66E+06	22.65	22.81	22.93	23.18	22.89	22.68	22.19	22.47	22.39	22.81	22.89	22.39	0.14349	0.254029	0.144115			
1098	O00178	GTP-bindir	GTPBP1	3	5.7	2.93E+06	2.82E+06	2.53E+06	3.22E+06	2.59E+06	3.85E+06	2.24E+06			21.46	21.65	21.57	21.65	21.51	22.16	21.01			21.57	21.65	21.01	0.093153	0.341475				
628	E7EMB6	Aspartyl ar	DNPEP	2	6.3	1.74E+06	1.77E+06	1.94E+06	2.27E+06	1.93E+06	2.06E+06	2.46E+06	2.24E+06	2.04E+06	20.62	21.06	21.24	21.13	21.1	21.29	21.15	20.93	20.18	21.06	21.13	20.93	0.314536	0.102838	0.506			
796	F8VTY2	Nucleopori	NUP37	4	23.9	7.96E+06	9.75E+06	8.15E+06	1.10E+07	9.40E+06	7.30E+06	7.97E+06	6.82E+06	6.12E+06	22.87	23.39	23.25	23.38	23.32	23.06	22.86	22.46	22.01	23.25	23.32	22.46	0.273146	0.167169	0.426216			
801	F8VXC8	SWI/SNF co	SMARCC2	9	17.2	1.62E+07	1.66E+07	1.68E+07	1.96E+07	1.72E+07	1.66E+07	2.03E+07	1.51E+07	9.15E+06	23.91	24.14	24.23	24.27	24.21	24.22	24.29	23.64	22.64	24.14	24.22	23.64	0.163022	0.032927	0.833233			
3268	R4GNH3	26S protea	PSMC3	21	57.9	1.49E+08	1.56E+08	1.32E+08	1.39E+08	1.68E+08	1.45E+08	1.35E+08	1.86E+08	1.02E+08	27.22	27.63	27.39	27.26	27.47	27.56	27.01	27.26	26.06	27.39	27.47	27.01	0.204206	0.150013	0.635896			
197	A0A0A0MT	Cullin-2	CUL2	4	6.1		2.46E+06	2.71E+06	1.84E+06	2.79E+06	3.44E+06	2.20E+06				21.5	21.66	20.82	21.65	22.02	20.96			21.58	21.65	20.96	0.11371	0.615962				
2189	Q13637	Ras-relatec	RAB32	5	23.1	6.02E+06	5.93E+06	6.22E+06	6.82E+06	8.30E+06	5.81E+06	5.20E+06	4.45E+06	6.01E+06	22.45	22.67	22.87	22.73	23.14	22.75	22.27	21.88	21.97	22.67	22.75	21.97	0.208502	0.227917	0.206501			
1977	P62263	40S ribosoi	RPS14	8	40.4	9.30E+07	1.07E+08	8.00E+07	9.96E+07	6.21E+07	9.21E+07	9.29E+07	9.86E+07	8.44E+07	26.57	26.95	26.62	26.69	26.08	26.71	26.49	26.34	25.78	26.62	26.69	26.34	0.206684	0.356178	0.372575			
2379	Q5RKV6	Exosome c	EXOSC6	4	25	6.46E+06	6.48E+06	8.57E+06	3.03E+06	7.80E+06	6.51E+06	6.01E+06			22.56	22.81	23.3	21.55	23.07	22.89	22.49			22.81	22.89	22.49	0.379604	0.826454				
2793	Q96TA2-3	ATP-depen	YME1L1	3	6.4		1.66E+06	1.71E+06			1.80E+06					20.95	21.06			21.07				21	21.07		0.077809					
3164	Q9UN86-2	Ras GTPase	G3BP2	6	18.7	4.19E+06	9.10E+06	1.14E+07	6.89E+06	1.01E+07	9.08E+06	5.68E+06	7.19E+06		21.96	23.29	23.72	22.75	23.43	23.36	22.41	22.54		23.29	23.36	22.48	0.920109	0.376773	0.093891			
1889	P52597	Heterogen	HNRNPF	10	43.6	1.09E+08	1.04E+08	8.72E+07	1.15E+08	1.18E+08	9.62E+07	8.45E+07	1.38E+08	7.31E+07	26.81	26.88	26.71	26.88	27	26.78	26.31	26.81	25.55	26.81	26.88	26.31	0.087745	0.111636	0.63389			
1601	P23786	Carnitine C	CPT2	3	7.1		1.88E+06	2.21E+06			2.11E+06					21.14	21.4			21.34				21.27	21.34		0.184897					
410	F8W118	Nucleosom	NAP1L1	7	34	1.82E+07	2.75E+07	2.74E+07	2.42E+07	2.87E+07	3.40E+07	1.87E+07	2.22E+07	1.43E+07	24.1	24.89	24.9	24.58	24.96	25.29	24.13	24.22	23.38	24.89	24.96	24.13	0.459072	0.357344	0.462102			
2848	Q9BTE3-3	Mini-chron	MCMBP	5	11.3	4.79E+06	6.11E+06	7.89E+06	5.73E+06	7.26E+06	6.04E+06	4.57E+06			22.11	22.72	23.2	22.47	22.98	22.79	22.09			22.72	22.79	22.09	0.544097	0.260406				
3194	Q9Y2H6-2	Fibronectir	FNDC3A	6	7.2	4.44E+06	1.80E+06	3.23E+06	4.17E+06	4.23E+06	3.33E+06	1.56E+06	2.59E+06		22.02	21.08	21.93	22	22.19	21.97	20.52	21.12		21.93	22	20.82	0.518328	0.119167	0.424091			
3248	Q9Y5Q9	General tra	GTF3C3	8	12.2	4.12E+06	5.28E+06	3.71E+06	4.51E+06	4.32E+06	4.09E+06	4.18E+06	3.75E+06		21.93	22.54	22.16	22.1	22.23	22.25	21.97	21.65		22.16	22.23	21.81	0.307281	0.081542	0.226948			
1186	O43242	26S protea	PSMD3	21	44	9.19E+07	9.07E+07	9.11E+07	8.34E+07	1.18E+08	9.55E+07	8.96E+07	9.86E+07	2.66E+07	26.56	26.69	26.8	26.47	27	26.75	26.4	26.34	24.19	26.69	26.75	26.34	0.120735	0.268258	1.259054			
2852	Q9BU76-3	Multiple m	MMTAG2	3	17.4	4.42E+06	3.55E+06	2.86E+06	4.42E+06	3.96E+06	3.36E+06	3.80E+06	2.94E+06		22.02	22.03	21.76	22.09	22.11	21.98	21.81	21.33		22.02	22.09	21.57	0.152127	0.068822	0.333188			
680	E9PDF6	Unconvent	MYO1B	23	24.1	2.34E+07	2.54E+07	2.51E+07	2.61E+07	2.71E+07	2.63E+07	1.91E+07	2.25E+07	1.00E+07	24.44	24.77	24.81	24.7	24.88	24.84	24.18	24.24	22.8	24.77	24.84	24.18	0.204563	0.095307	0.818544			
2305	Q15904	V-type pro	ATP6AP1	2	5.3	2.00E+06	1.08E+06	1.21E+06	1.68E+06	1.36E+06	1.46E+06				20.85	20.42	20.58	20.65	20.63	20.77				20.58	20.65		0.216103	0.077317				
3082	Q9P219	Protein Da	CCDC88C	96	48.1	7.96E+08	7.17E+08	6.42E+08	1.07E+09	8.57E+08	7.11E+08	8.69E+08	9.97E+08	6.88E+08	29.62	29.84	29.77	30.24	29.84	29.7	29.61	29.83	28.72	29.77	29.84	29.61	0.111408	0.283143	0.586706			
1307	O94991	SLIT and N	SLITRK5	20	24.4	2.51E+07	3.15E+07	3.02E+07	2.46E+07	3.23E+07	3.30E+07	2.07E+07	2.66E+07		24.53	25.04	25.05	24.62	25.11	25.26	24.32	24.48		25.04	25.11	24.4	0.295747	0.335816	0.115948			
1884	P52292	Importin su	KPNA2	6	15.7	4.93E+06	4.14E+06	5.12E+06	5.42E+06	4.41E+06	4.33E+06	4.35E+06	4.90E+06		22.16	22.26	22.59	22.37	22.26	22.33	22.02	22.01		22.26	22.33	22.02	0.224292	0.053124	0.008862			
1247	O75116	Rho-associ	ROCK2	16	22	9.15E+06	7.69E+06	9.19E+06	7.09E+06	8.36E+06	8.32E+06	1.01E+07	5.52E+06		23.09	23.06	23.41	22.79	23.15	23.24	23.21	22.17		23.09	23.15	22.69	0.192055	0.240969	0.734962			
3081	Q9P1Y5	Calmodulir	CAMSAP3	6	8	4.82E+06	3.07E+06	3.19E+06	3.65E+06	3.97E+06	3.35E+06	4.85E+06	6.00E+06		22.12	21.8	21.91	21.79	22.11	21.97	22.15	22.28		21.91	21.97	22.22	0.165997	0.161114	0.091468			+
588	D6REC6	Cyclin-depe	CDK7	2	16.1	4.23E+06	3.22E+06			3.82E+06	3.22E+06				21.97	21.88			22.06	21.92				21.93	21.99		0.064094	0.100329				
118	A0A087X0I	Nucleolar p	NOL4L	4	5.4		2.03E+06	2.41E+06		2.26E+06	2.49E+06					21.25	21.51		21.32	21.56				21.38	21.44		0.17768	0.169726				
1465	P0DN79	Cystathion	CBS	7	16.7	8.83E+06	7.86E+06	7.99E+06	9.23E+06	7.75E+06	8.67E+06	7.41E+06	5.77E+06		23.03	23.08	23.22	23.14	23.06	23.28	22.75	22.23		23.08	23.14	22.49	0.094189	0.112451	0.369222			
1488	P11388	DNA topois	TOP2A	16	12.3	1.40E+07	1.20E+07	1.44E+07	1.38E+07	1.29E+07	1.59E+07	8.83E+06	1.33E+07	2.96E+06	23.68	23.72	24	23.74	23.78	24.15	23.02	23.45	20.91	23.72	23.78	23.02	0.172768	0.222639	1.358453			
1003	J3KPP6	RUN and F	RUFY1	4	11.7		3.34E+06	3.40E+06	3.47E+06	3.75E+06	3.45E+06	3.69E+06				21.94	21.98	21.72	22.03	22.02	21.77			21.96	22.02	21.77	0.029769	0.177878				
1965	P61964	WD repeat	WDR5	7	32	2.15E+07	2.24E+07	1.41E+07	2.08E+07	2.68E+07	1.66E+07	2.03E+07	2.46E+07		24.31	24.58	23.97	24.37	24.85	24.22	24.29	24.38		24.31	24.37	24.34	0.306212	0.333175	0.060365	+		
658	E7EU96	Casein kina	CSNK2A1	10	37.1	1.32E+07	2.52E+07	1.88E+07	1.28E+07	2.02E+07	2.15E+07	1.23E+07	9.45E+06	6.13E+06	23.61	24.75	24.39	23.63	24.44	24.57	23.46	22.94	22.02	24.39	24.44	22.94	0.581838	0.509742	0.731939			
3022	Q9NSV4	Protein dia	DIAPH3	11	10.8	5.62E+06	7.36E+06	5.60E+06	5.82E+06	6.52E+06	6.11E+06	5.74E+06	6.33E+06	6.33E+06	22.33	23	22.75	22.49	22.83	22.81	22.42	22.35	22.09	22.75	22.81	22.35	0.338338	0.191726	0.173535			
2137	Q12824	SWI/SNF-re	SMARCB1	4	16.4	1.50E+07	1.12E+07	9.70E+06	1.70E+07	1.20E+07	1.04E+07	9.18E+06	1.18E+07		23.79	23.61	23.48	24.07	23.66	23.53	23.08	23.28		23.61	23.66	23.18	0.154739	0.278639	0.146314			
2712	Q96BW9-3	Phosphatic	TAMM41	2	7.4		1.27E+06	1.08E+06		1.20E+06	1.33E+06		1.29E+06			20.6	20.39		20.42	20.68		20.15		20.5	20.55	20.15	0.145231	0.184024				
917	H0YNG3	Signal pept	SEC11A	3	17.8	6.29E+06	7.98E+06	8.95E+06	7.73E+06	9.44E+06	7.80E+06				22.52	23.1	23.35	22.9	23.33	23.16				23.1	23.16		0.430643	0.217161				
2868	Q9BW27	Nuclear po	NUP85	15	28.8	7.07E+07	5.68E+07	6.70E+07	7.00E+07	6.34E+07	5.59E+07	5.08E+07	5.23E+07	3.43E+07	26.08	26.03	26.32	26.14	26.13	25.93	25.58	25.46	24.61	26.08	26.13	25.46	0.159588	0.116692	0.532961			
1081	M0R300	Unconvent	MYO9B	10	5.9	5.27E+06	3.94E+06	3.92E+06	4.62E+06	5.07E+06	4.22E+06	6.36E+06	5.56E+06		22.24	22.2	22.24	22.15	22.48	22.29	22.56	22.18		22.24	22.29	22.37	0.020253	0.166338	0.267948			+
1531	P15924	Desmoplak	DSP	66	26.2	9.11E+07	9.39E+07	6.83E+07	1.38E+08	8.65E+07	6.60E+07	1.23E+08	8.00E+07	8.84E+07	26.53	26.72	26.37	27.25	26.58	26.2	26.88	26.06	25.94	26.53	26.58	26.06	0.175794	0.52949	0.512947			
3183	Q9Y230	RuvB-like 2	RUVBL2	18	46	7.80E+07	8.70E+07	8.64E+07	9.50E+07	8.09E+07	8.88E+07	8.20E+07	8.26E+07	6.26E+07	26.25	26.59	26.69	26.64	26.45	26.67	26.25	26.12	25.37	26.59	26.64	26.12	0.23088	0.117125	0.474569			
3025	Q9NTJ3	Structural i	SMC4	39	35	8.56E+07	7.60E+07	7.52E+07	8.47E+07	8.43E+07	7.11E+07	8.52E+07	5.06E+07	6.50E+07	26.36	26.43	26.53	26.48	26.53	26.33	26.33	25.4	25.41	26.43	26.48	25.41	0.083253	0.101374	0.533148			
2708	Q96B26	Exosome c	EXOSC8	2	10.1	1.81E+06	3.26E+06	3.57E+06	4.02E+06	3.13E+06	4.04E+06	3.47E+06			20.7	21.89	22.08	21.94	21.8	22.24	21.66			21.89	21.94	21.66	0.75041	0.224344				
2639	Q8WVM8	Sec1 family	SCFD1	13	31	2.93E+07	2.21E+07	2.62E+07	2.32E+07	2.65E+07	2.64E+07	2.28E+07	2.96E+07	2.32E+07	24.78	24.56	24.87	24.52	24.83	24.85	24.45	24.65	24.02	24.78	24.83	24.45	0.15567	0.181042	0.320411			
350	F2Z2V0	Copine-1	CPNE1	3	6.4	2.76E+06	2.71E+06	4.03E+06	2.34E+06	3.72E+06	2.64E+06	2.31E+06			21.36	21.6	22.26	21.19	22.02	21.65	21.07			21.6	21.65	21.07	0.470207	0.41293				
1789	P46060	Ran GTPas	RANGAP1	22	43.3	4.83E+07	3.86E+07	4.57E+07	3.55E+07	4.36E+07	4.24E+07	3.16E+07	2.31E+07		25.49	25.41	25.7	25.19	25.54	25.58	24.9	24.27		25.49	25.54	24.59	0.147387	0.212181	0.443265			
125	A0A0A0MS	RecQ-med	RMI1	3	9.2	1.96E+06	1.05E+06	1.67E+06	1.55E+06	5.33E+06	1.59E+06	1.39E+06			20.83	20.36	21	20.52	22.56	20.87	20.29			20.83	20.87	20.29	0.328661	1.088624				
2400	Q5UIP0-2	Telomere-a	RIF1	16	10.3	1.25E+07	1.39E+07	1.46E+07	1.62E+07	1.29E+07	1.39E+07	1.34E+07	1.08E+07		23.55	23.9	24.02	24.01	23.78	23.95	23.59	23.13		23.9	23.95	23.36	0.243638	0.118276	0.326849			
1319	O95340	Bifunctiona	PAPSS2	11	19.7	1.85E+07	1.23E+07	1.35E+07	1.38E+07	1.48E+07	1.44E+07	9.59E+06	1.19E+07		24.13	23.75	23.92	23.74	23.97	24	23.14	23.31		23.92	23.97	23.22	0.192067	0.140318	0.120652			
1456	P09543-2	2,3-cyclic-r	CNP	23	45.4	1.98E+08	1.83E+08	1.58E+08	2.14E+08	1.62E+08	1.84E+08	1.74E+08	2.14E+08	1.35E+08	27.69	27.91	27.58	27.74	27.42	27.89	27.38	27.44	26.35	27.69	27.74	27.38	0.171099	0.2373	0.612996			
459	C9JG87	39S ribosoi	MRPL39	8	25.9	1.50E+07	1.49E+07	1.50E+07	1.43E+07	1.79E+07	1.48E+07	1.08E+07	1.59E+07	1.00E+07	23.79	24	24.06	23.8	24.25	24.04	23.29	23.73	22.8	24	24.04	23.29	0.139117	0.228373	0.465241			
2590	Q8NDZ4	Deleted in	C3orf58	3	6.3	2.81E+06	2.52E+06	1.77E+06	2.50E+06	2.54E+06	2.22E+06	4.65E+06	6.14E+06	2.41E+06	21.38	21.53	21.11	21.3	21.49	21.42	22.11	22.32	20.57	21.38	21.42	22.11	0.208964	0.098073	0.951267			
2173	Q13409-6	Cytoplasmi	DYNC1I2	4	5.6		4.54E+06	4.96E+06		4.81E+06	5.27E+06	5.15E+06				22.36	22.55		22.41	22.6	22.25			22.46	22.5	22.25	0.136398	0.135391				
1847	P49902-2	Cytosolic p	NT5C2	15	39.3	1.07E+08	1.09E+08	1.14E+08	1.18E+08	1.20E+08	1.31E+08	9.85E+07	7.24E+07	3.28E+07	26.77	26.99	27.07	26.91	27.03	27.39	26.57	25.91	24.53	26.99	27.03	25.91	0.154953	0.251649	1.040337			
822	F8WA39	Myotubula	MTMR1	15	26.4	4.16E+07	2.75E+07	2.82E+07	4.15E+07	2.96E+07	2.81E+07	6.20E+07	5.07E+07	3.84E+07	25.29	24.89	24.95	25.42	24.99	24.99	25.84	25.4	24.73	24.95	24.99	25.4	0.217921	0.248501	0.560784			
693	E9PGM9	RNA-bindir	RBM6	3	6.8	2.08E+06	2.86E+06	2.40E+06	2.23E+06	2.91E+06	2.46E+06	3.23E+06	3.03E+06		20.92	21.68	21.49	21.1	21.71	21.53	21.58	21.38		21.49	21.53	21.48	0.398472	0.315299	0.140475	+		
1318	O95336	6-phospho	PGLS	9	51.9	1.66E+07	2.50E+07	2.02E+07	1.95E+07	2.63E+07	2.06E+07	1.16E+07	1.19E+07	1.25E+07	23.95	24.73	24.48	24.26	24.81	24.52	23.38	23.31	23.16	24.48	24.52	23.31	0.399158	0.275636	0.11333			
270	A0A0J9YW	Peroxisom	DECR2	2	10.9			1.27E+06			1.38E+06						20.68			20.72				20.68	20.72							
3236	Q9Y4W2-2	Ribosomal	LAS1L	4	8.2	9.83E+06	6.93E+06	6.64E+06	8.72E+06	6.45E+06	6.88E+06	9.09E+06	9.03E+06		23.2	22.93	22.93	23.05	22.82	22.97	23.05	22.88		22.93	22.97	22.96	0.156302	0.117717	0.126231		+	
3146	Q9UKA4	A-kinase ar	AKAP11	11	7.1	1.01E+07	8.26E+06	6.05E+06	1.13E+07	8.64E+06	6.81E+06	1.27E+07	1.37E+07	1.07E+07	23.25	23.16	22.83	23.42	23.19	22.95	23.52	23.49	22.92	23.16	23.19	23.49	0.219233	0.236329	0.336732			
420	J3KMX2	SWI/SNF-re	SMARCD2	4	14.3	4.59E+06	3.30E+06	3.00E+06	4.06E+06	4.22E+06	3.04E+06	2.93E+06	3.12E+06		22.06	21.92	21.83	21.96	22.18	21.83	21.43	21.42		21.92	21.96	21.43	0.118635	0.180486	0.01102			
2504	Q86TI2	Dipeptidyl	DPP9	15	19.6	6.36E+07	4.25E+07	4.54E+07	5.59E+07	4.93E+07	4.24E+07	5.98E+07	4.20E+07	3.51E+07	25.86	25.56	25.69	25.83	25.73	25.58	25.8	25.11	24.64	25.69	25.73	25.11	0.152692	0.127226	0.585961			
852	G5E9G4	Tetratricop	TTC7A	4	5.7	9.92E+06	8.59E+06	9.05E+06		9.05E+06					23.22	23.21	23.37		23.25					23.22	23.25		0.094245					
1044	K7EK07	Histone H3	H3F3B	6	50	9.36E+08	6.13E+08	2.04E+08	8.04E+08	7.14E+08	4.60E+08	1.88E+09	1.41E+09	2.52E+09	29.93	29.49	28.14	29.65	29.53	29.1	30.9	30.4	30.13	29.49	29.53	30.4	0.936535	0.290118	0.39232			
62	A0A087W\	Tetratricop	TTC28	14	7.8	5.23E+06	5.51E+06	4.28E+06	5.35E+06	5.15E+06	4.52E+06	4.20E+06	3.82E+06		22.23	22.6	22.35	22.35	22.5	22.38	21.98	21.68		22.35	22.38	21.83	0.186546	0.078432	0.210749			
1275	O75616	GTPase Era	ERAL1	4	10.5	7.81E+06	7.59E+06	6.73E+06	8.98E+06	7.24E+06	5.86E+06	8.62E+06	9.28E+06	9.92E+06	22.82	23.04	22.94	23.1	22.97	22.75	22.98	22.91	22.78	22.94	22.97	22.91	0.111225	0.174722	0.10441			
2619	Q8TDY2-2	RB1-induci	RB1CC1	42	29.5	6.61E+07	5.35E+07	4.60E+07	7.83E+07	5.62E+07	5.15E+07	4.93E+07	6.04E+07	3.23E+07	25.91	25.89	25.71	26.33	25.92	25.86	25.54	25.65	24.5	25.89	25.92	25.54	0.110211	0.257997	0.630371			
1300	O94874	E3 UFM1-p	UFL1	13	18.4	1.16E+07	1.20E+07	1.19E+07	1.11E+07	1.29E+07	1.23E+07	1.36E+07	1.26E+07	7.69E+06	23.45	23.72	23.75	23.39	23.78	23.75	23.64	23.38	22.4	23.72	23.75	23.38	0.166776	0.215827	0.654559			
1056	K7EN45	Peptidyl-pr	PIN1	3	54.4	3.11E+06	6.16E+06	9.41E+06			5.95E+06				21.54	22.74	23.44			22.77				22.74	22.77		0.957742					
3034	Q9NVC6	Mediator c	MED17	4	7.8	4.51E+06	3.88E+06	3.49E+06	4.70E+06	3.83E+06	3.40E+06	5.32E+06	5.71E+06	4.47E+06	22.04	22.17	22.02	22.17	22.07	22	22.32	22.22	21.46	22.04	22.07	22.22	0.084739	0.086387	0.471112			+

3042	Q9NVP2	Histone ch	ASF1B	3	21.3			6.03E+06		6.65E+06							22.83		22.86					22.83	22.86							
17	A0A087W1	Phosphoac	PGM3	4	8.4		6.55E+06	5.75E+06		7.28E+06	5.56E+06	5.18E+06				22.82	22.78		22.99	22.68	22.26			22.8	22.83	22.26	0.030454	0.220335				
2231	Q14683	Structural	SMC1A	31	27.9	5.53E+07	4.85E+07	4.35E+07	4.95E+07	5.45E+07	4.32E+07	3.67E+07	2.44E+07	1.78E+07	25.69	25.69	25.59	25.72	25.85	25.62	25.14	24.36	23.69	25.69	25.72	24.36	0.056425	0.115901	0.725167			
3237	Q9Y4W6	AFG3-like p	AFG3L2	13	19.3	1.56E+07	1.33E+07	1.21E+07	1.49E+07	1.25E+07	1.32E+07	1.83E+07	1.56E+07	1.49E+07	23.84	23.84	23.78	23.87	23.72	23.87	24.1	23.7	23.44	23.84	23.87	23.7	0.03829	0.084464	0.333692			
2991	Q9NP72	Ras-relatec	RAB18	3	16	4.09E+06	3.67E+06	2.44E+06	3.51E+06	4.11E+06	3.29E+06		3.40E+06		21.92	22.07	21.52	21.73	22.14	21.95		21.52		21.92	21.95	21.52	0.282941	0.204257				
2391	Q5T7U1	General tra	GTF3C5	7	18.4	6.31E+06	4.49E+06	5.63E+06	5.41E+06	5.30E+06	7.00E+06	5.41E+06	3.46E+06	2.89E+06	22.52	22.35	22.77	22.37	22.55	23	22.34	21.54	20.9	22.52	22.55	21.54	0.210285	0.328666	0.724804			
1794	P46783	40S riboso	RPS10	6	34.5	2.69E+07	6.90E+07	1.02E+08	3.17E+07	7.41E+07	7.81E+07	2.70E+07	2.71E+07		24.62	26.3	26.98	25	26.33	26.49	24.68	24.5		26.3	26.33	24.59	1.217048	0.814724	0.127357			
2222	Q14432	cGMP-inhil	PDE3A	9	10.4	1.07E+07	1.27E+07	9.14E+06	1.13E+07	1.76E+07	8.98E+06	1.66E+07	1.30E+07	1.39E+07	23.33	23.79	23.39	23.42	24.23	23.34	23.95	23.42	23.33	23.39	23.42	23.42	0.251429	0.496072	0.334516		+	
2298	Q15717	ELAV-like p	ELAVL1	9	33.7	2.15E+07	2.16E+07	2.02E+07	2.26E+07	1.51E+07	2.04E+07	1.32E+07	1.62E+07	1.49E+07	24.31	24.53	24.48	24.51	24	24.51	23.57	23.75	23.44	24.48	24.51	23.57	0.117137	0.294858	0.155082			
983	I3L2C7	Gem-assoc	GEMIN4	23	24.1	5.30E+07	7.55E+07	7.44E+07	8.33E+07	7.83E+07	7.66E+07	7.89E+07	9.76E+07	1.07E+07	25.63	26.42	26.52	26.46	26.39	26.45	26.18	26.32	22.92	26.42	26.45	26.18	0.489855	0.033961	1.919935			
1259	O75368	SH3 domai	SH3BGRL	3	37.7			3.30E+06			3.36E+06						21.96			21.98				21.96	21.98							
1995	P62753	40S riboso	RPS6	9	34.1	8.37E+07	9.15E+07	6.94E+07	5.60E+07	8.59E+07	7.48E+07	4.81E+07	5.77E+07	3.40E+07	26.34	26.7	26.4	25.84	26.56	26.42	25.49	25.6	24.58	26.4	26.42	25.49	0.194075	0.380004	0.557517			
3166	Q9UNF1	Melanoma	MAGED2	16	29	3.87E+07	3.77E+07	3.69E+07	3.40E+07	4.66E+07	3.63E+07	4.18E+07	3.55E+07	1.63E+07	25.18	25.36	25.39	25.14	25.65	25.39	25.31	24.89	23.59	25.36	25.39	24.89	0.116557	0.254232	0.893684			
1921	P55081	Microfibrill	MFAP1	2	6.2	2.24E+06	1.55E+06	1.76E+06			1.77E+06	1.84E+06	1.74E+06		21.01	20.88	21.1			21.03	20.71	20.53		21.01	21.03	20.62	0.113679		0.12866			
2245	Q14C86-4	GTPase-act	GAPVD1	14	13.6	8.96E+06	1.14E+07	9.99E+06	1.22E+07	1.20E+07	9.99E+06	9.68E+06	1.28E+07	1.85E+07	23.06	23.63	23.53	23.55	23.66	23.48	23.15	23.4	23.74	23.53	23.55	23.4	0.304224	0.091118	0.293883			
53	F5GYR3	Probable 2	NOP2	2	6.5			7.29E+05	6.67E+05	6.03E+05	6.24E+05		6.61E+05				19.71	19.61	19.73	19.99		19.33		19.71	19.73	19.33		0.198063				
1124	O00767	Acyl-CoA d	SCD	3	11.1	1.14E+07	1.18E+07	9.28E+06	1.82E+07	9.96E+06	9.62E+06		9.78E+06	1.27E+07	23.42	23.69	23.42	24.18	23.41	23.45		23	23.18	23.42	23.45	23.09	0.156543	0.431846	0.125784			
406	H7C2W9	60S riboso	RPL31	6	44.4	6.49E+07	8.99E+07	6.75E+07		9.63E+07	5.72E+07	1.32E+07	7.17E+07		25.9	26.67	26.35		26.77	25.97	23.57	25.88		26.35	26.37	24.73	0.390984	0.56526	1.632057			
2921	Q9H1I8-3	Activating	ASCC2	5	7.3	3.97E+06	5.51E+06	5.43E+06	6.40E+06	5.64E+06	3.91E+06	4.64E+06	5.29E+06		21.88	22.6	22.71	22.65	22.62	22.18	22.11	22.08		22.6	22.62	22.1	0.448885	0.25952	0.015573			
2196	Q13867	Bleomycin	BLMH	3	8.6	7.09E+06	8.21E+06	7.42E+06	9.10E+06	6.96E+06	7.96E+06		8.51E+06	1.45E+07	22.69	23.15	23.11	23.13	22.94	23.19		22.8	23.39	23.11	23.13	23.1	0.25417	0.130605	0.41791	+		
3011	Q9NRF8	CTP syntha	CTPS2	8	16.4	9.79E+06	8.07E+06	7.68E+06	9.42E+06	9.40E+06	5.85E+06	1.02E+07	1.09E+07	1.01E+07	23.19	23.12	23.16	23.18	23.32	22.75	23.22	23.14	22.81	23.16	23.18	23.14	0.034079	0.295428	0.217258			
1548	P18031	Tyrosine-p	PTPN1	5	13.1	1.08E+07	8.64E+06	1.30E+07	1.02E+07	9.71E+06	1.01E+07	1.32E+07	1.15E+07		23.34	23.21	23.89	23.29	23.36	23.5	23.57	23.22		23.34	23.36	23.4	0.357051	0.104601	0.248981			+
104	A0A087WZ	Beta-parvi	PARVB	6	22.8	6.72E+06	4.93E+06	3.53E+06	7.36E+06	5.11E+06	3.65E+06	5.96E+06	8.26E+06	8.67E+06	22.6	22.47	22.06	22.85	22.49	22.1	22.48	22.76	22.57	22.47	22.49	22.57	0.286249	0.373079	0.144406			+
1167	O15269	Serine palr	SPTLC1	6	13.7	7.17E+06	1.28E+07	1.09E+07	1.46E+07	1.00E+07	1.17E+07	1.59E+07	1.37E+07	1.71E+07	22.71	23.8	23.64	23.83	23.42	23.66	23.87	23.49	23.65	23.64	23.66	23.65	0.591501	0.208216	0.192259	+		
2700	Q96A65	Exocyst co	EXOC4	15	20.6	1.22E+07	7.48E+06	1.20E+07	8.95E+06	1.10E+07	1.06E+07	1.68E+07	9.17E+06	8.33E+06	23.51	23.02	23.77	23.1	23.53	23.55	23.97	22.9	22.51	23.51	23.53	22.9	0.377834	0.256475	0.753105			
1100	O00203-3	AP-3 comp	AP3B1	26	26.2	4.52E+07	5.26E+07	4.05E+07	4.17E+07	4.42E+07	4.25E+07	4.81E+07	4.04E+07	2.48E+07	25.42	25.87	25.53	25.42	25.55	25.59	25.49	25.07	24.11	25.53	25.55	25.07	0.231226	0.085667	0.708217			
733	E9PQP1	NADH deh	NDUFV1	2	10.1	1.80E+06	1.91E+06	1.77E+06		1.68E+06	2.09E+06		1.74E+06		20.68	21.17	21.11		20.94	21.32		20.53		21.11	21.13	20.53	0.264844	0.264573				
2722	Q96DG6	Carboxyme	CMBL	11	42	1.33E+08	7.65E+07	7.36E+07	8.62E+07	8.34E+07	1.08E+08	9.17E+07	1.27E+08	1.88E+08	27.01	26.44	26.5	26.52	26.51	27.02	26.46	26.71	26.77	26.5	26.52	26.71	0.313968	0.291784	0.165928			
90	A0A087WY	Stomatin-li	STOML2	3	13.9	1.95E+06	1.39E+06	1.72E+06		1.66E+06	1.37E+06		2.06E+06		20.81	20.68	21.07		20.93	20.72		20.83		20.81	20.83	20.83	0.199238	0.153255			+	
361	B1ANR0	Polyadenyl	PABPC4	14	39.8	5.63E+07	6.34E+07	7.23E+07	7.12E+07	6.68E+07	8.06E+07	7.16E+07	6.36E+07	2.73E+07	25.73	26.17	26.47	26.16	26.18	26.55	26.02	25.74	24.23	26.17	26.18	25.74	0.373765	0.21695	0.962143			
682	E9PDP5	Ankyrin re	ANKHD1	3	6.8	4.98E+05	5.82E+05	7.91E+05	4.91E+05	5.63E+05	5.60E+05				19.33	19.58	19.94	19.33	19.6	19.88				19.58	19.6		0.305109	0.273088				
1191	O43324	Eukaryotic	EEF1E1	10	63.2	5.77E+08	2.98E+08	3.17E+08	5.03E+08	3.75E+08	4.19E+08	2.02E+08	3.82E+08	3.45E+08	29.15	28.61	28.85	28.86	28.58	28.96	27.62	28.06	27.52	28.85	28.86	27.62	0.271749	0.195112	0.289231			
1152	O15031	Plexin-B2	PLXNB2	8	6.2	4.71E+06	3.16E+06	4.10E+06	3.82E+06	3.90E+06	3.93E+06				22.09	21.85	22.28	21.87	22.1	22.19				22.09	22.1		0.217556	0.16853				
810	F8W0Q9	Periphilin-	PPHLN1	9	24.2	6.08E+07	4.20E+07	3.55E+07	5.49E+07	4.11E+07	4.11E+07	5.24E+07	5.15E+07	2.64E+07	25.8	25.55	25.31	25.82	25.44	25.56	25.6	25.43	24.18	25.55	25.56	25.43	0.243174	0.194236	0.777274			
1625	P26583	High mobil	HMGB2	3	23		1.50E+06	4.68E+06			2.65E+06					20.82	22.46			21.65				21.64	21.65		1.164858					
2720	Q96CW5	Gamma-tu	TUBGCP3	24	30.8	3.84E+07	5.92E+07	3.67E+07	4.13E+07	3.81E+07	3.60E+07	6.95E+07	3.82E+07	4.55E+07	25.16	26.06	25.37	25.41	25.35	25.38	25.96	25	24.96	25.37	25.38	25	0.470127	0.030945	0.566101			
2572	Q8N9N2-2	Activating	ASCC1	6	15.4	5.09E+06	7.87E+06	6.75E+06	6.38E+06	7.07E+06	6.85E+06		5.37E+06		22.2	23.09	22.94	22.64	22.95	22.96		22.11		22.94	22.95	22.11	0.478111	0.183607				
2949	Q9H5Q4	Dimethylac	TFB2M	14	35.9	2.17E+07	2.16E+07	1.86E+07	2.12E+07	1.63E+07	1.81E+07	2.84E+07	3.06E+07	2.26E+07	24.32	24.53	24.37	24.41	24.12	24.38	24.77	24.73	24	24.37	24.38	24.73	0.111591	0.16032	0.433514			
3088	Q9P287-4	BRCA2 and	BCCIP	3	16.1	6.00E+06	1.08E+07	1.04E+07	6.68E+06	1.44E+07	1.07E+07	1.00E+07	8.36E+06	7.17E+06	22.44	23.55	23.57	22.7	23.93	23.56	23.2	22.78	22.27	23.55	23.56	22.78	0.646676	0.627952	0.464596			
386	Q8NBY1	Serine/thre	STK26	11	30.4	2.57E+07	2.55E+07	2.46E+07	3.21E+07	2.56E+07	2.54E+07	2.82E+07	2.87E+07	2.21E+07	24.56	24.78	24.79	25.03	24.79	24.76	24.75	24.6	23.98	24.78	24.79	24.6	0.129256	0.146361	0.403929			
2696	Q969Z0	Protein TB	TBRG4	4	8.2	2.31E+06	2.86E+06	4.20E+06	2.33E+06	2.85E+06	2.96E+06	2.10E+06			21.09	21.68	22.32	21.18	21.69	21.81	20.84			21.68	21.69	20.84	0.6165	0.331909				
2667	Q92621	Nuclear po	NUP205	31	17.3	3.40E+07	3.61E+07	3.35E+07	3.14E+07	4.02E+07	3.19E+07	2.00E+07	2.34E+07	1.28E+07	25	25.29	25.18	24.95	25.41	25.18	24.24	24.3	23.19	25.18	25.18	24.24	0.145356	0.226548	0.622422			
1475	P10909-4	Clusterin	CLU	10	29.8	4.21E+07	5.58E+07	5.10E+07	5.89E+07	4.99E+07	5.81E+07	5.28E+07	7.26E+07	3.40E+07	25.32	25.98	25.91	25.92	25.75	26.01	25.62	25.91	24.58	25.91	25.92	25.62	0.358566	0.127248	0.697568			
1882	P51991-2	Heterogen	HNRNPA3	5	22.2	5.58E+06	4.69E+06	6.00E+06	5.55E+06	4.43E+06	6.29E+06	4.42E+06	4.15E+06		22.32	22.4	22.82	22.41	22.28	22.84	22.04	21.79		22.4	22.41	21.91	0.270736	0.296768	0.175866			
3076	Q9P0K7-4	Ankycorbir	RAI14	5	6.4	2.34E+06	1.82E+06	2.47E+06	2.09E+06	1.95E+06	1.85E+06	2.34E+06	1.76E+06		21.1	21.1	21.54	21	21.11	21.14	21.08	20.56		21.1	21.11	20.82	0.252211	0.074251	0.368713			
2718	Q96CS3	FAS-associ	FAF2	7	21.6	9.48E+06	1.18E+07	1.09E+07	1.60E+07	1.18E+07	1.16E+07	1.52E+07	1.32E+07	1.00E+07	23.14	23.69	23.64	23.99	23.63	23.65	23.83	23.44	22.8	23.64	23.65	23.44	0.303432	0.20005	0.524211			
2288	Q15477	Helicase SK	SKIV2L	12	12.1	7.01E+06	6.15E+06	5.46E+06	6.51E+06	6.42E+06	5.76E+06	9.33E+06	7.40E+06	7.37E+06	22.68	22.74	22.72	22.66	22.81	22.73	23.1	22.58	22.32	22.72	22.73	22.58	0.031495	0.074126	0.394772			
1214	O60264	SWI/SNF-re	SMARCA5	5	5.2	1.17E+06	1.20E+06	1.36E+06	1.32E+06	1.27E+06	1.24E+06				20.11	20.52	20.72	20.36	20.52	20.62				20.52	20.52		0.313882	0.135006				
1769	P42224	Signal tran	STAT1	16	23.2	1.59E+07	1.56E+07	1.50E+07	1.25E+07	1.72E+07	1.50E+07	1.71E+07	1.74E+07	1.57E+07	23.87	24.06	24.06	23.58	24.21	24.06	24.01	23.85	23.53	24.06	24.06	23.85	0.104882	0.329029	0.245711			
73	A0A087WV	THO comp	THOC1	3	5.6		1.73E+06	1.94E+06	2.25E+06	1.57E+06	2.43E+06	1.93E+06				21	21.24	21.12	20.8	21.51	20.78			21.12	21.12	20.78	0.166512	0.354625				
1741	P38646	Stress-70 p	HSPA9	32	50.2	1.02E+09	7.95E+08	8.30E+08	1.12E+09	1.05E+09	9.12E+08	1.04E+09	1.01E+09	8.82E+08	30.24	30.13	30.32	30.31	30.24	30.04	29.95	29.86	29.05	30.24	30.24	29.86	0.090468	0.139795	0.491767			
1409	P06576	ATP syntha	ATP5B	24	66	2.03E+08	1.51E+08	2.96E+08	1.78E+08	2.02E+08	2.42E+08	2.03E+08	6.85E+07	1.05E+08	27.73	27.54	28.77	27.49	27.73	28.31	27.64	25.83	26.09	27.73	27.73	26.09	0.660923	0.421375	0.983081			
3071	Q9NZM1-6	Myoferlin	MYOF	60	34.1	8.28E+07	1.02E+08	7.95E+07	7.53E+07	9.32E+07	8.52E+07	1.15E+08	1.18E+08	4.70E+07	26.32	26.84	26.6	26.28	26.7	26.6	26.79	26.62	25.01	26.6	26.6	26.62	0.26233	0.220211	0.980875			
1330	O95602	DNA-direct	POLR1A	6	5.3	3.11E+06	2.59E+06	2.67E+06	2.63E+06	2.63E+06	2.69E+06				21.54	21.56	21.64	21.38	21.56	21.67				21.56	21.56		0.053724	0.145501				
471	C9JXB8	60S riboso	RPL24	6	41.3	5.31E+07	3.20E+07	1.98E+07	3.28E+07	4.13E+07	2.69E+07	2.53E+07	2.75E+07	4.56E+07	25.63	25.08	24.45	25.08	25.45	24.89	24.6	24.52	24.97	25.08	25.08	24.6	0.592885	0.282671	0.239549			
3263	Q9Y6G9	Cytoplasmi	DYNC1LI1	8	26	1.41E+07	1.13E+07	1.11E+07	1.32E+07	1.13E+07	1.34E+07	9.96E+06	1.08E+07		23.69	23.62	23.68	23.68	23.57	23.89	23.19	23.13		23.68	23.68	23.16	0.036641	0.159567	0.040975			
3212	Q9Y3B4	Splicing fac	SF3B6	4	40.8	1.69E+07	1.68E+07	2.09E+07	1.49E+07	2.06E+07	1.62E+07		1.11E+07		23.97	24.18	24.55	23.87	24.47	24.18		23.17		24.18	24.18	23.17	0.293106	0.301253				
433	C9J0K6	Sorcin	SRI	3	18.7			3.67E+06	3.73E+06	4.11E+06	4.13E+06	3.61E+06		5.03E+06			22.14	21.84	22.14	22.27	21.72		21.7	22.14	22.14	21.71		0.220612	0.01627			
3143	Q9UJX3-2	Anaphase-	ANAPC7	6	15.1	6.08E+06	4.70E+06	5.18E+06	5.66E+06	4.96E+06	5.13E+06	5.36E+06	6.06E+06		22.46	22.41	22.61	22.46	22.44	22.56	22.32	22.3		22.46	22.46	22.31	0.101874	0.064195	0.017259			
440	C9J3G2	COBW don	CBWD2	5	17.8	5.61E+06	4.36E+06	3.49E+06	5.14E+06	5.22E+06	4.21E+06	6.51E+06	7.02E+06		22.33	22.31	22.02	22.3	22.53	22.28	22.61	22.51		22.31	22.3	22.56	0.174373	0.13664	0.07151			
13	E9PKG6	Nucleobin	NUCB2	2	8.9		3.56E+06	2.83E+06			3.13E+06					22.03	21.74			21.88				21.89	21.88		0.20734					
2919	Q9H1A4	Anaphase-	ANAPC1	9	6	4.80E+06	4.19E+06	4.20E+06	4.08E+06	4.53E+06	4.14E+06	5.59E+06	3.89E+06		22.12	22.28	22.32	21.97	22.32	22.27	22.39	21.71		22.28	22.27	22.05	0.10582	0.190376	0.480599			
2971	Q9H9B4	Sideroflexi	SFXN1	5	19.6	7.88E+06	8.47E+06	7.81E+06	1.06E+07	6.08E+06	7.86E+06	4.49E+06	5.34E+06		22.83	23.19	23.18	23.34	22.73	23.17	22.07	22.1		23.18	23.17	22.08	0.201096	0.313964	0.019753			
661	E7EVA0	Microtubu	MAP4	12	7.6	1.60E+07	1.56E+07	1.65E+07	1.53E+07	1.56E+07	1.54E+07	1.49E+07	1.16E+07		23.89	24.06	24.2	23.89	24.05	24.11	23.79	23.24		24.06	24.05	23.52	0.156722	0.110589	0.392236			
1740	P38606-2	V-type pro	ATP6V1A	11	25.7	1.56E+07	2.67E+07	3.50E+07	1.49E+07	2.81E+07	2.62E+07	2.01E+07	3.05E+07	6.86E+06	23.84	24.84	25.28	23.87	24.92	24.83	24.26	24.72	22.2	24.84	24.83	24.26	0.73717	0.582044	1.340333			
1662	P30153	Serine/thre	PPP2R1A	18	41.6	6.54E+07	1.00E+08	1.34E+08	7.51E+07	9.83E+07	1.08E+08	8.92E+07	8.63E+07	4.88E+07	25.9	26.81	27.41	26.27	26.8	27.02	26.39	26.17	25.06	26.81	26.8	26.17	0.758287	0.38351	0.714921			
3126	Q9UHD2	Serine/thre	TBK1	12	21.9	1.44E+07	1.07E+07	1.03E+07	1.22E+07	1.13E+07	1.03E+07	1.23E+07	1.15E+07	1.10E+07	23.72	23.53	23.56	23.55	23.57	23.52	23.46	23.22	22.97	23.56	23.55	23.22	0.105193	0.026174	0.243501			
912	H0YME5	Eukaryotic	EIF2AK4	8	7.1	2.44E+06	3.01E+06	3.26E+06	2.99E+06	3.04E+06	3.55E+06		3.76E+06		21.18	21.76	21.94	21.53	21.75	22.05		21.65		21.76	21.75	21.65	0.400025	0.258578				
2585	Q8NCW5-2	NAD(P)H-h	APOA1BP	2	16.2	2.33E+06	3.27E+06	3.35E+06			3.14E+06				21.1	21.9	21.97			21.89				21.9	21.89		0.486035					
2208	Q14145	Kelch-like E	KEAP1	6	10.1	9.40E+06	4.27E+06	4.89E+06	4.69E+06	5.18E+06	6.17E+06	4.47E+06			23.13	22.29	22.53	22.17	22.52	22.82	22.06			22.53	22.52	22.06	0.431552	0.326292				
1377	P04040	Catalase	CAT	5	14.6	3.29E+06		2.92E+06	3.37E+06	2.83E+06	2.78E+06				21.62		21.78	21.69	21.68	21.73				21.7	21.69		0.114165	0.025024				
2775	Q96QR8	Transcripti	PURB	2	12.5		3.33E+06			3.11E+06	3.60E+06	1.68E+06				21.94			21.78	22.07	20.6			21.94	21.93	20.6		0.20071				
1785	P43686	26S protea	PSMC4	22	57.2	1.17E+08	1.79E+08	1.42E+08	1.09E+08	1.68E+08	1.55E+08	1.51E+08	2.11E+08	8.68E+07	26.9	27.85	27.48	26.8	27.47	27.66	27.18	27.42	25.89	27.48	27.47	27.18	0.48006	0.45131	0.823138			
857	G5EA06	28S riboso	MRPS27	7	21.5	8.55E+06	1.29E+07	1.05E+07	8.90E+06	1.13E+07	1.23E+07	1.04E+07	1.12E+07	9.81E+06	22.98	23.82	23.58	23.08	23.57	23.75	23.24	23.19	22.75	23.58	23.57	23.19	0.429946	0.346669	0.271481			
2726	Q96EK5	KIF1-bindir	KIAA1279	4	10	4.34E+06	4.51E+06	4.69E+06			4.41E+06				22	22.36	22.47			22.34				22.36	22.34		0.244796					
2178	Q13464	Rho-associ	ROCK1	48	39.2	1.64E+08	1.16E+08	1.59E+08	1.60E+08	1.42E+08	1.46E+08	1.71E+08	1.59E+08	6.49E+07	27.38	27.09	27.59	27.36	27.22	27.57	27.33	27.02	25.41	27.38	27.36	27.02	0.250639	0.17703	1.033567			
212	A0A0C4DF	Lanosterol	CYP51A1	4	7.5	2.12E+06	1.83E+06	3.03E+06	2.21E+06	1.92E+06	2.29E+06	2.57E+06			20.95	21.11	21.84	21.09	21.09	21.45	21.21			21.11	21.09	21.21	0.471949	0.207248				

331	A8MQB8	Fragile X m	FMR1	7	13.9	1.35E+07	1.01E+07	9.59E+06	1.16E+07	1.06E+07	9.71E+06	1.44E+07	1.02E+07	9.37E+06	23.63	23.45	23.48	23.46	23.5	23.45	23.74	23.05	22.68	23.48	23.46	23.05	0.097704	0.023812	0.536252
819	F8W7U8	Double-str	MRE11A	13	24.2	4.13E+07	3.76E+07	3.86E+07	3.95E+07	3.51E+07	3.89E+07	4.10E+07	4.20E+07	3.09E+07	25.28	25.36	25.46	25.34	25.23	25.47	25.27	25.11	24.41	25.36	25.34	25.11	0.093493	0.122524	0.457049
2965	Q9H871	Protein RM	RMND5A	7	22.3	1.11E+07	9.43E+06	6.07E+06	1.05E+07	1.14E+07	6.88E+06	1.02E+07	9.12E+06	6.16E+06	23.39	23.35	22.83	23.33	23.59	22.97	23.22	22.89	22.04	23.35	23.33	22.89	0.310806	0.311423	0.608162
1303	O94906-2	Pre-mRNA	PRPF6	15	19.3	1.27E+07	1.35E+07	1.23E+07	1.41E+07	1.15E+07	1.39E+07	1.46E+07	5.43E+06		23.57	23.86	23.79	23.78	23.6	23.95	23.77	22.13		23.79	23.78	22.95	0.152495	0.173874	1.164206
2912	Q9H0C8	Integrin-lin	ILKAP	3	7.7	2.27E+06	3.20E+06	2.97E+06	3.64E+06	3.12E+06	3.45E+06	3.92E+06			21.06	21.87	21.81	21.78	21.79	22.02	21.85			21.81	21.79	21.85	0.452351	0.136354	
3133	Q9UHX1-6	Poly(U)-bir	PUF60	16	46.6	8.80E+07	6.36E+07	6.50E+07	7.84E+07	5.56E+07	6.69E+07	6.39E+07	7.68E+07	4.01E+07	26.47	26.18	26.26	26.34	25.9	26.24	25.86	26	24.8	26.26	26.24	25.86	0.147157	0.2298	0.658803
2062	Q00839	Heterogen	HNRNPU	21	30.5	4.59E+08	2.00E+08	2.82E+08	3.90E+08	3.97E+08	3.77E+08	1.06E+08	1.16E+08	6.85E+07	28.84	28.01	28.7	28.5	28.68	28.83	26.7	26.57	25.49	28.7	28.68	26.57	0.4468	0.160578	0.664011
1985	P62316	Small nucle	SNRPD2	15	71.2	3.39E+09	2.72E+09	2.50E+09	4.25E+09	6.68E+09	4.18E+09	1.14E+10	1.49E+10	2.03E+10	32.36	32.37	32.43	31.96	32.87	32.35	33.91	33.45	32.73	32.37	32.35	33.45	0.035076	0.456055	0.596607
151	U3KQC1	WD repeat	WDR18	5	11.2	2.19E+06	5.53E+06	3.53E+06	3.69E+06	3.77E+06	4.17E+06		3.77E+06		20.99	22.6	22.06	21.82	22.04	22.28		21.66		22.06	22.04	21.66	0.820269	0.230243	
2300	Q15750	TGF-beta-a	TAB1	26	76.2	7.44E+08	5.27E+08	5.64E+08	6.84E+08	7.28E+08	6.24E+08	7.04E+08	7.54E+08	5.34E+08	29.52	29.29	29.66	29.3	29.55	29.49	29.23	29.15	28.45	29.52	29.49	29.15	0.187283	0.134369	0.431245
2225	Q14566	DNA replic	MCM6	24	34	3.98E+07	5.50E+07	3.72E+07	3.32E+07	3.98E+07	4.28E+07	2.38E+07	9.66E+06	5.94E+06	25.23	25.97	25.41	25.09	25.39	25.6	24.49	22.98	21.94	25.41	25.39	22.98	0.382688	0.257682	1.279523
348	H3BPC4	SUMO-con	UBE2I	4	51.4	1.08E+07	1.36E+07	1.50E+07	1.11E+07	1.36E+07	1.79E+07	9.48E+06	5.94E+06	7.73E+06	23.34	23.87	24.06	23.39	23.85	24.36	23.13	22.27	22.41	23.87	23.85	22.41	0.371191	0.483026	0.459426
3053	Q9NXS2	Glutaminyl	QPCTL	17	42.4	9.03E+08	6.26E+08	4.35E+08	7.75E+08	7.83E+08	4.17E+08	9.02E+08	1.06E+09	7.78E+08	29.91	29.53	29.25	29.51	29.74	28.94	29.65	29.89	28.88	29.53	29.51	29.65	0.330109	0.412163	0.529293
2035	P78417	Glutathion	GSTO1	14	39	1.72E+08	1.79E+08	1.92E+08	2.31E+08	2.10E+08	2.23E+08	1.27E+08	1.45E+08	8.78E+07	27.47	27.85	28	27.83	27.78	28.11	26.95	26.88	25.92	27.85	27.83	26.88	0.272637	0.181023	0.57754
1655	P30048-2	Thioredoxi	PRDX3	9	39.9	3.65E+07	5.22E+07	4.52E+07	3.08E+07	5.47E+07	4.41E+07	5.62E+07	3.86E+07	2.32E+07	25.09	25.86	25.68	24.92	25.87	25.66	25.71	25.01	24.02	25.68	25.66	25.01	0.402241	0.499412	0.850449
1871	P51452	Dual specif	DUSP3	4	29.7	3.20E+06	3.74E+06	4.96E+06	3.94E+06		4.19E+06	4.13E+06		3.62E+06	21.58	22.12	22.55	21.91		22.28	21.95		21.16	22.12	22.1	21.55	0.487473	0.260799	0.559621
789	F8VPD4	CAD protei	CAD	51	30.4	1.91E+08	2.16E+08	1.86E+08	2.00E+08	2.26E+08	1.91E+08	2.18E+08	2.10E+08	2.03E+08	27.64	28.13	27.95	27.66	27.93	27.98	27.76	27.4	26.85	27.95	27.93	27.4	0.244222	0.171746	0.460273
2523	Q86YP4-2	Transcripti	GATAD2A	3	6.1	1.92E+06	1.13E+06		1.54E+06	1.30E+06	1.92E+06	1.80E+06	1.50E+06	1.66E+06	20.79	20.44		20.51	20.59	21.2	20.69	20.27	19.87	20.62	20.59	20.27	0.250944	0.378697	0.40966
1519	P14174	Macrophag	MIF	2	17.4	1.00E+07	2.12E+07	2.04E+07			1.95E+07	8.31E+06	1.26E+07		23.24	24.48	24.51			24.46	22.92	23.38		24.48	24.46	23.15	0.726099		0.327083
1315	O95239	Chromosor	KIF4A	14	13	7.27E+06	6.67E+06	7.63E+06	6.86E+06	6.46E+06	6.81E+06	8.36E+06	6.45E+06		22.72	22.85	23.15	22.74	22.82	22.95	22.93	22.38		22.85	22.82	22.65	0.220804	0.102412	0.389957
954	H7C0R7	NADH-cytc	CYB5R1	2	8.8		8.84E+05	1.16E+06			8.49E+05					19.99	20.53			20.23				20.26	20.23		0.377938		
2589	Q8NDI1-3	EH domain	EHBP1	41	39.5	3.53E+08	3.78E+08	2.27E+08	4.02E+08	2.86E+08	2.85E+08	6.58E+08	4.27E+08	2.82E+08	28.51	28.95	28.33	28.58	28.28	28.49	29.15	28.23	27.23	28.51	28.49	28.23	0.321354	0.150699	0.957894
1793	P46778	60S riboso	RPL21	4	31.9	1.58E+07	1.99E+07	1.24E+07	9.83E+06	2.65E+07	1.29E+07	1.34E+07	7.21E+06	5.52E+06	23.86	24.39	23.81	23.25	24.83	23.84	23.59	22.55	21.84	23.86	23.84	22.55	0.320673	0.798804	0.881025
2632	Q8WUM0	Nuclear po	NUP133	33	36.2	7.72E+07	7.40E+07	6.62E+07	5.66E+07	8.33E+07	6.74E+07	4.44E+07	7.88E+07	1.86E+07	26.23	26.39	26.28	25.86	26.5	26.25	25.36	26.05	23.74	26.28	26.25	25.36	0.081616	0.321295	1.182577
2027	P68371	Tubulin be	TUBB4B	29	67	3.15E+09	3.13E+09	3.42E+09	5.21E+09	5.59E+09	4.34E+09	6.10E+09	4.71E+09	5.77E+09	32.12	32.48	32.77	32.24	32.46	32.59	32.1	31.69	31.07	32.48	32.46	31.69	0.327816	0.175865	0.521717
1632	X1WI28	60S riboso	RPL10	7	39.8	1.20E+07	1.20E+07	1.09E+07	1.34E+07		1.04E+07				23.49	23.72	23.64	23.7		23.53				23.64	23.62		0.118732	0.119043	
1490	P11498	Pyruvate c	PC	26	29.9	3.18E+07	3.62E+07	3.35E+07	3.26E+07	3.31E+07	3.39E+07	2.82E+07	3.63E+07	1.32E+07	24.94	25.3	25.18	25.06	25.15	25.28	24.75	24.92	23.26	25.18	25.15	24.75	0.184374	0.110973	0.915568
2989	Q9HD20-2	Manganes	ATP13A1	8	8.2	2.67E+06	2.75E+06	2.91E+06	2.27E+06	2.78E+06	2.54E+06	2.90E+06			21.31	21.62	21.77	21.13	21.64	21.59	21.41			21.62	21.59	21.41	0.233178	0.279931	
1086	M0R208	ATP-deper	CLPP	2	14.2	1.45E+06	1.92E+06	1.11E+06		1.20E+06					20.39	21.19	20.45		20.42					20.45	20.42		0.444265		
216	A0A0C4DF	Protein UX	UXT	2	14.2		3.71E+06	4.50E+06	3.67E+06	4.32E+06	4.32E+06	4.05E+06	3.74E+06			22.1	22.41	21.8	22.23	22.32	21.91	21.64		22.26	22.23	21.78	0.222431	0.276389	0.190911
1327	O95456-2	Proteasom	PSMG1	2	9	1.91E+07	1.67E+07	1.55E+07	1.64E+07	1.90E+07	1.58E+07	1.88E+07	1.81E+07	1.55E+07	24.16	24.16	24.11	24.03	24.34	24.13	24.15	23.91	23.51	24.16	24.13	23.91	0.029832	0.158261	0.324889
3192	Q9Y2H1	Serine/thre	STK38L	43	73.9	5.77E+09	3.78E+09	4.02E+09	7.80E+09	7.29E+09	6.30E+09	7.06E+09	9.79E+09	2.58E+10	33.11	32.73	33.07	33.39	32.98	33.04	32.78	32.78	33.19	33.07	33.04	32.78	0.208851	0.224473	0.237794
2659	Q92542-2	Nicastrin	NCSTN	4	5.8	2.72E+06	2.08E+06	4.15E+06	2.41E+06	2.23E+06	2.42E+06				21.33	21.3	22.3	21.24	21.3	21.5				21.33	21.3		0.569431	0.136043	
2598	Q8NFQ8	Torsin-1A-i	TOR1AIP2	2	6.6		2.36E+06	2.73E+06			2.44E+06					21.43	21.67			21.52				21.55	21.52		0.169534		
1693	P32321	Deoxycytic	DCTD	2	14.6		1.41E+06	1.24E+06		1.36E+06						20.71	20.62		20.63					20.66	20.63		0.066398		
1180	O43143	Pre-mRNA	DHX15	29	38.2	1.39E+08	1.13E+08	1.29E+08	1.20E+08	1.24E+08	1.25E+08	8.47E+07	9.01E+07	4.01E+07	27.12	27.02	27.32	26.95	27.08	27.26	26.32	26.25	24.8	27.12	27.08	26.25	0.152083	0.157089	0.859615
2006	P62913	60S riboso	RPL11	5	28.1	1.20E+08	1.03E+08	7.91E+07	1.19E+08	9.50E+07	9.82E+07	1.07E+08	1.13E+08		26.91	26.86	26.58	26.93	26.76	26.82	26.71	26.5		26.86	26.82	26.6	0.174102	0.086542	0.152757
3038	Q9NVI1	Fanconi an	FANCI	13	12.6	8.49E+06	7.80E+06	7.95E+06	5.81E+06	7.58E+06	7.22E+06	9.19E+06	9.39E+06	7.11E+06	22.97	23.07	23.21	22.48	23.04	23.04	23.08	22.93	22.25	23.07	23.04	22.93	0.116997	0.322544	0.439375
2859	Q9BV44	THUMP do	THUMPD3	5	10.7	3.43E+06	3.67E+06	3.26E+06	4.05E+06	3.08E+06	3.18E+06	5.54E+06	6.07E+06	7.29E+06	21.67	22.07	21.94	21.95	21.77	21.91	22.37	22.3	22.31	21.94	21.91	22.31	0.202565	0.094252	0.039008
307	A6NDU8	UPF0600 p	C5orf51	6	37.4	1.40E+07	1.55E+07	1.42E+07	1.30E+07	1.58E+07	1.39E+07	1.50E+07	2.63E+07	1.31E+07	23.68	24.05	23.98	23.66	24.07	23.95	23.81	24.47	23.24	23.98	23.95	23.81	0.194622	0.211801	0.616527
1943	P60891	Ribose-pho	PRPS1	3	40.9	3.91E+06	5.57E+06	7.80E+06		5.25E+06	5.32E+06	5.41E+06			21.87	22.61	23.17		22.54	22.61	22.34			22.61	22.58	22.34	0.656456	0.05288	
1546	P17931	Galectin-3	LGALS3	9	37.2	2.50E+08	2.63E+08	2.41E+08	3.88E+08	2.67E+08	2.75E+08	2.99E+08	4.02E+08	1.84E+08	27.96	28.42	28.46	28.47	28.15	28.38	28.06	28.15	26.7	28.42	28.38	28.06	0.282223	0.165925	0.810997
590	D6RAA6	Transmem	TMEM33	4	18.9	2.88E+07	1.01E+07	4.75E+06	1.26E+07	9.99E+06	6.61E+06			1.72E+07	24.74	23.45	22.49	23.59	23.42	22.91			23.66	23.45	23.42	23.66	1.128986	0.355561	
836	G3V325	ATP syntha	ATP5J2-PT	4	6.1		1.42E+07	2.12E+07	9.82E+06	2.86E+07	1.66E+07	1.50E+07				23.93	24.58	23.24	24.95	24.22	23.81			24.25	24.22	23.81	0.455555	0.857279	
2380	Q5SRE5	Nucleopor	NUP188	23	15.4	2.27E+07	2.43E+07	2.13E+07	2.37E+07	2.20E+07	2.31E+07	1.55E+07	2.39E+07	9.86E+06	24.38	24.68	24.58	24.54	24.53	24.68	23.84	24.34	22.76	24.58	24.54	23.84	0.154612	0.081086	0.806845
3221	Q9Y3I1-3	F-box only	FBXO7	3	9.8		4.76E+06	2.63E+06	4.33E+06	2.91E+06	3.37E+06		4.28E+06			22.43	21.63	22.06	21.71	21.99		21.83		22.03	21.99	21.83	0.566929	0.184824	
1868	P51149	Ras-relatec	RAB7A	4	23.7	1.33E+07	5.83E+06	3.22E+06	6.31E+06	7.23E+06	4.03E+06	1.42E+07	6.49E+06	1.80E+07	23.61	22.65	21.92	22.61	22.97	22.23	23.72	22.38	23.7	22.65	22.61	23.7	0.848715	0.37016	0.769076
1846	P49841	Glycogen s	GSK3B	4	15	7.89E+06	6.51E+06	5.46E+06	7.04E+06	6.30E+06	5.46E+06	7.80E+06	8.64E+06	7.26E+06	22.84	22.82	22.72	22.78	22.79	22.64	22.84	22.83	22.3	22.82	22.78	22.83	0.064219	0.082834	0.311706
2442	Q6P996-4	Pyridoxal-c	PDXDC1	23	43.8	8.60E+07	8.69E+07	7.92E+07	1.02E+08	8.50E+07	6.09E+07	1.70E+08	1.32E+08	8.51E+07	26.38	26.58	26.59	26.72	26.54	26.08	27.32	26.75	25.83	26.58	26.54	26.75	0.121179	0.331016	0.750859
794	F8VRR3	Methionin	METAP2	5	20.8	4.06E+06	3.01E+06	3.50E+06		2.60E+06	4.01E+06	2.78E+06	1.89E+06		21.91	21.76	22.03		21.52	22.22	21.34	20.7		21.91	21.87	21.02	0.132697	0.489968	0.452846
1244	O60942	mRNA-cap	RNGTT	15	27.1	4.49E+07	4.14E+07	3.98E+07	4.27E+07	3.70E+07	5.04E+07	3.21E+07	3.27E+07	6.78E+06	25.42	25.53	25.5	25.46	25.33	25.8	24.94	24.8	22.18	25.5	25.46	24.8	0.056991	0.242509	1.552406
2520	Q86XI2	Condensin	NCAPG2	10	9.8	5.55E+06	6.08E+06	4.84E+06	4.79E+06	5.03E+06	4.92E+06	5.12E+06	5.47E+06	5.98E+06	22.31	22.71	22.52	22.19	22.47	22.49	22.24	22.15	21.96	22.52	22.47	22.15	0.198923	0.170266	0.145373
3222	Q9Y3P9	Rab GTPas	RABGAP1	6	7.3	2.30E+06	3.13E+06	3.46E+06	2.78E+06	3.47E+06	2.90E+06	3.13E+06	2.87E+06		21.08	21.83	22.01	21.43	21.92	21.78	21.54	21.3		21.83	21.78	21.42	0.49016	0.250417	0.166021
1476	P11021	78 kDa glu	HSPA5	36	54	1.41E+09	1.09E+09	1.10E+09	1.55E+09	1.42E+09	1.22E+09	1.43E+09	1.40E+09	1.50E+09	30.79	30.67	30.81	30.74	30.78	30.6	30.66	30.35	29.68	30.79	30.74	30.35	0.075135	0.097215	0.497262
3096	Q9UBB6-2	Neurochor	NCDN	5	9	3.91E+06	3.95E+06	3.86E+06	3.96E+06	4.18E+06	4.56E+06				21.87	22.21	22.22	21.93	22.17	22.39				22.21	22.17		0.200966	0.231451	
1392	P04899	Guanine nu	GNAI2	7	22	1.24E+07	1.48E+07	1.28E+07	1.45E+07	1.38E+07	1.08E+07	1.31E+07	1.33E+07	1.57E+07	23.54	23.99	23.87	23.82	23.87	23.57	23.56	23.45	23.53	23.87	23.82	23.53	0.233269	0.161053	0.056464
3197	Q9Y2Q9	28S riboso	MRPS28	3	15.5	5.84E+06	5.57E+06	5.82E+06	6.15E+06	5.37E+06	4.46E+06	5.87E+06	6.34E+06	6.24E+06	22.4	22.61	22.8	22.59	22.57	22.36	22.46	22.36	22.07	22.61	22.57	22.36	0.19983	0.12525	0.198971
2998	Q9NPJ8	NTF2-relat	NXT2	3	21.1	7.86E+06	5.71E+06	5.92E+06		3.55E+06	1.09E+07	6.32E+06	7.83E+06		22.83	22.63	22.81		21.96	23.58	22.55	22.68		22.81	22.77	22.62	0.109264	1.14663	0.094696
3102	Q9UBM7	7-dehydro	DHCR7	5	13.3	2.04E+07	1.45E+07	1.43E+07	1.57E+07	1.87E+07	1.22E+07	2.71E+07	2.35E+07		24.24	23.95	23.99	23.94	24.32	23.73	24.68	24.31		23.99	23.94	24.49	0.15476	0.298034	0.265929
3000	Q9NQ88	Fructose-2	TIGAR	2	11.1		2.91E+06				2.69E+06	3.42E+06				21.72				21.67	21.65			21.72	21.67	21.65			
1842	P49756	RNA-bindir	RBM25	7	11.2	8.00E+06	8.72E+06	9.25E+06	7.50E+06	8.51E+06	9.16E+06	2.87E+06	5.26E+06		22.87	23.23	23.41	22.87	23.18	23.38	21.41	22.07		23.23	23.18	21.74	0.2749	0.253483	0.471272
966	H7C3L7	DnaJ homc	DNAJC2	2	11.5		1.03E+06	1.23E+06			9.98E+05	1.06E+06				20.32	20.6			20.41	19.84			20.46	20.41	19.84	0.196865		
1818	P48739	Phosphatic	PITPNB	8	39.9	1.07E+07	1.12E+07	7.39E+06	1.11E+07	7.81E+06	8.57E+06	8.42E+06	7.88E+06	8.78E+06	23.33	23.61	23.1	23.39	23.07	23.27	22.94	22.7	22.58	23.33	23.27	22.7	0.254748	0.163359	0.180195
1780	P43246	DNA mism	MSH2	27	34.8	7.00E+07	7.28E+07	6.17E+07	7.14E+07	6.26E+07	5.92E+07	3.50E+07	5.09E+07	1.17E+07	26.03	26.36	26.15	26.18	26.09	26.04	25.06	25.41	23.07	26.15	26.09	25.06	0.169985	0.069757	1.265046
2636	Q8WUY1	Protein TH	THEM6	3	12.5	2.90E+06	3.17E+06	3.83E+06		3.21E+06	2.92E+06				21.43	21.86	22.21		21.83	21.79				21.86	21.81		0.386788	0.026922	
730	E9PQ57	mRNA exp	RAE1	7	24.5	1.37E+07	1.17E+07	9.16E+06	1.26E+07	1.63E+07	1.03E+07	1.11E+07	1.27E+07	1.32E+07	23.65	23.68	23.4	23.59	24.12	23.52	23.31	23.39	23.26	23.65	23.59	23.31	0.152292	0.326561	0.068748
1173	O15371-2	Eukaryotic	EIF3D	18	46.3	1.33E+08	1.09E+08	1.23E+08	1.08E+08	1.44E+08	1.05E+08	1.65E+08	1.17E+08		27.01	26.99	27.24	26.77	27.27	26.96	27.28	26.59		27.01	26.96	26.93	0.137819	0.249183	0.48683
2746	Q96I25	Splicing fac	RBM17	5	13.5	4.98E+06	3.49E+06	7.60E+06	3.95E+06	4.01E+06	5.93E+06	4.19E+06	3.53E+06		22.18	22.01	23.14	21.92	22.12	22.77	21.97	21.56		22.18	22.12	21.77	0.610232	0.443026	0.290936
2537	Q8IXI1	Mitochond	RHOT2	3	5.2	4.26E+06	2.89E+06	3.51E+06	3.96E+06	3.25E+06	3.77E+06	4.57E+06	4.15E+06		21.98	21.71	22.04	21.93	21.84	22.13	22.09	21.79		21.98	21.93	21.94	0.177284	0.150996	0.215294
883	H0Y7L2	Dedicator	DOCK7	20	11.7	1.97E+07	1.49E+07	1.50E+07	1.62E+07	1.52E+07	8.84E+06	1.83E+07	1.63E+07		24.21	24	24.06	24.01	24	23.31	24.1	23.76		24.06	24	23.93	0.108421	0.398793	0.241882
1981	P62280	40S riboso	RPS11	12	56.3	1.30E+08	1.66E+08	1.64E+08	1.19E+08	1.85E+08	1.89E+08	1.87E+08	1.72E+08	2.99E+08	26.99	27.7	27.65	26.93	27.6	27.96	27.52	27.16	27.33	27.65	27.6	27.33	0.397659	0.522078	0.179804
3118	Q9UGJ1-2	Gamma-tu	TUBGCP4	6	12.3	6.56E+06	6.05E+06	5.84E+06	7.94E+06	5.68E+06	4.91E+06	8.54E+06	8.79E+06	8.03E+06	22.57	22.7	22.81	22.92	22.64	22.49	22.96	22.84	22.45	22.7	22.64	22.84	0.115492	0.220412	0.266333
2518	Q86X55-2	Histone-ar	CARM1	5	13	9.09E+06	5.71E+06	7.68E+06	8.58E+06	7.14E+06	7.28E+06	7.58E+06		7.70E+06	23.08	22.63	23.16	23.02	22.96	23.06	22.78		22.4	23.08	23.02	22.59	0.282263	0.050693	0.26303
3142	Q9UJX2	Cell divisio	CDC23	5	7.5	2.85E+06	2.36E+06	2.98E+06	2.62E+06	2.04E+06	2.40E+06	1.92E+06	2.13E+06		21.41	21.43	21.82	21.37	21.18	21.49	20.76	20.91		21.43	21.37	20.83	0.230741	0.159829	0.103362
975	I3L0N3	Vesicle-fus	NSF	16	24.4	5.24E+07	4.00E+07	4.17E+07	4.35E+07	3.96E+07	4.25E+07	3.98E+07	3.58E+07	3.02E+07	25.61	25.46	25.55	25.49	25.38	25.59	25.24	24.89	24.36	25.55	25.49	24.89	0.077719	0.104788	0.442223
1091	O00139-2	Kinesin-like	KIF2A	4	5.6	4.22E+06	3.34E+06	3.63E+06	3.21E+06	3.73E+06	3.20E+06	3.57E+06	2.91E+06		21.97	21.94	22.12	21.64	22.02	21.91	21.7	21.32		21.97	21.91	21.51	0.096671	0.193569	0.271565
1198	O43617-2	Trafficking	TRAPPC3	4	31.3	4.41E+06	5.10E+06	3.66E+06	4.41E+06	3.62E+06	4.49E+06	3.92E+06			22.02	22.5	22.14	22.08	21.98	22.37	21.85			22.14	22.08	21.85	0.251787	0.201853	

1243	O60934	Nibrin	NBN	4	6.4		1.49E+06	1.71E+06	1.89E+06	1.42E+06	1.72E+06	1.82E+06				20.79	21.06	20.87	20.69	20.99	20.7			20.92	20.87	20.7	0.18833	0.148879	
691	H0Y8C4	Serine/thre	PPP2R5D	4	9.1	2.45E+06	2.91E+06	4.03E+06	2.38E+06	2.80E+06	4.81E+06				21.19	21.72	22.26	21.21	21.66	22.45				21.72	21.66		0.538635	0.627533	
2254	Q15024	Exosome c	EXOSC7	5	24.1	8.18E+06	1.17E+07	8.21E+06	9.35E+06	8.65E+06	8.27E+06	6.60E+06	8.76E+06	5.78E+06	22.93	23.68	23.26	23.16	23.2	23.23	22.62	22.84	21.9	23.26	23.2	22.62	0.376678	0.037364	0.490788
1973	P62195	26S protea	PSMC5	21	58.4	1.43E+08	1.47E+08	1.28E+08	1.37E+08	1.23E+08	1.22E+08	1.14E+08	1.56E+08	8.47E+07	27.17	27.5	27.28	27.23	27.06	27.22	26.78	26.99	25.82	27.28	27.22	26.78	0.171138	0.095888	0.624297
3073	Q9P015	39S riboso	MRPL15	6	23.6	6.58E+06	6.78E+06	5.62E+06	6.42E+06	6.15E+06	5.68E+06	4.69E+06	5.42E+06	2.72E+06	22.58	22.87	22.76	22.65	22.76	22.7	22.11	22.12	20.83	22.76	22.7	22.11	0.146312	0.054083	0.744089
1177	O15460-2	Prolyl 4-hy	P4HA2	6	14.6	3.83E+06	3.64E+06	4.58E+06	4.17E+06	2.75E+06	3.98E+06	3.42E+06	3.39E+06		21.84	22.06	22.44	22	21.63	22.21	21.65	21.51		22.06	22	21.58	0.301439	0.29391	0.100329
2612	Q8TCS8	Polyribonu	PNPT1	17	24.6	2.96E+07	2.58E+07	2.85E+07	2.66E+07	2.54E+07	2.48E+07	3.23E+07	3.01E+07	1.84E+07	24.8	24.81	24.97	24.72	24.77	24.75	24.94	24.68	23.72	24.81	24.75	24.68	0.093889	0.025368	0.6432
1669	P30566	Adenylosu	ADSL	6	16.1			6.93E+06	6.68E+06	6.92E+06	1.00E+07	4.09E+06					22.99	22.7	22.93	23.49	21.94			22.99	22.93	21.94		0.40374	
694	E9PGT3	Ribosomal	RPS6KA1	5	18.4	3.62E+06	3.57E+06	2.91E+06	3.46E+06	2.72E+06	4.05E+06	3.08E+06	2.97E+06		21.75	22.04	21.77	21.71	21.62	22.24	21.5	21.35		21.77	21.71	21.43	0.161104	0.334789	0.106825
3098	Q9UBF2-2	Coatomer	COPG2	3	6.1		2.83E+06	1.91E+06		2.34E+06						21.66	21.22		21.38					21.44	21.38		0.306886		
107	A0A087WZ	Myotubula	SBF2	10	7.1	8.63E+06	5.80E+06	4.51E+06	7.23E+06	5.45E+06	5.14E+06	8.17E+06	4.88E+06		23	22.65	22.42	22.81	22.59	22.56	22.9	22		22.65	22.59	22.45	0.292125	0.13973	0.633504
812	F8W1U7	ADP-ribosy	ARFGAP1	3	15.9	3.10E+06	3.15E+06	2.59E+06			2.47E+06	2.86E+06	2.76E+06	2.61E+06	21.54	21.84	21.61			21.54	21.4	21.25	20.73	21.61	21.54	21.25	0.161541		0.35326
1849	P49915-2	GMP synth	GMPS	17	40.1	3.05E+07	3.30E+07	3.83E+07	3.15E+07	3.38E+07	2.98E+07	3.57E+07	2.87E+07	3.31E+07	24.86	25.14	25.44	24.97	25.17	25.08	25.11	24.6	24.56	25.14	25.08	24.6	0.294086	0.099677	0.306443
2576	Q8NB90	Spermatog	SPATA5	4	8.8	2.10E+06	5.69E+06	2.72E+06	2.25E+06	2.84E+06	2.55E+06	2.38E+06	2.90E+06		20.94	22.63	21.66	21.12	21.68	21.6	21.11	21.31		21.66	21.6	21.21	0.845656	0.305301	0.137225
2279	Q15386	Ubiquitin-p	UBE3C	6	7	2.36E+06	2.49E+06	2.55E+06		2.35E+06	2.42E+06		2.53E+06		21.11	21.51	21.58		21.38	21.5		21.1		21.51	21.44	21.1	0.252858	0.084649	
2691	Q93100-4	Phosphory	PHKB	6	7.1	6.54E+06	6.30E+06	6.77E+06	4.80E+06	5.97E+06	6.35E+06	7.33E+06	6.70E+06	2.58E+06	22.57	22.77	22.95	22.19	22.7	22.85	22.74	22.43	20.71	22.77	22.7	22.43	0.19096	0.346125	1.096585
2581	Q8NBS9-2	Thioredoxi	TXNDC5	9	35.8	2.86E+07	3.18E+07	4.26E+07	1.79E+07	3.27E+07	2.83E+07	3.13E+07	2.92E+07	7.71E+06	24.72	25.07	25.57	24.14	25.12	25	24.87	24.62	22.41	25.07	25	24.62	0.426654	0.537801	1.354449
2286	Q15437	Protein tra	SEC23B	4	10		2.38E+06	2.53E+06	2.24E+06		2.88E+06					21.44	21.57	21.11		21.78				21.51	21.44		0.092607	0.473886	
2152	Q13085	Acetyl-CoA	ACACA	53	29	6.96E+07	7.55E+07	7.03E+07	7.87E+07	7.26E+07	7.30E+07	6.86E+07	7.21E+07	6.04E+07	26.01	26.42	26.41	26.35	26.29	26.39	25.95	25.9	25.3	26.41	26.35	25.9	0.235663	0.050482	0.359123
2729	Q96EN8	Molybdenu	MOCOS	6	8.7		3.41E+06	1.69E+06	3.32E+06	2.42E+06	1.82E+06					21.97	21.04	21.68	21.43	21.11				21.5	21.43		0.6588	0.290142	
2149	Q13045-2	Protein flig	FLII	15	15.8	1.37E+07	8.86E+06	9.46E+06	1.10E+07	1.06E+07	9.17E+06	1.12E+07	8.70E+06		23.65	23.24	23.45	23.38	23.5	23.38	23.32	22.84		23.45	23.38	23.08	0.203521	0.069946	0.344778
2514	Q86W42-3	THO comp	THOC6	4	18.2	8.76E+06	6.43E+06	6.50E+06	7.54E+06	4.94E+06	6.29E+06	6.81E+06	7.79E+06		23.02	22.8	22.91	22.88	22.44	22.84	22.65	22.67		22.91	22.84	22.66	0.111509	0.24584	0.013051
3246	Q9Y5M8	Signal reco	SRPRB	9	37.6	1.96E+07	2.14E+07	2.04E+07	1.58E+07	2.01E+07	2.79E+07	1.49E+07	1.67E+07	1.84E+07	24.19	24.5	24.51	23.96	24.43	24.98	23.79	23.78	23.72	24.5	24.43	23.78	0.179897	0.510364	0.039139
863	G8JLD5	Dynamin-1	DNM1L	15	29.2	1.83E+07	2.09E+07	2.33E+07	1.60E+07	1.95E+07	2.14E+07	1.48E+07	1.86E+07	1.15E+07	24.11	24.45	24.71	23.99	24.38	24.56	23.78	23.95	23.04	24.45	24.38	23.78	0.299957	0.295413	0.485831
670	E7EX90	Dynactin su	DCTN1	40	43.7	3.65E+07	5.43E+07	5.56E+07	4.92E+07	5.46E+07	5.14E+07	5.83E+07	3.95E+07	1.53E+07	25.09	25.92	26.01	25.71	25.86	25.85	25.77	25.05	23.48	25.92	25.85	25.05	0.507349	0.083664	1.168545
357	B1ALK7	Rho guanin	ARHGEF7	4	8.1		2.18E+06	2.66E+06		2.54E+06	2.13E+06	2.91E+06				21.35	21.64		21.49	21.35	21.42			21.49	21.42	21.42	0.202291	0.095759	
2743	Q96HS1	Serine/thre	PGAM5	21	68.5	9.61E+08	8.25E+08	6.60E+08	8.77E+08	7.73E+08	8.66E+08	3.89E+08	5.38E+08	2.71E+08	29.95	30.22	29.85	29.88	29.69	29.96	28.41	28.62	27.19	29.95	29.88	28.41	0.1907	0.136343	0.772674
1836	P49643	DNA prima	PRIM2	2	5.3	2.39E+06	1.70E+06	2.32E+06	2.38E+06		1.62E+06	1.46E+06			21.13	20.97	21.45	21.21		20.9	20.37			21.13	21.06	20.37	0.244867	0.221876	
1023	J3KTJ8	60S riboso	RPL26	6	37.1	1.21E+07	1.49E+07	1.65E+07	1.56E+07	1.21E+07	2.23E+07	1.50E+07	1.83E+07	1.61E+07	23.5	24	24.2	23.93	23.68	24.62	23.81	23.93	23.57	24	23.93	23.81	0.360931	0.487234	0.184459
3167	Q9UNH6-2	Sorting ne	SNX7	3	11	3.58E+06	3.83E+06	3.60E+06	4.49E+06	3.42E+06	3.46E+06	4.31E+06	5.33E+06	5.23E+06	21.73	22.16	22.1	22.1	21.91	22.03	22.01	22.09	21.76	22.1	22.03	22.01	0.23037	0.093093	0.170434
2485	Q7L592	NADH deh	NDUFAF7	6	16.1	8.20E+06	6.82E+06	5.72E+06	7.20E+06	6.21E+06	6.21E+06	7.63E+06	1.01E+07	6.61E+06	22.93	22.88	22.78	22.81	22.78	22.83	22.79	23.04	22.16	22.88	22.81	22.79	0.07802	0.021398	0.454258
1890	P52701	DNA mism	MSH6	30	25	6.30E+07	6.32E+07	6.07E+07	6.58E+07	6.91E+07	6.06E+07	5.82E+07	5.83E+07	1.96E+07	25.85	26.17	26.14	26.06	26.22	26.07	25.76	25.61	23.81	26.14	26.07	25.61	0.174284	0.090919	1.082791
1473	P10768	S-formylglu	ESD	5	20.6	4.05E+06	8.78E+06	6.31E+06			6.11E+06	5.62E+06	4.63E+06		21.91	23.23	22.88			22.81	22.4	21.92		22.88	22.81	22.16	0.688584		0.338515
1071	M0QXN5	Nuclear po	NUP62	5	16.8		1.25E+07	1.38E+07		1.22E+07	1.32E+07	8.81E+06				23.77	23.94		23.69	23.87	23.02			23.86	23.78	23.02	0.124337	0.124832	
833	G3V1B6	Apolipopro	APOO	2	22.8		2.59E+06	2.39E+06			2.28E+06					21.56	21.49			21.45				21.52	21.45		0.048592		
3056	Q9NYJ8	TGF-beta-a	TAB2	7	15	1.02E+07	8.13E+06	7.29E+06	8.52E+06	1.37E+07	7.27E+06	9.93E+06	1.12E+07	5.31E+06	23.27	23.13	23.08	23.02	23.86	23.06	23.18	23.19	21.78	23.13	23.06	23.18	0.095567	0.475591	0.809722
1406	P05783	Keratin, ty	KRT18	26	73.7	3.96E+08	3.97E+08	3.37E+08	3.88E+08	4.70E+08	3.85E+08	2.49E+08	2.71E+08	1.94E+08	28.6	29	28.94	28.47	29.01	28.86	27.88	27.67	26.8	28.94	28.86	27.67	0.214327	0.279039	0.574417
1160	O15160-2	DNA-direct	POLR1C	7	25.4	5.95E+06	5.35E+06	5.21E+06	6.14E+06	5.08E+06	4.84E+06	4.91E+06	6.41E+06	4.88E+06	22.43	22.56	22.62	22.58	22.48	22.46	22.18	22.37	21.64	22.56	22.48	22.18	0.096375	0.064453	0.379702
2621	Q8TEM1	Nuclear po	NUP210	21	13.8	3.88E+07	3.32E+07	3.00E+07	3.43E+07	3.19E+07	2.77E+07	3.42E+07	3.25E+07	2.47E+07	25.18	25.16	25.04	25.16	25.08	24.96	25.01	24.78	24.1	25.16	25.08	24.78	0.074231	0.099744	0.475648
1718	P35813	Protein ph	PPM1A	13	51.8	8.27E+07	7.80E+07	6.99E+07	7.78E+07	6.97E+07	7.15E+07	8.53E+07	1.07E+08	4.75E+07	26.31	26.46	26.41	26.33	26.24	26.35	26.33	26.43	25.03	26.41	26.33	26.33	0.077526	0.058992	0.781055
1471	X6R5C5	Carboxype	CTSA	2	6.9		2.13E+06	1.91E+06			1.91E+06					21.32	21.22			21.2				21.27	21.2		0.072497		
3229	Q9Y4B5	Microtubu	MTCL1	8	5.2	3.30E+06	2.40E+06	2.47E+06	2.83E+06	2.54E+06	1.85E+06		2.44E+06		21.63	21.45	21.54	21.46	21.49	21.14		21.04		21.54	21.46	21.04	0.085154	0.192462	
990	J3KN10	Phosphatic	PI4KA	41	23.2	7.04E+07	4.99E+07	4.79E+07	5.89E+07	4.81E+07	4.72E+07	3.67E+07	2.27E+07	6.84E+06	26.06	25.75	25.82	25.92	25.69	25.74	25.14	24.26	22.2	25.82	25.74	24.26	0.16669	0.119281	1.508719
54	J3KSI4	Mannose-P	MPDU1	2	38.5			5.56E+06			5.53E+06						22.74			22.66				22.74	22.66				
1261	O75385	Serine/thre	ULK1	7	7.5	4.81E+07	3.47E+07	3.16E+07	3.67E+07	3.34E+07	3.07E+07	3.86E+07	5.00E+07		25.48	25.24	25.09	25.25	25.16	25.13	25.2	25.38		25.24	25.16	25.29	0.198814	0.065565	0.132061
1856	P50454	Serpin H1	SERPINH1	21	58.4	7.27E+08	7.67E+08	7.39E+08	8.73E+08	1.01E+09	8.16E+08	8.62E+08	7.84E+08	6.86E+08	29.47	30.06	29.95	29.83	30.08	29.87	29.57	29.27	28.65	29.95	29.87	29.27	0.311313	0.136589	0.471167
384	E9PNU1	Nucleoside	NME7	4	17.2	2.55E+06	1.92E+06	1.94E+06	2.20E+06	2.00E+06	2.05E+06	2.18E+06	2.33E+06	2.29E+06	21.25	21.19	21.24	21.08	21.15	21.29	20.93	20.98	20.51	21.24	21.15	20.93	0.033434	0.104692	0.257929
1563	P19784	Casein kina	CSNK2A2	7	23.4	5.04E+06	6.20E+06	6.82E+06	3.53E+06	6.26E+06	5.55E+06	4.49E+06	4.83E+06	4.07E+06	22.19	22.75	22.97	21.74	22.79	22.67	22.07	21.99	21.31	22.75	22.67	21.99	0.401222	0.574545	0.417878
653	E7ESY4	Metastasis	MTA1	9	18.2	1.81E+07	1.39E+07	1.45E+07	1.56E+07	1.55E+07	1.33E+07	1.45E+07	1.65E+07		24.08	23.9	24.01	23.93	24.03	23.88	23.76	23.78		24.01	23.93	23.77	0.09158	0.078714	0.01469
177	J3KNW4	Four and a	FHL2	10	31.6	2.08E+07	2.26E+07	1.79E+07	2.19E+07	1.40E+07	1.70E+07	2.51E+07	1.85E+07	1.14E+07	24.26	24.6	24.35	24.45	23.89	24.26	24.58	23.95	23.03	24.35	24.26	23.95	0.174074	0.285273	0.77969
3020	Q9NSK0-5	Kinesin lig	KLC4	8	28.6	7.45E+06	7.15E+06	7.28E+06	7.60E+06	6.00E+06	6.76E+06	9.60E+06	6.61E+06	5.39E+06	22.76	22.97	23.08	22.88	22.71	22.94	23.14	22.4	21.8	22.97	22.88	22.4	0.162164	0.118045	0.670052
2888	Q9BZE1	39S riboso	MRPL37	10	27.4	1.11E+07	1.26E+07	1.19E+07	1.14E+07	1.27E+07	1.18E+07	1.23E+07	1.46E+07	7.60E+06	23.39	23.78	23.75	23.43	23.75	23.67	23.46	23.61	22.37	23.75	23.67	23.46	0.214844	0.166109	0.673902
1171	O15355	Protein ph	PPM1G	5	10.3	4.89E+06	5.31E+06	8.07E+06	4.83E+06	6.12E+06	4.83E+06	5.30E+06			22.15	22.54	23.24	22.2	22.75	22.46	22.3			22.54	22.46	22.3	0.553973	0.275499	
2985	Q9HCC0-2	Methylcrot	MCCC2	5	14.1	5.50E+06	4.50E+06	5.29E+06	5.02E+06	3.39E+06	5.08E+06	4.33E+06			22.29	22.35	22.66	22.27	21.9	22.53	22.02			22.35	22.27	22.02	0.198598	0.319222	
1588	P22626	Heterogen	HNRNPA2B	13	42.8	8.73E+07	9.96E+07	1.03E+08	9.04E+07	9.33E+07	9.85E+07	6.05E+07	5.88E+07	4.06E+07	26.45	26.79	26.99	26.59	26.71	26.84	25.82	25.63	24.82	26.79	26.71	25.63	0.275709	0.12482	0.529383
1212	O60244	Mediator c	MED14	11	10.3		3.07E+06	3.61E+06	3.78E+06	3.51E+06	3.11E+06	5.25E+06	3.87E+06	2.98E+06		21.8	22.11	21.86	21.94	21.87	22.29	21.7	20.93	21.95	21.87	21.7	0.220199	0.044573	0.677551
3266	Q9Y6Y0	Influenza v	IVNS1ABP	25	57.2	3.29E+08	3.49E+08	3.22E+08	2.66E+08	4.14E+08	3.77E+08	4.96E+08	3.95E+08	1.83E+08	28.36	28.81	28.89	28.09	28.73	28.83	28.9	28.14	26.69	28.81	28.73	28.14	0.284372	0.397193	1.12381
1389	P04818	Thymidylat	TYMS	8	38.3	9.53E+06	1.86E+07	1.65E+07	1.35E+07	1.63E+07	1.80E+07	1.37E+07	1.42E+07	9.82E+06	23.15	24.3	24.2	23.71	24.12	24.37	23.66	23.55	22.75	24.2	24.12	23.55	0.639168	0.334834	0.49526
1600	P23528	Cofilin-1	CFL1	17	80.7	6.25E+08	6.15E+08	8.49E+08	4.65E+08	6.59E+08	6.71E+08	6.29E+08	6.22E+08	2.69E+08	29.29	29.51	30.47	28.76	29.42	29.6	29.1	28.78	27.17	29.51	29.42	28.78	0.626081	0.446314	1.035883
87	A0A087WY	Nuclear mi	NUMA1	18	10.7	9.81E+06	1.24E+07	1.42E+07	1.56E+07	1.12E+07	1.18E+07	6.81E+06	5.56E+06	6.01E+06	23.19	23.76	23.98	23.93	23.55	23.67	22.65	22.18	21.97	23.76	23.67	22.18	0.406222	0.190334	0.349728
424	C9IY40	39S riboso	MRPL2	2	16.4		3.82E+06	4.59E+06			4.00E+06					22.15	22.44			22.21				22.3	22.21		0.20593		
245	A0A0D9SE	Ran-bindin	RANBP10	7	21.1	1.71E+07	8.91E+06	1.08E+07	1.54E+07	8.36E+06	1.05E+07	1.63E+07	1.41E+07	1.16E+07	23.99	23.25	23.63	23.9	23.15	23.54	23.93	23.53	23.05	23.63	23.54	23.53	0.370821	0.37264	0.438328
2645	Q8WWM7	Ataxin-2-lik	ATXN2L	4	5.2		2.07E+06	1.25E+06		1.98E+06	1.24E+06					21.29	20.64		21.13	20.62				20.97	20.88		0.457161	0.36171	
2007	P62917	60S riboso	RPL8	13	46.7	1.22E+08	9.79E+07	1.85E+08	1.11E+08	4.72E+07	2.28E+08	2.13E+08	1.55E+08		26.92	26.77	27.92	26.83	25.68	28.15	27.71	26.98		26.92	26.83	27.34	0.627589	1.240697	0.519256
3180	Q9UQE7	Structural	SMC3	41	36.6	5.35E+07	6.04E+07	5.62E+07	6.09E+07	5.83E+07	5.14E+07	4.46E+07	3.92E+07	3.97E+07	25.64	26.09	26.03	25.94	25.97	25.85	25.37	25.03	24.78	26.03	25.94	25.03	0.242579	0.064871	0.295864
1697	P33991	DNA replic	MCM4	19	28.3	2.96E+07	5.39E+07	2.89E+07	2.94E+07	3.15E+07	2.69E+07	1.49E+07	2.16E+07		24.8	25.9	24.98	24.84	25.06	24.89	23.79	24.18		24.98	24.89	23.99	0.591483	0.111591	0.269434
2239	Q14789-4	Golgin sub	GOLGB1	34	14.6	1.30E+07	2.16E+07	2.02E+07	2.10E+07	2.05E+07	1.81E+07	1.74E+07	2.35E+07		23.6	24.53	24.48	24.39	24.46	24.38	24.04	24.31		24.48	24.39	24.17	0.526041	0.044288	0.187879
2554	Q8N1I0	Dedicator	DOCK4	102	55.7	2.34E+09	2.07E+09	1.95E+09	3.84E+09	4.45E+09	3.33E+09	3.36E+09	6.88E+09	5.66E+09	31.83	32.02	32.16	31.89	31.93	32.07	31.45	32.15	30.96	32.02	31.93	31.45	0.162932	0.094245	0.595247
2172	Q13405	39S riboso	MRPL49	3	23.5	1.03E+07	1.24E+07	1.07E+07	1.17E+07	1.67E+07	1.03E+07	9.46E+06	1.27E+07		23.28	23.76	23.61	23.48	24.17	23.52	23.12	23.39		23.61	23.52	23.26	0.244467	0.387683	0.193824
679	E9PCY5	DNA topois	TOP2B	7	12.3	5.66E+06	6.08E+06	5.44E+06	6.32E+06	5.65E+06	5.76E+06	3.54E+06	6.54E+06	3.40E+06	22.35	22.71	22.71	22.62	22.62	22.73	21.69	22.39	21.08	22.71	22.62	21.69	0.207991	0.061333	0.652716
318	A6NJD9	39S riboso	MRPL23	3	27.6	6.48E+06	6.90E+06	7.31E+06	7.24E+06	5.83E+06	6.98E+06			5.37E+06	22.56	22.91	23.09	22.82	22.68	23			21.79	22.91	22.82	21.79	0.268557	0.160004	
1837	P49721	Proteasom	PSMB2	7	32.3	1.38E+07	2.32E+07	2.71E+07	1.69E+07	2.19E+07	2.39E+07	8.51E+06	1.31E+07		23.66	24.61	24.89	24.06	24.52	24.72	22.96	23.43		24.61	24.52	23.19	0.647094	0.337814	0.336599
1682	P31751-2	RAC-beta s	AKT2	6	18.3	2.39E+06	5.12E+06	5.24E+06	5.55E+06	4.27E+06	5.90E+06	5.40E+06			21.13	22.5	22.64	22.41	22.21	22.76	22.34			22.5	22.41	22.34	0.833868	0.28174	
3120	Q9UGP8	Translocati	SEC63	13	21.1	2.68E+07	1.88E+07	2.34E+07	2.03E+07	2.52E+07	2.06E+07	1.76E+07	1.43E+07	2.15E+07	24.61	24.32	24.73	24.32	24.75	24.52	24.05	23.57	23.96	24.61	24.52	23.96	0.211594	0.211802	0.255014
986	I3L3P7	40S riboso	RPS15A	8	68	8.66E+07	8.64E+07	8.40E+07	8.48E+07	5.67E+07	1.06E+08	6.40E+07	9.86E+07	5.83E+07	26.39	26.58	26.65	26.49	25.93	26.99	25.87	26.34	25.26	26.58	26.49	25.87	0.133926	0.528701	0.543787
2710	Q96BN8	Ubiquitin t	OTULIN	5	14.2	8.75E+06	1.11E+07	8.25E+06	9.41E+06	8.31E+06	8.56E+06		1.04E+07		23.02	23.59	23.26	23.17	23.14	23.27		23.08		23.26	23.17	23.08	0.287718	0.068069	
1767	P42166	Lamina-ass	TMPO	15	28.7	1.72E+08	1.36E+08	1.18E+08	1.93E+08	1.45E+08	1.25E+08	1.60E+08	1.45E+08	8.72E+07	27.47	27.38	27.12	27.6	27.29	27.26	27.24	26.88	25.9	27.38	27.29	26.88	0.180602	0.189135	0.694279

1123	O00764-2	Pyridoxal k	PDXK	2	9.5		6.11E+06	5.60E+06			5.46E+06					22.72	22.75			22.64				22.74	22.64		0.022901		
3124	Q9UHB9	Signal reco	SRP68	12	22.5	1.04E+07	1.14E+07	1.69E+07	7.30E+06	1.11E+07	1.11E+07	9.87E+06	4.16E+06		23.29	23.63	24.24	22.83	23.54	23.6	23.17	21.8		23.63	23.54	22.48	0.477356	0.426061	0.973392
127	A0A087X1I	U4/U6.U5	USP39	3	8		2.94E+06	3.15E+06			2.77E+06					21.73	21.9			21.72				21.82	21.72		0.117474		
1122	O00743	Serine/thre	PPP6C	7	29.2	1.67E+07	1.52E+07	1.21E+07	1.48E+07	1.37E+07	1.61E+07	1.49E+07	1.91E+07	9.88E+06	23.96	24.03	23.78	23.85	23.86	24.17	23.79	23.99	22.77	23.96	23.86	23.79	0.131224	0.181961	0.658478
2143	Q12931-2	Heat shock	TRAP1	20	35.2	1.38E+08	1.07E+08	1.17E+08	1.05E+08	1.17E+08	1.21E+08	1.13E+08	1.12E+08	8.46E+07	27.09	26.95	27.11	26.75	26.99	27.21	26.77	26.48	25.8	27.09	26.99	26.48	0.08533	0.227334	0.500025
1918	P55036	26S protea	PSMD4	17	51.2	9.72E+08	6.94E+08	5.31E+08	9.57E+08	5.97E+08	6.74E+08	5.63E+08	9.87E+08	6.87E+08	29.99	29.73	29.5	30.05	29.3	29.63	29.03	29.7	28.69	29.73	29.63	29.03	0.245089	0.378779	0.515183
597	D6RBW1	Eukaryotic	EIF4E	3	13.1	3.92E+06	4.56E+06	4.17E+06	3.84E+06	4.28E+06	4.80E+06	3.27E+06			21.87	22.37	22.31	21.88	22.21	22.45	21.6			22.31	22.21	21.6	0.274458	0.285993	
714	E9PKV2	39S ribosoı	MRPL17	2	11.3	3.57E+06	4.69E+06	3.98E+06	4.57E+06	4.93E+06	3.82E+06				21.73	22.4	22.25	22.13	22.43	22.15				22.25	22.15		0.355666	0.168467	
1113	O00442	RNA 3-terr	RTCA	3	9	3.03E+06	2.78E+06	2.56E+06			2.39E+06				21.52	21.63	21.59			21.49				21.59	21.49		0.054217		
2951	Q9H5Z1-2	Probable A	DHX35	7	11.9	6.13E+06	5.07E+06	4.94E+06	7.31E+06	4.78E+06	3.87E+06	8.34E+06	6.92E+06		22.47	22.49	22.55	22.84	22.39	22.17	22.92	22.48		22.49	22.39	22.7	0.039121	0.341112	0.310597
1748	P40222	Alpha-taxil	TXLNA	3	7		2.37E+06	2.81E+06		3.00E+06	1.97E+06	2.76E+06				21.44	21.73		21.74	21.23	21.32			21.58	21.48	21.32	0.203566	0.359065	
2816	Q99986	Serine/thre	VRK1	3	9.3	1.85E+06	2.05E+06	2.03E+06		1.94E+06	1.98E+06				20.77	21.27	21.29		21.1	21.24				21.27	21.17		0.295489	0.095873	
335	H7BY10	60S ribosoı	RPL23A	6	27.2		1.69E+07	2.31E+07		1.43E+07	2.55E+07	1.82E+07				24.2	24.7		23.92	24.78	24.09			24.45	24.35	24.09	0.350213	0.60834	
1939	P60660-2	Myosin ligł	MYL6	9	73.5	3.02E+08	3.63E+08	3.07E+08	4.88E+08	4.05E+08	3.06E+08	3.61E+08	4.78E+08	4.02E+08	28.16	28.86	28.81	28.79	28.71	28.63	28.3	28.4	27.8	28.81	28.71	28.3	0.392509	0.078507	0.318441
2460	Q6UXN9	WD repeat	WDR82	7	27.8	1.08E+07	1.19E+07	1.08E+07	1.03E+07	1.10E+07	1.14E+07	1.35E+07	1.25E+07		23.34	23.7	23.63	23.32	23.53	23.62	23.61	23.37		23.63	23.53	23.49	0.191925	0.157898	0.171455
1131	O14656	Torsin-1A	TOR1A	2	7.2	1.60E+06	1.47E+06	1.17E+06		1.03E+06	1.21E+06				20.51	20.76	20.54		20.29	20.59				20.54	20.44		0.140092	0.207116	
1479	P11166	Solute carr	SLC2A1	3	5.5	1.30E+07	7.94E+06	9.35E+06	8.75E+06	1.25E+07	8.94E+06	8.46E+06	1.12E+07	6.13E+06	23.6	23.1	23.43	23.06	23.72	23.33	22.94	23.19	22.02	23.43	23.33	22.94	0.252823	0.335628	0.619079
262	A0A0G2JN	Protein scr	SCRIB	16	12.7	1.11E+07	8.80E+06	1.13E+07	1.02E+07	9.18E+06	1.09E+07	8.06E+06	6.44E+06	5.92E+06	23.39	23.24	23.7	23.29	23.27	23.58	22.88	22.37	21.94	23.39	23.29	22.37	0.237196	0.17085	0.474014
1861	P50748	Kinetochor	KNTC1	23	12.3	2.27E+07	2.11E+07	1.74E+07	1.94E+07	1.81E+07	1.77E+07	2.53E+07	2.40E+07	1.94E+07	24.38	24.47	24.28	24.25	24.28	24.33	24.6	24.35	23.8	24.38	24.28	24.35	0.096578	0.039106	0.408512
659	E7EUI8	Glucose-6-	G6PD	7	20.6	8.30E+06	1.16E+07	1.13E+07	8.84E+06	1.12E+07	1.61E+07	1.42E+07	7.88E+06	1.67E+07	22.95	23.66	23.7	23.07	23.55	24.17	23.72	22.7	23.63	23.66	23.55	23.63	0.424662	0.552027	0.566986
2202	Q14019	Coactosin-l	COTL1	5	21.8	1.13E+07	1.59E+07	1.07E+07	1.26E+07	9.12E+06	1.02E+07	7.70E+06			23.41	24.09	23.61	23.59	23.26	23.5	22.82			23.61	23.5	22.82	0.349803	0.170208	
2784	Q96SB4	SRSF prote	SRPK1	4	7.8	3.57E+06	3.55E+06	4.32E+06	2.45E+06	3.47E+06	4.34E+06				21.73	22.03	22.36	21.27	21.92	22.33				22.03	21.92		0.319048	0.535651	
1897	P52948-5	Nuclear po	NUP98	19	12.1	4.62E+07	3.60E+07	4.35E+07	3.38E+07	3.73E+07	3.87E+07	2.75E+07	2.85E+07	4.62E+06	25.45	25.29	25.59	25.12	25.34	25.46	24.7	24.58	21.54	25.45	25.34	24.58	0.150487	0.171607	1.795515
292	A1L0T0	Acetolacta	ILVBL	6	16.6	4.58E+06	5.33E+06	5.61E+06	4.61E+06	4.97E+06	6.72E+06	4.28E+06	6.80E+06		22.06	22.55	22.76	22.14	22.44	22.93	22	22.45		22.55	22.44	22.22	0.359707	0.398559	0.315588
3232	Q9Y4E8	Ubiquitin c	USP15	57	59.1	7.47E+08	8.80E+08	7.80E+08	9.03E+08	7.94E+08	8.80E+08	7.92E+08	7.60E+08	3.57E+08	29.53	30.36	30.06	29.95	29.78	29.98	29.43	29.2	27.61	30.06	29.95	29.2	0.41645	0.10825	0.993533
3156	Q9ULK4-4	Mediator c	MED23	50	42.4	9.85E+08	6.52E+08	7.82E+08	7.94E+08	9.47E+08	1.03E+09	5.87E+08	8.85E+08	7.81E+08	30.1	29.61	30.1	29.62	29.99	30.32	29.05	29.49	28.9	30.1	29.99	29.05	0.280472	0.352618	0.303455
2865	Q9BVK6	Transmem	TMED9	2	8.9	1.35E+06	1.99E+06	3.28E+06			1.84E+06		1.25E+06		20.29	21.23	21.95			21.12		20.07		21.23	21.12	20.07	0.832513		
1544	P17812	CTP syntha	CTPS1	20	36.2	1.36E+08	1.23E+08	1.18E+08	1.23E+08	1.19E+08	1.25E+08	1.22E+08	1.13E+08	9.26E+07	27.04	27.26	27.12	27.01	27.02	27.26	26.86	26.5	25.98	27.12	27.02	26.5	0.109404	0.145495	0.443886
2673	Q92769-3	Histone de	HDAC2	7	29.5	4.23E+07	3.24E+07	3.49E+07	3.43E+07	3.47E+07	2.95E+07	3.12E+07	3.27E+07	2.73E+07	25.34	25.1	25.27	25.16	25.21	25.07	24.86	24.8	24.23	25.27	25.16	24.8	0.122648	0.071752	0.346752
301	A3KN83-3	Protein str	SBNO1	10	9.9	2.89E+06	3.44E+06	3.59E+06	2.93E+06	4.35E+06	3.12E+06	4.74E+06		3.84E+06	21.43	21.98	22.09	21.51	22.24	21.87	22.12		21.22	21.98	21.87	21.67	0.356742	0.362736	0.637874
2127	Q10570	Cleavage a	CPSF1	16	11.2	1.33E+07	1.20E+07	1.14E+07	1.10E+07	1.74E+07	1.12E+07	6.75E+06	7.64E+06	3.25E+06	23.61	23.72	23.72	23.38	24.22	23.61	22.64	22.65	21.02	23.72	23.61	22.64	0.060869	0.437421	0.935396
1262	O75391	Sperm-ass	SPAG7	4	26.9	2.84E+06	6.18E+06	4.58E+06		6.77E+06	2.84E+06		3.57E+06		21.4	22.75	22.44		22.9	21.76		21.58		22.44	22.33	21.58	0.70801	0.804411	
1831	P49458	Signal reco	SRP9	3	34.9	6.35E+06					4.67E+06		4.63E+06		22.53					22.42		21.92		22.53	22.42	21.92			
398	B5MDF5	GTP-bindir	RAN	12	45.5	1.91E+08	2.22E+08	1.80E+08	2.18E+08	1.48E+08	2.56E+08	1.12E+08	1.71E+08	1.68E+08	27.64	28.16	27.87	27.75	27.34	28.35	26.75	27.13	26.59	27.87	27.75	26.75	0.261813	0.509383	0.277901
2433	Q6NZI2	Polymeras	PTRF	8	23.3	9.63E+06	1.38E+07	1.31E+07	1.23E+07	1.31E+07	1.24E+07	1.12E+07	1.35E+07		23.17	23.89	23.9	23.56	23.81	23.78	23.32	23.46		23.89	23.78	23.39	0.418042	0.131903	0.099009
2724	Q96DV4	39S ribosoı	MRPL38	5	15.5	7.42E+06	7.19E+06	7.25E+06	5.57E+06	6.65E+06	6.54E+06	6.97E+06	6.40E+06	6.15E+06	22.75	22.97	23.07	22.42	22.86	22.89	22.68	22.36	22.03	22.97	22.86	22.36	0.166264	0.262531	0.326322
1142	O14920-4	Inhibitor of	IKBKB	4	5.9	3.71E+06	4.07E+06	3.50E+06	3.94E+06	5.18E+06	3.20E+06	4.57E+06	3.77E+06		21.78	22.24	22.03	21.91	22.52	21.91	22.09	21.66		22.03	21.91	21.87	0.229938	0.350941	0.309532
878	H0Y6E7	RNA-bindir	RBMX	6	17.8	2.25E+07	3.09E+07	2.78E+07	2.21E+07	2.62E+07	3.54E+07	2.58E+07	3.55E+07		24.36	25.02	24.92	24.47	24.8	25.32	24.64	24.89		24.92	24.8	24.76	0.35494	0.42849	0.174599
1706	P35249	Replicatior	RFC4	8	27.8	1.44E+07	1.41E+07	1.28E+07	1.24E+07	1.28E+07	1.23E+07	1.21E+07	1.30E+07	1.25E+07	23.72	23.92	23.87	23.57	23.77	23.75	23.43	23.42	23.16	23.87	23.75	23.42	0.102915	0.109017	0.156707
1821	P49207	60S ribosoı	RPL34	3	13.7	1.01E+08	7.28E+07	6.00E+07	8.30E+07	5.18E+07	6.70E+07	1.18E+08	7.75E+07		26.7	26.36	26.11	26.45	25.81	26.25	26.83	26.02		26.36	26.25	26.42	0.293657	0.32584	0.572746
2622	Q8TEQ6	Gem-assoc	GEMIN5	24	21.4	3.11E+07	2.72E+07	2.46E+07	2.26E+07	2.52E+07	2.60E+07	1.45E+07	1.79E+07		24.9	24.86	24.79	24.51	24.75	24.81	23.76	23.88		24.86	24.75	23.82	0.058368	0.162386	0.091182
70	A0A087W\	DNA polym	POLD2	5	14.1	6.12E+06	5.89E+06	6.15E+06	6.20E+06	4.96E+06	5.11E+06	5.60E+06	6.24E+06	7.44E+06	22.47	22.67	22.86	22.59	22.44	22.55	22.39	22.33	22.33	22.67	22.55	22.33	0.193418	0.077855	0.036226
1764	P41743	Protein kin	PRKCI	3	8.2	5.55E+06	6.24E+06	6.46E+06	6.38E+06	5.69E+06	4.44E+06				22.31	22.76	22.9	22.64	22.65	22.35				22.76	22.64		0.307911	0.168962	
1095	O00159-2	Unconvent	MYO1C	30	32.9	7.64E+07	7.88E+07	7.04E+07	7.74E+07	8.08E+07	6.39E+07	7.32E+07	7.36E+07	7.42E+07	26.2	26.48	26.42	26.3	26.44	26.18	26.06	25.94	25.6	26.42	26.3	25.94	0.146261	0.132302	0.239627
2974	Q9H9T3-2	Elongator (	ELP3	3	5.8	3.28E+06	3.38E+06	3.08E+06	2.23E+06	3.02E+06	2.83E+06	2.90E+06	2.80E+06		21.62	21.95	21.87	21.1	21.75	21.75	21.41	21.27		21.87	21.75	21.34	0.175073	0.376361	0.098466
2947	Q9H4L7	SWI/SNF-r	SMARCAD1	6	8	2.97E+06	4.64E+06	4.59E+06	5.02E+06	3.55E+06	5.36E+06	4.73E+06	7.45E+06		21.49	22.39	22.44	22.27	21.96	22.62	22.12	22.59		22.39	22.27	22.36	0.533819	0.334472	0.336621
1555	P18615	Negative e	NELFE	10	39.2	1.04E+07	1.37E+07	1.10E+07	1.47E+07	9.24E+06	1.05E+07	1.07E+07	1.21E+07		23.29	23.88	23.66	23.84	23.3	23.54	23.27	23.34		23.66	23.54	23.31	0.298412	0.27203	0.047245
3123	Q9UH99	SUN doma	SUN2	28	55.2	5.54E+08	7.04E+08	7.65E+08	8.09E+08	7.29E+08	7.17E+08	9.91E+08	8.87E+08	4.33E+08	29.04	29.82	30.01	29.7	29.59	29.72	29.9	29.5	27.99	29.82	29.7	29.5	0.514421	0.071658	1.006805
1281	O75792	Ribonuclea	RNASEH2A	6	22.4	5.76E+06	5.35E+06	4.70E+06	5.06E+06	4.65E+06	5.45E+06	5.21E+06	7.04E+06	6.14E+06	22.38	22.56	22.47	22.28	22.35	22.64	22.28	22.51	22.02	22.47	22.35	22.28	0.090934	0.18993	0.243258
2897	Q9C0C2	182 kDa ta	TNKS1BP1	9	7.9	4.03E+06	3.46E+06	3.41E+06	3.11E+06	3.31E+06	3.91E+06	3.21E+06			21.9	21.99	21.99	21.59	21.87	22.18	21.57			21.99	21.87	21.57	0.053772	0.294101	
2217	Q14247-3	Src substra	CTTN	15	33.7	5.21E+07	4.77E+07	4.84E+07	4.52E+07	4.15E+07	4.07E+07	4.16E+07	4.72E+07	3.64E+07	25.6	25.66	25.83	25.57	25.46	25.54	25.29	25.3	24.68	25.66	25.54	25.29	0.121403	0.054382	0.35692
916	H0YN81	WD repeat	WDR61	7	36.4	1.59E+07	1.37E+07	1.01E+07	1.25E+07	1.33E+07	1.23E+07	1.42E+07	1.56E+07	7.56E+06	23.87	23.88	23.54	23.58	23.82	23.75	23.72	23.7	22.37	23.87	23.75	23.7	0.196937	0.123984	0.772056
2885	Q9BYD6	39S ribosoı	MRPL1	3	11.4	5.30E+06	6.20E+06	4.49E+06	4.95E+06	4.44E+06	5.48E+06	4.62E+06	4.01E+06		22.25	22.75	22.41	22.24	22.29	22.65	22.1	21.74		22.41	22.29	21.92	0.255532	0.225267	0.258278
1120	O00625	Pirin	PIR	2	6.6		4.70E+06				4.22E+06					22.41				22.29				22.41	22.29				
1151	O15020-2	Spectrin be	SPTBN2	22	15.7	2.58E+07	1.87E+07	1.67E+07	2.88E+07	1.69E+07	1.44E+07	1.07E+07	1.98E+07		24.56	24.31	24.22	24.83	24.19	24	23.27	24.07		24.31	24.19	23.67	0.178554	0.434397	0.561984
729	G3V158	Deoxyribos	DERA	4	25.2		4.36E+06	3.11E+06		3.20E+06	3.74E+06		4.82E+06			22.31	21.89		21.82	22.13		21.99		22.1	21.97	21.99	0.297657	0.216924	
382	B4DR61	Protein tra	SEC61A1	6	12.7	1.37E+08	3.88E+07	3.69E+07	8.36E+07	3.66E+07	2.47E+07	1.07E+08	7.20E+07	2.02E+08	27.07	25.42	25.39	26.47	25.3	24.74	26.71	25.89	26.83	25.42	25.3	26.71	0.957765	0.885142	0.50927
2948	Q9H583	HEAT repe	HEATR1	19	11	1.25E+07	1.45E+07	1.27E+07	1.28E+07	1.45E+07	1.22E+07	1.58E+07	2.07E+07		23.55	23.95	23.85	23.63	23.93	23.73	23.86	24.14		23.85	23.73	24	0.209663	0.152903	0.194493
2151	Q13057	Bifunctiona	COASY	5	12.6	5.65E+06	7.12E+06	7.62E+06	7.28E+06	4.12E+06	6.39E+06		7.08E+06		22.35	22.96	23.15	22.83	22.14	22.86		22.52		22.96	22.83	22.52	0.418791	0.404286	
734	E9PR30	40S ribosoı	FAU	2	11.2		6.33E+06	1.07E+07			7.31E+06	7.40E+06				22.78	23.61			23.07	22.75			23.19	23.07	22.75	0.587028		
908	Q5SZE2	Ceramide s	CERS2	2	19.4	5.13E+06	4.13E+06	3.91E+06	4.59E+06		3.61E+06				22.2	22.26	22.23	22.13		22.07				22.23	22.1		0.029671	0.041725	
2628	Q8WU90	Zinc finger	ZC3H15	3	10.8	4.19E+06	4.18E+06	3.75E+06	3.76E+06	3.78E+06	4.14E+06				21.96	22.27	22.17	21.85	22.04	22.27				22.17	22.04		0.161614	0.210209	
1200	O43719	HIV Tat-sp	HTATSF1	5	10.2		2.80E+06	4.42E+06		3.19E+06	3.30E+06					21.64	22.39		21.81	21.96				22.01	21.88		0.52896	0.103085	
MUT	A0A087W>	TAR DNA-b	TARDBP	4	21	1.25E+07	1.58E+07	1.77E+07	1.58E+07	1.55E+07	1.27E+07	3.18E+07	3.01E+07	4.93E+07	23.55	24.09	24.31	23.96	24.03	23.81	24.92	24.68	25.1	24.09	23.96	24.92	0.392642	0.109594	0.205893
2257	Q15042	Rab3 GTPa	RAB3GAP1	15	20.9	1.56E+07	2.54E+07	1.95E+07	1.41E+07	1.86E+07	2.12E+07	1.23E+07	1.36E+07		23.84	24.77	24.43	23.78	24.3	24.55	23.46	23.48		24.43	24.3	23.47	0.469942	0.395516	0.014638
1364	P01111	GTPase NR	NRAS	3	16.9	7.54E+06	5.61E+06	6.14E+06		5.17E+06	5.91E+06	6.57E+06		6.19E+06	22.77	22.62	22.85		22.51	22.77	22.62		22.05	22.77	22.64	22.33	0.11637	0.178832	0.401925
3170	Q9UNS2	COP9 signa	COPS3	4	12.1	1.30E+07	2.66E+07	2.48E+07	1.57E+07	2.45E+07	2.43E+07	2.47E+07		1.92E+07	23.6	24.83	24.8	23.94	24.67	24.73	24.56		23.78	24.8	24.67	24.17	0.702639	0.437751	0.551915
1777	P42858	Huntingtin	HTT	15	5.6	6.19E+06	6.85E+06	8.15E+06	6.94E+06	5.94E+06	6.90E+06	3.82E+06	7.67E+06		22.49	22.89	23.25	22.75	22.69	22.98	21.82	22.65		22.89	22.75	22.23	0.380426	0.149257	0.585994
2844	Q9BT22	Chitobiosyl	ALG1	3	9.1		2.85E+06	3.45E+06	3.42E+06							21.67	22	21.7						21.84	21.7		0.234407		
1437	P08243-2	Asparagine	ASNS	7	16.3	9.78E+06	1.10E+07	1.24E+07	6.77E+06	1.02E+07	1.36E+07	5.58E+06	7.30E+06		23.19	23.58	23.81	22.71	23.45	23.91	22.38	22.56		23.58	23.45	22.47	0.313082	0.602823	0.126669
137	A0A087X2	Calponin	CNN2	5	38.5	2.09E+07	1.98E+07	1.58E+07	1.79E+07	1.93E+07	1.58E+07	1.52E+07			24.27	24.38	24.13	24.14	24.36	24.13	23.83			24.27	24.14	23.83	0.127778	0.131703	
2100	Q06323	Proteasom	PSME1	11	47.4	1.96E+07	3.05E+07	4.40E+07	2.42E+07	2.70E+07	3.11E+07	9.94E+06	1.76E+07	3.06E+06	24.19	25.01	25.61	24.58	24.87	25.16	23.19	23.86	20.98	25.01	24.87	23.19	0.71059	0.291427	1.507474
3177	Q9UQ13-2	Leucine-ric	SHOC2	6	14.4	7.93E+06	6.88E+06	7.10E+06	6.95E+06	4.90E+06	7.47E+06	6.07E+06			22.86	22.9	23.02	22.76	22.42	23.1	22.51			22.9	22.76	22.51	0.086526	0.336836	
3032	Q9NUQ9	Protein FAI	FAM49B	9	34.6	1.48E+07	1.94E+07	1.77E+07	1.27E+07	1.68E+07	1.67E+07	1.22E+07	1.39E+07	1.22E+07	23.77	24.37	24.31	23.61	24.18	24.23	23.44	23.51	23.12	24.31	24.18	23.44	0.33065	0.345052	0.209006
2805	Q99615-2	DnaJ homc	DNAJC7	6	17.6	7.39E+06	7.62E+06	9.77E+06	6.11E+06	6.80E+06	8.69E+06	7.76E+06	5.34E+06		22.74	23.05	23.5	22.58	22.91	23.29	22.83	22.1		23.05	22.91	22.47	0.383694	0.355634	0.52227
1716	P35658-2	Nuclear po	NUP214	19	13.2	3.41E+07	3.77E+07	3.52E+07	2.09E+07	3.34E+07	3.25E+07	1.38E+07	1.96E+07	7.15E+06	25.01	25.36	25.3	24.38	25.16	25.24	23.68	24.04	22.26	25.3	25.16	23.68	0.187687	0.477004	0.939178
194	A0A0A0MT	Integrin-lin	ILK	9	18.8	8.77E+06	9.38E+06	8.99E+06	8.15E+06	8.66E+06	9.12E+06	9.64E+06	1.11E+07	9.48E+06	23.03	23.34	23.37	22.96	23.2	23.37	23.15	23.17	22.71	23.34	23.2	23.15	0.189206	0.205377	0.260589

2594	Q8NEW0	Zinc transp	SLC30A7	3	10.6	2.98E+06	3.04E+06	2.31E+06		2.77E+06	1.81E+06	3.09E+06			21.5	21.78	21.45		21.64	21.09	21.5			21.5	21.36	21.5	0.180086	0.386531	
2633	Q8WUM4	Programm	PDCD6IP	36	50.8	9.13E+07	1.39E+08	8.75E+07	8.24E+07	8.63E+07	8.72E+07	7.18E+07	5.53E+07	3.28E+07	26.54	27.41	26.71	26.43	26.57	26.63	26.03	25.53	24.53	26.71	26.57	25.53	0.46127	0.105045	0.761504
221	A0A0C4DG	Serine prot	HTRA2	4	11.3	4.49E+06	4.29E+06	4.07E+06			3.77E+06	3.84E+06	4.67E+06	3.96E+06	22.03	22.3	22.27			22.13	21.82	21.94	21.26	22.27	22.13	21.82	0.146875		0.366479
112	A0A087X0:	Ribosome	SBDS	10	36.4	1.72E+07	2.40E+07	2.96E+07	2.29E+07	1.74E+07	3.27E+07	8.59E+06	9.49E+06	1.88E+07	24.01	24.66	25.03	24.52	24.22	25.25	22.98	22.95	23.75	24.66	24.52	22.98	0.517313	0.527923	0.455707
2779	Q96S44	TP53-regul	TP53RK	3	19.4		1.98E+06	2.06E+06			1.84E+06					21.23	21.3			21.12				21.27	21.12		0.05579		
629	E7EMM4	Acid ceram	ASAH1	2	5.9		1.91E+06	2.14E+06	2.25E+06					7.02E+06		21.17	21.35	21.12					22.24	21.26	21.12	22.24	0.130345		
767	F5H442	Tumor sus	TSG101	6	21.1	9.18E+06	7.35E+06	6.64E+06	7.41E+06	7.42E+06	6.04E+06	6.47E+06	8.07E+06	5.82E+06	23.1	23	22.93	22.86	23.01	22.79	22.59	22.73	21.92	23	22.86	22.59	0.088388	0.112257	0.429134
2058	Q00653-4	Nuclear fac	NFKB2	7	10.6	5.52E+06	3.50E+06	5.34E+06	4.66E+06	3.85E+06	3.92E+06	3.39E+06	2.80E+06		22.3	22.01	22.68	22.16	22.08	22.19	21.64	21.27		22.3	22.16	21.46	0.333911	0.056744	0.258318
2281	Q15393	Splicing fac	SF3B3	31	30	7.70E+07	5.54E+07	8.67E+07	5.17E+07	6.21E+07	7.28E+07	4.64E+07	4.66E+07	3.22E+07	26.22	25.97	26.7	25.75	26.08	26.39	25.42	25.29	24.48	26.22	26.08	25.29	0.368732	0.315771	0.508165
2479	Q7KZF4	Staphyloco	SND1	31	38.1	6.72E+07	8.00E+07	1.21E+08	8.03E+07	6.91E+07	9.55E+07	8.18E+07	6.29E+07	5.84E+06	25.94	26.5	27.22	26.36	26.22	26.75	26.24	25.71	21.93	26.5	26.36	25.71	0.639018	0.277337	2.351186
2535	Q8IX12-2	Cell divisio	CCAR1	19	16.8	7.14E+07	5.01E+07	4.62E+07	6.28E+07	4.57E+07	3.83E+07	5.94E+07	2.85E+07		26.11	25.75	25.73	25.99	25.61	25.44	25.79	24.58		25.75	25.61	25.19	0.211981	0.281555	0.854388
2810	Q99747	Gamma-so	NAPG	3	12.8		2.90E+06	2.41E+06			2.32E+06			2.51E+06		21.71	21.51			21.47			20.66	21.61	21.47	20.66	0.147216		
2028	P68402	Platelet-ac	PAFAH1B2	2	12.2		3.93E+06	1.14E+07	2.54E+06	6.42E+06	6.72E+06					22.2	23.72	21.33	22.81	22.93				22.96	22.81		1.078188	0.891423	
2401	Q5VSL9	Striatin-int	STRIP1	5	7.5	5.00E+06	3.71E+06	2.47E+06	5.48E+06	3.29E+06	3.29E+06	4.01E+06			22.18	22.1	21.54	22.39	21.85	21.95	21.89			22.1	21.95	21.89	0.348898	0.285949	
1159	O15145	Actin-relat	ARPC3	3	17.4	2.24E+07	3.27E+07	3.46E+07	2.70E+07	2.95E+07	2.81E+07	3.34E+07	2.80E+07	2.05E+07	24.35	25.12	25.24	24.74	24.98	24.99	24.98	24.55	23.9	25.12	24.98	24.55	0.486367	0.138359	0.545109
2498	Q7Z4Q2	HEAT repe	HEATR3	4	7.4		2.24E+06	2.74E+06			2.17E+06					21.36	21.68			21.37				21.52	21.37		0.222945		
2540	Q8IY17-2	Neuropath	PNPLA6	5	5.7	2.49E+06	1.76E+06	2.24E+06	2.19E+06	1.85E+06	2.63E+06	2.13E+06	2.66E+06		21.21	21.04	21.42	21.07	21.06	21.64	20.89	21.19		21.21	21.07	21.04	0.191822	0.332144	0.206112
2996	Q9NPJ3-2	Acyl-coenz	ACOT13	2	18.8	4.62E+06	6.46E+06	7.19E+06	5.53E+06		6.69E+06		4.14E+06		22.07	22.81	23.05	22.4		22.92		21.78		22.81	22.66	21.78	0.511962	0.368293	
2099	Q06210-2	Glutamine-	GFPT1	22	36.9	3.33E+07	3.62E+07	3.86E+07	3.41E+07	2.36E+07	3.20E+07	4.43E+07	3.49E+07	2.72E+07	24.98	25.3	25.46	25.15	24.62	25.19	25.35	24.87	24.21	25.3	25.15	24.87	0.243675	0.320315	0.574054
2377	Q5QPE4	Sorting nex	SNX5	2	11.3	1.48E+06	1.96E+06	1.59E+06	1.72E+06		1.54E+06	1.47E+06	1.68E+06	1.65E+06	20.42	21.21	20.91	20.69		20.84	20.39	20.45	19.72	20.91	20.76	20.39	0.40239	0.104802	0.408756
2903	Q9GZS1-2	DNA-direct	POLR1E	7	15.5	7.17E+06	6.94E+06	7.51E+06	7.07E+06	5.51E+06	7.85E+06	7.33E+06			22.71	22.93	23.13	22.78	22.6	23.17	22.74			22.93	22.78	22.74	0.213648	0.288526	
3152	Q9UKV8-2	Protein arg	AGO2	20	32.7	2.78E+07	2.06E+07	2.06E+07	2.09E+07	2.15E+07	1.25E+07	2.73E+07	2.38E+07	4.93E+06	24.67	24.43	24.53	24.38	24.51	23.79	24.69	24.33	21.67	24.53	24.38	24.33	0.120401	0.3818	1.651267
654	E9PF06	39S riboso	MRPL3	2	11.9		3.05E+06	2.92E+06			2.61E+06					21.79	21.78			21.63				21.78	21.63		0.003777		
974	I3L0E3	28S riboso	hCG_1984:	3	12.9	6.78E+06	6.75E+06	6.32E+06	6.65E+06	7.99E+06	5.75E+06	8.09E+06	8.00E+06		22.62	22.87	22.89	22.69	23.09	22.72	22.89	22.71		22.87	22.72	22.8	0.149543	0.226055	0.131121
1128	O14617	AP-3 comp	AP3D1	22	24.2	3.74E+07	3.83E+07	3.21E+07	2.85E+07	2.94E+07	3.19E+07	3.38E+07	1.53E+07		25.12	25.4	25.12	24.82	24.97	25.18	25	23.66		25.12	24.97	24.33	0.158457	0.184024	0.946748
2953	Q9H6S0	Probable A	YTHDC2	8	5.6	4.83E+06	4.39E+06	2.95E+06	3.10E+06	3.62E+06	3.87E+06	3.34E+06	1.80E+06		22.13	22.32	21.8	21.59	21.98	22.17	21.62	20.6		22.13	21.98	21.11	0.263137	0.296799	0.721618
755	F5GYN4	Ubiquitin t	OTUB1	8	39	1.58E+07	2.50E+07	3.10E+07	2.39E+07	2.27E+07	2.92E+07	1.21E+07	9.48E+06	7.83E+06	23.86	24.73	25.08	24.55	24.58	25.04	23.43	22.95	22.43	24.73	24.58	22.95	0.627222	0.276609	0.504163
1657	P30050	60S riboso	RPL12	7	60	3.11E+08	2.80E+08	2.26E+08	2.22E+08	2.84E+08	2.28E+08	2.58E+08	2.85E+08	1.37E+08	28.22	28.51	28.31	27.77	28.25	28.15	27.92	27.76	26.37	28.31	28.15	27.76	0.146848	0.253524	0.849027
2121	Q08J23	tRNA (cyto	NSUN2	19	33	6.43E+07	5.92E+07	5.51E+07	5.60E+07	6.99E+07	3.58E+07	1.02E+08	7.48E+07	1.02E+08	25.89	26.06	26	25.84	26.24	25.36	26.6	25.95	26.06	26	25.84	26.06	0.087317	0.439242	0.346821
2958	Q9H7D7-2	WD repeat	WDR26	17	26.8	7.56E+07	5.15E+07	5.18E+07	6.52E+07	5.05E+07	4.70E+07	7.14E+07	6.30E+07	5.31E+07	26.19	25.82	25.93	26.04	25.78	25.73	26.01	25.71	25.18	25.93	25.78	25.71	0.18732	0.16965	0.421656
2501	Q7Z6Z7-2	E3 ubiquiti	HUWE1	28	8.9	1.54E+07	2.14E+07	2.81E+07	2.15E+07	1.91E+07	1.71E+07	2.80E+07	2.89E+07	1.35E+07	23.82	24.5	24.94	24.43	24.35	24.27	24.73	24.62	23.29	24.5	24.35	24.62	0.563495	0.080535	0.802416
412	B7ZC39	Endophilin	SH3GLB2	5	12.8		1.80E+06	2.41E+06		1.58E+06	2.32E+06	2.13E+06				21.08	21.51		20.81	21.47	20.89			21.29	21.14	20.89	0.299466	0.460931	
226	A0A0C4DG	Enoyl-CoA	ECI2	4	11.5	5.85E+06	5.23E+06	8.32E+06	5.04E+06	4.70E+06	4.80E+06	6.89E+06	9.27E+06		22.4	22.52	23.27	22.27	22.37	22.45	22.67	22.91		22.52	22.37	22.79	0.472342	0.086709	0.172371
1495	P11940	Polyadenyl	PABPC1	30	51.4	4.04E+08	4.08E+08	4.28E+08	4.24E+08	4.31E+08	5.12E+08	4.48E+08	3.88E+08	1.65E+08	28.64	29.02	29.23	28.65	28.87	29.3	28.69	28.1	26.56	29.02	28.87	28.1	0.298628	0.330346	1.100917
191	D6RDV0	Tumor sup	TUSC3	2	6.9	2.09E+06	1.94E+06	1.77E+06	1.98E+06	1.70E+06	1.64E+06				20.93	21.2	21.11	20.96	20.97	20.93				21.11	20.96		0.135257	0.022581	
2348	Q4G0N4	NAD kinas	NADK2	12	34.4	2.07E+07	2.10E+07	1.93E+07	1.97E+07	1.54E+07	1.70E+07	2.47E+07	2.77E+07	2.14E+07	24.25	24.46	24.42	24.28	24.02	24.26	24.56	24.53	23.96	24.42	24.26	24.53	0.111396	0.146529	0.339659
1421	P07384	Calpain-1 c	CAPN1	9	12.5	6.28E+06	1.17E+07	1.40E+07	7.19E+06	1.09E+07	1.48E+07	1.27E+07	4.59E+06	6.39E+06	22.51	23.68	23.96	22.81	23.52	24.04	23.52	21.91	22.1	23.68	23.52	22.1	0.768033	0.620688	0.879264
173	A0A0A0M:	Multidrug	ABCC1	5	5.8	2.26E+06	2.67E+06	2.67E+06	1.97E+06	2.41E+06	2.35E+06				21.04	21.58	21.64	20.95	21.43	21.47				21.58	21.43		0.334208	0.288549	
1654	P30046	D-dopachr	DDT	6	55.9	9.77E+06	9.76E+06	1.18E+07	8.61E+06	9.01E+06	1.43E+07	8.96E+06			23.19	23.4	23.75	23.03	23.24	23.98	23.04			23.4	23.24	23.04	0.282282	0.50181	
2913	Q9H0D6-2	5-3 exoribo	XRN2	5	9.3	2.80E+06	4.18E+06	4.07E+06	4.06E+06	3.97E+06	4.85E+06	3.60E+06	4.45E+06	4.19E+06	21.37	22.27	22.27	21.96	22.11	22.46	21.72	21.88	21.34	22.27	22.11	21.72	0.522264	0.259206	0.277105
641	H7C1W2	Isocitrate c	IDH3G	3	25.6	3.23E+06		3.99E+06	3.43E+06		3.04E+06				21.6		22.25	21.71		21.83				21.93	21.77		0.461885	0.08457	
1391	P04844	Dolichyl-di	RPN2	15	34.9	6.37E+07	5.64E+07	7.06E+07	5.22E+07	5.30E+07	5.81E+07	5.91E+07	5.14E+07		25.87	26	26.43	25.76	25.84	26.01	25.78	25.42		26	25.84	25.6	0.294032	0.124588	0.25661
2197	Q13868-3	Exosome c	EXOSC2	3	14.4	6.35E+06	5.99E+06	5.33E+06	6.10E+06	4.99E+06		3.98E+06			22.53	22.68	22.68	22.57	22.46		21.87			22.68	22.51	21.87	0.08551	0.084133	
2782	Q96S59	Ran-bindin	RANBP9	16	28.9	7.69E+07	5.62E+07	5.14E+07	8.18E+07	5.10E+07	5.09E+07	8.30E+07	7.54E+07	7.13E+07	26.22	25.99	25.92	26.41	25.79	25.83	26.29	25.98	25.52	25.99	25.83	25.98	0.152868	0.345704	0.386558
1969	P62081	40S riboso	RPS7	13	61.9	4.97E+07	9.27E+07	1.71E+08	4.97E+07	8.51E+07	8.41E+07	8.63E+07	5.14E+07	2.61E+07	25.54	26.71	27.79	25.72	26.55	26.59	26.35	25.42	24.16	26.71	26.55	25.42	1.124298	0.48891	1.102398
3030	Q9NUQ3-2	Gamma-ta	TXLNG	3	7.1		2.52E+06	1.98E+06	1.74E+06	2.12E+06	2.30E+06	1.77E+06				21.53	21.25	20.72	21.23	21.46	20.65			21.39	21.23	20.65	0.194517	0.379583	
2801	Q99567	Nuclear po	NUP88	14	27.9	3.50E+07	3.01E+07	3.30E+07	3.03E+07	3.56E+07	2.68E+07	2.20E+07	2.21E+07	8.22E+06	25.04	24.98	25.15	24.87	25.25	24.88	24.4	24.21	22.49	25.04	24.88	24.21	0.083008	0.219106	1.05528
1905	P53618	Coatomer	COPB1	29	36.7	5.36E+07	7.07E+07	5.86E+07	4.05E+07	5.59E+07	5.82E+07	2.77E+07	6.05E+07	5.01E+07	25.65	26.33	26.08	25.38	25.91	26.01	24.71	25.65	25.1	26.08	25.91	25.1	0.346828	0.341671	0.47494
140	A0A087X2I	39S riboso	MRPL45	2	9.2		3.79E+06	4.48E+06			3.67E+06	3.70E+06				22.14	22.41			22.11	21.78			22.27	22.11	21.78	0.185872		
2317	Q16576	Histone-bi	RBBP7	14	40.9	6.93E+07	1.01E+08	9.05E+07	8.12E+07	8.85E+07	1.09E+08	6.60E+07	9.29E+07	6.11E+07	26	26.82	26.78	26.37	26.62	27.03	25.89	26.27	25.31	26.78	26.62	25.89	0.462304	0.337995	0.481357
3256	Q9Y678	Coatomer	COPG1	20	30.5	6.25E+07	7.46E+07	3.64E+07	3.87E+07	4.97E+07	4.45E+07	1.07E+08	6.31E+07	1.15E+08	25.83	26.41	25.35	25.3	25.74	25.66	26.71	25.72	26.19	25.83	25.66	26.19	0.531541	0.232857	0.495537
83	H7BXI1	Extended s	ESYT2	12	17.2	3.97E+07	4.08E+07	2.66E+07	3.26E+07	6.39E+07	2.32E+07	3.88E+07	2.58E+07	4.64E+06	25.23	25.49	24.88	25.06	26.15	24.68	25.22	24.44	21.54	25.23	25.06	24.44	0.304767	0.760999	1.935341
2830	Q9BRP4	Proteasom	PAAF1	3	7.9	3.67E+06	3.36E+06	2.73E+06	3.00E+06		2.69E+06				21.77	21.95	21.67	21.54		21.67				21.77	21.61		0.141931	0.091231	
2190	Q13642-1	Four and a	FHL1	5	23.6	5.41E+06	3.67E+06	2.84E+06	5.05E+06	3.40E+06	2.93E+06	7.80E+06	6.05E+06		22.28	22.07	21.75	22.28	21.9	21.79	22.84	22.29		22.07	21.9	22.57	0.267869	0.25311	0.389401
1887	P52306-4	Rap1 GTPa	RAP1GDS1	5	10.5	3.18E+06	2.86E+06	3.09E+06		2.38E+06	2.58E+06	2.92E+06	2.44E+06		21.56	21.68	21.87		21.41	21.62	21.43	21.04		21.68	21.51	21.24	0.156288	0.147998	0.275852
377	B4DJV5	Periodic tr	PWP1	6	17.5	4.06E+06	6.35E+06	5.22E+06		3.63E+06	6.69E+06	3.65E+06	3.84E+06		21.91	22.78	22.62		21.99	22.92	21.73	21.69		22.62	22.46	21.71	0.462936	0.661764	0.031787
2768	Q96PU8-5	Protein qu	QKI	3	11.4	2.94E+06	2.94E+06	3.28E+06		2.73E+06	2.42E+06				21.47	21.73	21.95		21.62	21.5				21.73	21.56		0.237169	0.082669	
1761	P41240	Tyrosine-p	CSK	4	10.4	3.82E+06	3.74E+06	3.68E+06	4.41E+06	3.11E+06	3.29E+06	4.77E+06	4.40E+06	4.68E+06	21.84	22.12	22.15	22.08	21.78	21.95	22.13	21.85	21.56	22.12	21.95	21.85	0.171185	0.149414	0.283217
1302	O94905	Erlin-2	ERLIN2	8	25.4	4.02E+07	2.54E+07	2.81E+07	2.75E+07	2.69E+07	2.27E+07	2.80E+07	1.44E+07	1.32E+07	25.25	24.77	24.94	24.77	24.86	24.65	24.73	23.58	23.26	24.94	24.77	23.58	0.240943	0.108101	0.772528
1119	O00560-3	Syntenin-1	SDCBP	2	9.9	1.31E+06	1.51E+06	1.43E+06	1.33E+06	1.34E+06	1.36E+06	1.53E+06	1.60E+06		20.25	20.83	20.78	20.38	20.61	20.7	20.46	20.35		20.78	20.61	20.4	0.325033	0.167555	0.07728
2565	Q8N5W9	Filamin-int	FAM101B	4	34.6	1.90E+07	1.51E+07	1.70E+07	1.60E+07	1.56E+07	1.23E+07				24.16	24.02	24.25	23.99	24.05	23.75				24.16	23.99		0.115405	0.157237	
2203	Q14103-3	Heterogen	HNRNPD	15	40.5	2.05E+08	2.15E+08	1.99E+08	2.06E+08	3.56E+08	1.87E+08	1.02E+08	1.68E+08	1.24E+08	27.76	28.11	28.1	27.69	28.5	27.93	26.6	27.09	26.26	28.1	27.93	26.6	0.201382	0.413903	0.416341
2986	Q9HCE1	Putative he	MOV10	19	21	1.87E+07	1.77E+07	1.63E+07	1.89E+07	1.53E+07	1.20E+07	2.11E+07	2.34E+07		24.14	24.26	24.18	24.22	24.01	23.69	24.34	24.3		24.18	24.01	24.32	0.058441	0.263321	0.031471
2108	Q07864	DNA polym	POLE	15	8.3	5.75E+06	6.03E+06	6.09E+06	5.93E+06	5.39E+06	5.03E+06	6.20E+06	5.40E+06		22.37	22.69	22.84	22.51	22.57	22.52	22.53	22.12		22.69	22.52	22.33	0.23948	0.031819	0.291881
30	A0A087WU	DNA polym	POLA1	13	13.3	1.06E+07	1.18E+07	1.20E+07	9.10E+06	1.19E+07	1.03E+07	1.58E+07	1.72E+07	1.09E+07	23.31	23.69	23.77	23.13	23.64	23.52	23.86	23.82	22.96	23.69	23.52	23.82	0.244234	0.269847	0.509134
712	E9PKG1	Protein arg	PRMT1	19	57.8	2.38E+08	2.10E+08	2.32E+08	2.34E+08	2.10E+08	2.41E+08	2.51E+08	2.84E+08	1.78E+08	27.93	28.05	28.38	27.88	27.78	28.29	27.89	27.73	26.64	28.05	27.88	27.73	0.229997	0.269618	0.679566
1408	P06493	Cyclin-depe	CDK1	26	75.4	5.42E+09	4.83E+09	3.97E+09	7.69E+09	4.78E+09	5.00E+09	6.67E+09	7.84E+09	6.64E+09	33.03	33.11	32.99	33.18	32.15	32.86	32.65	32.33	31.36	33.03	32.86	32.33	0.06095	0.527135	0.673671
2085	Q03169	Tumor nec	TNFAIP2	4	6.7		3.13E+06	3.02E+06	2.57E+06	3.60E+06		3.48E+06				21.83	21.83	21.34	21.97		21.67			21.83	21.66	21.67	0.003182	0.444255	
765	F5H365	Protein tra	SEC23A	11	20	1.60E+07	2.02E+07	2.37E+07	1.43E+07	1.75E+07	2.07E+07	1.83E+07	1.62E+07	1.20E+07	23.89	24.4	24.75	23.8	24.23	24.53	24.1	23.75	23.1	24.4	24.23	23.75	0.431079	0.369875	0.507687
1273	O75592-2	E3 ubiquiti	MYCBP2	19	5	1.04E+07	1.30E+07	1.02E+07	1.45E+07	9.12E+06	9.14E+06	8.02E+06	8.85E+06	4.91E+06	23.29	23.82	23.55	23.82	23.26	23.37	22.87	22.85	21.66	23.55	23.37	22.85	0.264864	0.2949	0.69223
702	E9PJ81	UBX doma	UBXN1	5	31.4	7.88E+06	8.52E+06	8.25E+06		8.95E+06	6.10E+06	8.52E+06	7.46E+06		22.83	23.2	23.26		23.24	22.81	22.96	22.6		23.2	23.02	22.78	0.231138	0.30491	0.25265
1645	P29373	Cellular ret	CRABP2	6	39.9	1.27E+07	1.49E+07	1.69E+07			1.28E+07		5.57E+06		23.57	24	24.24			23.82		22.19		24	23.82	22.19	0.337521		
2607	Q8TC07-2	TBC1 doma	TBC1D15	3	5	4.08E+06	2.77E+06	3.14E+06	3.29E+06	3.84E+06	2.76E+06	3.89E+06	3.82E+06	4.57E+06	21.92	21.63	21.89	21.67	22.07	21.72	21.85	21.68	21.53	21.89	21.72	21.68	0.162428	0.220203	0.159665
277	A0A0U1RC	Forkhead b	FOXP2	8	21	1.36E+07	1.26E+07	1.11E+07	1.19E+07	1.46E+07	9.04E+06	2.00E+07	1.78E+07	6.46E+06	23.64	23.78	23.68	23.5	23.94	23.36	24.24	23.87	22.13	23.68	23.5	23.87	0.070781	0.306106	1.1286
2347	Q4G0J3	La-related	LARP7	6	14.4	4.84E+06	2.39E+06	2.95E+06	2.32E+06	3.09E+06	2.58E+06				22.14	21.45	21.8	21.16	21.78	21.62				21.8	21.62		0.344086	0.319264	
2186	Q13616	Cullin-1	CUL1	17	25.3	1.23E+07	1.68E+07	1.63E+07	1.33E+07	2.05E+07	1.44E+07	1.86E+07	1.68E+07	9.41E+06	23.52	24.18	24.18	23.69	24.46	24	24.12	23.79	22.7	24.18	24	23.79	0.37772	0.389227	0.745852
1099	O00186	Syntaxin-bi	STXBP3	4	7.4	3.56E+06	2.81E+06	3.06E+06	2.37E+06	3.00E+06	2.47E+06	2.65E+06	2.34E+06		21.72	21.64	21.86	21.2	21.74	21.54	21.25	21		21.72	21.54	21.12	0.107377	0.271178	0.176732

2495	Q7Z3U7-2	Protein MC	MON2	11	7.9	9.14E+06	9.49E+06	9.35E+06	7.98E+06	8.62E+06	7.95E+06	9.11E+06	1.47E+07		23.09	23.37	23.43	22.93	23.19	23.19	23.06	23.62		23.37	23.19	23.34	0.18257	0.152363	0.391956
2834	Q9BRX2	Protein pel	PELO	6	16.9	5.04E+06	4.87E+06	4.29E+06	4.56E+06	4.20E+06	4.11E+06	4.88E+06			22.19	22.45	22.35	22.12	22.17	22.26	22.16			22.35	22.17	22.16	0.130983	0.065999	
3162	Q9UMZ2-6	Synergin ga	SYNRG	5	7.9	3.06E+06	3.17E+06	3.51E+06	2.85E+06	3.05E+06	2.70E+06	3.65E+06			21.53	21.86	22.04	21.48	21.76	21.68	21.73			21.86	21.68	21.73	0.258779	0.14329	
2215	Q14232	Translatior	EIF2B1	8	26.6	9.09E+06	9.80E+06	9.25E+06	8.06E+06	8.83E+06	9.32E+06	7.68E+06	9.57E+06	7.19E+06	23.08	23.4	23.41	22.95	23.22	23.4	22.8	22.97	22.27	23.4	23.22	22.8	0.188918	0.230147	0.36072
249	A0A0U1RC	Dynamin-1	DNM1	4	11.9	9.96E+05		1.69E+06	7.49E+05	9.66E+05	1.24E+06				19.74		21.04	19.83	20.21	20.62				20.39	20.21		0.915684	0.397164	
3184	Q9Y263	Phospholip	PLAA	9	13.6	8.07E+06	8.97E+06	7.92E+06	8.46E+06	8.31E+06	7.09E+06	8.05E+06	5.51E+06		22.89	23.27	23.2	23	23.14	23.02	22.88	22.17		23.2	23.02	22.52	0.200004	0.075503	0.503075
300	A3KMH1-3	von Willeb	VWA8	15	10.1	1.39E+07	9.50E+06	9.79E+06	1.38E+07	9.21E+06	8.92E+06	1.37E+07	1.17E+07		23.67	23.37	23.51	23.74	23.29	23.32	23.66	23.26		23.51	23.32	23.46	0.150068	0.252866	0.28173
2987	Q9HCN4	GPN-loop (	GPN1	3	12		3.73E+06	2.96E+06	3.19E+06	3.53E+06	2.87E+06	7.51E+06	4.76E+06	4.71E+06		22.11	21.8	21.63	21.95	21.77	22.76	21.97	21.57	21.96	21.77	21.97	0.218654	0.157787	0.605137
1469	P10515	Dihydrolipo	DLAT	13	29.4	4.84E+07	5.45E+07	4.42E+07	4.21E+07	4.88E+07	3.15E+07	5.74E+07	4.15E+07	1.30E+07	25.49	25.92	25.63	25.44	25.71	25.17	25.73	25.1	23.22	25.63	25.44	25.1	0.220377	0.269332	1.301984
1659	Q5T0D2	UMP-CMP	CMPK1	2	12.4	2.09E+06	2.05E+06	6.94E+05		1.49E+06					20.93	21.27	19.33		20.75					20.93	20.75		1.037084		
3155	Q9ULC4-2	Malignant	MCTS1	4	29.6	6.20E+06	6.81E+06	6.12E+06	6.44E+06	4.84E+06	7.19E+06	5.05E+06		6.14E+06	22.49	22.88	22.84	22.66	22.41	23.03	22.22		22.02	22.84	22.66	22.12	0.212905	0.313524	0.140643
1867	P51116	Fragile X m	FXR2	4	15.3	1.21E+07	8.91E+06	7.48E+06	8.83E+06	8.59E+06	6.98E+06	9.91E+06	9.80E+06		23.5	23.25	23.13	23.07	23.19	23	23.18	23.01		23.25	23.07	23.09	0.189888	0.094626	0.121997
1633	P27694	Replicatior	RPA1	13	30.8	1.66E+07	1.76E+07	2.29E+07	1.59E+07	1.57E+07	2.17E+07	2.39E+07	2.11E+07		23.95	24.25	24.68	23.97	24.06	24.58	24.5	24.15		24.25	24.06	24.33	0.370441	0.331875	0.24775
2966	Q9H8S9	MOB kinas	MOB1A	5	23.1	1.08E+08	2.96E+07		4.89E+07	9.02E+07	1.94E+07	3.54E+07	3.82E+07	1.40E+08	26.8	24.96		25.69	26.65	24.45	25.09	25	26.39	25.88	25.69	25.09	1.299115	1.101929	0.780066
1455	P09497-2	Clathrin lig	CLTB	3	12.3	2.16E+06	2.13E+06		1.84E+06		1.83E+06				20.98	21.32		20.82		21.11				21.15	20.96		0.24523	0.211649	
1066	K9J957	Proteasom	PSME3	8	39	2.31E+07	2.29E+07	2.01E+07	1.97E+07	1.95E+07	1.72E+07	1.64E+07	1.67E+07	1.59E+07	24.41	24.6	24.47	24.28	24.38	24.28	23.94	23.78	23.55	24.47	24.28	23.78	0.098485	0.059515	0.193578
1174	O15381-5	Nuclear va	NVL	6	9.5	1.97E+06	3.72E+06	1.72E+06	1.59E+06	2.10E+06					20.84	22.11	21.07	20.56	21.21					21.07	20.89		0.674566	0.462541	
1914	P54619-2	5-AMP-act	PRKAG1	7	25.4	1.52E+07	1.28E+07	1.19E+07	1.20E+07	1.16E+07	1.26E+07	9.87E+06	1.55E+07	1.48E+07	23.8	23.8	23.75	23.52	23.61	23.81	23.17	23.68	23.43	23.8	23.61	23.43	0.025952	0.144777	0.256039
2258	Q15046	Lysine--tRN	KARS	31	50.8	6.32E+08	3.50E+08	4.28E+08	4.90E+08	4.73E+08	5.54E+08	3.24E+08	3.56E+08	2.74E+08	29.31	28.82	29.23	28.83	29.04	29.37	28.17	27.98	27.2	29.23	29.04	27.98	0.262589	0.274967	0.511428
1924	P55263-3	Adenosine	ADK	3	5.6		2.84E+06	4.41E+06			3.05E+06	2.56E+06				21.66	22.38			21.83	21.21			22.02	21.83	21.21	0.509313		
3244	Q9Y5K6	CD2-associ	CD2AP	5	10.8	6.74E+06	8.58E+06	9.56E+06	7.93E+06	7.43E+06	7.26E+06	5.55E+06	7.15E+06		22.61	23.2	23.47	22.92	23.01	23.05	22.37	22.53		23.2	23.01	22.45	0.439082	0.068353	0.112953
414	B7ZM99	Monofunct	MTHFD1L	22	26.8	8.57E+07	6.04E+07	6.40E+07	7.26E+07	6.01E+07	5.01E+07	7.93E+07	7.73E+07	6.63E+07	26.37	26.09	26.22	26.21	26.03	25.78	26.19	26.01	25.44	26.22	26.03	26.01	0.141952	0.216012	0.3884
2995	Q9NPF4	Probable tl	OSGEP	3	13.4	3.13E+06	3.34E+06	2.15E+06	2.53E+06	2.38E+06		2.37E+06			21.56	21.94	21.36	21.32	21.41		21.11			21.56	21.36	21.11	0.296264	0.063718	
2228	Q14574-2	Desmocolli	DSC3	4	7.4	1.72E+06	1.89E+06	1.63E+07			1.68E+06			7.43E+06	20.6	21.15	24.18			20.96			22.33	21.15	20.96	22.33	1.92885		
1835	P49642	DNA prima	PRIM1	4	11.7	2.44E+06	3.47E+06	2.81E+06	3.17E+06	2.31E+06	2.46E+06	2.36E+06	2.65E+06		21.18	21.99	21.73	21.62	21.36	21.53	21.1	21.17		21.73	21.53	21.14	0.416858	0.134238	0.051124
14	E2QRB9	Thioredoxi	TXNRD1	3	12.6	5.73E+06	6.03E+06	5.33E+06		4.76E+06	5.21E+06				22.37	22.69	22.68		22.39	22.58				22.68	22.48		0.1831	0.134166	
2378	Q5QPL9	RNA-bindir	RALY	5	25.3	7.37E+06	6.11E+06	5.30E+06	5.96E+06	3.93E+06	5.38E+06	4.39E+06	5.58E+06	4.24E+06	22.73	22.72	22.67	22.53	22.1	22.63	22.03	22.19	21.35	22.72	22.53	22.03	0.033979	0.27841	0.444776
2087	Q04446	1,4-alpha-g	GBE1	8	15.4	2.26E+07	1.69E+07	2.00E+07	1.56E+07	1.68E+07	2.05E+07	1.77E+07	1.73E+07		24.37	24.2	24.46	23.93	24.18	24.51	24.06	23.83		24.37	24.18	23.95	0.132926	0.295122	0.15792
2940	Q9H3U1-2	Protein un	UNC45A	17	20.8	1.02E+07	2.21E+07	1.21E+07	1.25E+07	1.07E+07	1.13E+07	2.02E+07	1.16E+07	1.50E+07	23.27	24.56	23.78	23.58	23.51	23.61	24.28	23.24	23.45	23.78	23.58	23.45	0.653993	0.053243	0.547886
2695	Q969X5-2	Endoplasm	ERGIC1	6	29.8	1.21E+07	1.14E+07	1.07E+07	1.31E+07	9.79E+06	9.46E+06	1.47E+07	1.46E+07		23.5	23.63	23.61	23.67	23.37	23.41	23.78	23.61		23.61	23.41	23.69	0.06996	0.162331	0.121566
2675	Q92820	Gamma-glu	GGH	5	15.7	1.28E+07	1.47E+07	1.61E+07	1.20E+07	1.39E+07	1.24E+07	1.38E+07	1.41E+07	1.43E+07	23.58	23.97	24.16	23.52	23.88	23.78	23.68	23.53	23.38	23.97	23.78	23.53	0.295763	0.184098	0.148235
3259	Q9Y6A4	Cilia- and fl	CFAP20	2	10.4	7.05E+06	4.94E+06			4.74E+06			4.06E+06	7.08E+06	22.68	22.48			22.38			21.77	22.25	22.58	22.38	22.01	0.143926		0.341409
2112	Q08211	ATP-depen	DHX9	20	20	2.13E+07	3.10E+07	3.63E+07	1.96E+07	2.65E+07	3.19E+07	2.08E+07	2.44E+07		24.3	25.02	25.34	24.27	24.83	25.18	24.33	24.36		25.02	24.83	24.34	0.532786	0.459267	0.027875
3085	Q9P258	Protein RC	RCC2	16	39.7	4.34E+07	4.10E+07	4.23E+07	3.15E+07	3.67E+07	5.29E+07	3.35E+07	3.25E+07	6.94E+06	25.37	25.5	25.56	24.97	25.3	25.89	24.98	24.78	22.22	25.5	25.3	24.78	0.09564	0.466529	1.540416
2221	Q5QPP3	UDP-gluco	GALE	4	22.5	2.51E+06	2.86E+06	4.15E+06		2.18E+06	2.73E+06				21.23	21.68	22.3		21.27	21.7				21.68	21.48		0.539643	0.306779	
160	A0A0A0MF	Heterogen	HNRNPUL1	9	14.6	3.06E+07	2.40E+07	2.28E+07	2.57E+07	1.96E+07	1.98E+07	3.31E+07	1.98E+07	2.48E+07	24.86	24.66	24.68	24.69	24.39	24.48	24.96	24.07	24.11	24.68	24.48	24.11	0.112218	0.15036	0.503725
1964	P61962	DDB1- and	DCAF7	4	15.5	9.98E+06	4.78E+06	5.09E+06	5.45E+06	5.30E+06	4.07E+06	5.88E+06	9.12E+06		23.23	22.43	22.58	22.38	22.55	22.24	22.46	22.89		22.58	22.38	22.67	0.420807	0.15248	0.299633
2292	Q15637-4	Splicing fac	SF1	3	7.5	1.83E+06	1.90E+06	2.14E+06	1.70E+06	2.59E+06	1.68E+06				20.74	21.16	21.35	20.67	21.51	20.96				21.16	20.96		0.310278	0.427701	
598	D6RCD0	Estradiol 1	HSD17B11	3	16	2.85E+06	2.30E+06	1.82E+06	2.30E+06	3.20E+06	1.91E+06	3.17E+06	3.13E+06		21.41	21.4	21.15	21.15	21.82	21.2	21.56	21.42		21.4	21.2	21.49	0.14195	0.375004	0.094896
2617	Q8TDN6	Ribosome	BRIX1	4	12.5	7.66E+06	1.20E+07	1.24E+07	9.26E+06	1.14E+07	1.03E+07	8.99E+06	7.06E+06		22.79	23.72	23.81	23.15	23.59	23.52	23.05	22.51		23.72	23.52	22.78	0.561593	0.232537	0.375517
1536	P16403	Histone H1	HIST1H1C	5	23.5	6.80E+06	9.82E+06	1.03E+07	3.16E+06	8.68E+06	1.42E+07	1.33E+07			22.63	23.41	23.56	21.62	23.21	23.97	23.59			23.41	23.21	23.59	0.499497	1.200954	
3223	Q9Y3T9	Nucleolar c	NOC2L	7	9.3	4.15E+06	6.61E+06	6.14E+06	6.23E+06	9.82E+06	5.41E+06	4.53E+06			21.94	22.83	22.85	22.6	23.38	22.63	22.08			22.83	22.63	22.08	0.518346	0.438488	
2817	Q9BPW8	Protein Nip	NIPSNAP1	3	16.5		3.79E+06	3.85E+06		3.14E+06	3.84E+06					22.14	22.21		21.8	22.16				22.18	21.98		0.048957	0.252173	
2677	Q92878	DNA repair	RAD50	33	25.4	7.35E+07	6.62E+07	6.62E+07	8.14E+07	5.85E+07	5.87E+07	5.40E+07	4.40E+07	3.42E+07	26.14	26.23	26.28	26.38	25.98	26.02	25.65	25.18	24.6	26.23	26.02	25.18	0.070939	0.215856	0.528035
1611	P25311	Zinc-alpha-	AZGP1	4	17.1		1.21E+06	5.39E+06	5.93E+06	2.40E+06	2.00E+06	3.14E+06	5.88E+06			20.55	22.69	22.51	21.42	21.26	21.54	22.25		21.62	21.42	21.9	1.513219	0.682451	0.501566
2879	Q9BXY0	Protein MA	MAK16	2	6.7	1.82E+06	1.93E+06	1.63E+06	1.74E+06	1.47E+06	1.75E+06	1.48E+06	1.60E+06		20.72	21.19	20.94	20.72	20.74	21.02	20.4	20.35		20.94	20.74	20.38	0.237493	0.171777	0.038661
2791	Q96T76	MMS19 nu	MMS19	13	17.1	1.09E+07	1.18E+07	1.07E+07	9.23E+06	1.01E+07	9.34E+06	5.04E+06	7.56E+06	6.35E+06	23.36	23.69	23.61	23.14	23.43	23.41	22.22	22.63	22.1	23.61	23.41	22.22	0.17259	0.158398	0.280318
1951	R4GMT0	Alpha-cent	ACTR1A	9	46.4	8.81E+07	9.40E+07	7.38E+07	7.14E+07	7.94E+07	7.00E+07	1.18E+08	1.27E+08	6.20E+07	26.47	26.74	26.51	26.18	26.42	26.31	26.83	26.71	25.34	26.51	26.31	26.71	0.14306	0.122004	0.826201
1572	P20618	Proteasom	PSMB1	3	13.7	5.86E+06	8.01E+06	3.70E+06	3.52E+06	4.26E+06	7.87E+06	3.45E+06	3.29E+06		22.4	23.11	22.15	21.74	22.2	23.17	21.66	21.48		22.4	22.2	21.57	0.495347	0.734385	0.126117
2650	Q8WXX5	DnaJ homo	DNAJC9	5	23.5	7.72E+06	7.55E+06	6.63E+06	4.84E+06	6.02E+06	7.52E+06	5.17E+06	6.06E+06	4.73E+06	22.81	23.04	22.92	22.21	22.72	23.11	22.26	22.3	21.59	22.92	22.72	22.26	0.114404	0.452352	0.399361
2057	Q00587-2	Cdc42 effe	CDC42EP1	2	7		1.07E+06			9.12E+05	9.18E+05					20.4			20.09	20.29				20.4	20.19			0.139734	
2891	Q9BZH6	WD repeat	WDR11	5	6.1	2.05E+06	2.79E+06	2.40E+06	2.10E+06	2.22E+06	2.48E+06	2.19E+06			20.89	21.64	21.49	21.01	21.29	21.55	20.95			21.49	21.29	20.95	0.396336	0.269805	
3262	Q9Y6D6	Brefeldin A	ARFGEF1	9	5.9	6.64E+06	4.84E+06	3.71E+06	4.20E+06	4.36E+06	4.05E+06	6.06E+06	4.71E+06	4.54E+06	22.59	22.44	22.16	22.02	22.25	22.24	22.51	21.96	21.5	22.44	22.24	21.96	0.221788	0.130632	0.505658
2122	Q09028-3	Histone-bir	RBBP4	8	45.6	2.05E+07	3.25E+07	3.19E+07	2.40E+07	2.76E+07	3.03E+07	1.86E+07	2.73E+07	1.55E+07	24.24	25.11	25.1	24.56	24.9	25.1	24.12	24.5	23.51	25.1	24.9	24.12	0.499973	0.27179	0.503495
124	A0A087X0	Heterogen	HNRNPM	19	34.7	1.36E+08	1.58E+08	1.70E+08	1.23E+08	1.66E+08	1.52E+08	1.50E+08	1.08E+08	3.23E+07	27.04	27.64	27.77	27.01	27.43	27.64	27.17	26.44	24.5	27.64	27.43	26.44	0.391281	0.32105	1.377948
2671	Q92747	Actin-relat	ARPC1A	3	8.9	3.40E+06	4.66E+06	2.95E+06	3.47E+06	2.61E+06	2.54E+06	3.63E+06	3.19E+06	2.62E+06	21.66	22.4	21.8	21.72	21.54	21.59	21.72	21.45	20.77	21.8	21.59	21.45	0.390553	0.091878	0.494162
811	F8W1A4	Adenylate	AK2	6	30.6	1.16E+07	1.51E+07	1.61E+07	1.44E+07	1.04E+07	1.42E+07	1.86E+07	1.18E+07	1.36E+07	23.45	24.02	24.16	23.81	23.47	23.97	24.12	23.28	23.29	24.02	23.81	23.29	0.375633	0.2575	0.479313
2911	Q9H0B6	Kinesin ligh	KLC2	14	41.5	2.93E+07	2.11E+07	1.89E+07	2.13E+07	1.80E+07	1.60E+07	2.11E+07	1.85E+07	1.63E+07	24.78	24.47	24.39	24.41	24.26	24.16	24.34	23.95	23.59	24.47	24.26	23.95	0.204134	0.128303	0.374847
1402	P05388	60S acidic r	RPLP0	15	49.5	6.21E+08	5.34E+08	4.70E+08	4.99E+08	5.07E+08	4.68E+08	5.25E+08	6.48E+08	3.56E+08	29.27	29.35	29.38	28.84	29.14	29.15	28.96	28.82	27.57	29.35	29.14	28.82	0.05751	0.174882	0.764891
2161	S4R3G0	Serine/argi	SRSF9	3	26.9	4.15E+06	3.94E+06	2.88E+06	3.51E+06	3.05E+06	2.72E+06				21.94	22.2	21.76	21.73	21.76	21.69				21.94	21.73		0.221419	0.036913	
198	A0A0A0MT	Glucose-6-	GPI	9	23.6	6.72E+06	1.78E+07	2.84E+07	7.74E+06	1.56E+07	1.99E+07	1.23E+07			22.6	24.26	24.96	22.9	24.05	24.49	23.46			24.26	24.05	23.46	1.209432	0.819453	
3019	Q9NSE4	Isoleucine-	IARS2	15	17	2.90E+07	2.12E+07	2.33E+07	2.01E+07	2.13E+07	2.65E+07	1.03E+07	1.10E+07	1.09E+07	24.76	24.48	24.71	24.31	24.5	24.86	23.23	23.15	22.96	24.71	24.5	23.15	0.146325	0.276154	0.14001
2155	Q13155	Aminoacyl	AIMP2	12	55.9	3.79E+08	2.11E+08	2.17E+08	2.32E+08	2.39E+08	2.90E+08	9.23E+07	2.22E+08	1.58E+08	28.58	28.07	28.22	27.85	28.01	28.56	26.47	27.49	26.51	28.22	28.01	26.51	0.264077	0.373409	0.577507
2758	Q96L92-3	Sorting nex	SNX27	4	9.1		2.10E+06	2.27E+06			1.86E+06					21.31	21.43			21.16				21.37	21.16		0.088838		
1678	P31327	Carbamoyl	CPS1	82	65.4	1.40E+09	1.01E+09	1.53E+09	1.05E+09	1.20E+09	1.34E+09	1.23E+09	1.32E+09	1.12E+09	30.73	30.57	31.37	30.19	30.52	30.68	30.34	30.22	29.4	30.73	30.52	30.22	0.421947	0.251661	0.512193
2080	Q02809	Procollage	PLOD1	12	21.3	3.09E+07	3.74E+07	4.03E+07	2.90E+07	3.44E+07	3.08E+07	3.45E+07	2.23E+07	4.89E+06	24.89	25.35	25.52	24.84	25.2	25.14	25.03	24.23	21.65	25.35	25.14	24.23	0.328237	0.192241	1.770388
1049	K7ELL7	Glucosidas	PRKCSH	9	20.2	4.11E+07	3.19E+07	5.87E+07	3.21E+07	3.15E+07	4.07E+07	4.45E+07	3.38E+07	2.68E+07	25.27	25.07	26.08	25.03	25.06	25.54	25.36	24.82	24.2	25.27	25.06	24.82	0.532948	0.287153	0.582091
1618	P25789	Proteasom	PSMA4	20	68.2	4.95E+09	3.93E+09	3.51E+09	6.54E+09	4.78E+09	4.35E+09	9.52E+09	1.05E+10	1.00E+10	32.92	32.96	32.83	32.82	32.15	32.71	33.14	32.99	32.28	32.92	32.71	32.99	0.068089	0.359321	0.461612
1725	P36507	Dual specif	MAP2K2	11	31	2.37E+07	2.52E+07	1.91E+07	1.94E+07	1.62E+07	1.90E+07	1.18E+07	1.12E+07		24.46	24.75	24.4	24.25	24.11	24.43	23.39	23.19		24.46	24.25	23.29	0.184439	0.162541	0.14496
2717	Q96CN7	Isochorism	ISOC1	3	12.1	4.93E+06	5.70E+06	4.75E+06			4.16E+06				22.16	22.63	22.49			22.28				22.49	22.28		0.240442		
2353	Q53GQ0	Very-long-	HSD17B12	5	21.8	7.62E+06	1.76E+07	2.25E+07	1.11E+07	2.55E+07	1.47E+07	1.59E+07			22.79	24.25	24.66	23.39	24.78	24.03	23.87			24.25	24.03	23.87	0.986321	0.694779	
1283	O75821	Eukaryotic	EIF3G	13	32.2	5.88E+07	8.97E+07	7.07E+07	7.32E+07	8.65E+07	6.28E+07	7.46E+07	8.46E+07		25.78	26.66	26.44	26.22	26.58	26.14	26.09	26.15		26.44	26.22	26.12	0.457012	0.234823	0.043847
2322	Q16659	Mitogen-ac	MAPK6	23	40.2	1.85E+08	1.20E+08	1.30E+08	1.34E+08	1.33E+08	1.07E+08	2.37E+08	2.66E+08	1.35E+08	27.59	27.18	27.35	27.2	27.13	27	27.81	27.63	26.35	27.35	27.13	27.63	0.204371	0.097781	0.79648
1569	P20290-2	Transcripti	BTF3	3	35.8	1.69E+07	1.47E+07	2.59E+07		1.43E+07	1.10E+07				23.97	23.97	24.85		23.92	23.59				23.97	23.75		0.509104	0.230775	
727	E9PNW4	CD59 glyco	CD59	2	22.2			2.02E+06			1.79E+06						21.28			21.06				21.28	21.06				
2076	Q02413	Desmoglei	DSG1	14	15.3	2.19E+07	1.90E+07	1.45E+08	4.18E+07	1.63E+07	1.55E+07	2.23E+07	2.74E+07	2.36E+08	24.32	24.34	27.52	25.43	24.12	24.11	24.41	24.51	27.05	24.34	24.12	24.51	1.842366	0.761217	1.497304
2963	Q9H845	Acyl-CoA d	ACAD9	5	8.9	5.80E+06	4.91E+06	5.49E+06	4.88E+06	4.35E+06	5.67E+06	4.09E+06	4.80E+06		22.39	22.46	22.73	22.21	22.24	22.7	21.94	21.98		22.46	22.24	21.96	0.180805	0.272213	0.030713

1760	P41214	Eukaryotic	EIF2D	6	18	6.95E+06	6.31E+06	5.87E+06	5.96E+06	9.21E+06	5.11E+06	7.83E+06	6.38E+06	9.39E+06	22.65	22.77	22.81	22.53	23.29	22.55	22.85	22.36	22.69	22.77	22.55	22.69	0.079968	0.43356	0.248726
1067	M0QXB4	Coatomer :	COPE	9	31.4	1.09E+07	1.69E+07	1.79E+07	1.28E+07	1.49E+07	1.80E+07	1.36E+07	1.50E+07	7.15E+06	23.36	24.2	24.35	23.63	23.98	24.37	23.64	23.63	22.26	24.2	23.98	23.63	0.532335	0.371736	0.794591
1783	P43487-2	Ran-specifi	RANBP1	6	39	2.03E+07	3.49E+07	3.47E+07	3.21E+07	3.18E+07	1.79E+07	1.79E+07	1.74E+07	2.11E+07	24.23	25.26	25.25	25.03	25.07	24.36	24.07	23.85	23.93	25.25	25.03	23.93	0.589187	0.400594	0.111568
2786	Q96SQ9	Cytochrom	CYP2S1	6	13.9	1.14E+07	1.02E+07	8.70E+06	9.02E+06	1.02E+07	8.02E+06	1.10E+07	1.07E+07	1.01E+07	23.42	23.47	23.32	23.11	23.45	23.2	23.3	23.12	22.81	23.42	23.2	23.12	0.077216	0.173125	0.244444
715	H0YDR3	Tetratricop	TTC9C	2	34.8			2.07E+06		1.87E+06	1.82E+06						21.31		21.08	21.11				21.31	21.09			0.020387	
898	H0YHA7	60S riboso	RPL18	5	37.1	5.23E+07	1.93E+07	9.48E+06	1.79E+07	9.85E+06	1.76E+07	1.35E+07			25.6	24.36	23.45	24.14	23.39	24.31	23.61			24.36	24.14	23.61	1.079972	0.489756	
2030	P78318	Immunogl	IGBP1	3	10		1.50E+06	1.65E+06			1.31E+06					20.82	20.97			20.67				20.89	20.67		0.10684		
2056	Q00577	Transcripti	PURA	3	18	1.27E+07	1.15E+07	7.10E+06	9.90E+06	1.56E+07	9.02E+06	1.14E+07	1.64E+07	1.07E+07	23.57	23.64	23.02	23.26	24.05	23.35	23.35	23.77	22.92	23.57	23.35	23.35	0.339698	0.434073	0.422721
1772	P42566	Epidermal	EPS15	6	9.8	1.78E+06	1.85E+06	1.67E+06	1.91E+06	1.52E+06	1.26E+06				20.67	21.12	21	20.89	20.77	20.65				21	20.77		0.231906	0.12002	
2686	Q92990-2	Glomulin	GLMN	4	8.6	5.94E+06	6.60E+06	7.26E+06	6.22E+06	5.57E+06	6.89E+06	6.31E+06	8.64E+06		22.42	22.83	23.07	22.6	22.61	22.97	22.55	22.83		22.83	22.61	22.69	0.327891	0.213504	0.198972
2978	Q9HAV4	Exportin-5	XPO5	40	40	7.09E+07	1.39E+08	1.41E+08	1.09E+08	1.36E+08	1.68E+08	1.37E+08	1.09E+08	4.87E+06	26.09	27.41	27.47	26.8	27.19	27.75	27.07	26.45	21.63	27.41	27.19	26.45	0.781237	0.477941	2.97917
2486	Q7L5D6	Golgi to ER	GET4	3	12.2	1.56E+06	1.89E+06	1.66E+06	1.67E+06		1.61E+06	1.79E+06	1.82E+06		20.48	21.15	20.98	20.63		20.88	20.68	20.62		20.98	20.76	20.65	0.347908	0.182908	0.044526
1851	P50213	Isocitrate c	IDH3A	6	17.5	4.32E+06	7.26E+06	5.26E+06	5.23E+06		5.00E+06	5.76E+06	5.87E+06	5.18E+06	21.99	22.99	22.65	22.33		22.51	22.43	22.25	21.75	22.65	22.42	22.25	0.504852	0.127253	0.349877
2385	Q5T4S7-3	E3 ubiquiti	UBR4	33	7.6	2.51E+07	2.60E+07	2.60E+07	2.83E+07	2.24E+07	2.17E+07	2.01E+07	4.38E+07	2.77E+07	24.53	24.81	24.86	24.81	24.56	24.58	24.26	25.16	24.26	24.81	24.58	24.26	0.174023	0.136746	0.52269
1025	J3QK89	Calcium hc	CHERP	9	10.2	7.51E+06	6.10E+06	5.93E+06	3.41E+06	5.25E+06	6.69E+06	2.11E+06	4.04E+06		22.77	22.72	22.82	21.7	22.54	22.92	20.87	21.75		22.77	22.54	21.31	0.049923	0.624919	0.625049
1786	P43897	Elongation	TSFM	4	16	3.57E+06	4.08E+06	3.53E+06	2.99E+06	3.91E+06	3.04E+06	3.96E+06	3.57E+06		21.73	22.25	22.06	21.53	22.1	21.83	21.86	21.58		22.06	21.83	21.72	0.264539	0.281911	0.201422
793	F8VR84	UPF0160 p	C12orf10	5	26.3	2.15E+07	1.11E+07	1.10E+07	1.14E+07	7.29E+06	9.63E+06	7.63E+06	8.61E+06	3.88E+06	24.31	23.59	23.66	23.43	22.99	23.45	22.79	22.82	21.24	23.66	23.43	22.79	0.394344	0.259134	0.904587
989	J3KMZ8	Zinc finger	DPF2	8	27.2	3.05E+07	2.44E+07	2.07E+07	2.13E+07	2.45E+07	1.95E+07	2.72E+07	3.94E+07	1.26E+07	24.86	24.69	24.53	24.41	24.67	24.46	24.69	25.04	23.17	24.69	24.46	24.69	0.161529	0.133845	0.995136
1952	P61201	COP9 signa	COPS2	9	20.3	1.18E+07	1.58E+07	1.38E+07	8.67E+06	1.24E+07	1.41E+07	9.33E+06	9.31E+06	5.54E+06	23.47	24.09	23.94	23.04	23.71	23.96	23.1	22.92	21.85	23.94	23.71	22.92	0.319875	0.476027	0.673952
1156	O15091-2	Mitochond	KIAA0391	5	11.8		2.85E+06	2.00E+06		2.06E+06	2.02E+06	1.86E+06				21.67	21.26		21.2	21.28	20.73			21.47	21.24	20.73	0.288158	0.056676	
445	C9J6V2	Retinitis pi	RP9	2	13.9	2.91E+06		2.83E+06	2.61E+06	2.30E+06			2.12E+06		21.45		21.74	21.37	21.35			20.89		21.59	21.36	20.89	0.206195	0.010635	
222	F5GYV5	ADP-ribosy	ARL6IP4	4	23.7		9.22E+06	8.16E+06			7.24E+06					23.31	23.25			23.05				23.28	23.05		0.041716		
1545	P17858	ATP-deper	PFKL	7	15.3	6.58E+06	6.51E+06	7.67E+06	5.34E+06	6.23E+06	5.22E+06	4.32E+06	4.45E+06		22.58	22.82	23.16	22.35	22.79	22.59	22.01	21.88		22.82	22.59	21.94	0.288895	0.218435	0.094983
1574	P21283	V-type pro	ATP6V1C1	4	8.4	2.16E+06	2.20E+06	2.72E+06	1.97E+06		2.06E+06				20.98	21.36	21.66	20.95		21.29				21.36	21.12		0.34366	0.241612	
638	E7EQ69	N-alpha-ac	NAA50	4	23.8		3.28E+06	4.77E+06			3.34E+06					21.91	22.5			21.97				22.2	21.97		0.4172		
1802	P47755	F-actin-cap	CAPZA2	10	54.2	2.76E+07	3.42E+07	2.52E+07	2.09E+07	2.29E+07	2.59E+07	2.26E+07	3.22E+07	1.24E+07	24.66	25.22	24.82	24.38	24.59	24.8	24.44	24.76	23.14	24.82	24.59	24.44	0.288329	0.212837	0.856599
304	A5YKK6	CCR4-NOT	CNOT1	31	15.5	2.87E+07	3.11E+07	2.79E+07	2.24E+07	2.47E+07	2.44E+07	3.17E+07	3.39E+07	1.84E+07	24.73	25.03	24.92	24.49	24.69	24.73	24.91	24.83	23.72	24.92	24.69	24.83	0.149298	0.130097	0.663392
1955	P61224-2	Ras-relatec	RAP1B	4	26.3	8.08E+06	6.35E+06	1.02E+07		5.94E+06	5.42E+06				22.9	22.78	23.55		22.69	22.63				22.9	22.66		0.413469	0.042953	
1458	P09661	U2 small n	SNRPA1	6	27.8	1.92E+07	1.68E+07	1.79E+07	1.57E+07	1.27E+07	1.69E+07	1.24E+07	1.18E+07		24.17	24.18	24.35	23.94	23.75	24.25	23.48	23.28		24.18	23.94	23.38	0.099298	0.252808	0.140687
3338	X6RAL5	Histone de	SAP18	4	19.2	1.73E+07	1.09E+07	1.04E+07	1.02E+07	9.50E+06	1.84E+07	1.27E+07	1.17E+07		24.02	23.57	23.57	23.29	23.34	24.4	23.52	23.26		23.57	23.34	23.39	0.260067	0.627805	0.185818
375	B4DJ81	NADH-ubic	NDUFS1	16	38.3	2.35E+07	2.05E+07	2.40E+07	1.45E+07	1.72E+07	1.84E+07	1.65E+07	1.58E+07	3.75E+06	24.45	24.43	24.76	23.82	24.21	24.4	23.94	23.72	21.18	24.45	24.21	23.72	0.189421	0.295608	1.533409
3089	Q9P2I0	Cleavage a	CPSF2	6	8.6	6.20E+06	4.66E+06	3.25E+06	4.81E+06	2.86E+06	3.85E+06	2.74E+06			22.49	22.4	21.94	22.19	21.69	22.16	21.31			22.4	22.16	21.31	0.297516	0.279034	
700	E9PII3	Band 4.1-li	EPB41L2	4	7.4	1.39E+08	1.26E+08	1.36E+08	1.24E+08	9.92E+07	1.35E+08				27.12	27.27	27.42	27.03	26.82	27.43				27.27	27.03		0.152241	0.309601	
656	E7ETY4	Serine/thre	MARK2	5	9.3	4.55E+06	4.90E+06	4.02E+06	3.83E+06	8.40E+06	3.45E+06	5.39E+06	4.76E+06		22.04	22.45	22.26	21.87	23.17	22.02	22.33	21.97		22.26	22.02	22.15	0.204804	0.708638	0.255373
1087	M0R2B7	DNA polym	POLD1	23	23.7	2.11E+07	1.84E+07	1.60E+07	1.65E+07	1.49E+07	1.53E+07	1.46E+07	1.42E+07	1.52E+07	24.28	24.29	24.15	24.04	23.98	24.1	23.77	23.55	23.46	24.28	24.04	23.55	0.080754	0.060024	0.159792
675	E9PC74	Translatior	EIF2B5	4	5.8	4.10E+06	3.83E+06	3.61E+06	3.24E+06	3.32E+06	3.61E+06	3.34E+06	2.39E+06		21.93	22.16	22.11	21.66	21.87	22.07	21.62	21.02		22.11	21.87	21.32	0.121875	0.208163	0.421859
1272	O75534-2	Cold shock	CSDE1	13	20.5	1.67E+07	2.19E+07	2.12E+07	1.60E+07	2.03E+07	1.76E+07	2.18E+07	1.51E+07		23.96	24.55	24.58	23.99	24.45	24.31	24.38	23.64		24.55	24.31	24.01	0.350027	0.237735	0.518056
2321	Q16658	Fascin	FSCN1	5	14	3.21E+06	6.33E+06	6.75E+06	5.30E+06	5.25E+06	7.98E+06	4.06E+06			21.59	22.78	22.94	22.34	22.54	23.19	21.92			22.78	22.54	21.92	0.737908	0.445759	
1640	P28074	Proteasom	PSMB5	11	43.3	1.97E+07	2.47E+07	3.33E+07	2.21E+07	1.78E+07	4.25E+07	2.32E+07	1.91E+07	1.98E+07	24.21	24.71	25.16	24.47	24.24	25.59	24.47	23.99	23.84	24.71	24.47	23.99	0.478225	0.72127	0.329352
1268	O75477	Erlin-1	ERLIN1	4	19.4	6.35E+06	7.09E+06	6.86E+06	5.48E+06	8.88E+06	5.72E+06	5.50E+06	7.75E+06		22.53	22.95	22.98	22.39	23.23	22.71	22.36	22.66		22.95	22.71	22.51	0.249815	0.424109	0.21197
2665	Q92616	Translatior	GCN1L1	69	29.4	8.70E+07	1.11E+08	1.04E+08	1.00E+08	9.61E+07	9.73E+07	8.17E+07	8.95E+07	3.53E+07	26.43	27.01	27.01	26.7	26.77	26.8	26.23	26.24	24.65	27.01	26.77	26.23	0.332757	0.049765	0.915027
106	A0A087WZ	Isocitrate c	IDH3B	4	12.1	3.54E+06	3.89E+06	4.22E+06	4.96E+06	3.39E+06	3.26E+06	4.45E+06	4.70E+06		21.71	22.18	22.33	22.25	21.9	21.94	22.05	21.95		22.18	21.94	22	0.324718	0.191284	0.070778
2797	Q99471	Prefoldin s	PFDN5	4	29.2	3.82E+06	4.09E+06	4.24E+06	3.57E+06	3.71E+06	4.97E+06	3.72E+06			21.84	22.25	22.33	21.75	22.01	22.5	21.78			22.25	22.01	21.78	0.266306	0.381546	
1400	P05386	60S acidic r	RPLP1	3	74.6	2.11E+07	4.38E+07	5.78E+07	4.00E+07	5.70E+07	3.56E+07	4.95E+07	6.43E+07		24.28	25.6	26.06	25.36	25.94	25.35	25.55	25.75		25.6	25.36	25.65	0.922253	0.34238	0.144404
993	J3KN32	REST corep	RCOR1	3	7.2	3.36E+06	2.71E+06	3.62E+06	2.49E+06	2.79E+06	2.21E+06	1.90E+06	2.31E+06		21.65	21.6	22.12	21.29	21.65	21.41	20.75	20.96		21.65	21.41	20.85	0.284901	0.184686	0.149432
850	G5E933	Myotubula	SBF1	48	33.6	1.65E+08	1.20E+08	1.05E+08	1.30E+08	1.11E+08	9.50E+07	1.95E+08	1.71E+08	1.32E+08	27.41	27.18	27.03	27.13	26.94	26.73	27.56	27.13	26.33	27.18	26.94	27.13	0.188765	0.200713	0.620121
1183	O43172-2	U4/U6 sma	PRPF4	3	5.2	1.51E+06	1.44E+06	1.54E+06		9.23E+05	1.51E+06				20.45	20.72	20.87		20.15	20.81				20.72	20.48		0.210355	0.463287	
2123	Q09161	Nuclear ca	NCBP1	13	22	2.77E+07	2.41E+07	2.52E+07	2.21E+07	1.95E+07	1.89E+07	2.02E+07	1.91E+07		24.66	24.67	24.82	24.47	24.38	24.42	24.28	23.99		24.67	24.42	24.13	0.08997	0.041299	0.200545
2541	Q8IY21	Probable A	DDX60	28	18.8	2.55E+07	3.34E+07	2.60E+07	2.14E+07	2.35E+07	2.88E+07	1.69E+07	1.58E+07	3.03E+06	24.55	25.18	24.86	24.42	24.61	25.01	23.98	23.72	20.96	24.86	24.61	23.72	0.312677	0.301573	1.675099
1161	O15173	Membrane	PGRMC2	5	20.2	4.20E+06	4.53E+06	4.74E+06	4.54E+06	2.82E+06	5.16E+06	3.20E+06	2.72E+06		21.96	22.36	22.49	22.11	21.67	22.57	21.57	21.23		22.36	22.11	21.4	0.27455	0.44948	0.235505
2428	Q6L8Q7-2	2,5-phosph	PDE12	5	10.3	3.59E+06		3.54E+06	3.23E+06	2.61E+06	3.14E+06	3.60E+06			21.74		22.06	21.65	21.54	21.89	21.72			21.9	21.65	21.72	0.22914	0.179082	
1735	P37108	Signal reco	SRP14	4	46.3	3.27E+07	4.15E+07	5.11E+07	3.25E+07	3.63E+07	3.55E+07	2.96E+07	2.78E+07	3.99E+07	24.97	25.53	25.91	25.05	25.28	25.33	24.8	24.53	24.78	25.53	25.28	24.78	0.47222	0.152214	0.149032
1542	P17342-2	Atrial natri	NPR3	4	9.8	1.81E+06	2.43E+06	1.83E+06	1.74E+06	1.72E+06	1.63E+06	1.53E+06			20.7	21.48	21.16	20.72	20.99	20.91	20.46			21.16	20.91	20.46	0.390984	0.14316	
1828	P49411	Elongation	TUFM	24	52.2	8.91E+08	6.94E+08	6.43E+08	7.84E+08	6.72E+08	6.56E+08	2.30E+08	5.94E+08	3.88E+08	29.83	29.73	29.8	29.55	29.44	29.56	27.78	28.74	27.73	29.8	29.55	27.78	0.051591	0.067651	0.571464
1796	P46926	Glucosamii	GNPDA1	5	22.5	4.28E+06	4.78E+06	3.79E+06	4.01E+06	3.12E+06	4.24E+06		3.32E+06		21.99	22.43	22.19	21.94	21.79	22.3		21.49		22.19	21.94	21.49	0.224303	0.260023	
1771	P42345	Serine/thre	MTOR	17	8.8	7.80E+06	1.10E+07	9.47E+06	6.54E+06	8.64E+06	8.04E+06	1.37E+07	9.42E+06		22.81	23.58	23.45	22.67	23.19	23.21	23.66	22.93		23.45	23.19	23.3	0.409341	0.305759	0.51314
2266	Q15102	Platelet-ac	PAFAH1B3	4	16.5	6.63E+06	6.58E+06	5.47E+06	5.80E+06	4.26E+06	4.86E+06	4.20E+06	3.89E+06		22.59	22.83	22.73	22.48	22.2	22.47	21.98	21.71		22.73	22.47	21.84	0.118837	0.158762	0.189669
962	H7C2Q8	Probable r	EBNA1BP2	9	27.1	7.46E+06	8.38E+06	9.99E+06	6.81E+06	6.84E+06	7.63E+06	5.89E+06	4.70E+06		22.76	23.18	23.53	22.73	22.92	23.14	22.47	21.95		23.18	22.92	22.21	0.383629	0.206581	0.361511
930	H3BP71	E3 ubiquiti	RNF40	2	5.3		1.20E+06	1.46E+06		1.05E+06	1.12E+06					20.52	20.82		20.33	20.49				20.67	20.41		0.211938	0.117898	
1804	P47813	Eukaryotic	EIF1AX	3	26.4	4.15E+06	6.80E+06	8.56E+06		4.50E+06	6.69E+06				21.94	22.87	23.3		22.31	22.92				22.87	22.62		0.693042	0.435223	
2060	Q00765	Receptor e	REEP5	4	10.6	3.66E+06	4.24E+06	4.07E+06	3.61E+06	3.72E+06	3.90E+06		2.51E+06		21.76	22.28	22.27	21.77	22.02	22.18		21.07		22.27	22.02	21.07	0.296561	0.205019	
2560	Q8N3U4	Cohesin su	STAG2	7	8.2	4.36E+06	3.27E+06	4.43E+06	4.04E+06	2.96E+06	2.81E+06		2.13E+06		22	21.9	22.39	21.95	21.72	21.74		20.91		22	21.74	20.91	0.257404	0.125675	
2229	Q14643-4	Inositol 1,4	ITPR1	18	8.7	2.03E+07	2.25E+07	2.33E+07	1.64E+07	1.88E+07	2.08E+07	1.28E+07	1.18E+07	4.14E+06	24.23	24.59	24.71	24.03	24.33	24.54	23.54	23.28	21.33	24.59	24.33	23.28	0.249457	0.256008	1.208918
2703	Q96AC1	Fermitin fa	FERMT2	12	25.6	4.62E+07	5.21E+07	5.65E+07	4.42E+07	4.51E+07	5.97E+07	4.81E+07	4.62E+07	4.92E+07	25.45	25.85	26.04	25.53	25.59	26.05	25.49	25.27	25.09	25.85	25.59	25.27	0.300802	0.284759	0.205013
2744	Q96HW7	Integrator	INTS4	47	56.3	4.30E+08	3.83E+08	3.25E+08	4.10E+08	3.92E+08	4.56E+08	2.03E+08	5.25E+08	1.98E+08	28.72	28.98	28.9	28.64	28.64	29.05	27.64	28.5	26.81	28.9	28.64	27.64	0.138068	0.239684	0.844731
2312	Q16513-2	Serine/thre	PKN2	6	9.3	2.30E+06	2.32E+06	2.59E+06	1.84E+06	1.99E+06	2.16E+06				21.08	21.41	21.61	20.82	21.15	21.37				21.41	21.15		0.264013	0.279458	
2022	P68032	Actin, alph	ACTC1	7	58.6	2.03E+08	2.10E+08	1.85E+08	1.81E+08	1.96E+08	2.00E+08	1.52E+08	1.75E+08	9.08E+07	27.73	28.05	27.92	27.51	27.66	28.01	27.19	27.18	25.96	27.92	27.66	27.18	0.161504	0.258725	0.708211
1090	O00116	Alkyldihyd	AGPS	7	16.6	1.37E+07	1.51E+07	1.65E+07	9.61E+06	1.27E+07	1.51E+07	1.36E+07	1.35E+07	1.29E+07	23.65	24.02	24.2	23.21	23.75	24.07	23.64	23.46	23.21	24.02	23.75	23.46	0.283881	0.437133	0.217384
3108	Q9UBT2	SUMO-acti	UBA2	9	16.2	9.72E+06	1.34E+07	1.50E+07	7.25E+06	1.14E+07	1.30E+07	8.69E+06	7.46E+06	7.52E+06	23.18	23.85	24.06	22.82	23.59	23.85	22.99	22.6	22.36	23.85	23.59	22.6	0.456953	0.533663	0.319618
2430	Q6NUK1	Calcium-bi	SLC25A24	2	5.5	1.73E+06	1.80E+06	1.84E+06		1.43E+06	1.64E+06				20.61	21.08	21.16		20.71	20.93				21.08	20.82		0.298355	0.156542	
2557	Q8N3C0	Activating	ASCC3	24	12.7	3.11E+07	2.72E+07	2.45E+07	2.11E+07	2.52E+07	2.20E+07	2.41E+07	1.95E+07	2.11E+07	24.9	24.86	24.78	24.4	24.75	24.6	24.52	24.03	23.93	24.86	24.6	24.03	0.060899	0.17345	0.313401
217	F5H3C5	Superoxide	SOD2	2	20.7		1.52E+06	1.45E+06			1.20E+06					20.84	20.81			20.56				20.83	20.56		0.024426		
2728	Q96EL3	39S riboso	MRPL53	2	20.5	2.26E+06	2.51E+06	2.35E+06	2.37E+06	1.69E+06	2.90E+06		3.09E+06		21.04	21.52	21.47	21.2	20.95	21.78		21.4		21.47	21.2	21.4	0.263785	0.425371	
1996	P62805	Histone H4	HIST1H4A	9	53.4	8.51E+08	5.05E+08	6.09E+08	7.17E+08	6.42E+08	6.84E+08	1.31E+09	1.44E+09	8.62E+08	29.81	29.23	29.68	29.41	29.35	29.65	30.55	30.45	28.98	29.68	29.41	30.45	0.302515	0.159322	0.878695
179	F8W9B8	Exocyst co	EXOC5	5	8.4	3.02E+06	3.89E+06	3.82E+06	3.94E+06	3.53E+06	2.98E+06	3.65E+06			21.52	22.18	22.2	21.91	21.95	21.81	21.73			22.18	21.91	21.73	0.389903	0.071351	
2492	Q7Z3B4-3	Nucleopori	NUP54	5	11.8	4.42E+06	2.60E+06	3.23E+06	2.89E+06	2.80E+06	2.96E+06				22.02	21.56	21.93	21.5	21.66	21.81				21.93	21.66		0.24035	0.151981	
1195	O43583	Density-re	DENR	2	12.6	8.46E+06	3.88E+06	4.31E+06			3.63E+06				22.97	22.17	22.36			22.09				22.36	22.09		0.415701		

374	B4DGU4	Catenin be	CTNNB1	9	15.2	1.23E+07	1.32E+07	1.25E+07	1.05E+07	1.12E+07	1.25E+07	1.22E+07	1.18E+07	8.06E+06	23.52	23.83	23.82	23.33	23.55	23.79	23.44	23.28	22.46	23.82	23.55	23.28	0.175393	0.229932	0.527587
223	B3KRD1	Syntabulin	SYBU	3	5.9	3.78E+06	2.20E+06	2.74E+06		2.38E+06					21.82	21.36	21.68		21.41					21.68	21.41		0.239544		
2889	Q9BZE4	Nucleolar (	GTPBP4	6	9.8	6.40E+06	5.19E+06	4.72E+06	4.44E+06	4.69E+06	4.06E+06	6.38E+06	4.32E+06		22.54	22.51	22.49	22.09	22.36	22.24	22.57	21.84		22.51	22.24	22.2	0.0289	0.136564	0.516223
1194	O43488	Aflatoxin B	AKR7A2	12	46.5	3.35E+08	3.02E+08	2.92E+08	3.45E+08	1.99E+08	2.85E+08	2.83E+08	2.84E+08	1.27E+08	28.38	28.63	28.71	28.36	27.7	28.49	27.98	27.73	26.3	28.63	28.36	27.73	0.170133	0.424557	0.907234
3181	Q9Y223	Bifunctiona	GNE	4	6.5	2.39E+06	2.41E+06	2.75E+06	2.07E+06	2.23E+06	1.90E+06	2.36E+06	2.10E+06		21.13	21.46	21.69	20.98	21.3	21.19	21.1	20.87		21.46	21.19	20.99	0.280415	0.164931	0.159741
1934	P58107	Epiplakin	EPPK1	25	25.8	1.73E+07	3.48E+07	3.36E+07	3.07E+07	2.52E+07	3.63E+07	3.32E+07	5.03E+07	1.07E+07	24.02	25.25	25.18	24.91	24.75	25.39	24.97	25.39	22.92	25.18	24.91	24.97	0.689605	0.331162	1.319365
1263	O75400-2	Pre-mRNA	PRPF40A	7	8.5	7.84E+06	8.15E+06	9.76E+06	4.89E+06	6.67E+06	6.84E+06	5.18E+06	4.84E+06		22.83	23.14	23.49	22.22	22.87	22.96	22.26	22		23.14	22.87	22.13	0.333357	0.403312	0.189559
2183	Q13561	Dynactin si	DCTN2	17	49.9	7.37E+07	8.89E+07	6.91E+07	6.80E+07	5.60E+07	7.11E+07	7.79E+07	7.16E+07	3.22E+07	26.15	26.65	26.39	26.11	25.92	26.33	26.14	25.88	24.48	26.39	26.11	25.88	0.249924	0.209255	0.888379
1472	P10644	cAMP-depe	PRKAR1A	10	22.8	1.41E+07	1.65E+07	1.23E+07	1.00E+07	1.08E+07	1.21E+07	1.38E+07	1.69E+07		23.69	24.12	23.79	23.26	23.52	23.71	23.68	23.8		23.79	23.52	23.74	0.227391	0.225081	0.089355
1344	O96005-3	Cleft lip an	CLPTM1	3	5.5	1.77E+06	1.80E+06	1.77E+06	1.68E+06	1.86E+06	1.51E+06	1.78E+06	1.51E+06		20.66	21.08	21.11	20.65	21.07	20.81	20.66	20.29		21.08	20.81	20.48	0.252938	0.21345	0.260979
933	H3BPG8	Exportin-6	XPO6	3	21.1		1.33E+06	1.36E+06		1.17E+06			1.07E+06			20.63	20.72		20.4			19.78		20.67	20.4	19.78	0.065706		
684	E9PEB5	Far upstrea	FUBP1	11	22	2.54E+07	2.42E+07	2.73E+07	1.81E+07	1.98E+07	2.73E+07	1.83E+07	1.52E+07	9.48E+06	24.55	24.68	24.9	24.17	24.4	24.93	24.1	23.66	22.71	24.68	24.4	23.66	0.177065	0.389894	0.712301
2283	Q15417-3	Calponin-3	CNN3	3	18	4.32E+06	5.16E+06	3.50E+06	3.28E+06	3.13E+06	2.82E+06		3.37E+06		21.99	22.51	22.03	21.66	21.8	21.75		21.5		22.03	21.75	21.5	0.288143	0.066199	
40	A0A087WL	Bifunctiona	TKFC	5	8.7	2.97E+06	3.30E+06	3.96E+06		2.42E+06	3.08E+06	3.04E+06			21.49	21.92	22.24		21.43	21.85	21.49			21.92	21.64	21.49	0.375349	0.296411	
1664	P30405	Peptidyl-pr	PPIF	3	31.4	3.68E+06	5.54E+06	4.51E+06		3.30E+06	4.67E+06		3.15E+06		21.78	22.61	22.42		21.86	22.42		21.43		22.42	22.14	21.43	0.433896	0.39513	
695	H7C3S9	COP9 signa	COPS8	4	69.9	4.40E+06	6.17E+06	5.24E+06	3.96E+06	4.67E+06	7.56E+06	6.45E+06	6.02E+06		22.01	22.75	22.64	21.93	22.36	23.12	22.58	22.29		22.64	22.36	22.43	0.395483	0.602602	0.208726
632	E7EPB3	60S riboso	RPL14	5	42.7	1.02E+08	8.97E+07	6.04E+07	4.64E+07	7.61E+07	7.81E+07	7.39E+07	6.84E+07	2.56E+07	26.72	26.66	26.13	25.61	26.38	26.49	26.08	25.82	24.14	26.66	26.38	25.82	0.323153	0.479435	1.052951
2264	Q15075	Early endo	EEA1	13	11.3	8.66E+06	8.25E+06	6.68E+06	8.05E+06	6.04E+06	5.28E+06	6.64E+06	4.52E+06	7.46E+06	23	23.15	22.94	22.94	22.72	22.6	22.63	21.9	22.34	23	22.72	22.34	0.111797	0.172964	0.370519
669	E9PGZ1	Caldesmor	CALD1	9	21.5	2.35E+07	2.75E+07	2.82E+07	2.45E+07	2.34E+07	2.29E+07	2.48E+07	1.54E+07		24.45	24.89	24.95	24.61	24.6	24.66	24.57	23.67		24.89	24.61	24.12	0.272535	0.032481	0.631573
1390	P04843	Dolichyl-di	RPN1	22	48.3	9.50E+07	1.13E+08	1.21E+08	1.03E+08	1.16E+08	9.44E+07	1.05E+08	1.25E+08	7.58E+07	26.61	27.02	27.22	26.74	26.98	26.71	26.68	26.69	25.64	27.02	26.74	26.68	0.310276	0.146933	0.600562
2687	Q93008-1	Probable u	USP9X	28	13.6	2.71E+07	3.26E+07	3.03E+07	2.76E+07	2.79E+07	2.55E+07	2.25E+07	1.87E+07	9.17E+06	24.63	25.12	25.06	24.77	24.91	24.78	24.43	23.96	22.65	25.06	24.78	23.96	0.268138	0.08038	0.92142
1021	J3QR09	Ribosomal	RPL19	3	17.1			7.09E+06	1.28E+07	1.03E+07	8.24E+06	3.43E+06				22.95	23.87	23.32	23.12	22.01				23.41	23.12		0.647509	0.704405	
1845	P49792	E3 SUMO-p	RANBP2	47	19.7	3.58E+07	6.08E+07	6.41E+07	3.87E+07	6.28E+07	5.07E+07	2.81E+07	3.68E+07	6.06E+06	25.06	26.1	26.23	25.3	26.11	25.81	24.74	24.94	21.99	26.1	25.81	24.74	0.640813	0.409411	1.648496
1653	P30044-2	Peroxiredo	PRDX5	11	60.5	7.09E+07	8.73E+07	1.05E+08	7.74E+07	7.39E+07	8.79E+07	7.12E+07	5.71E+07	7.58E+07	26.09	26.6	27.03	26.3	26.32	26.66	26	25.58	25.64	26.6	26.32	25.64	0.472467	0.202281	0.228881
1250	O75165	DnaJ homo	DNAJC13	26	14	1.82E+07	1.96E+07	1.94E+07	1.63E+07	1.60E+07	1.78E+07	1.20E+07	1.39E+07		24.1	24.38	24.43	24.02	24.09	24.34	23.42	23.51		24.38	24.09	23.47	0.175687	0.169534	0.063393
1811	R4GMR5	26S protea	PSMD8	9	35.9	2.86E+07	2.46E+07	1.72E+07	2.21E+07	1.81E+07	1.85E+07	9.20E+06	8.32E+06		24.72	24.7	24.26	24.47	24.28	24.41	23.08	22.77		24.7	24.41	22.93	0.257391	0.098123	0.22158
1855	P50416-2	Carnitine C	CPT1A	6	9	4.14E+06	4.94E+06	4.81E+06	4.31E+06	4.24E+06	4.29E+06	4.42E+06	4.21E+06		21.94	22.48	22.51	22.05	22.19	22.3	22.04	21.81		22.48	22.19	21.92	0.321969	0.126022	0.16076
1589	P22695	Cytochrom	UQCRC2	10	34	1.42E+07	1.45E+07	1.42E+07	1.14E+07	1.20E+07	1.77E+07	1.17E+07	1.14E+07	1.22E+07	23.7	23.95	23.98	23.43	23.66	24.33	23.39	23.21	23.12	23.95	23.66	23.21	0.152581	0.463255	0.135811
859	G5EA31	Protein tra	SEC24C	14	17.9	1.07E+07	1.13E+07	1.23E+07	1.23E+07	8.17E+06	8.97E+06	1.12E+07	8.05E+06		23.33	23.62	23.79	23.56	23.12	23.33	23.32	22.72		23.62	23.33	23.02	0.237133	0.222438	0.427118
3240	Q9Y570	Protein ph	PPME1	11	29	1.49E+07	2.30E+07	2.61E+07	1.44E+07	1.87E+07	1.95E+07	1.32E+07	1.39E+07	5.68E+06	23.78	24.6	24.86	23.81	24.32	24.46	23.57	23.51	21.88	24.6	24.32	23.51	0.566105	0.341676	0.95879
1721	P35998	26S protea	PSMC2	27	64.2	2.26E+08	2.39E+08	1.76E+08	1.89E+08	1.77E+08	1.75E+08	1.54E+08	2.24E+08	9.30E+07	27.87	28.24	27.81	27.58	27.57	27.83	27.21	27.51	25.98	27.87	27.58	27.21	0.232635	0.145851	0.80716
1136	O14776-2	Transcripti	TCERG1	7	5.8	5.90E+06	7.73E+06	7.39E+06	4.78E+06	6.19E+06	6.18E+06	4.86E+06	5.19E+06		22.41	23.06	23.1	22.18	22.77	22.82	22.16	22.06		23.06	22.77	22.11	0.385903	0.353223	0.067497
2275	Q15366-6	Poly(rC)-bi	PCBP2	14	55.1	3.48E+08	3.13E+08	2.49E+08	3.01E+08	2.85E+08	2.35E+08	2.99E+08	3.47E+08	4.73E+08	28.43	28.72	28.5	28.21	28.27	28.21	28.06	27.92	28.22	28.5	28.21	28.06	0.148171	0.034996	0.148
284	A0A0X1KG	Negative e	NELFB	4	6.8	3.67E+06	4.48E+06	5.10E+06	3.60E+06	3.80E+06	3.87E+06	3.81E+06	3.64E+06		21.77	22.34	22.59	21.76	22.05	22.17	21.81	21.6		22.34	22.05	21.71	0.417217	0.207731	0.149082
2413	Q5W0B1	RING finge	RNF219	27	35.1	3.26E+08	2.56E+08	2.33E+08	2.64E+08	3.18E+08	2.17E+08	4.62E+08	3.50E+08	2.13E+08	28.35	28.38	28.41	28.05	28.35	28.08	28.75	27.95	26.94	28.38	28.08	27.95	0.030393	0.164269	0.905466
2603	Q8NI27	THO comp	THOC2	12	7.5	1.02E+07	9.47E+06	7.89E+06	8.28E+06	7.10E+06	8.60E+06	4.02E+06	4.89E+06		23.27	23.36	23.2	22.97	22.96	23.28	21.9	22.01		23.27	22.97	21.95	0.082637	0.182765	0.074834
2399	Q5U5X0	Complex II	LYRM7	2	24			1.63E+06			1.26E+06						20.94			20.65				20.94	20.65				
1954	P61221	ATP-bindin	ABCE1	7	12.9	1.06E+07	8.73E+06	1.14E+07	7.24E+06	7.44E+06	9.61E+06	7.64E+06	5.77E+06		23.31	23.23	23.72	22.82	23.02	23.44	22.79	22.23		23.31	23.02	22.51	0.263383	0.318373	0.396031
2818	Q9BPX3	Condensin	NCAPG	16	16.4	1.07E+07	2.16E+07	2.37E+07	1.74E+07	1.77E+07	1.97E+07	1.77E+07	1.65E+07	5.80E+06	23.33	24.53	24.75	24.09	24.24	24.47	24.06	23.78	21.92	24.53	24.24	23.78	0.766221	0.192089	1.163963
3058	Q9NYU1	UDP-gluco	UGGT2	28	20	4.29E+07	3.66E+07	3.21E+07	3.32E+07	2.57E+07	2.89E+07	3.78E+07	3.75E+07	3.22E+07	25.36	25.32	25.12	25.09	24.79	25.02	25.17	24.98	24.48	25.32	25.02	24.98	0.128466	0.157676	0.353246
2075	Q02388-2	Collagen al	COL7A1	13	7.2	8.04E+06	1.23E+07	1.25E+07	1.29E+07	9.82E+06	9.71E+06	1.19E+07	9.58E+06		22.89	23.75	23.82	23.65	23.38	23.45	23.41	22.97		23.75	23.45	23.19	0.520443	0.13872	0.310435
1886	P52298	Nuclear ca	NCBP2	2	15.4			5.44E+06			4.66E+06						22.71			22.41				22.71	22.41				
2657	Q92538-3	Golgi-speci	GBF1	11	6.4	6.01E+06	6.40E+06	5.78E+06	6.25E+06	5.11E+06	4.49E+06	4.80E+06	4.85E+06	5.57E+06	22.45	22.79	22.79	22.61	22.49	22.37	22.14	22	21.86	22.79	22.49	22	0.200238	0.118697	0.136658
1881	P51970	NADH deh	NDUFA8	3	19.2		2.43E+06	2.17E+06	1.94E+06	1.97E+06	2.18E+06	2.38E+06				21.48	21.37	20.91	21.12	21.39	21.11			21.42	21.12	21.11	0.074103	0.237003	
995	J3KNF4	Copper cha	CCS	3	11.8		1.81E+06	1.79E+06			1.52E+06					21.09	21.14			20.81				21.12	20.81		0.032858		
2564	Q8N5K1	CDGSH iror	CISD2	2	20	4.44E+06	4.48E+06	4.22E+06	3.82E+06	3.74E+06	3.72E+06	3.35E+06	3.91E+06		22.02	22.34	22.33	21.87	22.03	22.12	21.63	21.72		22.33	22.03	21.67	0.18002	0.127855	0.063441
3186	Q9Y266	Nuclear mi	NUDC	10	35.3	2.66E+07	3.66E+07	2.84E+07	2.46E+07	2.44E+07	2.61E+07	2.62E+07	2.45E+07		24.61	25.32	24.96	24.62	24.66	24.82	24.65	24.37		24.96	24.66	24.51	0.354799	0.110276	0.196384
1805	P47897	Glutamine-	QARS	41	58.6	6.64E+08	3.69E+08	4.90E+08	5.27E+08	4.92E+08	5.95E+08	2.89E+08	4.05E+08	1.66E+08	29.4	28.89	29.41	29.02	29.1	29.44	28.02	28.17	26.57	29.4	29.1	28.02	0.294796	0.219058	0.886663
2910	Q9H0A0	N-acetyltra	NAT10	11	12.1	6.20E+06	6.50E+06	1.07E+07	4.88E+06	5.17E+06	5.40E+06	1.84E+06		2.20E+06	22.49	22.82	23.61	22.21	22.51	22.63	20.71		20.43	22.82	22.51	20.57	0.573215	0.215988	0.201505
949	H7BXY3	Putative AT	DHX30	17	17.7	1.72E+07	1.29E+07	1.41E+07	8.64E+06	1.20E+07	1.67E+07	1.12E+07	1.33E+07	4.11E+06	24.01	23.82	23.97	23.03	23.66	24.23	23.32	23.45	21.32	23.97	23.66	23.32	0.10168	0.59952	1.19575
1665	P30419-2	Glycylpept	NMT1	3	9.4		3.15E+06	3.56E+06			2.66E+06		1.95E+06			21.84	22.08			21.66		20.74		21.96	21.66	20.74	0.166637		
2932	Q9H307	Pinin	PNN	8	11.7	1.21E+07	9.87E+06	7.72E+06	9.76E+06	6.98E+06	7.54E+06	9.55E+06	7.28E+06		23.5	23.42	23.17	23.22	22.95	23.11	23.13	22.55		23.42	23.11	22.84	0.175455	0.140717	0.406696
1440	P08574	Cytochrom	CYC1	4	13.8	6.02E+06	8.91E+06	1.08E+07	4.98E+06	6.99E+06	1.01E+07	4.02E+06	5.38E+06		22.45	23.25	23.63	22.26	22.95	23.5	21.9	22.11		23.25	22.95	22.01	0.601172	0.620281	0.149984
2930	Q9H2U2	Inorganic p	PPA2	5	20.4	8.48E+06	8.69E+06	9.74E+06	6.97E+06		7.33E+06	6.10E+06	6.96E+06		22.97	23.22	23.49	22.77		23.07	22.52	22.5		23.22	22.92	22.51	0.258919	0.212357	0.013014
1916	P54709	Sodium/pc	ATP1B3	5	22.9	1.48E+07	1.74E+07	1.54E+07	1.23E+07	1.46E+07	1.25E+07	1.14E+07	1.17E+07	8.27E+06	23.77	24.24	24.1	23.56	23.94	23.79	23.35	23.26	22.5	24.1	23.79	23.26	0.239857	0.191643	0.470465
2324	Q16719	Kynurenina	KYNU	8	24.7	5.03E+06	1.43E+07	1.13E+07	6.22E+06	9.88E+06	1.21E+07	9.74E+06	8.25E+06		22.19	23.94	23.7	22.6	23.4	23.71	23.16	22.76		23.7	23.4	22.96	0.949698	0.57183	0.28725
1757	P40938-2	Replication	RFC3	6	17.7	6.05E+06	8.88E+06	7.14E+06	6.80E+06	5.32E+06	5.77E+06	5.57E+06	5.54E+06	6.30E+06	22.46	23.25	23.03	22.72	22.55	22.74	22.38	22.18	22.08	23.03	22.72	22.18	0.408097	0.102588	0.152408
651	E7ESC6	Exportin-7	XPO7	17	17.6	1.42E+07	1.45E+07	1.48E+07	1.11E+07	1.19E+07	1.30E+07	1.01E+07	4.08E+06		23.7	23.95	24.03	23.39	23.64	23.85	23.21	21.77		23.95	23.64	22.49	0.17211	0.228655	1.017367
3136	Q9UI26	Importin-1	IPO11	6	9	4.67E+06	7.78E+06	5.07E+06	2.48E+06	4.41E+06	4.86E+06	3.29E+06	3.47E+06		22.08	23.07	22.57	21.28	22.26	22.47	21.61	21.54		22.57	22.26	21.57	0.494582	0.637065	0.047271
463	C9JIG9	Serine/thre	OXSR1	3	7.1	3.98E+06	4.51E+06	5.20E+06	3.73E+06		4.11E+06			2.66E+06	21.88	22.36	22.62	21.84		22.26			20.81	22.36	22.05	20.81	0.372353	0.295852	
1896	P52907	F-actin-cap	CAPZA1	12	65	7.27E+07	8.58E+07	7.03E+07	6.57E+07	6.27E+07	6.66E+07	5.38E+07	7.15E+07	5.18E+07	26.13	26.56	26.41	26.05	26.1	26.23	25.64	25.87	25.13	26.41	26.1	25.64	0.222879	0.090295	0.378112
2315	Q16543	Hsp90 co-c	CDC37	8	26.7	1.81E+07	2.39E+07	2.51E+07	1.94E+07	1.90E+07	2.07E+07	2.56E+07	3.69E+07	3.54E+07	24.08	24.65	24.81	24.25	24.34	24.53	24.62	24.95	24.66	24.65	24.34	24.66	0.381503	0.144092	0.177217
1623	P26368-2	Splicing fac	U2AF2	4	17.2	7.18E+06	3.81E+06	4.11E+06	4.24E+06		3.25E+06	3.70E+06			22.71	22.15	22.29	22.02		21.93	21.78			22.29	21.98	21.78	0.291317	0.067051	
1202	O43765	Small gluta	SGTA	3	11.8	3.74E+06	4.69E+06	3.62E+06		2.70E+06	3.42E+06				21.8	22.4	22.12		21.6	22.01				22.12	21.8		0.301566	0.284847	
2188	Q13619	Cullin-4A	CUL4A	18	26.4	2.26E+07	3.22E+07	3.68E+07	2.27E+07	2.54E+07	3.03E+07	3.80E+07	2.78E+07	7.63E+06	24.37	25.09	25.38	24.51	24.77	25.1	25.18	24.53	22.38	25.09	24.77	24.53	0.520529	0.29269	1.464522
1807	P48047	ATP syntha	ATP5O	9	54.5	1.85E+07	2.55E+07	3.15E+07	1.94E+07	2.05E+07	2.54E+07	1.42E+07	1.73E+07	9.77E+06	24.13	24.78	25.09	24.25	24.46	24.76	23.72	23.83	22.74	24.78	24.46	23.72	0.487737	0.258987	0.599375
3196	Q9Y2P8	RNA 3-term	RCL1	11	33.5	6.67E+07	5.38E+07	4.37E+07	4.54E+07	5.57E+07	3.54E+07	4.82E+07	4.60E+07	2.73E+07	25.93	25.9	25.6	25.58	25.91	25.32	25.51	25.25	24.23	25.9	25.58	25.25	0.182961	0.295415	0.677053
3190	Q9Y295	Developme	DRG1	13	43.1	3.05E+07	3.05E+07	2.94E+07	2.01E+07	2.47E+07	2.69E+07	2.20E+07	2.84E+07	1.57E+07	24.86	25.01	25.02	24.31	24.69	24.89	24.4	24.58	23.53	25.01	24.69	24.4	0.092499	0.293824	0.563277
210	Q5W0H4	Translation	TPT1	5	29.8	1.31E+07	1.87E+07	1.39E+07	1.22E+07	1.41E+07	1.15E+07	1.02E+07	1.15E+07	8.93E+06	23.61	24.31	23.95	23.55	23.9	23.64	23.22	23.22	22.61	23.95	23.64	23.22	0.35192	0.180515	0.35482
2206	Q14137-2	Ribosome	BOP1	2	5.2	2.91E+06	2.80E+06	2.29E+06		1.98E+06	1.84E+06	2.67E+06			21.45	21.64	21.44		21.13	21.12	21.28			21.45	21.13	21.28	0.111995	0.007131	
3009	Q9NR31	GTP-bindir	SAR1A	3	22.7	1.04E+07	1.79E+07	2.24E+07	1.06E+07	1.53E+07	1.39E+07	1.13E+07	9.99E+06		23.29	24.26	24.66	23.34	24.01	23.95	23.34	23.02		24.26	23.95	23.18	0.701979	0.368015	0.222657
1000	J3KNQ4	Alpha-parv	PARVA	4	15.3	2.35E+06	3.65E+06	3.44E+06	3.28E+06	2.83E+06	3.95E+06	3.59E+06	4.05E+06	2.78E+06	21.11	22.06	22	21.66	21.68	22.2	21.71	21.76	20.86	22	21.68	21.71	0.533071	0.305706	0.505918
660	E7EX73	Eukaryotic	EIF4G1	15	13.5	4.14E+07	6.45E+07	8.12E+07	3.17E+07	5.49E+07	5.80E+07	2.20E+07	1.98E+07		25.28	26.2	26.63	25	25.88	25.99	24.4	24.07		26.2	25.88	24.23	0.686866	0.542973	0.236753
1532	P15927	Replication	RPA2	3	15.6	1.84E+06	5.47E+06	3.77E+06	3.72E+06	3.31E+06	3.64E+06				20.76	22.59	22.19	21.83	21.87	22.1				22.19	21.87		0.960505	0.144256	
167	A0A0A0MI	Unconvent	MYO6	14	13.6	8.98E+06	9.51E+06	7.59E+06	7.30E+06	6.45E+06	5.93E+06	7.05E+06	8.47E+06		23.07	23.37	23.14	22.83	22.82	22.77	22.7	22.8		23.14	22.82	22.75	0.161173	0.032982	0.067839
992	J3KN29	26S protea	PSMD9	5	26.6	7.42E+06	8.70E+06	8.15E+06	7.75E+06	5.70E+06	8.56E+06	5.22E+06	6.64E+06		22.75	23.23	23.25	22.9	22.65	23.27	22.28	22.41		23.23	22.9	22.34	0.283088	0.311578	0.09045
1675	P31040	Succinate c	SDHA	13	23.3	4.74E+07	4.22E+07	4.00E+07	2.00E+07	3.43E+07	3.80E+07	3.01E+07	1.68E+07		25.47	25.55	25.51	24.31	25.19	25.43	24.83	23.79		25.51	25.19	24.31	0.04287	0.588484	0.735817

1676	P31151	Protein S1(	S100A7	2	11.9	8.04E+06				6.95E+06	3.92E+06		8.35E+06		22.89				22.94	22.19		22.77		22.89	22.56	22.77		0.528235	
1380	P04083	Annexin A1	ANXA1	18	57.5	5.38E+07	6.68E+07	6.43E+07	8.24E+07	2.82E+07	5.47E+07	4.15E+07	2.93E+07	9.54E+06	25.65	26.24	26.24	26.43	24.92	25.91	25.29	24.63	22.72	26.24	25.91	24.63	0.338218	0.764908	1.335735
117	A0A087X0I	Tight junct	TJP1	8	6.7	2.23E+06	3.43E+06	2.60E+06	1.68E+06	2.22E+06	2.19E+06	1.66E+06	1.77E+06		21	21.97	21.61	20.65	21.29	21.4	20.57	20.58		21.61	21.29	20.58	0.492276	0.404421	0.008197
2024	P68104	Elongation	EEF1A1	30	73.8	4.51E+09	5.40E+09	4.09E+09	5.43E+09	6.18E+09	5.25E+09	6.24E+09	5.95E+09	6.25E+09	32.77	33.2	33.13	32.33	32.8	32.95	32.26	31.91	31.21	33.13	32.8	31.91	0.229841	0.32301	0.534742
1288	O75909	Cyclin-K	CCNK	3	6.7		1.61E+06	2.18E+06	1.73E+06	1.59E+06	1.52E+06	1.52E+06				20.9	21.38	20.7	20.83	20.81	20.43			21.14	20.81	20.43	0.343719	0.071868	
3260	Q9Y6A5	Transformi	TACC3	6	12.1		4.05E+06	3.87E+06	4.28E+06		2.84E+06	4.92E+06				22.24	22.22	22.04		21.76	22.18			22.23	21.9	22.18	0.01069	0.200821	
1870	P51398-2	28S ribosoı	DAP3	4	13.2	6.81E+06	7.24E+06	6.12E+06	4.97E+06	6.38E+06	5.02E+06	5.59E+06	6.95E+06	5.11E+06	22.63	22.98	22.84	22.25	22.81	22.52	22.39	22.49	21.73	22.84	22.52	22.39	0.176481	0.276729	0.414766
1256	O75348	V-type pro	ATP6V1G1	4	30.5	5.45E+06	7.29E+06	6.80E+06	5.66E+06	5.67E+06	7.16E+06	6.47E+06	5.38E+06	4.55E+06	22.29	22.99	22.96	22.46	22.63	23.03	22.59	22.11	21.51	22.96	22.63	22.11	0.399382	0.293862	0.538762
1843	P49770	Translatior	EIF2B2	3	11.7	2.34E+06	2.83E+06	2.21E+06	2.05E+06	2.38E+06	1.80E+06	2.16E+06	2.69E+06		21.1	21.66	21.4	20.97	21.41	21.07	20.92	21.2		21.4	21.07	21.06	0.276955	0.22948	0.203757
2131	Q12768	WASH com	KIAA0196	9	10.6	5.19E+06	5.33E+06	5.12E+06	4.09E+06	4.46E+06	4.02E+06	5.69E+06	5.64E+06	4.51E+06	22.22	22.55	22.59	21.97	22.29	22.22	22.41	22.2	21.47	22.55	22.22	22.2	0.202954	0.16933	0.4958
3131	Q9UHV9	Prefoldin s	PFDN2	4	32.5	8.50E+06	1.33E+07	1.26E+07	1.04E+07	1.07E+07	1.45E+07	1.06E+07	8.21E+06	1.01E+07	22.98	23.84	23.84	23.33	23.51	24.02	23.26	22.75	22.81	23.84	23.51	22.81	0.497924	0.359164	0.277665
3158	Q9ULV4	Coronin-1C	CORO1C	4	12.4	8.62E+06	1.10E+07	1.07E+07	5.55E+06	9.03E+06	9.59E+06	8.71E+06	8.16E+06		23	23.58	23.61	22.41	23.25	23.44	23	22.74		23.58	23.25	22.87	0.345817	0.546414	0.181688
1349	P00387-2	NADH-cytc	CYB5R3	8	34.5	3.01E+07	2.75E+07	2.89E+07	2.53E+07	2.15E+07	2.13E+07	4.17E+07	3.60E+07	2.79E+07	24.84	24.89	24.98	24.66	24.51	24.56	25.3	24.91	24.28	24.89	24.56	24.91	0.071937	0.076241	0.515807
584	D3YTB1	60S ribosoı	RPL32	7	41.4	1.73E+07	1.54E+07	2.05E+07		9.22E+06	1.57E+07	1.26E+07	1.11E+07		24.02	24.04	24.52		23.29	24.12	23.5	23.17		24.04	23.71	23.34	0.284351	0.583755	0.235742
1610	P25205	DNA replic	MCM3	37	51.2	3.13E+08	2.93E+08	1.82E+08	2.37E+08	2.26E+08	2.20E+08	2.74E+08	1.35E+08	1.78E+08	28.26	28.57	27.89	27.92	27.93	28.09	27.96	26.77	26.64	28.26	27.93	26.77	0.340731	0.100007	0.726074
3092	Q9P2R3	Rabankyrir	ANKFY1	33	36.1	2.20E+08	1.60E+08	1.99E+08	1.77E+08	1.70E+08	1.61E+08	1.78E+08	2.20E+08	7.89E+07	27.83	27.65	28.1	27.47	27.5	27.7	27.41	27.47	25.7	27.83	27.5	27.41	0.224143	0.12218	1.006234
2169	Q13363-2	C-terminal·	CTBP1	8	21.4	1.45E+07	1.46E+07	1.10E+07	9.86E+06	9.94E+06	1.21E+07	9.97E+06	9.60E+06	6.05E+06	23.74	23.96	23.66	23.25	23.41	23.71	23.19	22.97	21.98	23.74	23.41	22.97	0.154675	0.234383	0.644123
2170	Q13371-2	Phosducin·	PDCL	3	14.2	2.52E+06	3.13E+06	3.99E+06	2.52E+06	2.56E+06	2.40E+06	2.41E+06			21.23	21.83	22.25	21.31	21.5	21.49	21.12			21.83	21.49	21.12	0.513571	0.10815	
2107	Q07812-5	Apoptosis ı	BAX	3	26.2		3.59E+06	2.82E+06		2.43E+06	2.68E+06			4.78E+06		22.04	21.73		21.44	21.66			21.6	21.89	21.55	21.6	0.219033	0.154661	
211	H0Y882	Forkhead k	FOXP1	5	13.1	1.65E+07	1.48E+07	1.63E+07	1.20E+07	1.58E+07	1.16E+07	2.57E+07			23.93	23.99	24.18	23.52	24.07	23.65	24.63			23.99	23.65	24.63	0.129105	0.289125	
2591	Q8NE71-2	ATP-bindin	ABCF1	16	23.3	8.93E+06	1.47E+07	1.91E+07	1.20E+07	1.18E+07	1.60E+07	2.85E+06	9.64E+06		23.06	23.97	24.4	23.52	23.63	24.16	21.39	22.98		23.97	23.63	22.18	0.688007	0.340023	1.125025
3195	Q9Y2L1	Exosome c	DIS3	9	11.5	6.60E+06	9.17E+06	8.78E+06	5.64E+06	7.18E+06	7.89E+06	6.64E+06	3.43E+06		22.58	23.3	23.34	22.44	22.96	23.18	22.63	21.52		23.3	22.96	22.08	0.424154	0.379039	0.784901
2012	P63010	AP-2 comp	AP2B1	20	25.9	2.70E+07	5.67E+07	3.51E+07	2.04E+07	2.86E+07	3.21E+07	1.64E+07	1.42E+07	4.25E+06	24.62	26.02	25.29	24.33	24.95	25.2	23.94	23.55	21.37	25.29	24.95	23.55	0.699947	0.446232	1.38308
2267	Q15113	Procollage	PCOLCE	3	8.2		1.16E+06	1.12E+06		7.76E+05	1.02E+06	1.14E+06	1.22E+06			20.46	20.47		19.81	20.43	19.99	20.02		20.46	20.12	20.01	0.004578	0.436189	0.015958
832	G3V1D1	Ferritin	FTH1	4	38.1	9.12E+06	1.48E+07	1.63E+07	1.29E+07	1.22E+07	1.09E+07	3.48E+06			23.08	23.99	24.18	23.65	23.69	23.58	21.67			23.99	23.65	21.67	0.584577	0.058037	
1583	P22102	Trifunction	GART	22	29.3	5.55E+07	5.44E+07	5.38E+07	4.48E+07	4.50E+07	4.61E+07	4.19E+07	4.10E+07	2.65E+07	25.69	25.92	25.96	25.55	25.58	25.71	25.31	25.09	24.18	25.92	25.58	25.09	0.14389	0.085415	0.597716
1660	P30086	Phosphatic	PEBP1	10	64.2	3.45E+07	4.77E+07	4.33E+07	2.05E+07	3.54E+07	3.39E+07	1.82E+07	1.57E+07		25.03	25.66	25.58	24.34	25.24	25.28	24.09	23.71		25.58	25.24	23.9	0.347178	0.530157	0.270359
1094	O00154-6	Cytosolic a	ACOT7	7	31	1.42E+07	1.07E+07	1.05E+07	8.98E+06	9.00E+06	1.23E+07	8.04E+06	6.99E+06	6.45E+06	23.7	23.53	23.58	23.1	23.24	23.75	22.87	22.5	22.12	23.58	23.24	22.5	0.089611	0.342646	0.375312
2265	Q15084-3	Protein dis	PDIA6	10	28.4	5.74E+07	8.71E+07	8.26E+07	4.40E+07	7.11E+07	7.70E+07	6.66E+07	6.69E+07	3.93E+07	25.75	26.59	26.64	25.51	26.25	26.47	25.89	25.8	24.76	26.59	26.25	25.8	0.497707	0.504452	0.626926
175	A0A0A0MS	Exocyst coı	EXOC7	4	7.6	3.12E+06	3.06E+06	3.29E+06	2.82E+06	3.47E+06	2.08E+06	5.00E+06	3.69E+06	4.41E+06	21.55	21.79	21.95	21.45	21.92	21.3	22.2	21.62	21.42	21.79	21.45	21.62	0.201532	0.322868	0.407217
443	C9J5N1	Alanyl-tRN.	PTGES3L-A	4	9.3	2.51E+06	2.34E+06	2.51E+06		1.75E+06	1.85E+06	1.53E+06	2.26E+06	2.21E+06	21.23	21.42	21.55		21	21.14	20.46	20.94	20.46	21.42	21.07	20.46	0.165277	0.098747	0.274832
1514	P13804	Electron tr	ETFA	11	45	1.53E+07	2.83E+07	2.42E+07	1.08E+07	2.00E+07	2.14E+07	1.93E+07	2.03E+07	1.48E+07	23.81	24.91	24.77	23.36	24.43	24.56	24.19	24.11	23.43	24.77	24.43	24.11	0.599134	0.660566	0.418167
2105	Q07065	Cytoskelet	CKAP4	16	34.4	3.89E+07	3.53E+07	3.74E+07	2.65E+07	2.83E+07	3.07E+07	2.55E+07	1.97E+07	2.30E+07	25.19	25.28	25.42	24.72	24.93	25.13	24.61	24.05	24.01	25.28	24.93	24.05	0.116885	0.204041	0.337031
366	G3V4W0	Heterogen	HNRNPC	10	33.6	1.65E+08	1.14E+08	9.22E+07	1.23E+08	9.22E+07	9.14E+07	1.05E+08	1.12E+08	5.70E+07	27.41	27.04	26.83	27.01	26.68	26.7	26.68	26.48	25.23	27.04	26.7	26.48	0.290932	0.183025	0.788124
1220	O60427	Fatty acid (	FADS1	5	14.6	5.17E+06	6.39E+06	5.63E+06	5.35E+06	4.89E+06	6.29E+06	6.61E+06	5.19E+06	6.58E+06	22.21	22.79	22.77	22.35	22.42	22.84	22.63	22.06	22.15	22.77	22.42	22.15	0.327804	0.265596	0.30494
2754	Q96KP1	Exocyst coı	EXOC2	12	15.2	5.34E+06	1.01E+07	9.14E+06	7.48E+06	7.64E+06	8.55E+06	4.84E+06	6.54E+06	3.29E+06	22.26	23.45	23.39	22.87	23.05	23.27	22.15	22.39	21.05	23.39	23.05	22.15	0.671395	0.199771	0.714589
1271	O75533	Splicing fac	SF3B1	26	26.8	8.10E+07	8.73E+07	8.65E+07	6.23E+07	7.60E+07	6.76E+07	7.01E+07	2.55E+07	8.66E+06	26.28	26.6	26.69	25.97	26.37	26.26	25.98	24.42	22.56	26.6	26.26	24.42	0.219829	0.208563	1.710715
2688	Q93009-3	Ubiquitin c	USP7	15	17.7	1.24E+07	2.01E+07	2.04E+07	1.41E+07	1.61E+07	1.49E+07	9.96E+06	2.02E+07		23.54	24.4	24.51	23.78	24.1	24.05	23.19	24.1		24.4	24.05	23.65	0.53288	0.175491	0.644469
2842	Q9BSL1	Ubiquitin-a	UBAC1	4	8.1	3.87E+06	6.80E+06	5.44E+06	4.39E+06	5.20E+06	4.48E+06	6.40E+06	6.27E+06		21.85	22.87	22.71	22.07	22.52	22.36	22.57	22.34		22.71	22.36	22.46	0.548578	0.229117	0.166942
1012	J3KR97	Tubulin-sp	TBCD	10	9.7	7.31E+06	8.88E+06	7.16E+06	5.54E+06	5.93E+06	7.50E+06	4.54E+06	6.89E+06		22.73	23.25	23.04	22.41	22.69	23.1	22.08	22.47		23.04	22.69	22.28	0.261337	0.350037	0.275101
3242	Q9Y5B9	FACT comp	SUPT16H	21	25.2	1.71E+08	1.77E+08	1.68E+08	1.62E+08	9.81E+07	1.42E+08	1.77E+08	1.40E+08	1.53E+08	27.46	27.82	27.73	27.38	26.79	27.5	27.4	26.84	26.47	27.73	27.38	26.84	0.189272	0.378733	0.467318
1217	O60313	Dynamin-li	OPA1	12	17.7		8.94E+06	5.13E+06	5.90E+06	5.40E+06	5.35E+06	8.50E+06				23.26	22.6	22.5	22.58	22.62	22.95			22.93	22.58	22.95	0.470308	0.060603	
1596	P23381-2	Tryptophai	WARS	31	75.1	9.02E+08	1.04E+09	8.17E+08	8.76E+08	1.05E+09	8.51E+08	2.31E+09	1.76E+09	1.14E+09	29.89	30.61	30.26	29.85	30.24	29.91	31.04	30.71	29.47	30.26	29.91	30.71	0.364203	0.206201	0.831442
1752	P40763	Signal tran	STAT3	48	65.7	1.86E+09	1.96E+09	1.93E+09	2.73E+09	2.94E+09	2.97E+09	4.97E+09	4.24E+09	2.07E+09	31.5	31.91	32.06	31.33	31.56	31.81	31.88	31.44	29.95	31.91	31.56	31.44	0.292617	0.237312	1.015023
2624	Q8TEX9	Importin-4	IPO4	10	12.6	7.88E+06	8.63E+06	8.31E+06	5.74E+06	6.64E+06	6.96E+06	1.24E+07	8.27E+06	1.04E+07	22.83	23.21	23.27	22.47	22.85	22.99	23.48	22.76	22.87	23.21	22.85	22.87	0.236129	0.269083	0.387661
1602	P23921	Ribonuclec	RRM1	17	28.9	4.40E+07	6.36E+07	7.08E+07	3.59E+07	5.24E+07	5.61E+07	4.67E+07	3.05E+07	5.61E+06	25.39	26.18	26.45	25.2	25.83	25.95	25.44	24.72	21.87	26.18	25.83	24.72	0.548154	0.400667	1.886758
872	H0Y512	Adipocyte	APMAP	3	6.6	2.62E+06	3.48E+06	4.67E+06		2.66E+06	2.76E+06	2.84E+06	2.32E+06		21.28	22	22.46		21.58	21.72	21.38	20.98		22	21.65	21.18	0.595889	0.098579	0.285403
472	C9JP16	Cartilage-a	CRTAP	6	18.2	1.80E+07	1.67E+07	1.37E+07	1.35E+07	1.16E+07	1.47E+07	1.19E+07	1.42E+07	1.13E+07	24.07	24.16	23.93	23.71	23.61	24.03	23.41	23.55	23.01	24.07	23.71	23.41	0.113998	0.220674	0.282541
3104	Q9UBQ5-2	Eukaryotic	EIF3K	7	38.4	6.42E+07	6.86E+07	6.46E+07	5.84E+07	6.44E+07	5.14E+07	7.26E+07	7.10E+07	4.12E+07	25.88	26.29	26.25	25.89	26.16	25.85	26.04	25.86	24.84	26.25	25.89	25.86	0.22658	0.169384	0.646363
2496	Q7Z406	Myosin-14	MYH14	2	5.2	5.40E+07	6.70E+07	5.39E+07	5.16E+07	4.12E+07	4.30E+07				25.66	26.25	25.97	25.75	25.44	25.61				25.97	25.61		0.294159	0.150649	
2850	Q9BTV4	Transmem	TMEM43	4	9.8	6.10E+06	4.39E+06	4.31E+06	4.64E+06	3.67E+06	3.10E+06	5.29E+06	6.01E+06	4.81E+06	22.47	22.32	22.36	22.15	22	21.86	22.3	22.28	21.61	22.36	22	22.28	0.078405	0.144974	0.392061
1491	P11586	C-1-tetrahy	MTHFD1	34	44.5	1.27E+08	1.34E+08	1.30E+08	1.18E+08	1.17E+08	1.08E+08	9.50E+07	6.13E+07	7.79E+07	26.97	27.35	27.35	26.91	26.99	27.02	26.52	25.68	25.68	27.35	26.99	25.68	0.220112	0.058557	0.485401
1679	P31350	Ribonuclec	RRM2	4	10.5	5.62E+06	5.36E+06	3.60E+06	4.11E+06	3.18E+06	5.16E+06	4.95E+06	4.65E+06	4.51E+06	22.33	22.56	22.1	21.97	21.81	22.57	22.19	21.94	21.47	22.33	21.97	21.94	0.230538	0.401129	0.364611
2139	Q12874	Splicing fac	SF3A3	5	15.4	8.56E+06	6.25E+06	8.95E+06	5.10E+06	5.66E+06	5.76E+06	3.86E+06			22.99	22.76	23.35	22.3	22.63	22.73	21.83			22.99	22.63	21.83	0.299584	0.222814	
85	A0A087WY	Unconvent	MYO5A	24	14.5	1.39E+07	1.63E+07	1.13E+07	1.18E+07	9.56E+06	8.31E+06	1.52E+07	1.26E+07	8.84E+06	23.67	24.11	23.7	23.49	23.34	23.24	23.83	23.38	22.59	23.7	23.34	23.38	0.246122	0.126719	0.627582
2272	Q15276-2	Rab GTPas	RABEP1	3	6.2		9.11E+06	8.56E+06		6.67E+06	7.00E+06					23.29	23.3		22.87	23				23.3	22.94		0.004173	0.097094	
1207	O43865-2	Putative ac	AHCYL1	4	8.7		8.98E+06	6.99E+06		6.17E+06	6.02E+06	6.00E+06				23.27	23		22.76	22.79	22.49			23.13	22.77	22.49	0.193511	0.016699	
1027	J3QQJ0	SAP30-binc	SAP30BP	5	18.5	7.68E+06	4.75E+06	5.24E+06	6.31E+06	2.86E+06	4.15E+06	4.53E+06			22.8	22.42	22.64	22.61	21.69	22.27	22.08			22.64	22.27	22.08	0.188983	0.46507	
1742	P38919	Eukaryotic	EIF4A3	16	43.3	1.13E+08	1.03E+08	1.21E+08	8.52E+07	7.25E+07	7.95E+07	4.63E+07	5.87E+07	4.16E+07	26.84	26.86	27.22	26.49	26.28	26.52	25.42	25.62	24.86	26.86	26.49	25.42	0.210777	0.130505	0.39031
854	G5E9W7	28S ribosoı	MRPS22	7	25.4	1.23E+07	1.02E+07	7.92E+06	8.31E+06	8.14E+06	8.59E+06	7.12E+06	8.42E+06	6.10E+06	23.52	23.47	23.2	22.98	23.11	23.28	22.71	22.79	22	23.47	23.11	22.71	0.172296	0.149467	0.431281
1950	P61160	Actin-relat	ACTR2	12	31.2	3.48E+07	4.19E+07	3.81E+07	3.27E+07	2.46E+07	3.09E+07	3.36E+07	4.21E+07	2.37E+07	25.04	25.54	25.44	25.07	24.68	25.15	24.99	25.12	24.06	25.44	25.07	24.99	0.264545	0.252122	0.579934
1637	P28066	Proteasom	PSMA5	4	23.2		4.65E+06	3.40E+06		2.15E+06	4.61E+06		2.92E+06			22.39	21.98		21.24	22.4		21.32		22.19	21.82	21.32	0.287636	0.820094	
3189	Q9Y281	Cofilin-2	CFL2	5	54.2	4.93E+06	1.07E+07	7.19E+06	5.45E+06	5.92E+06	6.16E+06				22.16	23.53	23.05	22.38	22.69	22.82				23.05	22.69		0.693992	0.224026	
3033	Q9NV70-2	Exocyst coı	EXOC1	13	16.8	1.15E+07	1.07E+07	9.45E+06	8.89E+06	6.96E+06	1.22E+07	7.31E+06	6.31E+06	3.35E+06	23.44	23.53	23.44	23.08	22.94	23.73	22.74	22.35	21.07	23.44	23.08	22.35	0.050198	0.419627	0.875321
1667	P30520	Adenylosu	ADSS	9	28.5	7.10E+06	1.19E+07	1.08E+07	5.83E+06	9.10E+06	1.17E+07	7.71E+06	8.54E+06		22.69	23.7	23.63	22.49	23.26	23.66	22.82	22.81		23.63	23.26	22.81	0.563015	0.594709	0.008606
1042	K7EJE8	Lon protea	LONP1	20	32.3	1.72E+07	2.71E+07	2.24E+07	1.55E+07	1.94E+07	1.73E+07	2.38E+07	2.58E+07		24.01	24.86	24.66	23.91	24.37	24.29	24.49	24.44		24.66	24.29	24.47	0.44368	0.245339	0.031823
2359	Q58FF8	Putative he	HSP90AB2I	2	21	1.00E+07	4.90E+07	6.91E+07	1.85E+07	3.71E+07	5.29E+07	2.32E+07	4.06E+07		23.24	25.7	26.39	24.2	25.33	25.89	24.47	25.07		25.7	25.33	24.77	1.655978	0.863583	0.426813
1312	O95163	Elongator (	IKBKAP	19	20.5	1.02E+07	1.64E+07	1.51E+07	1.19E+07	1.23E+07	1.34E+07	1.28E+07	1.17E+07	5.12E+06	23.27	24.12	24.07	23.5	23.7	23.89	23.54	23.26	21.74	24.07	23.7	23.26	0.479027	0.190449	0.970612
3250	Q9Y5S9-2	RNA-bindir	RBM8A	6	42.2	3.13E+07	2.35E+07	2.89E+07	2.41E+07	1.67E+07	2.10E+07	1.56E+07	1.87E+07		24.92	24.64	24.98	24.56	24.17	24.55	23.85	23.96		24.92	24.55	23.91	0.183489	0.223447	0.078577
640	E7EQB2	Lactotrans	LTF	3	7	9.35E+06		6.99E+05	1.22E+06	2.81E+06		1.34E+06			23.12		19.58	20.3	21.67		20.19			21.35	20.98	20.19	2.50503	0.970502	
3165	Q9UNE7	E3 ubiquiti	STUB1	5	18.8	5.47E+06	6.71E+06	5.58E+06	3.96E+06	4.72E+06	5.82E+06	4.44E+06	2.13E+06	4.53E+06	22.29	22.85	22.75	21.93	22.38	22.75	22.05	20.91	21.49	22.75	22.38	21.49	0.298641	0.410705	0.570282
1004	J3KPT4	TraB doma	TRABD	2	5.3		2.87E+06	2.71E+06		2.23E+06			2.82E+06			21.69	21.66		21.3			21.29		21.67	21.3	21.29	0.023158		
164	A0A0A0MI	Sorting ne›	SNX6	7	17.9	5.00E+06	1.27E+07	7.62E+06	5.20E+06	6.20E+06	8.24E+06	5.02E+06	6.44E+06		22.18	23.79	23.15	22.32	22.78	23.23	22.21	22.37		23.15	22.78	22.29	0.810458	0.454536	0.113367
3161	Q9UMX0-2	Ubiquilin-1	UBQLN1	8	18.7	5.88E+06	6.99E+06	8.96E+06		5.10E+06	5.47E+06	5.13E+06			22.41	22.94	23.36		22.49	22.65	22.24			22.94	22.57	22.24	0.476211	0.114929	
1292	O75976	Carboxype	CPD	8	6.7	3.55E+06	3.17E+06	3.27E+06	2.68E+06	2.68E+06	2.38E+06	5.02E+06	4.04E+06		21.71	21.86	21.94	21.4	21.59	21.49	22.21	21.75		21.86	21.49	21.98	0.115946	0.092314	0.324948
2097	Q06124	Tyrosine-p	PTPN11	9	17.4	9.64E+06	9.27E+06	1.16E+07	8.05E+06	6.53E+06	8.80E+06	1.01E+07	8.38E+06		23.17	23.32	23.74	22.94	22.83	23.3	23.21	22.78		23.32	22.94	23	0.295249	0.244589	0.302766
1904	Q5T4U8	Geranylger	RABGGTB	2	12.5	5.71E+06	3.39E+06	3.26E+06		2.65E+06	2.55E+06				22.36	21.96	21.94		21.57	21.6				21.96	21.58		0.238092	0.023356	

2753	Q96KB5	Lymphokin	PBK	6	22	1.40E+07	1.41E+07	1.55E+07	1.38E+07	9.78E+06	1.06E+07	1.35E+07	1.51E+07	1.27E+07	23.68	23.92	24.11	23.74	23.37	23.55	23.61	23.64	23.18	23.92	23.55	23.61	0.214169	0.18815	0.258958
2918	Q9H0U4	Ras-relatec	RAB1B	6	40.3	4.48E+07	5.19E+07	5.56E+07	4.18E+07	5.06E+07	3.87E+07	5.44E+07	4.28E+07	5.09E+07	25.41	25.84	26.01	25.43	25.79	25.46	25.68	25.14	25.12	25.84	25.46	25.14	0.308438	0.195011	0.315308
2928	Q9H2M9	Rab3 GTPa	RAB3GAP2	32	26	8.54E+07	8.88E+07	6.72E+07	7.49E+07	5.25E+07	5.73E+07	6.01E+07	5.76E+07	1.78E+07	26.35	26.65	26.33	26.26	25.83	25.98	25.81	25.59	23.69	26.35	25.98	25.59	0.176065	0.216481	1.16786
1397	P05109	Protein S1(	S100A8	3	31.2	2.96E+07	6.01E+06	3.35E+06	7.16E+06	3.21E+06		7.68E+06	1.11E+07		24.8	22.69	21.97	22.8	21.83		22.8	23.17		22.69	22.31	22.98	1.470603	0.687851	0.262408
2811	Q99805	Transmem	TM9SF2	5	7.4	2.85E+06	3.90E+06	3.87E+06		2.97E+06	3.14E+06	2.21E+06			21.41	22.19	22.22		21.73	21.89	20.98			22.19	21.81	20.98	0.461069	0.113688	
674	E9PC44	Protein tra	SEC24D	3	12.2		1.54E+06	2.60E+06			1.57E+06					20.87	21.61			20.86				21.24	20.86		0.528471		
886	H0Y8X4	2-deoxynu	DNPH1	4	13.6		9.36E+06	1.67E+07			9.29E+06					23.34	24.22			23.4				23.78	23.4		0.624861		
2034	P78371	T-complex	CCT2	16	46.9	7.48E+07	7.24E+07	7.21E+07	6.24E+07	6.45E+07	4.94E+07	7.00E+07	3.25E+07	1.04E+08	26.17	26.36	26.46	25.98	26.17	25.77	25.98	24.78	26.08	26.36	25.98	25.98	0.150587	0.200343	0.719162
905	H0YK42	Sorting ne	SNX1	2	13.5		2.47E+06	2.91E+06		1.91E+06	2.24E+06	2.82E+06				21.5	21.77		21.09	21.43	21.36			21.64	21.26	21.36	0.190865	0.240934	
1578	P21912	Succinate (	SDHB	4	16.8	3.42E+06	5.45E+06	6.75E+06	3.01E+06	4.27E+06	4.82E+06	2.94E+06	3.21E+06		21.67	22.59	22.94	21.55	22.21	22.46	21.44	21.45		22.59	22.21	21.45	0.658113	0.470184	0.007406
826	F8WCF6	Actin-relat	ARPC4-TTL	5	27.6	4.10E+07	5.59E+07	4.91E+07	4.31E+07	3.40E+07	4.57E+07	3.88E+07	4.99E+07	3.76E+07	25.27	25.98	25.86	25.47	25.17	25.69	25.22	25.37	24.72	25.86	25.47	25.22	0.382214	0.261228	0.342151
2629	Q8WUA2	Peptidyl-pr	PPIL4	10	30.1	1.47E+07	2.03E+07	2.08E+07	1.78E+07	1.39E+07	1.47E+07	2.02E+07	1.26E+07	7.90E+06	23.76	24.41	24.54	24.12	23.88	24.03	24.28	23.38	22.44	24.41	24.03	23.38	0.420377	0.120523	0.917537
144	A0A087X2I	26S protea	PSMC6	16	49.9	1.39E+08	1.30E+08	1.01E+08	9.97E+07	9.39E+07	9.73E+07	9.79E+07	1.51E+08	6.75E+07	27.12	27.32	26.96	26.69	26.73	26.8	26.57	26.93	25.47	27.12	26.73	26.57	0.184082	0.052445	0.760772
130	A0A087X1I	Ribonuclea	RPP40	3	15		2.73E+06	3.50E+06	2.12E+06	2.77E+06	2.25E+06	2.42E+06				21.61	22.03	21.03	21.64	21.43	21.13			21.82	21.43	21.13	0.297547	0.306043	
1356	Q5T9B7	Adenylate	AK1	2	11.9	3.78E+06	5.88E+06	1.81E+06	2.64E+06	3.36E+06	2.27E+06	1.88E+06	1.73E+06		21.82	22.66	21.15	21.39	21.88	21.44	20.74	20.51		21.82	21.44	20.63	0.759081	0.272025	0.157514
1107	O00303	Eukaryotic	EIF3F	9	31.4	1.35E+08	1.22E+08	1.05E+08	1.09E+08	8.11E+07	8.74E+07	1.29E+08	1.46E+08	7.85E+07	27.03	27.24	27.03	26.8	26.47	26.65	26.97	26.91	25.69	27.03	26.65	26.91	0.119881	0.166903	0.720915
2194	Q13813-3	Spectrin al	SPTAN1	2	64.5	4.85E+06	9.41E+06	8.86E+06	6.73E+06	7.92E+06	6.83E+06		8.85E+06		22.14	23.34	23.34	22.71	23.08	22.95		22.85		23.34	22.95	22.85	0.694533	0.189719	
2184	Q13564-3	NEDD8-act	NAE1	5	18.2	2.67E+06	3.24E+06	3.38E+06	2.89E+06	2.38E+06	2.59E+06	3.10E+06	2.62E+06		21.31	21.89	21.98	21.5	21.41	21.63	21.51	21.15		21.89	21.5	21.33	0.360238	0.110077	0.256601
1328	O95486	Protein tra	SEC24A	7	8.5	4.81E+06	4.60E+06	7.10E+06	3.28E+06	3.65E+06	4.02E+06	2.80E+06	4.64E+06		22.12	22.38	23.02	21.66	22	22.22	21.35	21.93		22.38	22	21.64	0.462958	0.280148	0.411101
1947	P61088	Ubiquitin-c	UBE2N	3	23.7	6.61E+06	1.04E+07	1.47E+07	7.65E+06	8.03E+06	1.22E+07	8.38E+06			22.59	23.49	24.02	22.89	23.1	23.73	22.93			23.49	23.1	22.93	0.725986	0.435726	
2114	Q08378-2	Golgin sub	GOLGA3	37	33.5	6.14E+07	5.33E+07	4.49E+07	4.54E+07	3.91E+07	3.78E+07	4.50E+07	2.51E+07	8.75E+06	25.81	25.89	25.67	25.58	25.37	25.42	25.38	24.4	22.58	25.81	25.42	24.4	0.10906	0.109229	1.422233
2984	Q9HC36	rRNA meth	RNMTL1	3	6.7		3.76E+06			3.01E+06	2.80E+06		3.66E+06			22.13			21.74	21.74		21.61		22.13	21.74	21.61		0.000531	
1516	P13861-2	cAMP-depe	PRKAR2A	12	42.9	1.42E+07	1.83E+07	1.56E+07	1.37E+07	1.20E+07	1.45E+07	1.49E+07	1.55E+07	6.69E+06	23.7	24.29	24.12	23.73	23.66	24.02	23.79	23.68	22.17	24.12	23.73	23.68	0.299375	0.189865	0.90951
1941	P60842	Eukaryotic	EIF4A1	27	62.8	4.68E+08	6.71E+08	6.21E+08	5.50E+08	5.51E+08	6.35E+08	4.96E+08	4.69E+08	4.50E+08	28.86	29.65	29.69	29.11	29.26	29.53	28.9	28.33	28.1	29.65	29.26	28.33	0.468578	0.215351	0.413408
1790	P46063	ATP-deper	RECQL	4	8	3.26E+06	3.19E+06	3.52E+06	2.84E+06	2.06E+06	2.54E+06	2.65E+06	1.85E+06		21.61	21.87	22.05	21.47	21.2	21.59	21.25	20.65		21.87	21.47	20.95	0.220958	0.201364	0.422868
1551	P18124	60S ribosoı	RPL7	14	44.8	3.14E+08	2.71E+08	2.73E+08	2.65E+08	2.18E+08	2.76E+08	2.99E+08	2.68E+08	3.47E+07	28.27	28.47	28.65	28.07	27.85	28.4	28.06	27.64	24.63	28.47	28.07	27.64	0.188917	0.27742	1.869496
0	A0A024QZ	Serpin B6	SERPINB6	10	35.8	3.47E+07	3.29E+07	3.19E+07	2.79E+07	2.48E+07	2.25E+07	3.54E+07	3.83E+07	3.07E+07	25.03	25.13	25.1	24.79	24.71	24.64	25.09	25.01	24.39	25.1	24.71	25.01	0.053154	0.075005	0.383323
1	A0A024R4I	Vigilin	HDLBP	26	25.1	2.71E+07	3.85E+07	3.83E+07	2.49E+07	3.03E+07	2.93E+07	1.89E+07	2.35E+07	6.20E+06	24.63	25.41	25.44	24.63	25.01	25.06	24.16	24.31	22.06	25.41	25.01	24.16	0.460013	0.231834	1.258462
2216	Q14244	Ensconsin	MAP7	8	10.8	9.67E+06	1.07E+07	4.53E+06	6.51E+06	9.15E+06	5.99E+06	7.54E+06	7.43E+06		23.18	23.53	22.42	22.66	23.27	22.78	22.77	22.59		23.18	22.78	22.68	0.564513	0.322147	0.125867
757	F5GZS6	4F2 cell-su	SLC3A2	27	46.7	4.40E+08	4.28E+08	4.08E+08	3.93E+08	4.70E+08	3.55E+08	3.73E+08	3.31E+08	3.70E+08	28.77	29.12	29.17	28.54	29.01	28.71	28.35	27.9	27.69	29.12	28.71	27.9	0.212833	0.236318	0.335687
235	A0A0C4DG	Golgin sub	GOLGA2	4	7.1	3.44E+06	2.95E+06	3.50E+06	2.32E+06	2.28E+06	2.73E+06	2.67E+06	2.46E+06		21.68	21.74	22.03	21.16	21.34	21.7	21.28	21.06		21.74	21.34	21.17	0.18636	0.275414	0.155193
1844	P49773	Histidine tı	HINT1	6	70.6	2.21E+07	2.14E+07	3.85E+07	1.76E+07	1.35E+07	3.23E+07	1.82E+07		1.99E+07	24.34	24.5	25.45	24.1	23.84	25.22	24.09		23.85	24.5	24.1	23.97	0.599633	0.731296	0.168684
876	H0Y5R6	Uroporphy	UROD	2	10.1	1.34E+06	2.22E+06	1.49E+06		1.13E+06	1.14E+06	1.41E+06			20.27	21.36	20.83		20.35	20.51	20.35			20.83	20.43	20.35	0.545486	0.11398	
42	A0A087WX	Retrotrans	PEG10	13	28.7	6.99E+07	5.67E+07	5.67E+07	4.11E+07	4.59E+07	5.24E+07	4.07E+07	2.52E+07	5.41E+06	26.02	26.02	26.04	25.4	25.62	25.88	25.26	24.41	21.81	26.02	25.62	24.41	0.014086	0.239648	1.795222
3200	Q9Y2T3	Guanine de	GDA	5	11.9	8.37E+06	1.10E+07	1.24E+07	8.47E+06	8.45E+06	9.47E+06	9.88E+06	9.98E+06	8.08E+06	22.96	23.58	23.81	23.01	23.17	23.42	23.18	23.02	22.46	23.58	23.17	23.02	0.441572	0.205788	0.374691
2447	Q6PI48	Aspartate-	DARS2	4	9.3	4.36E+06	6.01E+06	7.03E+06	4.33E+06	4.44E+06	6.61E+06	4.75E+06	3.64E+06		22	22.69	23.01	22.06	22.29	22.91	22.12	21.6		22.69	22.29	21.86	0.513489	0.43848	0.370725
2795	Q99459	Cell divisio	CDC5L	7	12.5	3.66E+06	7.38E+06	5.23E+06		4.00E+06	4.33E+06	4.44E+06	4.64E+06		21.76	23.01	22.63		22.12	22.33	22.05	21.93		22.63	22.22	21.99	0.636814	0.146221	0.084296
2253	Q15021	Condensin	NCAPD2	25	24.1	4.05E+07	3.82E+07	3.57E+07	2.62E+07	3.04E+07	2.71E+07	1.70E+07	1.82E+07	9.13E+06	25.25	25.38	25.32	24.71	25.01	24.92	24	23.92	22.63	25.32	24.92	23.92	0.064347	0.157217	0.767183
881	H0Y750	Renin rece	ATP6AP2	4	16	3.94E+06	3.01E+06	3.09E+06		2.61E+06	2.18E+06	2.98E+06			21.87	21.76	21.87		21.54	21.39	21.47			21.87	21.46	21.47	0.063835	0.109368	
2250	Q15008	26S protea	PSMD6	16	37	4.47E+07	6.31E+07	5.46E+07	4.28E+07	4.47E+07	4.20E+07	3.95E+07	4.36E+07	2.73E+07	25.41	26.16	25.98	25.46	25.57	25.57	25.23	25.15	24.23	25.98	25.57	25.15	0.392863	0.062859	0.556059
846	G3XAH6	Poly(A) pol	PAPOLA	7	12.7	4.33E+06	5.23E+06	7.35E+06	4.18E+06	4.18E+06	3.67E+06	4.17E+06	3.27E+06		22	22.52	23.09	22	22.17	22.11	21.96	21.47		22.52	22.11	21.72	0.545871	0.081454	0.346819
2799	Q99536	Synaptic ve	VAT1	8	29	7.68E+06	8.87E+06	8.05E+06			6.21E+06	6.47E+06			22.8	23.24	23.24			22.83	22.59			23.24	22.83	22.59	0.255263		
378	B4DKY1	Cysteine--t	CARS	10	16.8	7.83E+06	9.72E+06	1.08E+07	5.91E+06	7.25E+06	7.62E+06	1.06E+07	7.42E+06		22.83	23.39	23.63	22.51	22.98	23.13	23.26	22.59		23.39	22.98	22.92	0.41494	0.325252	0.475189
2840	Q9BSJ2	Gamma-tu	TUBGCP2	14	20.1	1.15E+07	1.66E+07	8.35E+06	8.60E+06	6.45E+06	8.27E+06	1.61E+07	1.32E+07		23.44	24.14	23.27	23.03	22.82	23.23	23.89	23.44		23.44	23.03	23.66	0.460465	0.208514	0.317938
2942	Q9H479	Fructosam	FN3K	4	15.5		2.06E+06	1.61E+06	1.72E+06	1.29E+06	1.46E+06					21.28	20.92	20.69	20.57	20.77				21.1	20.69		0.253613	0.103897	
1743	P39019	40S ribosoı	RPS19	8	43.4	3.26E+07	4.96E+07	6.65E+07	2.82E+07	3.68E+07	5.66E+07	3.55E+07	2.00E+07	2.84E+07	24.97	25.73	26.31	24.8	25.32	25.96	25.1	24.09	24.28	25.73	25.32	24.28	0.673661	0.580256	0.535609
1074	M0QY97	Zinc finger	ZC3H4	4	9		2.52E+06	2.25E+06	1.82E+06	1.85E+06	2.58E+06					21.53	21.43	20.78	21.06	21.62				21.48	21.06		0.07115	0.427552	
1299	O94826	Mitochond	TOMM70A	6	12	4.24E+06	7.23E+06	8.98E+06	4.52E+06	5.37E+06	8.06E+06	4.49E+06	4.95E+06		21.98	22.98	23.36	22.1	22.57	23.21	22.07	22.02		22.98	22.57	22.04	0.715573	0.554583	0.036347
1966	P61978	Heterogen	HNRNPK	20	56.2	1.68E+09	1.26E+09	1.55E+09	1.80E+09	1.15E+09	1.41E+09	1.30E+09	1.24E+09	9.33E+08	31.29	31.07	31.43	31.02	30.49	30.88	30.51	30.06	29.13	31.29	30.88	30.06	0.181636	0.275417	0.704986
3110	Q9UBV2	Protein sel	SEL1L	3	6.4		4.16E+06	3.75E+06	3.67E+06	4.57E+06	2.45E+06	5.98E+06	5.25E+06			22.27	22.17	21.8	22.34	21.52	22.48	22.07		22.22	21.8	22.27	0.068832	0.412645	0.295466
1526	P14923	Junction pl	JUP	14	22	7.10E+06	1.89E+07	2.04E+07	2.23E+07	1.04E+07	1.36E+07	1.23E+07	1.24E+07	8.20E+06	22.69	24.32	24.51	24.48	23.47	23.91	23.46	23.36	22.48	24.32	23.91	23.36	0.999295	0.510199	0.538778
1608	P24844	Myosin reg	MYL9	4	62.2	1.00E+07	1.16E+07	9.58E+06	1.40E+07	7.71E+06	6.23E+06				23.24	23.66	23.47	23.76	23.06	22.83				23.47	23.06		0.209789	0.482752	
2036	P78527	DNA-deper	PRKDC	157	38.3	4.01E+08	8.75E+08	8.33E+08	8.98E+08	9.39E+08	6.31E+08	9.76E+08	9.21E+08	6.68E+08	28.63	30.32	30.35	29.9	29.98	29.52	29.84	29.64	28.57	30.32	29.9	29.64	0.986612	0.247008	0.684154
2156	Q13162	Peroxiredo	PRDX4	8	47.2	3.84E+07	3.96E+07	4.86E+07	3.14E+07	3.05E+07	5.08E+07	3.48E+07	3.40E+07		25.16	25.44	25.84	24.95	25.02	25.82	25.05	24.85		25.44	25.02	24.95	0.343016	0.483602	0.145317
462	C9JIF9	Acylamino-	APEH	10	16.7	9.95E+06	1.06E+07	1.22E+07	6.79E+06	8.00E+06	8.08E+06	1.44E+07	8.55E+06	7.36E+06	23.22	23.52	23.78	22.72	23.1	23.21	23.74	22.81	22.32	23.52	23.1	22.81	0.279768	0.259835	0.723812
135	A0A087X2	Protein CIF	KIAA1524	5	6.2	4.02E+06	3.46E+06	3.24E+06	2.12E+06	3.65E+06		2.84E+06			21.89	21.99	21.93	21.03	22		21.38			21.93	21.52	21.38	0.048918	0.679508	
1235	O60749-2	Sorting ne	SNX2	8	23.9	6.20E+06	8.11E+06	7.15E+06	5.41E+06	5.64E+06	7.62E+06	4.82E+06	5.47E+06	6.50E+06	22.49	23.13	23.04	22.37	22.62	23.13	22.14	22.15	22.13	23.04	22.62	22.14	0.343126	0.391592	0.008661
1337	O95782-2	AP-2 comp	AP2A1	16	20	1.09E+07	2.42E+07	1.76E+07	1.13E+07	1.39E+07	1.65E+07	1.51E+07	7.85E+06		23.36	24.68	24.3	23.42	23.88	24.2	23.82	22.69		24.3	23.88	23.26	0.67889	0.39369	0.799597
1352	P00441	Superoxide	SOD1	6	60.4	1.04E+07	1.32E+07	9.63E+06	6.94E+06	7.71E+06	7.76E+06	6.52E+06	7.27E+06		23.29	23.83	23.48	22.75	23.06	23.15	22.61	22.55		23.48	23.06	22.58	0.27458	0.209004	0.044314
1770	P42285	Superkiller	SKIV2L2	13	14.6	1.08E+07	1.37E+07	1.30E+07	7.40E+06	1.03E+07	1.24E+07	6.69E+06	6.21E+06		23.34	23.88	23.89	22.85	23.46	23.78	22.64	22.32		23.88	23.46	22.48	0.313759	0.468719	0.222722
2213	Q14195-2	Dihydropyı	DPYSL3	6	14		2.74E+06	2.53E+06		1.95E+06	1.97E+06	3.69E+06	2.70E+06			21.61	21.57		21.11	21.23	21.77	21.22		21.59	21.17	21.49	0.027658	0.084152	0.388964
2857	Q9BUQ8	Probable A	DDX23	17	23	2.29E+07	2.52E+07	2.13E+07	1.57E+07	1.65E+07	1.82E+07	8.47E+06	8.97E+06		24.39	24.75	24.58	23.94	24.16	24.39	22.95	22.87		24.58	24.16	22.91	0.177683	0.223558	0.05687
2812	Q99832	T-complex	CCT7	34	67.6	6.58E+08	6.93E+08	6.50E+08	5.22E+08	5.53E+08	5.39E+08	6.18E+08	5.45E+08	2.84E+08	29.37	29.7	29.83	28.96	29.27	29.35	29.07	28.63	27.25	29.7	29.27	28.63	0.235245	0.209625	0.949737
385	B4DY09	Interleukin	ILF2	13	45.5	4.79E+07	5.21E+07	4.97E+07	3.18E+07	4.04E+07	5.44E+07	4.51E+07	4.41E+07	3.19E+07	25.47	25.85	25.87	25.02	25.42	25.91	25.39	25.18	24.45	25.85	25.42	25.18	0.222539	0.447726	0.491434
842	G3V5R9	Kinesin ligl	KLC1	31	49.7	2.68E+08	2.91E+08	3.19E+08	2.53E+08	2.65E+08	2.80E+08	2.99E+08	2.77E+08	8.75E+07	28.06	28.55	28.86	28.03	28.12	28.43	28.06	27.69	25.91	28.55	28.12	27.69	0.406589	0.209925	1.147268
2723	Q96DI7	U5 small n	SNRNP40	7	35.3	5.68E+06	6.32E+06	8.06E+06		4.56E+06	4.46E+06				22.36	22.77	23.24		22.33	22.36				22.77	22.35		0.442211	0.019379	
1141	O14908	PDZ domai	GIPC1	4	17.4		4.76E+06	3.68E+06	3.79E+06	3.22E+06	3.94E+06					22.43	22.15	21.86	21.83	22.2				22.29	21.86		0.198531	0.203228	
2769	Q96PZ0	Pseudouric	PUS7	7	12.3	3.20E+06	3.70E+06	5.20E+06	3.27E+06	2.60E+06	3.73E+06	2.70E+06	2.80E+06		21.58	22.09	22.62	21.66	21.52	22.12	21.29	21.27		22.09	21.66	21.28	0.517588	0.312961	0.010581
2538	Q8IXM3	39S ribosoı	MRPL41	2	16.1	2.98E+06	3.68E+06	3.90E+06			2.64E+06	4.06E+06			21.5	22.08	22.23			21.65	21.92			22.08	21.65	21.92	0.381863		
1102	O00232-2	26S protea	PSMD12	16	32.3	3.78E+07	4.90E+07	4.62E+07	4.13E+07	3.59E+07	3.09E+07	3.18E+07	4.65E+07	2.99E+07	25.14	25.7	25.73	25.41	25.27	25.15	24.92	25.28	24.34	25.7	25.27	24.92	0.333739	0.130952	0.473687
346	B0QY89	Eukaryotic	EIF3L	22	36.9	2.07E+08	2.00E+08	2.02E+08	1.89E+08	1.57E+08	1.64E+08	1.35E+08	1.95E+08	8.81E+07	27.77	28.01	28.11	27.58	27.41	27.73	27.01	27.3	25.93	28.01	27.58	27.01	0.174955	0.162201	0.723598
2635	Q8WUX2	Putative gl	CHAC2	3	21.2	1.68E+06	2.85E+06	5.10E+06	2.12E+06	2.91E+06	1.98E+06				20.56	21.67	22.59	21.03	21.71	21.24				21.67	21.24		1.015943	0.346706	
985	I3L397	Eukaryotic	EIF5A	15	70.7	8.68E+07	1.22E+08	1.73E+08	7.74E+07	9.86E+07	1.44E+08	6.20E+07	8.66E+07	2.78E+07	26.43	27.24	27.8	26.3	26.81	27.54	25.84	26.19	24.27	27.24	26.81	25.84	0.690424	0.620253	1.020616
1115	O00461	Golgi integ	GOLIM4	3	5.7			1.42E+06	1.14E+06		1.03E+06						20.77	20.23		20.44				20.77	20.33			0.149962	
2144	Q12965	Unconvent	MYO1E	6	5.1	6.66E+06	5.20E+06	3.56E+06	4.32E+06	4.28E+06	3.62E+06		3.48E+06		22.6	22.51	22.08	22.06	22.21	22.08		21.54		22.51	22.08	21.54	0.278949	0.08342	
2068	Q01650	Large neut	SLC7A5	5	11.6	9.53E+07	8.63E+07	8.79E+07	1.12E+08	6.30E+07	6.46E+07	7.11E+07	4.49E+07	8.47E+07	26.62	26.57	26.72	26.84	26.12	26.18	25.99	25.21	25.82	26.62	26.18	25.82	0.074701	0.398509	0.408235
6	F5H796	Diablo hon	DIABLO	6	40.1	8.25E+06	8.98E+06	8.98E+06	7.31E+06	6.26E+06	7.24E+06	7.69E+06	7.98E+06	4.46E+06	22.94	23.27	23.36	22.84	22.79	23.05	22.81	22.71	21.45	23.27	22.84	22.71	0.22173	0.139176	0.757892

688	E9PF19	Transducin	TBL2	5	12.9	5.95E+06	6.16E+06	5.50E+06	5.99E+06	4.49E+06	4.03E+06		7.05E+06	5.28E+06	22.43	22.74	22.73	22.55	22.3	22.23		22.51	21.77	22.73	22.3	22.14	0.17671	0.169336	0.521918
1703	P35221	Catenin alp	CTNNA1	20	31.3	8.14E+06	2.61E+07	1.28E+07	8.86E+06	1.01E+07	1.04E+07	7.57E+06	5.99E+06		22.91	24.82	23.87	23.08	23.43	23.53	22.77	22.28		23.87	23.43	22.52	0.950896	0.239905	0.351577
1434	P08237-2	ATP-depen	PFKM	17	32.3	2.73E+07	4.36E+07	3.86E+07	2.52E+07	3.06E+07	4.38E+07	2.38E+07	2.58E+07	1.08E+07	24.64	25.59	25.46	24.65	25.03	25.65	24.49	24.44	22.94	25.46	25.03	24.44	0.512946	0.50598	0.879178
2432	Q6NXE6-2	Armadillo r	ARMC6	5	12.6		4.92E+06	4.33E+06	4.14E+06	2.87E+06	3.79E+06	5.31E+06	4.48E+06			22.47	22.37	21.98	21.7	22.14	22.31	21.89		22.42	21.98	22.1	0.068903	0.223484	0.298941
2682	Q92947	Glutaryl-Co	GCDH	7	21.5	1.28E+07	1.08E+07	8.86E+06	1.20E+07	7.95E+06	7.54E+06	1.38E+07	1.44E+07	1.40E+07	23.58	23.55	23.34	23.52	23.09	23.11	23.68	23.58	23.34	23.55	23.11	23.58	0.131184	0.244246	0.173688
2255	Q15029-2	116 kDa U5	EFTUD2	28	39.4	4.85E+07	6.15E+07	5.99E+07	4.86E+07	4.66E+07	5.85E+07	3.55E+07	3.61E+07	6.87E+06	25.5	26.11	26.1	25.67	25.65	26.02	25.1	24.91	22.21	26.1	25.67	24.91	0.351176	0.208515	1.615714
1803	P47756-2	F-actin-cap	CAPZB	17	52.6	6.27E+07	9.25E+07	8.63E+07	7.40E+07	5.93E+07	7.15E+07	6.98E+07	7.18E+07	4.51E+07	25.84	26.7	26.68	26.25	26	26.35	25.97	25.89	24.95	26.68	26.25	25.89	0.490538	0.179422	0.568325
3117	Q9UGI8-2	Testin	TES	2	5.3			2.20E+06			1.68E+06						21.4			20.96				21.4	20.96				
1313	O95202	LETM1 anc	LETM1	6	11.5	7.13E+06	5.87E+06	7.33E+06	5.00E+06	4.03E+06	5.24E+06	5.83E+06	6.13E+06	7.53E+06	22.7	22.66	23.09	22.26	22.12	22.59	22.44	22.31	22.36	22.7	22.26	22.36	0.236322	0.241177	0.066491
2262	Q15057	Arf-GAP wi	ACAP2	14	22.1	2.39E+07	3.10E+07	3.10E+07	2.39E+07	3.28E+07	2.17E+07	2.99E+07	2.60E+07	6.29E+06	24.47	25.02	25.08	24.55	25.13	24.58	24.82	24.45	22.08	25.02	24.58	24.45	0.336641	0.327493	1.489514
1306	O94973	AP-2 comp	AP2A2	3	8.5			1.27E+06		9.20E+05	9.51E+05						20.68		20.13	20.37				20.68	20.25			0.169393	
428	C9J3L8	Translocon	SSR1	3	12.8	1.00E+07	1.01E+07	1.13E+07	8.50E+06	6.20E+06	9.48E+06				23.24	23.45	23.7	23.01	22.78	23.42				23.45	23.01		0.232425	0.325174	
143	A0A087X2I	E3 ubiquiti	HECTD1	13	6.5	6.09E+06	7.14E+06	7.43E+06	7.31E+06	4.17E+06	5.03E+06	4.30E+06	3.68E+06		22.46	22.96	23.11	22.84	22.16	22.52	22	21.61		22.96	22.52	21.81	0.339426	0.339191	0.275436
2135	Q12797-10	Aspartyl/as	ASPH	6	11.4	2.81E+06	3.71E+06	3.16E+06		2.38E+06	2.44E+06				21.38	22.1	21.91		21.41	21.52				21.91	21.46		0.374228	0.07486	
1634	P27695	DNA-(apur	APEX1	9	42.8	5.18E+06	1.15E+07	3.13E+07			8.01E+06				22.22	23.64	25.08			23.2				23.64	23.2		1.4333		
2166	Q13283	Ras GTPas	G3BP1	8	22.5	3.48E+07	3.99E+07	3.97E+07	2.86E+07	3.01E+07	3.50E+07	2.43E+07	2.75E+07		25.04	25.45	25.49	24.82	25	25.31	24.53	24.52		25.45	25	24.53	0.249638	0.248407	0.00818
1707	P35250	Replication	RFC2	12	39.5	1.95E+07	2.10E+07	2.01E+07	1.78E+07	1.54E+07	1.15E+07	1.62E+07	1.55E+07	1.58E+07	24.18	24.46	24.47	24.12	24.02	23.64	23.91	23.68	23.54	24.46	24.02	23.68	0.16361	0.254914	0.185818
259	A0A0G2JN	Glucosylce	GBA	2	8		1.94E+06	2.66E+06		1.70E+06						21.2	21.64		20.97					21.42	20.97		0.310177		
722	E9PMP7	LIM domai	LMO7	17	23.6	2.03E+07	1.67E+07	1.51E+07	1.18E+07	1.24E+07	1.56E+07	1.43E+07	8.57E+06		24.23	24.16	24.07	23.49	23.71	24.12	23.73	22.81		24.16	23.71	23.27	0.079441	0.316858	0.648261
349	B0QYW5	Peroxisom	SLC25A17	2	10.2			2.57E+06		2.12E+06	1.80E+06						21.59		21.23	21.07				21.59	21.15			0.107977	
2431	Q6NUQ4-2	Transmem	TMEM214	6	9	7.07E+06	5.42E+06	6.23E+06	4.95E+06	4.25E+06	5.58E+06	4.98E+06	4.52E+06	6.02E+06	22.69	22.58	22.87	22.24	22.19	22.69	22.2	21.9	21.98	22.69	22.24	21.98	0.14771	0.271988	0.157065
3006	Q9NQT5	Exosome c	EXOSC3	4	21.8	2.86E+06	4.33E+06	3.77E+06	3.13E+06	3.10E+06	2.80E+06	2.66E+06		4.04E+06	21.41	22.3	22.19	21.6	21.78	21.74	21.27		21.3	22.19	21.74	21.28	0.482287	0.094322	0.023548
203	F5GY88	Mediator c	MED24	4	6.2			2.09E+06	1.50E+06	1.62E+06	2.57E+06						21.32	20.49	20.87	21.61				21.32	20.87			0.568629	
1677	P31153	S-adenosyl	MAT2A	23	60.5	8.04E+08	8.42E+08	5.59E+08	6.50E+08	5.02E+08	6.11E+08	1.06E+09	1.08E+09	9.83E+08	29.66	30.27	29.61	29.22	29.12	29.48	30	29.94	29.23	29.66	29.22	29.94	0.369522	0.188159	0.428571
2331	Q29RF7	Sister chro	PDS5A	10	9.7	6.28E+06	1.19E+07	9.89E+06	6.81E+06	7.81E+06	8.50E+06	1.19E+07	7.30E+06		22.51	23.7	23.52	22.73	23.07	23.26	23.41	22.56		23.52	23.07	22.99	0.642333	0.269459	0.597217
1663	P30154-4	Serine/thre	PPP2R1B	3	20.5	2.66E+06	3.94E+06	4.72E+06	2.83E+06	3.04E+06	3.25E+06	2.51E+06			21.31	22.2	22.49	21.46	21.75	21.93	21.18			22.2	21.75	21.18	0.616052	0.236762	
2944	Q9H497-2	Torsin-3A	TOR3A	8	28	9.86E+06	6.67E+06	8.55E+06	5.42E+06	6.13E+06	7.57E+06	6.95E+06			23.21	22.85	23.3	22.37	22.76	23.12	22.68			23.21	22.76	22.68	0.238009	0.376317	
3206	Q9Y2Z0-2	Suppressor	SUGT1	5	20.1	5.27E+06	6.92E+06	7.76E+06	5.74E+06	4.36E+06	5.54E+06	5.58E+06	5.65E+06	5.03E+06	22.24	22.92	23.17	22.47	22.25	22.66	22.38	22.2	21.7	22.92	22.47	22.2	0.480187	0.207997	0.35699
652	E7ESC7	Macrophag	MAEA	7	15.4	1.82E+07	1.63E+07	1.49E+07	1.98E+07	1.06E+07	1.16E+07	2.19E+07	1.68E+07	1.40E+07	24.1	24.11	24.04	24.29	23.5	23.65	24.39	23.79	23.34	24.1	23.65	23.79	0.036764	0.422718	0.52555
1745	P39748	Flap endor	FEN1	9	37.9	1.65E+07	2.70E+07	2.87E+07	1.78E+07	1.98E+07	2.69E+07	2.04E+07	2.65E+07	1.46E+07	23.93	24.85	24.97	24.12	24.4	24.89	24.31	24.47	23.41	24.85	24.4	24.31	0.568644	0.392003	0.571321
3249	Q9Y5S2	Serine/thre	CDC42BPB	12	8.5	9.87E+06	1.12E+07	1.06E+07	9.18E+06	7.70E+06	8.40E+06	4.84E+06	4.13E+06		23.21	23.61	23.59	23.14	23.05	23.25	22.15	21.78		23.59	23.14	21.96	0.227106	0.101061	0.259203
1448	P09012	U1 small n	SNRPA	5	25.5	1.44E+07	1.89E+07	1.78E+07	1.52E+07	1.19E+07	1.32E+07	1.25E+07	1.46E+07	6.69E+06	23.72	24.32	24.33	23.89	23.64	23.87	23.49	23.61	22.17	24.32	23.87	23.49	0.347385	0.135313	0.800692
1439	P08559-3	Pyruvate d	PDHA1	11	38.7	2.29E+07	3.04E+07	2.54E+07	1.83E+07	1.94E+07	2.64E+07	2.08E+07	2.36E+07	1.47E+07	24.39	25	24.83	24.18	24.37	24.85	24.33	24.32	23.42	24.83	24.37	24.32	0.311617	0.343956	0.518312
2957	Q9H7D0	Dedicator c	DOCK5	9	6.2	5.17E+06	6.92E+06	5.82E+06	4.02E+06	4.58E+06	4.54E+06	4.15E+06	4.43E+06	4.63E+06	22.21	22.92	22.8	21.94	22.34	22.39	21.95	21.86	21.54	22.8	22.34	21.86	0.378251	0.243739	0.216599
3029	Q9NU22	Midasin	MDN1	26	6	2.27E+08	1.61E+08	1.61E+08	1.70E+08	1.40E+08	1.20E+08	3.13E+08	5.14E+08	9.55E+08	27.88	27.66	27.63	27.43	27.21	27.18	28.12	28.45	29.17	27.66	27.21	28.45	0.137709	0.135339	0.537752
1478	P11142	Heat shock	HSPA8	33	52.3	3.68E+09	3.22E+09	3.32E+09	4.74E+09	4.77E+09	3.98E+09	4.58E+09	4.46E+09	4.65E+09	32.5	32.61	32.55	32.09	32.04	32.2	31.78	31.5	30.67	32.55	32.09	31.5	0.055261	0.078903	0.579768
1813	P48637	Glutathion	GSS	5	8.9		3.17E+06	5.83E+06			3.12E+06					21.86	22.8			21.87				22.33	21.87		0.665105		
1223	O60502-4	Protein O-(	MGEA5	4	5.6		3.74E+06	2.43E+06	2.32E+06	2.31E+06	2.56E+06		2.73E+06			22.12	21.52	21.16	21.36	21.61		21.24		21.82	21.36	21.24	0.426771	0.223217	
2252	Q15020-4	Squamous	SART3	5	6.8	3.59E+06	5.04E+06	5.09E+06	3.04E+06	3.76E+06	3.51E+06				21.74	22.49	22.58	21.56	22.03	22.04				22.49	22.03		0.461939	0.274933	
956	H7C1E4	AP-1 comp	AP1S1	3	16.8	1.95E+06	2.69E+06	2.03E+06	1.77E+06	1.64E+06		2.54E+06			20.81	21.59	21.29	20.74	20.91		21.19			21.29	20.83	21.19	0.391947	0.116574	
909	H0YL43	Reticulocal	RCN2	3	22.6	8.76E+06	9.13E+06	7.61E+06	3.91E+06	5.88E+06	7.33E+06				23.02	23.3	23.14	21.9	22.68	23.07				23.14	22.68		0.138086	0.593351	
339	C9JL30	Sulfatase-m	SUMF2	5	23.5			8.47E+06			6.22E+06						23.29			22.83				23.29	22.83				
1528	P15170-2	Eukaryotic	GSPT1	17	34.1	5.08E+07	9.77E+07	9.87E+07	5.22E+07	7.31E+07	8.37E+07	7.89E+07	6.09E+07	3.53E+07	25.56	26.77	26.93	25.76	26.31	26.58	26.18	25.66	24.65	26.77	26.31	25.66	0.74618	0.41734	0.776173
145	A0A096LN	Dedicator c	DOCK1	19	12.1	1.72E+07	1.42E+07	1.44E+07	1.21E+07	1.00E+07	1.29E+07	7.63E+06	1.12E+07		24.01	23.93	24	23.54	23.42	23.84	22.79	23.19		24	23.54	22.99	0.041809	0.214002	0.285053
233	K7EM73	Calpain sm	CAPNS1	5	49.7	1.33E+07	1.69E+07	2.86E+07	6.97E+06	1.26E+07	2.33E+07	1.12E+07	9.25E+06		23.61	24.2	24.97	22.77	23.74	24.69	23.32	22.9		24.2	23.74	23.11	0.679997	0.959074	0.297392
1165	O15254-2	Peroxisom	ACOX3	5	10.6	4.65E+06	5.74E+06	6.48E+06	4.73E+06	4.20E+06	5.63E+06		3.90E+06		22.08	22.64	22.91	22.18	22.17	22.69		21.71		22.64	22.18	21.71	0.425044	0.299134	
2875	Q9BXP5-5	Serrate RN	SRRT	9	13	1.35E+07	3.16E+07	3.36E+07	2.20E+07	2.28E+07	2.79E+07	1.35E+07	1.38E+07	5.31E+06	23.63	25.05	25.18	24.46	24.58	24.98	23.61	23.5	21.78	25.05	24.58	23.5	0.858676	0.270766	1.025111
1877	P51659	Peroxisom	HSD17B4	15	28.7	3.74E+07	5.18E+07	4.77E+07	3.42E+07	3.68E+07	3.84E+07	5.35E+07	4.81E+07	1.13E+07	25.12	25.83	25.78	25.16	25.32	25.45	25.63	25.34	23.01	25.78	25.32	25.34	0.395798	0.145718	1.438031
281	A0A0U1RR	Histone H2	HIST1H2AJ	4	27.2	3.41E+08	1.35E+08	2.32E+08	1.14E+08	2.24E+08	1.89E+08	8.26E+08	8.59E+08	5.67E+08	28.4	27.36	28.38	26.87	27.91	27.96	29.5	29.42	28.48	28.38	27.91	29.42	0.590959	0.616378	0.564181
2113	Q08257	Quinone ox	CRYZ	5	15.2		6.23E+06	6.34E+06			4.45E+06					22.75	22.89			22.36				22.82	22.36		0.096168		
1543	P17655	Calpain-2 c	CAPN2	8	14.7	9.99E+06	9.83E+06	9.11E+06	6.66E+06	6.84E+06	9.55E+06	7.69E+06	6.84E+06		23.23	23.41	23.39	22.69	22.92	23.43	22.81	22.46		23.39	22.92	22.64	0.097469	0.377308	0.244222
2719	Q96CT7	Coiled-coil	CCDC124	4	21.1			4.86E+06			3.56E+06						22.52			22.05				22.52	22.05				
991	J3KN16	Proteasom	KIAA0368	23	15.4	1.46E+07	2.05E+07	2.44E+07	1.20E+07	1.47E+07	1.84E+07	1.27E+07	1.30E+07	3.83E+06	23.75	24.43	24.78	23.52	23.96	24.4	23.52	23.42	21.21	24.43	23.96	23.42	0.522779	0.438984	1.30554
2553	Q8N1G4	Leucine-ric	LRRC47	10	22.8	1.53E+07	1.67E+07	1.71E+07	1.33E+07	1.21E+07	1.51E+07	1.34E+07	1.18E+07	1.42E+07	23.81	24.16	24.26	23.69	23.68	24.07	23.59	23.28	23.37	24.16	23.69	23.37	0.233946	0.224381	0.160721
1529	P15374	Ubiquitin c	UCHL3	3	16.5	3.88E+06	6.92E+06	5.17E+06	3.49E+06		5.09E+06				21.86	22.92	22.6	21.73		22.54				22.6	22.13		0.546119	0.575624	
2545	Q8IZL8	Proline-, gl	PELP1	5	6.8	2.93E+06	3.44E+06	3.49E+06		2.43E+06	2.52E+06		1.98E+06		21.46	21.98	22.02		21.44	21.58		20.76		21.98	21.51	20.76	0.309513	0.093545	
964	H7C3F5	CCR4-NOT	CNOT3	3	8.9		2.65E+06	2.27E+06		1.61E+06	1.93E+06	2.06E+06				21.58	21.43		20.86	21.21	20.82			21.51	21.04	20.82	0.103281	0.247682	
814	F8W6I7	Heterogen	HNRNPA1	12	43.6	6.77E+07	7.11E+07	9.06E+07	4.31E+07	5.47E+07	7.69E+07	3.14E+07	3.87E+07	1.68E+07	25.95	26.34	26.79	25.47	25.87	26.47	24.89	25.02	23.64	26.34	25.87	24.89	0.419757	0.499187	0.759384
1591	P23193	Transcripti	TCEA1	5	17.6	5.30E+06	8.87E+06	5.24E+06		3.51E+06	4.54E+06			4.00E+06	22.25	23.24	22.64		21.94	22.39			21.27	22.64	22.16	21.27	0.499512	0.31784	
2582	Q8NBX0	Saccharopi	SCCPDH	6	19.8	1.96E+07	1.83E+07	1.59E+07	1.36E+07	9.20E+06	1.25E+07	1.98E+07	1.43E+07	1.36E+07	24.19	24.29	24.14	23.72	23.28	23.79	24.23	23.57	23.29	24.19	23.72	23.57	0.073824	0.276739	0.477987
1622	P26358	DNA (cytos	DNMT1	14	10	1.18E+07	1.23E+07	1.28E+07	9.81E+06	9.18E+06	9.58E+06	7.01E+06	5.50E+06		23.47	23.75	23.87	23.24	23.27	23.43	22.69	22.16		23.75	23.27	22.43	0.201222	0.104333	0.377125
3090	Q9P2J5	Leucine--tR	LARS	52	45.9	6.35E+08	4.32E+08	4.53E+08	3.75E+08	4.22E+08	4.86E+08	3.06E+08	3.71E+08	2.47E+08	29.34	29.13	29.3	28.41	28.83	29.23	28.09	28.03	27.12	29.3	28.83	28.03	0.112647	0.407753	0.54626
3097	Q9UBB9	Tuftelin-int	TFIP11	21	29.7	3.89E+07	4.13E+07	1.76E+07	3.03E+07	2.00E+07	2.38E+07	5.13E+07	2.87E+07	3.85E+07	25.19	25.52	24.3	24.87	24.43	24.71	25.59	24.6	24.73	25.19	24.71	24.73	0.629805	0.223582	0.537996
283	A0A0U1RR	Polypyrimi	PTBP1	11	33	8.29E+07	3.63E+07	5.62E+07	4.41E+07	4.51E+07	4.10E+07	2.31E+07	1.71E+07		26.32	25.31	26.03	25.52	25.59	25.55	24.46	23.81		26.03	25.55	24.14	0.523452	0.034437	0.459735
1236	O60763	General ve	USO1	15	16.2	2.22E+07	2.37E+07	2.75E+07	1.64E+07	1.66E+07	1.68E+07	1.70E+07	2.04E+07		24.34	24.64	24.91	24.03	24.16	24.25	24	24.12		24.64	24.16	24.06	0.284208	0.108339	0.08414
1824	P49327	Fatty acid s	FASN	93	47.5	6.97E+08	7.50E+08	7.65E+08	7.44E+08	6.96E+08	1.01E+09	4.94E+08	4.98E+08	2.24E+08	29.44	29.97	30.01	29.47	29.49	30.19	28.88	28.42	26.97	29.97	29.49	28.42	0.318998	0.412615	0.99428
1801	P47712	Cytosolic p	PLA2G4A	8	11.5	5.25E+06	7.06E+06	7.01E+06	4.63E+06	5.02E+06	5.19E+06	6.49E+06	3.92E+06	4.52E+06	22.23	22.95	23.01	22.15	22.47	22.57	22.6	21.72	21.48	22.95	22.47	21.72	0.428923	0.219658	0.590205
1891	P52732	Kinesin-like	KIF11	74	59.6	6.55E+09	6.60E+09	5.87E+09	7.63E+09	8.31E+09	6.86E+09	3.76E+09	8.30E+09	7.27E+09	33.21	33.58	33.63	33.01	33.28	33.1	31.57	32.48	31.74	33.58	33.1	31.74	0.231416	0.133768	0.486433
1295	O76071	Probable c	CIAO1	2	10.3	2.89E+06	2.95E+06	2.94E+06	1.94E+06	2.17E+06	2.65E+06	2.65E+06			21.43	21.74	21.79	20.91	21.26	21.65	21.25			21.74	21.26	21.25	0.196336	0.369094	
2923	Q9H269-2	Vacuolar p	VPS16	3	5.6	2.91E+06	1.88E+06	1.77E+06	1.59E+06	1.51E+06		2.96E+06			21.45	21.14	21.11	20.56	20.76		21.46			21.14	20.66	21.46	0.185112	0.144278	
1265	O75436	Vacuolar p	VPS26A	8	27.8	8.50E+06	1.16E+07	1.23E+07	8.70E+06	8.45E+06	8.50E+06	9.21E+06	9.25E+06	8.17E+06	22.98	23.66	23.79	23.04	23.17	23.26	23.09	22.9	22.47	23.66	23.17	22.9	0.436846	0.110701	0.313965
2836	Q9BS26	Endoplasm	ERP44	9	27.6	4.25E+07	3.04E+07	2.37E+07	2.28E+07	2.04E+07	2.55E+07	2.52E+07	2.79E+07	1.68E+07	25.35	25	24.75	24.52	24.46	24.78	24.59	24.55	23.64	25	24.52	24.55	0.304085	0.170811	0.53324
1976	P62258	14-3-3 prot	YWHAE	13	58.8	1.07E+08	1.45E+08	2.16E+08	1.22E+08	9.14E+07	1.44E+08	1.37E+08	1.25E+08	5.95E+07	26.77	27.47	28.21	26.99	26.67	27.54	27.07	26.69	25.29	27.47	26.99	26.69	0.719488	0.438464	0.933643
1332	O95630	STAM-bind	STAMBP	5	12		3.76E+06	2.61E+06		2.36E+06			2.72E+06			22.13	21.62		21.39			21.23		21.88	21.39	21.23	0.363165		
3008	Q9NR30-2	Nucleolar R	DDX21	7	11.9	7.90E+06	8.25E+06	4.58E+06	4.30E+06	4.68E+06	5.00E+06	4.90E+06	3.78E+06		22.84	23.15	22.44	22.05	22.36	22.51	22.17	21.66		22.84	22.36	21.92	0.358625	0.23714	0.358017
636	E7EQ29	Beta-galact	GLB1	4	9.2	2.82E+06	4.27E+06	4.16E+06		2.71E+06	3.38E+06	2.97E+06	3.07E+06		21.39	22.29	22.31		21.61	21.99	21.47	21.39		22.29	21.8	21.43	0.525788	0.26676	0.056497
2943	Q9H488-2	GDP-fucos	POFUT1	2	13.4		2.68E+06	2.18E+06		1.44E+06	2.02E+06					21.59	21.38		20.72	21.28				21.48	21		0.14346	0.392848	

367	M0QZS6	SUMO-acti	SAE1	6	29.8	9.56E+06	8.51E+06	1.31E+07	6.71E+06	5.47E+06	8.78E+06	7.34E+06			23.16	23.19	23.9	22.71	22.59	23.3	22.75			23.19	22.71	22.75	0.417001	0.37771	
180	A0A0A0MS	ATP-deper	DDX42	8	16.1	6.41E+06	5.00E+06	5.99E+06	3.69E+06	3.82E+06	4.26E+06		2.56E+06		22.55	22.48	22.82	21.82	22.06	22.3		21.1		22.55	22.06	21.1	0.179565	0.239606	
1938	P60510	Serine/thre	PPP4C	4	21.5	6.54E+06	8.49E+06	7.44E+06	6.35E+06	5.16E+06	6.39E+06	7.15E+06	6.81E+06	4.44E+06	22.57	23.19	23.12	22.63	22.51	22.86	22.72	22.45	21.44	23.12	22.63	22.45	0.339954	0.179333	0.67218
1694	P32322-2	Pyrroline-5	PYCR1	3	11.4	3.00E+06	5.03E+06	4.30E+06			3.10E+06	3.04E+06			21.51	22.49	22.35			21.86	21.49			22.35	21.86	21.49	0.530213		
2988	Q9HCS7	Pre-mRNA	XAB2	4	7.7		2.89E+06	2.41E+06	1.82E+06	1.96E+06	1.89E+06					21.71	21.51	20.78	21.12	21.18				21.61	21.12		0.142125	0.214683	
835	G3V3D2	Spermatog	SPATA7	2	21.4			2.09E+07			1.50E+07						24.55			24.06				24.55	24.06				
1816	P48681	Nestin	NES	7	5.9		3.18E+06	3.97E+06	3.05E+06	2.61E+06	2.85E+06					21.87	22.25	21.57	21.54	21.76				22.06	21.57		0.269281	0.122544	
744	F5GWE5	Phosphatic	PITPNA	4	23	2.29E+06	4.29E+06	3.03E+06		1.78E+06	2.68E+06		1.88E+06		21.07	22.3	21.84		21.03	21.66		20.68		21.84	21.35	20.68	0.618277	0.447812	
2757	Q96L58	Beta-1,3-ga	B3GALT6	3	10	2.41E+06	1.54E+06	1.90E+06	1.57E+06	1.50E+06	1.29E+06	1.40E+06			21.15	20.87	21.21	20.53	20.76	20.66	20.32			21.15	20.66	20.32	0.18469	0.114833	
1758	P40939	Trifunction	HADHA	13	28.4	1.89E+07	1.83E+07	2.24E+07	1.84E+07	1.13E+07	1.25E+07	1.42E+07	1.07E+07	3.03E+07	24.15	24.29	24.66	24.19	23.57	23.79	23.72	23.12	24.37	24.29	23.79	23.72	0.263077	0.311571	0.62609
2549	Q8N163-2	Cell cycle a	CCAR2	16	24.2	1.23E+07	1.83E+07	1.92E+07	8.45E+06	1.30E+07	1.56E+07	8.51E+06	1.10E+07	5.09E+06	23.52	24.29	24.41	23	23.79	24.12	22.96	23.15	21.72	24.29	23.79	22.96	0.479875	0.575296	0.774921
803	F8VYE8	Serine/thre	PPP1CC	2	32.6		9.43E+06	8.01E+06	4.90E+06		9.11E+06	3.66E+06				23.35	23.22	22.22		23.37	21.75			23.29	22.79	21.75	0.088052	0.808806	
7	A0A024RC	Large proli	BAT3	8	8.3	5.62E+06	1.00E+07	8.76E+06	5.96E+06	6.55E+06	7.19E+06	4.99E+06	5.66E+06		22.33	23.44	23.33	22.53	22.84	23.03	22.2	22.21		23.33	22.84	22.2	0.608835	0.255489	0.002449
1737	P38117	Electron tr	ETFB	10	46.3	1.17E+07	1.91E+07	1.93E+07	1.48E+07	8.85E+06	1.71E+07	1.13E+07	9.90E+06	1.28E+07	23.46	24.35	24.42	23.85	23.22	24.27	23.34	23.02	23.19	24.35	23.85	23.19	0.532031	0.526953	0.162177
1781	P43304-2	Glycerol-3-	GPD2	11	22.5	1.45E+07	1.39E+07	1.59E+07	1.12E+07	9.94E+06	1.53E+07	1.43E+07	1.54E+07	1.13E+07	23.74	23.9	24.14	23.41	23.41	24.1	23.73	23.67	23.01	23.9	23.41	23.67	0.200406	0.399957	0.4017
982	I3L2B0	Clustered r	CLUH	10	9.1	6.41E+06	7.33E+06	7.84E+06	5.90E+06	4.69E+06	5.29E+06	4.31E+06	3.57E+06		22.55	23	23.19	22.5	22.36	22.6	22.01	21.58		23	22.5	21.79	0.329235	0.121257	0.303294
2102	Q06830	Peroxiredo	PRDX1	20	76.4	4.87E+09	3.83E+09	2.49E+09	5.97E+09	5.00E+09	3.76E+09	5.31E+09	6.14E+09	6.77E+09	32.83	32.86	32.33	32.46	32.34	32.13	31.97	31.99	31.56	32.83	32.34	31.97	0.302385	0.16373	0.239965
2979	Q9HAV7	GrpE prote	GRPEL1	3	18	3.52E+06	4.57E+06	5.58E+06	2.78E+06	3.35E+06	3.96E+06				21.7	22.38	22.75	21.43	21.88	22.2				22.38	21.88		0.531606	0.385863	
437	C9J315	Lanosterol	LSS	3	15.6	1.01E+06	1.40E+06	1.65E+06		9.09E+05	9.47E+05				19.83	20.7	20.97		20.05	20.35				20.7	20.2		0.597724	0.207462	
2841	Q9BSJ8	Extended s	ESYT1	9	11.2	6.78E+06	6.57E+06	7.23E+06	5.08E+06	4.55E+06	4.70E+06	4.24E+06	4.69E+06		22.62	22.83	23.07	22.29	22.33	22.43	21.99	21.95		22.83	22.33	21.97	0.225069	0.06925	0.029237
1912	P54136	Arginine--t	RARS	44	61.5	1.17E+09	6.50E+08	8.15E+08	8.15E+08	7.71E+08	9.50E+08	4.81E+08	6.57E+08	4.79E+08	30.5	29.59	30.21	29.72	29.67	30.11	28.79	28.85	28.25	30.21	29.72	28.79	0.465732	0.24138	0.332987
3018	Q9NSD9	Phenylalan	FARSB	14	30.2	2.20E+07	2.16E+07	3.85E+07	1.64E+07	1.46E+07	2.88E+07	1.71E+07	1.57E+07	2.98E+06	24.33	24.53	25.45	24.03	23.94	25.01	24.01	23.71	20.93	24.53	24.03	23.71	0.597661	0.594311	1.694496
875	H0Y621	Endoplasm	ERGIC3	2	7.7		3.07E+06	3.41E+06		2.06E+06	2.54E+06		2.34E+06			21.8	21.99		21.2	21.59		21		21.89	21.39	21	0.135421	0.276765	
325	A6NNK5	Tumor sup	TP53BP1	8	6.5	5.22E+06	3.94E+06	3.76E+06	3.31E+06	2.88E+06	2.90E+06				22.23	22.2	22.18	21.68	21.7	21.78				22.2	21.7		0.023237	0.05352	
893	H0YD13	CD44 antig	CD44	4	23.3	3.10E+06	2.29E+06	2.56E+06	1.87E+06	2.67E+06	1.77E+06				21.54	21.39	21.59	20.85	21.58	21.03				21.54	21.03		0.103879	0.380425	
57	A0A087WV	cAMP-depe	PRKACB	5	15.1		9.37E+06	9.51E+06		6.67E+06	6.72E+06		6.67E+06			23.34	23.46		22.87	22.93		22.41		23.4	22.9	22.41	0.08562	0.045243	
132	A0A087X1	Proteasom	PSME2	8	33.1	1.53E+07	2.15E+07	2.91E+07	1.62E+07	1.08E+07	1.76E+07			1.03E+07	23.81	24.51	25.01	24.01	23.52	24.31			22.85	24.51	24.01	22.85	0.601225	0.399325	
1903	P53597	Succinyl-Co	SUCLG1	5	20.5		4.35E+06	3.69E+06		2.29E+06	3.64E+06	4.22E+06	3.85E+06			22.3	22.15		21.35	22.1	21.98	21.7		22.23	21.72	21.84	0.109629	0.528135	0.202994
163	A0A0A0MI	Melanoma	MIA3	6	7.5	3.00E+06	2.89E+06	2.81E+06	1.95E+06	2.43E+06	1.92E+06		1.62E+06		21.51	21.71	21.73	20.93	21.44	21.2		20.4		21.71	21.2	20.4	0.118777	0.256581	
687	E9PF10	Nuclear po	NUP155	24	23.8	3.79E+07	4.98E+07	4.90E+07	3.63E+07	3.43E+07	3.97E+07	2.13E+07	2.57E+07	5.38E+06	25.14	25.74	25.85	25.23	25.19	25.5	24.36	24.43	21.8	25.74	25.23	24.36	0.378488	0.168743	1.500538
2819	Q9BPX5	Actin-relat	ARPC5L	4	46.4	5.14E+06	9.12E+06	7.21E+06	6.05E+06	4.91E+06	6.80E+06	7.66E+06	8.81E+06	6.55E+06	22.21	23.3	23.07	22.56	22.43	22.94	22.79	22.85	22.14	23.07	22.56	22.79	0.574751	0.267852	0.391554
2870	Q9BWF3	RNA-bindir	RBM4	8	32.7	9.98E+06	9.71E+06	9.31E+06	8.23E+06	6.71E+06	5.88E+06	8.09E+06	7.51E+06		23.23	23.39	23.42	22.97	22.88	22.76	22.89	22.62		23.39	22.88	22.76	0.10478	0.104585	0.190954
2140	Q12904	Aminoacyl	AIMP1	18	77.9	5.08E+08	3.04E+08	3.34E+08	3.59E+08	3.18E+08	2.96E+08	1.98E+08	3.23E+08	2.12E+08	28.9	28.64	28.92	28.39	28.35	28.59	27.59	27.87	26.92	28.9	28.39	27.59	0.156425	0.130365	0.490565
3218	Q9Y3D6	Mitochond	FIS1	2	15.8			1.06E+07			7.42E+06						23.59			23.08				23.59	23.08				
605	D6RFN0	COP9 signa	COPS4	10	29.2	1.96E+07	2.66E+07	2.16E+07	1.61E+07	1.60E+07	2.23E+07	2.03E+07	1.93E+07	1.17E+07	24.19	24.83	24.6	24	24.09	24.62	24.29	24.02	23.07	24.6	24.09	24.02	0.320628	0.335672	0.644514
2907	Q9H078-5	Caseinolyti	CLPB	4	11.3	2.59E+06	3.71E+06	3.97E+06	2.94E+06	2.68E+06	2.55E+06	2.70E+06			21.26	22.1	22.25	21.52	21.59	21.6	21.29			22.1	21.59	21.29	0.529487	0.043446	
1149	O14980	Exportin-1	XPO1	25	28	7.37E+07	9.83E+07	9.11E+07	6.19E+07	7.17E+07	9.02E+07	3.32E+07	5.60E+07	1.26E+07	26.15	26.78	26.8	25.96	26.26	26.69	24.97	25.56	23.17	26.78	26.26	24.97	0.368316	0.36674	1.245354
3129	Q9UHI6	Probable A	DDX20	19	31.1	6.38E+07	7.06E+07	3.40E+07	4.03E+07	4.03E+07	3.57E+07	7.69E+07	3.32E+07	2.42E+07	25.87	26.33	25.21	25.36	25.41	25.35	26.11	24.81	24.08	25.87	25.36	24.81	0.56195	0.031896	1.029584
2679	Q92900-2	Regulator c	UPF1	19	23.4	2.63E+07	3.41E+07	3.32E+07	1.76E+07	2.42E+07	2.71E+07	1.73E+07	1.35E+07	8.30E+06	24.6	25.21	25.16	24.1	24.65	24.92	24.03	23.46	22.51	25.16	24.65	23.46	0.339237	0.41393	0.76833
1863	P50897	Palmitoyl-p	PPT1	2	10.8	2.88E+06	4.01E+06	3.09E+06	2.59E+06	2.12E+06	2.59E+06	2.75E+06			21.42	22.22	21.87	21.36	21.23	21.63	21.32			21.87	21.36	21.32	0.402113	0.204692	
2796	Q99460-2	26S protea	PSMD1	27	40.5	1.67E+08	1.69E+08	1.61E+08	1.29E+08	1.30E+08	1.30E+08	9.25E+07	1.27E+08	1.07E+08	27.43	27.73	27.63	27.11	27.11	27.37	26.48	26.71	26.11	27.63	27.11	26.48	0.155001	0.146024	0.298566
1524	P14735	Insulin-deg	IDE	15	16.6	1.67E+07	1.68E+07	1.65E+07	1.18E+07	1.20E+07	1.21E+07	1.99E+07	1.79E+07	9.05E+06	23.96	24.18	24.2	23.49	23.66	23.71	24.23	23.88	22.62	24.18	23.66	23.88	0.136239	0.115174	0.845637
2680	Q92905	COP9 signa	COPS5	7	22.8	7.72E+06	9.36E+06	8.38E+06	6.96E+06	5.77E+06	7.20E+06	5.80E+06	6.71E+06	5.02E+06	22.81	23.34	23.28	22.76	22.66	23.04	22.44	22.43	21.68	23.28	22.76	22.43	0.29049	0.194948	0.431992
1153	O15042-2	U2 snRNP-	U2SURP	11	12.9	7.57E+06	1.11E+07	1.38E+07	7.56E+06	7.85E+06	1.10E+07	4.33E+06	5.26E+06		22.78	23.59	23.94	22.88	23.08	23.59	22.02	22.07		23.59	23.08	22.05	0.598698	0.366448	0.039309
2608	Q8TC12-2	Retinol del	RDH11	4	19.3		8.19E+06	7.20E+06	6.15E+06	4.54E+06	6.73E+06	5.80E+06				23.14	23.06	22.59	22.32	22.94	22.44			23.1	22.59	22.44	0.058308	0.3077	
1705	P35241	Radixin	RDX	8	32.2	9.59E+06	1.08E+07	1.25E+07	5.46E+06	7.56E+06	9.01E+06	8.02E+06			23.16	23.55	23.82	22.39	23.03	23.34	22.87			23.55	23.03	22.87	0.330247	0.487057	
1375	P02792	Ferritin ligl	FTL	3	17.7		3.24E+06	3.59E+06		2.12E+06	2.77E+06					21.89	22.09		21.23	21.72				21.99	21.47		0.144575	0.34812	
1670	P30622-2	CAP-Gly dc	CLIP1	25	21.3	1.72E+07	2.25E+07	1.87E+07	1.60E+07	1.29E+07	1.31E+07	1.28E+07	6.79E+06		24.01	24.59	24.38	23.99	23.78	23.86	23.54	22.44		24.38	23.86	22.99	0.293883	0.103973	0.773152
2200	Q14008-2	Cytoskelet	CKAP5	29	16	2.73E+07	3.33E+07	3.66E+07	2.09E+07	2.43E+07	2.36E+07	2.38E+07	2.21E+07	1.19E+07	24.64	25.17	25.36	24.38	24.65	24.7	24.49	24.21	23.09	25.17	24.65	24.21	0.37066	0.174204	0.742687
3065	Q9NZB2-4	Constitutiv	FAM120A	16	18.9	2.51E+09	1.80E+09	1.77E+09	1.52E+09	2.10E+09	1.75E+09	1.53E+09	1.52E+09	7.60E+08	31.92	31.74	31.77	30.7	31.25	31.46	30.76	30.53	28.85	31.77	31.25	30.53	0.094984	0.39155	1.045413
1172	O15357	Phosphatic	INPPL1	12	11.6	7.83E+06	9.84E+06	8.96E+06	6.07E+06	6.55E+06	7.15E+06	7.20E+06	6.25E+06	1.78E+06	22.82	23.41	23.36	22.56	22.84	23.03	22.72	22.33	19.99	23.36	22.84	22.33	0.32763	0.232098	1.477719
2961	Q9H832-2	Ubiquitin-c	UBE2Z	3	13.4		2.42E+06	2.45E+06	1.85E+06		1.83E+06					21.47	21.53	20.84		21.11				21.5	20.98		0.041877	0.195261	
2666	Q92620	Pre-mRNA	DHX38	22	24.4	3.85E+07	4.20E+07	3.74E+07	2.43E+07	2.76E+07	3.01E+07	3.04E+07	2.22E+07	5.12E+06	25.17	25.55	25.42	24.59	24.9	25.09	24.84	24.22	21.74	25.42	24.9	24.22	0.192472	0.25122	1.643337
1562	P19623	Spermidine	SRM	8	31.1	1.81E+07	2.13E+07	2.04E+07	1.59E+07	1.38E+07	1.63E+07	1.76E+07	1.74E+07	1.64E+07	24.08	24.49	24.51	23.97	23.87	24.18	24.05	23.85	23.6	24.49	23.97	23.85	0.241088	0.160023	0.22239
241	A0A0D9SE	Cyclin-dep	CDK11B	13	17	8.66E+06	1.29E+07	9.52E+06	6.68E+06	6.96E+06	1.02E+07	5.40E+06	2.90E+06		23	23.82	23.46	22.7	22.94	23.5	22.34	21.31		23.46	22.94	21.82	0.406987	0.411288	0.726626
1936	P60228	Eukaryotic	EIF3E	21	51	3.17E+08	3.74E+08	3.00E+08	3.19E+08	2.76E+08	2.83E+08	4.16E+08	4.45E+08	1.61E+08	28.3	28.92	28.79	28.26	28.21	28.46	28.6	28.27	26.53	28.79	28.26	28.27	0.324718	0.13123	1.110947
586	H0Y9L5	Exosome c	EXOSC9	2	12.8	4.38E+06	3.97E+06	3.82E+06		2.52E+06	3.13E+06	2.68E+06	2.80E+06		22.01	22.22	22.2		21.48	21.88	21.28	21.27		22.2	21.68	21.28	0.117572	0.284357	0.005108
255	H9KV28	Protein dia	DIAPH1	15	16	1.28E+07	3.06E+07	3.61E+07	1.26E+07	2.09E+07	3.61E+07	1.07E+07	1.72E+07		23.58	25.01	25.33	23.59	24.49	25.11	23.27	23.82		25.01	24.49	23.55	0.932234	0.762193	0.388743
2760	Q96MX6	WD repeat	WDR92	4	12.3		3.84E+06	2.81E+06			2.22E+06	2.59E+06				22.16	21.73			21.42	21.22			21.94	21.42	21.22	0.310649		
1671	P30740	Leukocyte	SERPINB1	10	24.8	1.47E+07	1.63E+07	1.59E+07	1.08E+07	1.14E+07	1.40E+07	1.41E+07	1.75E+07	1.11E+07	23.76	24.11	24.14	23.36	23.59	23.95	23.7	23.86	22.98	24.11	23.59	23.7	0.213807	0.299868	0.467479
355	B1AKJ5	Nardilysin	NRD1	16	15.5	8.87E+06	1.22E+07	1.43E+07	6.07E+06	8.78E+06	1.28E+07	7.29E+06	4.39E+06		23.04	23.74	23.99	22.56	23.21	23.82	22.74	21.85		23.74	23.21	22.29	0.489676	0.629162	0.628555
3187	Q9Y276	Mitochond	BCS1L	3	8.8		2.01E+06	1.63E+06			1.20E+06					21.24	20.94			20.56				21.09	20.56		0.211727		
2129	Q10713	Mitochond	PMPCA	5	8.4		1.58E+06	3.78E+06	2.10E+06	1.70E+06	2.79E+06					20.89	22.19	21.01	20.97	21.73				21.54	21.01		0.919008	0.429178	
88	A0A087WV	AP-2 comp	AP2M1	7	18	8.50E+06	1.35E+07	1.47E+07	1.03E+07	9.49E+06	1.30E+07	8.06E+06	7.76E+06		22.98	23.86	24.02	23.32	23.33	23.85	22.88	22.67		23.86	23.33	22.78	0.562193	0.3031	0.153921
2813	Q99961	Endophilin	SH3GL1	5	17.9	8.24E+06	9.52E+06	9.50E+06	5.37E+06	6.62E+06	6.49E+06	5.52E+06	8.01E+06		22.94	23.38	23.46	22.36	22.85	22.88	22.37	22.71		23.38	22.85	22.54	0.280074	0.290977	0.244264
2747	Q96IR7	4-hydroxyp	HPDL	4	11.3		3.29E+06	3.03E+06	2.48E+06		2.22E+06	2.74E+06				21.91	21.84	21.28		21.42	21.31			21.88	21.35	21.31	0.054544	0.099779	
315	A6NHR9	Structural r	SMCHD1	24	15.1	1.92E+07	2.56E+07	2.22E+07	1.45E+07	1.63E+07	2.24E+07	1.23E+07	1.25E+07	2.84E+06	24.17	24.79	24.65	23.82	24.12	24.63	23.46	23.37	20.87	24.65	24.12	23.37	0.322783	0.410078	1.469457
750	F5GX77	Multifunct	TRMT112	2	25.5	3.51E+06	5.25E+06	4.70E+06		3.52E+06	3.26E+06	2.81E+06	2.99E+06		21.69	22.53	22.47		21.94	21.94	21.36	21.37		22.47	21.94	21.36	0.466823	0.00272	0.008433
1097	O00170	AH recepto	AIP	4	14.5		6.73E+06	5.82E+06			4.23E+06					22.86	22.8			22.29				22.83	22.29		0.045121		
2668	Q92626	Peroxidasi	PXDN	9	7.6	3.85E+06	5.23E+06	5.17E+06	3.19E+06	3.63E+06	4.42E+06	3.33E+06	3.55E+06		21.85	22.52	22.6	21.63	21.99	22.35	21.61	21.57		22.52	21.99	21.59	0.413823	0.356675	0.031441
136	A0A087X2	WASH com	KIAA1033	7	7.1	3.18E+06	3.69E+06	3.45E+06	2.48E+06	2.48E+06	2.52E+06		3.49E+06		21.56	22.08	22	21.28	21.47	21.58		21.55		22	21.47	21.55	0.279311	0.151277	
2160	Q13200	26S protea	PSMD2	31	41.2	1.51E+08	1.61E+08	9.83E+07	7.39E+07	9.28E+07	1.02E+08	1.13E+08	8.08E+07	6.53E+07	27.23	27.66	26.91	26.24	26.7	26.9	26.77	26.08	25.42	27.23	26.7	26.08	0.378227	0.336233	0.675084
407	B7Z524	HBS1-like p	HBS1L	4	11.5	6.89E+06	7.19E+06	7.66E+06	4.61E+06	6.10E+06	4.76E+06	4.24E+06	4.05E+06		22.64	22.97	23.15	22.14	22.74	22.44	21.99	21.76		22.97	22.44	21.88	0.259031	0.300736	0.16223
2063	Q01082	Spectrin be	SPTBN1	135	61.8	1.52E+09	1.48E+09	1.38E+09	1.59E+09	1.33E+09	1.38E+09	9.07E+08	9.92E+08	4.26E+08	31	31.52	31.29	30.88	30.66	30.76	29.68	29.79	27.93	31.29	30.76	29.68	0.260806	0.109637	1.041248
1500	P12270	Nucleoprot	TPR	66	32.9	2.65E+08	2.62E+08	2.36E+08	2.32E+08	1.91E+08	1.85E+08	1.39E+08	1.17E+08	1.12E+08	28.03	28.39	28.44	27.85	27.64	27.9	27.1	26.59	26.15	28.39	27.85	26.59	0.225021	0.139558	0.472518
2020	P67775	Serine/thre	PPP2CA	11	45	5.09E+07	5.18E+07	5.05E+07	3.38E+07	3.65E+07	5.21E+07	3.77E+07	4.77E+07	2.90E+07	25.58	25.83	25.9	25.12	25.29	25.87	25.16	25.32	24.31	25.83	25.29	25.16	0.168647	0.392697	0.540546
2858	Q9BV20-2	Methylthic	MRI1	6	22.4	5.62E+06	6.23E+06	7.28E+06	4.82E+06	4.80E+06	3.99E+06	5.37E+06	5.06E+06		22.33	22.75	23.08	22.2	22.4	22.21	22.33	22.04		22.75	22.21	22.18	0.373796	0.114238	0.202767

239	A0A0C4DG	Tripeptidyl	TPP1	2	5		2.36E+06	2.26E+06		1.46E+06	1.78E+06	2.03E+06				21.43	21.43		20.73	21.04	20.81			21.43	20.89	20.81	0.000307	0.221739	
1792	P46777	60S riboso	RPL5	8	32.3	8.45E+07	4.94E+07	4.43E+07	3.44E+07	1.99E+07	4.94E+07	3.46E+07	3.94E+07	1.42E+07	26.35	25.71	25.64	25.17	24.41	25.77	25.04	25.04	23.37	25.71	25.17	25.04	0.388481	0.678544	0.964397
1241	O60869-2	Endothelia	EDF1	2	16.5	3.75E+06	6.45E+06	4.79E+06	3.83E+06	3.56E+06	5.05E+06	5.20E+06	3.94E+06		21.81	22.81	22.51	21.87	21.96	22.53	22.27	21.72		22.51	21.96	22	0.514148	0.355369	0.387801
1817	P48735-2	Isocitrate c	IDH2	12	32.8	1.01E+07	1.56E+07	1.78E+07	9.12E+06	1.07E+07	1.63E+07	1.04E+07	7.34E+06		23.25	24.06	24.33	23.13	23.51	24.18	23.24	22.57		24.06	23.51	22.91	0.561189	0.531539	0.472527
2994	Q9NPD3	Exosome c	EXOSC4	5	25.7	1.45E+07	1.40E+07	1.29E+07	8.09E+06	9.47E+06	1.21E+07	8.78E+06	1.03E+07		23.74	23.91	23.88	22.96	23.33	23.71	23.01	23.07		23.88	23.33	23.04	0.090675	0.376877	0.0414
2014	P63104	14-3-3 prot	YWHAZ	16	59.2	4.22E+08	5.12E+08	5.23E+08	3.23E+08	3.98E+08	4.48E+08	4.41E+08	3.93E+08	1.23E+08	28.69	29.24	29.49	28.28	28.7	29.04	28.66	28.12	26.24	29.24	28.7	28.12	0.409968	0.38171	1.271476
797	F8VVA7	Coatomer :	COPZ1	5	31.3	6.98E+06	1.55E+07	1.98E+07	9.11E+06	1.06E+07	1.24E+07	1.51E+07	8.19E+06	5.96E+06	22.66	24.05	24.45	23.13	23.5	23.78	23.82	22.75	21.95	24.05	23.5	22.75	0.936338	0.32379	0.93729
110	D6R9P3	Heterogen	HNRNPAB	6	25.4	2.63E+07	2.07E+07	2.43E+07	8.71E+06	1.56E+07	1.66E+07	1.18E+07	1.10E+07		24.6	24.44	24.78	23.05	24.05	24.22	23.39	23.15		24.6	24.05	23.27	0.166483	0.63304	0.170405
1129	O14646-2	Chromodo	CHD1	13	9.4	9.42E+06	1.33E+07	1.11E+07	8.07E+06	8.28E+06	8.93E+06	8.47E+06	6.84E+06	4.55E+06	23.13	23.84	23.68	22.95	23.13	23.32	22.95	22.46	21.51	23.68	23.13	22.46	0.372612	0.186858	0.730075
2050	P98170	E3 ubiquiti	XIAP	3	9.1	1.43E+06	2.15E+06	3.33E+06		1.25E+06	1.80E+06				20.37	21.33	21.96		20.49	21.07				21.33	20.78		0.801625	0.411056	
269	A0A0J9YX8	PHD and R	PHRF1	6	5.9	1.77E+07	6.51E+06	1.12E+07	8.04E+06	8.31E+06	8.25E+06	1.55E+06	2.52E+06		24.05	22.82	23.69	22.94	23.14	23.23	20.51	21.08		23.69	23.14	20.79	0.631292	0.150941	0.409353
1841	P49755	Transmem	TMED10	6	29.2	2.40E+07	3.24E+07	4.42E+07	2.05E+07	2.22E+07	2.30E+07	1.87E+07	1.91E+07		24.48	25.1	25.63	24.34	24.55	24.67	24.13	23.99		25.1	24.55	24.06	0.57473	0.164834	0.098099
718	E9PLK3	Puromycin	NPEPPS	23	27.1	2.30E+07	4.24E+07	4.02E+07	2.49E+07	2.88E+07	3.38E+07	3.62E+07	2.48E+07	1.85E+07	24.4	25.56	25.51	24.63	24.96	25.27	25.13	24.39	23.74	25.51	24.96	24.39	0.655384	0.317805	0.694788
994	J3KN36	Nodal mod	NOMO3	9	11	5.22E+06	7.76E+06	6.80E+06	4.60E+06	4.83E+06	5.70E+06	7.69E+06	6.75E+06	5.80E+06	22.23	23.06	22.96	22.13	22.41	22.71	22.81	22.43	21.92	22.96	22.41	22.43	0.456347	0.286115	0.449033
176	E9PF18	Hydroxyac	HADH	3	11	6.13E+06	9.77E+06	9.87E+06	4.38E+06	6.59E+06	8.16E+06	5.86E+06			22.47	23.4	23.52	22.07	22.85	23.22	22.45			23.4	22.85	22.45	0.570963	0.586918	
369	B3KS98	Eukaryotic	EIF3H	14	41.5	6.99E+07	9.30E+07	7.65E+07	6.24E+07	5.85E+07	5.89E+07	8.98E+07	7.75E+07	2.62E+07	26.02	26.72	26.54	25.98	25.98	26.03	26.41	26.02	24.16	26.54	25.98	26.02	0.361188	0.02947	1.201114
2092	Q05048	Cleavage s	CSTF1	5	12.5		6.89E+06	5.02E+06		3.49E+06	4.73E+06		3.72E+06			22.9	22.57		21.93	22.43		21.63		22.74	22.18	21.63	0.239028	0.358419	
60	A0A087W\	Ribosome-	RRBP1	14	20.4	1.02E+07	1.03E+07	1.42E+07	7.98E+06	5.97E+06	8.31E+06	7.25E+06	3.48E+06		23.27	23.48	23.98	22.93	22.7	23.24	22.73	21.54		23.48	22.93	22.14	0.36664	0.269726	0.841208
1188	O43264-2	Centromer	ZW10	5	9.8	8.03E+06	1.10E+07	1.03E+07	8.46E+06	6.90E+06	8.70E+06	1.00E+07	1.44E+07		22.88	23.58	23.56	23	22.93	23.29	23.2	23.58		23.56	23	23.39	0.398038	0.192164	0.273103
2032	P78347-2	General tra	GTF2I	21	24.8	2.63E+07	3.24E+07	3.40E+07	1.98E+07	2.21E+07	3.09E+07	2.39E+07	1.51E+07	6.68E+06	24.6	25.1	25.21	24.29	24.54	25.15	24.5	23.64	22.16	25.1	24.54	23.64	0.326256	0.439009	1.18478
2792	Q96TA1-2	Niban-like	FAM129B	14	28.1	1.59E+07	2.78E+07	3.23E+07	1.64E+07	1.90E+07	2.35E+07	2.40E+07	2.04E+07		23.87	24.9	25.13	24.03	24.34	24.7	24.51	24.12		24.9	24.34	24.31	0.666817	0.333516	0.27737
862	X6RM00	ELKS/Rab6	ERC1	7	8.4	3.76E+06	3.69E+06	4.24E+06	2.83E+06	2.60E+06	2.66E+06		3.60E+06		21.81	22.08	22.33	21.46	21.52	21.66		21.59		22.08	21.52	21.59	0.262097	0.099884	
1304	O94915	Protein fur	FRYL	17	5.7	8.87E+07	1.21E+08	6.66E+07	6.24E+07	5.62E+07	5.04E+07	6.98E+07	9.53E+07	1.14E+07	26.48	27.21	26.32	25.98	25.92	25.8	25.97	26.3	23.03	26.48	25.92	25.97	0.47396	0.092634	1.801579
2954	Q9H6T3	RNA polym	RPAP3	7	11.9	4.69E+06	4.64E+06	4.42E+06	3.70E+06	3.03E+06	3.88E+06	3.34E+06	3.97E+06		22.08	22.39	22.39	21.83	21.75	22.17	21.62	21.73		22.39	21.83	21.68	0.176665	0.224177	0.077891
1651	P30040	Endoplasm	ERP29	7	32.6	2.43E+07	4.57E+07	4.63E+07	2.46E+07	3.18E+07	3.65E+07	2.83E+07	1.98E+07	2.06E+07	24.49	25.63	25.74	24.62	25.07	25.39	24.76	24.07	23.91	25.63	25.07	24.07	0.693246	0.390954	0.454316
1286	O75864	Protein ph	PPP1R37	11	23.6	1.89E+07	1.26E+07	9.36E+06	9.65E+06	1.22E+07	6.80E+06	1.89E+07	9.46E+06	1.31E+07	24.15	23.78	23.43	23.22	23.69	22.94	24.16	22.94	23.24	23.78	23.22	23.24	0.357414	0.379604	0.637181
1944	P60953	Cell divisio	CDC42	3	25.1	8.36E+06	2.11E+07	2.70E+07	1.01E+07	2.08E+07	1.36E+07	1.26E+07	1.19E+07		22.95	24.47	24.89	23.27	24.48	23.91	23.5	23.31		24.47	23.91	23.41	1.019923	0.605448	0.138518
3209	Q9Y376	Calcium-bi	CAB39	6	17.6	2.46E+06	3.54E+06	3.80E+06	2.39E+06	2.46E+06	3.08E+06	2.36E+06			21.2	22.02	22.2	21.23	21.45	21.85	21.1			22.02	21.45	21.1	0.535042	0.317117	
3171	Q9UNZ2	NSFL1 cofa	NSFL1C	5	20	3.66E+06	4.48E+06	4.46E+06			2.87E+06				21.76	22.34	22.4			21.77				22.34	21.77		0.351629		
1695	P33176	Kinesin-1 h	KIF5B	49	57.1	5.62E+08	6.75E+08	5.48E+08	5.24E+08	4.35E+08	4.93E+08	3.86E+08	5.34E+08	2.83E+08	29.08	29.67	29.55	28.98	28.88	29.26	28.39	28.6	27.24	29.55	28.98	28.39	0.308601	0.195007	0.730361
829	F8WJN3	Cleavage a	CPSF6	9	23.2	2.60E+07	2.00E+07	2.29E+07	1.62E+07	1.36E+07	2.01E+07	1.73E+07	1.40E+07	1.09E+07	24.58	24.39	24.68	24.01	23.85	24.5	24.03	23.52	22.96	24.58	24.01	23.52	0.147522	0.336768	0.533543
1135	O14745	Na(+)/H(+)	SLC9A3R1	4	19.8		4.58E+06	4.12E+06	3.57E+06		2.89E+06	2.66E+06				22.38	22.29	21.75		21.78	21.27			22.34	21.76	21.27	0.063573	0.019233	
1946	P61081	NEDD8-cor	UBE2M	5	30.1	1.07E+07	1.19E+07	5.39E+06	6.93E+06	6.11E+06	6.97E+06	5.19E+06	5.44E+06	7.32E+06	23.33	23.7	22.69	22.75	22.75	23	22.27	22.13	22.31	23.33	22.75	22.27	0.510415	0.141894	0.093305
2336	Q32P28-4	Prolyl 3-hy	LEPRE1	5	9		4.38E+06	4.99E+06	3.10E+06	3.30E+06	3.64E+06					22.31	22.56	21.59	21.86	22.1				22.44	21.86		0.175643	0.255706	
1605	P24534	Elongation	EEF1B2	8	48	1.31E+08	2.90E+08	4.01E+08	2.42E+08	2.31E+08	2.87E+08	1.96E+08	3.26E+08	7.12E+07	26.99	28.53	29.11	27.95	27.95	28.52	27.58	27.89	25.51	28.53	27.95	27.58	1.092762	0.328324	1.294006
1369	P01891	HLA class I	HLA-A	9	31.5	2.34E+07	2.99E+07	2.58E+07	1.94E+07	1.80E+07	2.41E+07	1.58E+07	1.43E+07		24.44	24.98	24.84	24.25	24.26	24.72	23.86	23.57		24.84	24.26	23.72	0.28184	0.269995	0.206474
589	D6RA82	Annexin	ANXA3	4	15.8	2.72E+06	2.76E+06	3.71E+06	1.74E+06	1.79E+06	2.87E+06	2.10E+06		2.39E+06	21.33	21.62	22.16	20.72	21.04	21.77	20.84		20.55	21.62	21.04	20.7	0.418322	0.541157	0.204449
2005	P62906	60S riboso	RPL10A	9	40.6	7.01E+07	6.36E+07	9.40E+07	4.61E+07	4.33E+07	7.86E+07	2.95E+07	2.82E+07	1.72E+07	26.04	26.18	26.86	25.6	25.52	26.49	24.8	24.57	23.66	26.18	25.6	24.57	0.440247	0.540739	0.601856
1508	P13489	Ribonuclea	RNH1	11	31.2	2.63E+07	3.20E+07	2.70E+07	1.99E+07	1.61E+07	2.63E+07	1.67E+07	1.41E+07	8.89E+06	24.6	25.08	24.89	24.3	24.1	24.84	23.96	23.53	22.6	24.89	24.3	23.53	0.243749	0.378915	0.694196
1132	O14737	Programm	PDCD5	7	40.8	7.46E+06	1.16E+07	1.17E+07	5.64E+06	7.84E+06	9.26E+06	7.24E+06	4.89E+06		22.76	23.66	23.74	22.44	23.07	23.4	22.73	22.01		23.66	23.07	22.37	0.543384	0.48398	0.511112
2774	Q96QK1	Vacuolar p	VPS35	12	17.8	2.36E+07	3.29E+07	3.63E+07	1.79E+07	2.22E+07	3.45E+07	2.46E+07	1.96E+07	1.55E+07	24.46	25.13	25.34	24.14	24.55	25.3	24.55	24.04	23.51	25.13	24.55	24.04	0.458839	0.588836	0.52085
1477	P11047	Laminin su	LAMC1	9	7.1	7.17E+06	9.92E+06	8.73E+06	8.26E+06	6.10E+06	5.76E+06	7.53E+06	5.67E+06		22.71	23.42	23.33	22.97	22.74	22.73	22.76	22.21		23.33	22.74	22.49	0.388176	0.136436	0.391176
59	A0A087W\	Clathrin he	CLTC	75	50.6	5.54E+08	7.75E+08	9.25E+08	6.04E+08	7.10E+08	1.05E+09	3.98E+08	6.74E+08	2.43E+08	29.04	30.1	30.6	29.16	29.51	30.37	28.48	28.9	27.08	30.1	29.51	28.48	0.795719	0.62611	0.956832
2601	Q8NHP8	Putative pl	PLBD2	5	10.9	8.72E+06	1.17E+07	1.06E+07	7.92E+06	7.38E+06	7.71E+06	1.27E+07	1.16E+07	8.58E+06	23.01	23.68	23.59	22.92	23.01	23.15	23.52	23.24	22.54	23.59	23.01	23.24	0.364142	0.118276	0.505638
1971	P62140	Serine/thre	PPP1CB	2	27.8	2.65E+06	4.91E+06	4.17E+06		2.11E+06	4.03E+06		2.00E+06		21.3	22.46	22.31		21.22	22.23		20.78		22.31	21.72	20.78	0.633428	0.716607	
402	B7WP74	Pre-mRNA-	CWC22	4	5.9		4.95E+07	4.27E+07	3.05E+07		3.24E+07					25.72	25.58	24.89		25.23				25.65	25.06		0.10371	0.238941	
1118	O00487	26S protea	PSMD14	9	47.1	5.27E+07	6.06E+07	5.12E+07	4.51E+07	3.26E+07	3.55E+07	2.35E+07	5.74E+07	2.76E+07	25.62	26.09	25.92	25.56	25.12	25.33	24.48	25.58	24.25	25.92	25.33	24.48	0.24103	0.221502	0.712543
1538	P16615-5	Sarcoplasn	ATP2A2	26	35.4	1.84E+08	1.71E+08	1.20E+08	1.13E+08	1.47E+08	1.06E+08	2.07E+08	1.01E+08	2.07E+08	27.58	27.75	27.19	26.85	27.32	26.99	27.69	26.38	26.87	27.58	26.99	26.87	0.287623	0.242321	0.658572
39	A0A087WL	Heterogen	HNRNPDL	5	18.5	5.45E+06	9.20E+06	8.69E+06	5.95E+06	6.02E+06	1.01E+07	5.26E+06	6.04E+06		22.29	23.31	23.31	22.52	22.72	23.5	22.29	22.29		23.31	22.72	22.29	0.591529	0.515648	1.32E-05
2387	Q5T6H7	Xaa-Pro an	XPNPEP1	2	6		2.98E+06	5.17E+06		2.33E+06	2.96E+06					21.75	22.6		21.37	21.81				22.18	21.59		0.60104	0.30789	
231	A0A0C4DG	Deoxyuridi	DUT	6	53.9	2.81E+07	4.18E+07	5.47E+07	3.11E+07	3.29E+07	2.71E+07	3.42E+07	2.42E+07	1.04E+07	24.69	25.54	25.99	24.94	25.14	24.92	25.01	24.35	22.87	25.54	24.94	24.35	0.658837	0.122769	1.098558
1763	P41252	Isoleucine-	IARS	61	49	1.62E+09	9.63E+08	1.17E+09	1.04E+09	1.08E+09	1.49E+09	3.58E+08	6.90E+08	3.32E+08	31.11	30.53	30.92	30.14	30.32	31.05	28.27	28.92	27.47	30.92	30.32	28.27	0.296337	0.480534	0.729277
204	A0A0B4J1\	N-alpha-ac	NAA15	19	23.4	1.54E+07	1.73E+07	1.82E+07	1.28E+07	1.06E+07	1.79E+07	1.36E+07	1.20E+07		23.82	24.23	24.36	23.63	23.5	24.36	23.64	23.32		24.23	23.63	23.48	0.280494	0.462896	0.224036
2042	P82933	28S riboso	MRPS9	4	12.6		5.54E+06	6.79E+06	3.82E+06	5.67E+06	3.91E+06					22.61	22.96	21.87	22.63	22.18				22.78	22.18		0.249263	0.385619	
3035	Q9NVE7	Pantothen	PANK4	21	31.6	2.78E+07	4.39E+07	4.51E+07	3.17E+07	2.68E+07	2.95E+07	3.59E+07	4.36E+07	5.48E+06	24.67	25.6	25.67	25	24.85	25.07	25.12	25.15	21.83	25.6	25	25.12	0.558861	0.111861	1.910828
607	D6RGG3	Collagen al	COL12A1	13	5.6	4.86E+06	8.66E+06	5.63E+06	4.67E+06	3.52E+06	4.02E+06	1.76E+06			22.14	23.22	22.77	22.16	21.94	22.22	20.64			22.77	22.16	20.64	0.541888	0.147184	
1798	P46940	Ras GTPas	IQGAP1	57	43.6	8.25E+07	1.20E+08	1.24E+08	8.98E+07	8.29E+07	9.78E+07	5.84E+07	6.52E+07	1.36E+07	26.3	27.18	27.25	26.58	26.49	26.81	25.77	25.78	23.29	27.18	26.58	25.77	0.53109	0.166606	1.433862
1178	O15498-2	Synaptobre	YKT6	2	18.3		2.11E+06			1.43E+06						21.31			20.71					21.31	20.71				
1276	O75643	U5 small n	SNRNP200	42	24.8	8.66E+07	8.25E+07	7.83E+07	5.97E+07	5.40E+07	6.04E+07	5.05E+07	3.72E+07		26.39	26.53	26.56	25.93	25.84	26.06	25.58	24.96		26.53	25.93	25.27	0.090486	0.110639	0.434266
1359	P00966	Argininosu	ASS1	21	55.3	3.65E+08	4.20E+08	3.55E+08	3.03E+08	3.36E+08	3.86E+08	2.88E+08	3.03E+08	3.38E+08	28.54	29.06	29.04	28.23	28.44	28.87	28.01	27.83	27.5	29.04	28.44	27.83	0.2951	0.330408	0.25896
1014	J3KRG2	Gasdermin	GSDMA	4	24.5			4.02E+06	3.23E+06								22.26	21.65						22.26	21.65				
1418	P07237	Protein dis	P4HB	22	48	1.54E+08	1.14E+08	1.68E+08	1.49E+08	9.01E+07	8.72E+07	1.58E+08	1.68E+08	2.10E+08	27.25	27.04	27.73	27.33	26.64	26.63	27.23	27.09	26.89	27.25	26.64	27.09	0.35045	0.399995	0.167845
3052	Q9NXG2	THUMP do	THUMPD1	4	18.7		3.31E+06	3.49E+06			2.14E+06	2.44E+06	2.66E+06			21.93	22.02			21.36	21.14	21.19		21.97	21.36	21.16	0.063506		0.034769
2980	Q9HB71	Calcyclin-b	CACYBP	10	51.8	4.36E+07	7.16E+07	6.63E+07	4.87E+07	3.04E+07	6.61E+07	4.69E+07	4.55E+07	2.15E+07	25.38	26.35	26.29	25.68	25.01	26.21	25.45	25.24	23.96	26.29	25.68	25.24	0.542644	0.598831	0.802374
1650	P50148	Guanine nu	GNAQ	2	5.6		1.22E+06	1.74E+06		1.01E+06	8.35E+05					20.57	21.09		20.24	20.18				20.83	20.21		0.362365	0.041769	
2825	Q9BQG0	Myb-bindi	MYBBP1A	15	12.5	1.11E+07	1.40E+07	1.26E+07	5.98E+06	9.38E+06	8.17E+06	9.70E+06	6.37E+06	6.08E+06	23.39	23.91	23.84	22.55	23.32	23.22	23.15	22.36	21.99	23.84	23.22	22.36	0.280566	0.420393	0.593817
2507	Q86UP2-2	Kinectin	KTN1	25	24.8	3.22E+07	3.32E+07	3.51E+07	2.34E+07	2.21E+07	2.72E+07	2.13E+07	1.57E+07		24.96	25.16	25.29	24.53	24.54	24.92	24.36	23.71		25.16	24.54	24.03	0.165704	0.222053	0.461164
1992	P62701	40S riboso	RPS4X	19	58.2	6.90E+07	8.07E+07	1.15E+08	5.41E+07	5.53E+07	7.93E+07	4.97E+07	5.55E+07	1.98E+07	25.99	26.51	27.09	25.8	25.89	26.5	25.56	25.54	23.84	26.51	25.89	25.54	0.545976	0.382529	0.990835
2439	Q6P2Q9	Pre-mRNA-	PRPF8	54	27.3	8.67E+07	9.26E+07	8.98E+07	6.66E+07	6.25E+07	7.65E+07	4.48E+07	4.51E+07	1.54E+07	26.41	26.71	26.77	26.08	26.09	26.44	25.37	25.22	23.49	26.71	26.09	25.22	0.190507	0.205611	1.0443
668	E7EX17	Eukaryotic	EIF4B	54	69	4.06E+10	1.71E+10	1.94E+10	3.64E+10	3.55E+10	2.80E+10	4.66E+10	3.69E+10	4.14E+10	35.71	35.15	35.81	35.03	35.09	35.78	34.98	34.87	33.58	35.71	35.09	34.87	0.354631	0.420075	0.776734
2010	P62995-3	Transform	TRA2B	6	46.8	4.39E+07	6.24E+07	5.81E+07	2.38E+07	4.13E+07	4.33E+07	1.72E+07	2.42E+07		25.39	26.14	26.07	24.55	25.45	25.63	24.02	24.35		26.07	25.45	24.19	0.417309	0.580906	0.237571
327	F6WIT2	Serine/thre	PPP2R4	4	16	4.45E+06	6.07E+06	6.67E+06		2.95E+06	4.80E+06	3.55E+06	3.07E+06	2.55E+06	22.03	22.71	22.93		21.72	22.45	21.69	21.39	20.68	22.71	22.08	21.39	0.470216	0.512913	0.521698
3168	Q9UNM6	26S protea	PSMD13	14	39.6	8.67E+07	8.76E+07	7.36E+07	5.75E+07	5.70E+07	3.57E+07	5.15E+07	6.88E+07	3.71E+07	26.41	26.62	26.5	25.88	25.94	25.35	25.6	25.84	24.7	26.5	25.88	25.6	0.102221	0.322541	0.59884
1624	P26373	60S riboso	RPL13	5	24.2	3.34E+07	4.44E+07	3.32E+07	1.98E+07	2.20E+07	2.74E+07	5.18E+06	6.93E+06		24.99	25.62	25.16	24.29	24.53	24.94	22.26	22.49		25.16	24.53	22.37	0.322957	0.327506	0.157051
82	A0A087W)	ATPase ASI	ASNA1	4	12.7		3.94E+06	4.55E+06			2.72E+06	2.64E+06				22.2	22.43			21.69	21.23			22.32	21.69	21.23	0.158685		
2488	Q7Z2W4	Zinc finger	ZC3HAV1	6	8.8	2.78E+06	4.09E+06	3.60E+06	2.29E+06	2.59E+06	2.36E+06	2.51E+06	2.59E+06	3.03E+06	21.36	22.25	22.1	21.14	21.51	21.48	21.18	21.12	20.96	22.1	21.48	21.12	0.476565	0.204474	0.112958
1287	O75874	Isocitrate c	IDH1	10	30.7	1.40E+07	2.32E+07	2.21E+07	1.06E+07	1.50E+07	1.68E+07	1.10E+07	9.62E+06		23.68	24.61	24.64	23.34	23.99	24.25	23.3	22.98		24.61	23.99	23.14	0.544744	0.465465	0.227001

1750	P40261	Nicotinami	NNMT	3	13.3	3.90E+06	5.81E+06	5.25E+06			3.44E+06				21.86	22.65	22.64			22.02				22.64	22.02		0.454158		
1860	P50570-5	Dynamin-2	DNM2	17	23.1	1.25E+07	2.13E+07	1.66E+07	1.22E+07	1.14E+07	1.43E+07	1.14E+07	1.00E+07		23.55	24.49	24.22	23.55	23.59	23.98	23.35	23.03		24.22	23.59	23.19	0.483743	0.240466	0.229658
2234	Q14694	Ubiquitin c	USP10	8	14.8	4.78E+06	9.33E+06	6.48E+06	3.73E+06	4.43E+06	4.96E+06	4.12E+06	5.52E+06	3.86E+06	22.11	23.33	22.91	21.84	22.28	22.5	21.95	22.17	21.23	22.91	22.28	21.95	0.619154	0.336507	0.493066
1838	P49736	DNA replic	MCM2	19	29.6	2.14E+07	2.39E+07	2.57E+07	1.42E+07	1.54E+07	2.60E+07	1.03E+07	6.25E+06		24.3	24.65	24.83	23.79	24.02	24.81	23.23	22.33		24.65	24.02	22.78	0.270304	0.537189	0.636581
2274	Q15365	Poly(rC)-bi	PCBP1	10	67.1	2.68E+08	1.81E+08	1.43E+08	1.38E+08	1.88E+08	1.09E+08	1.55E+08	2.27E+08	2.12E+08	28.06	27.88	27.49	27.25	27.63	27.03	27.22	27.54	26.92	27.88	27.25	27.22	0.292222	0.301284	0.309855
47	A0A087WU	Eukaryotic	EIF5B	13	16.1	7.58E+06	1.68E+07	1.48E+07	5.83E+06	9.90E+06	1.19E+07	4.33E+06	6.18E+06		22.78	24.18	24.03	22.49	23.4	23.68	22.02	22.32		24.03	23.4	22.17	0.768916	0.622004	0.213885
882	H0Y7A7	Calmodulir	CALM2	8	67.9	5.23E+07	7.31E+07	6.64E+07	5.46E+07	4.68E+07	4.29E+07	4.73E+07	2.97E+07	3.64E+07	25.6	26.37	26.3	25.81	25.66	25.61	25.46	24.65	24.68	26.3	25.66	24.68	0.423239	0.105485	0.457597
1580	P22033	Methylmal	MUT	7	12.4	5.93E+06	6.29E+06	6.81E+06	4.19E+06	4.04E+06	5.59E+06	4.52E+06	4.25E+06		22.42	22.76	22.96	22.01	22.13	22.69	22.07	21.82		22.76	22.13	21.95	0.274191	0.360819	0.180707
2646	Q8WWY3	U4/U6 sma	PRPF31	23	55.7	2.98E+08	3.05E+08	2.36E+08	2.72E+08	2.11E+08	1.33E+08	1.07E+09	6.55E+08	2.88E+08	28.14	28.66	28.44	28.13	27.8	27.41	30.03	28.83	27.28	28.44	27.8	28.83	0.259135	0.362405	1.376855
2794	Q99436	Proteasom	PSMB7	4	22	1.76E+07	2.50E+07	3.05E+07	1.74E+07	2.26E+07	1.32E+07	2.01E+07	2.20E+07		24.04	24.73	25.07	24.09	24.57	23.87	24.26	24.2		24.73	24.09	24.23	0.526771	0.358204	0.037791
98	A0A087WV	Prostaglan	PTGES3	5	34.1	1.09E+07	1.58E+07	1.71E+07	4.79E+06	1.02E+07	1.27E+07		6.65E+06		23.36	24.09	24.26	22.19	23.45	23.81		22.41		24.09	23.45	22.41	0.475953	0.852893	
1928	P55884	Eukaryotic	EIF3B	27	40.9	1.00E+08	1.97E+08	1.44E+08	9.81E+07	1.03E+08	1.18E+08	8.54E+07	9.85E+07	6.21E+06	26.67	27.99	27.5	26.67	26.85	27.15	26.34	26.33	22.07	27.5	26.85	26.33	0.666015	0.242453	2.463155
3027	Q9NTZ6	RNA-bindir	RBM12	11	13.1	4.56E+06	9.36E+06	4.72E+06	2.93E+06	4.15E+06	3.06E+06	5.05E+06			22.05	23.34	22.49	21.51	22.15	21.84	22.22			22.49	21.84	22.22	0.654914	0.319009	
753	F5GXU9	2-oxoisova	BCKDHA	2	5.5		1.40E+06		9.79E+05	9.32E+05		1.05E+06	1.20E+06			20.7		19.93	20.18		19.76	19.97		20.7	20.05	19.86		0.179067	0.148168
2669	Q92665	28S riboso	MRPS31	3	10.4		1.21E+06	1.58E+06			7.41E+05					20.55	20.9			20.08				20.72	20.08		0.241657		
1058	V9GZ55	Proteasom	PSMG2	2	9.1			2.66E+06			1.72E+06						21.64			20.99				21.64	20.99				
1324	O95400	CD2 antige	CD2BP2	5	15		3.80E+06	6.33E+06			3.11E+06					22.15	22.89			21.87				22.52	21.87		0.522248		
907	H0YKD8	60S riboso	RPL28	8	36.5	6.25E+07	5.12E+07	4.85E+07	1.12E+07	3.41E+07	4.51E+07	1.62E+07	2.37E+07		25.83	25.81	25.84	23.41	25.18	25.67	23.91	24.32		25.83	25.18	24.12	0.014879	1.19054	0.295355
1450	P09211	Glutathion	GSTP1	11	57.6	2.09E+08	3.19E+08	2.50E+08	1.36E+08	2.22E+08	2.05E+08	1.80E+08	1.38E+08	8.09E+07	27.79	28.73	28.52	27.21	27.87	28.05	27.43	26.81	25.72	28.52	27.87	26.81	0.497494	0.440644	0.863466
383	B4DUC8	S-methyl-5	MTAP	5	20.3	1.29E+07	1.04E+07	1.22E+07	8.04E+06	6.62E+06	9.53E+06	7.73E+06			23.59	23.49	23.78	22.94	22.85	23.43	22.83			23.59	22.94	22.83	0.147461	0.311488	
603	D6REX3	Protein tra	SEC31A	12	13.3	5.28E+07	6.21E+07	5.72E+07	3.05E+07	3.99E+07	5.07E+07	5.37E+07	3.54E+07		25.62	26.12	26.05	24.89	25.4	25.81	25.64	24.88		26.05	25.4	25.26	0.270971	0.460464	0.534578
209	K7EM56	40S riboso	RPS15	3	52.7			8.91E+06			5.60E+06						23.34			22.69				23.34	22.69				
61	A0A087WV	DNA-direct	POLR2E	3	21.7	5.71E+06	2.12E+06				2.05E+06				22.36	21.32				21.19				21.84	21.19		0.73812		
2094	Q05639	Elongation	EEF1A2	5	51.4	2.11E+07	6.83E+07	7.70E+07	2.77E+07	4.61E+07	6.22E+07	6.72E+07	3.66E+07		24.28	26.28	26.55	24.78	25.63	26.11	25.91	24.93		26.28	25.63	25.42	1.238736	0.674732	0.690107
2798	Q99497	Protein de	PARK7	8	55	9.17E+07	6.68E+07	1.62E+07	4.54E+07	5.83E+07	2.83E+07	2.16E+07	1.43E+07	5.78E+07	26.56	26.24	24.17	25.58	25.97	25	24.37	23.57	25.25	26.24	25.58	24.37	1.29664	0.488576	0.837986
1876	P51636-2	Caveolin-2	CAV2	3	30.9	6.84E+07	4.86E+07	4.78E+07		4.23E+07	2.56E+07	1.62E+07	1.88E+07		25.98	25.69	25.8		25.49	24.79	23.91	23.97		25.8	25.14	23.94	0.142494	0.496762	0.044903
1296	O76094	Signal reco	SRP72	6	11.5	1.21E+07	1.23E+07	1.39E+07	8.40E+06	7.95E+06	1.79E+07	1.59E+07	9.42E+06		23.5	23.75	23.95	23	23.09	24.36	23.87	22.93		23.75	23.09	23.4	0.2262	0.761887	0.664467
2804	Q99613-2	Eukaryotic	EIF3C	26	33.4	1.96E+08	2.44E+08	1.96E+08	1.34E+08	1.53E+08	1.42E+08	5.40E+07	8.77E+07	2.48E+07	27.68	28.29	28.03	27.2	27.37	27.5	25.65	26.23	24.11	28.03	27.37	25.65	0.306143	0.150933	1.093339
2512	Q86VP6	Cullin-asso	CAND1	37	33.9	1.87E+08	2.35E+08	1.98E+08	1.39E+08	1.59E+08	1.73E+08	1.29E+08	1.51E+08	1.14E+08	27.61	28.21	28.08	27.26	27.41	27.78	26.97	26.93	26.18	28.08	27.41	26.93	0.313158	0.268652	0.447796
114	E9PLA9	Caprin-1	CAPRIN1	6	32.8	1.83E+07	1.58E+07	1.83E+07	1.15E+07	1.02E+07	1.37E+07	8.83E+06	7.15E+06	8.71E+06	24.11	24.09	24.36	23.45	23.45	23.93	23.02	22.53	22.57	24.11	23.45	22.57	0.152978	0.278083	0.27191
1353	P00491	Purine nuc	PNP	11	57.4	3.90E+07	5.03E+07	4.61E+07	3.16E+07	3.15E+07	3.81E+07	2.98E+07	1.22E+07		25.2	25.77	25.72	24.98	25.06	25.43	24.81	23.35		25.72	25.06	24.08	0.318385	0.243551	1.035435
1101	O00231	26S protea	PSMD11	24	55.7	1.81E+08	2.14E+08	1.97E+08	1.70E+08	1.43E+08	1.31E+08	1.27E+08	1.54E+08	7.32E+07	27.53	28.1	28.06	27.43	27.24	27.39	26.95	26.96	25.57	28.06	27.39	26.95	0.314593	0.099279	0.798778
583	D3DQV9	Eukaryotic	EIF4G2	9	11	1.69E+06	4.75E+06	4.18E+06	1.83E+06	2.79E+06	2.82E+06		1.98E+06		20.58	22.42	22.32	20.79	21.65	21.75		20.76		22.32	21.65	20.76	1.032712	0.525034	
2748	Q96J01	THO comp	THOC3	7	26.2	3.16E+07	2.98E+07	2.62E+07	2.43E+07	1.71E+07	1.69E+07	1.73E+07	1.45E+07	8.63E+06	24.92	24.97	24.87	24.59	24.2	24.25	24.03	23.6	22.56	24.92	24.25	23.6	0.052294	0.209931	0.754694
2375	Q5LJA5	Ubiquitin c	UCHL5	5	14.6	4.94E+06	6.41E+06	7.40E+06	3.89E+06	4.05E+06	4.23E+06	4.42E+06			22.17	22.8	23.1	21.89	22.13	22.29	22.04			22.8	22.13	22.04	0.477339	0.200692	
2388	Q5T6V5	UPF0553 p	C9orf64	6	15.5	8.92E+06	6.89E+06	7.30E+06	5.46E+06	4.46E+06	5.74E+06	5.84E+06	5.81E+06	5.60E+06	23.05	22.9	23.08	22.39	22.29	22.71	22.45	22.24	21.87	23.05	22.39	22.24	0.096326	0.22197	0.293171
2090	Q04917	14-3-3 pro	YWHAH	7	41.5	4.02E+07	3.82E+07	3.47E+07	1.81E+07	2.28E+07	2.75E+07	1.61E+07	3.35E+07		25.25	25.38	25.25	24.17	24.58	24.95	23.89	24.81		25.25	24.58	24.35	0.07699	0.392796	0.653195
1219	O60341	Lysine-spe	KDM1A	11	16	4.94E+06	8.07E+06	8.70E+06	4.93E+06	4.99E+06	5.15E+06	5.61E+06	4.75E+06	2.57E+06	22.17	23.12	23.32	22.23	22.46	22.57	22.4	21.97	20.69	23.12	22.46	21.97	0.616442	0.169221	0.886225
2212	Q14166	Tubulin--ty	TTLL12	6	14.4	7.84E+06	1.11E+07	1.19E+07	6.91E+06	6.86E+06	7.87E+06	7.68E+06			22.83	23.59	23.75	22.75	22.92	23.17	22.8			23.59	22.92	22.8	0.494941	0.214579	
1661	P30101	Protein dis	PDIA3	18	42.8	4.01E+07	4.09E+07	6.02E+07	2.87E+07	2.20E+07	3.79E+07	1.90E+07	2.01E+07	1.23E+07	25.24	25.49	26.12	24.82	24.53	25.42	24.17	24.09	23.13	25.49	24.82	24.09	0.454602	0.45367	0.578924
3003	Q9NQG5	Regulation	RPRD1B	4	16.3	2.47E+06	2.91E+06	2.37E+06	1.80E+06	1.57E+06	2.21E+06	2.34E+06			21.2	21.72	21.47	20.76	20.8	21.41	21.08			21.47	20.8	21.08	0.259522	0.360258	
2765	Q96P70	Importin-9	IPO9	16	18.1	3.90E+07	4.59E+07	3.96E+07	2.45E+07	2.63E+07	2.89E+07	2.47E+07	1.90E+07	1.67E+07	25.2	25.64	25.49	24.61	24.81	25.02	24.56	23.98	23.63	25.49	24.81	23.98	0.226132	0.208314	0.468061
2316	Q16555-2	Dihydropy	DPYSL2	11	32.3	8.07E+06	1.02E+07	1.54E+07	4.19E+06	6.34E+06	9.19E+06	6.99E+06	7.30E+06	4.03E+06	22.89	23.47	24.1	22.01	22.8	23.39	22.69	22.56	21.29	23.47	22.8	22.56	0.602773	0.689455	0.7745
113	A0A087X0!	Hypoxia up	HYOU1	23	33.3	3.54E+07	4.12E+07	5.32E+07	2.22E+07	2.67E+07	3.97E+07	2.23E+07	2.15E+07	3.35E+06	25.05	25.51	25.95	24.48	24.84	25.5	24.41	24.17	21.07	25.51	24.84	24.17	0.452178	0.517258	1.865146
1496	P12004	Proliferatir	PCNA	13	67.4	4.40E+08	3.70E+08	2.53E+08	2.68E+08	2.46E+08	2.49E+08	3.19E+08	4.34E+08	2.95E+08	28.77	28.9	28.58	28.1	28.04	28.33	28.15	28.25	27.32	28.77	28.1	28.15	0.162286	0.154136	0.51275
22	A0A087W1	Roundabou	ROBO1	10	9.1	5.12E+06	3.90E+06	4.06E+06	2.98E+06	2.59E+06	3.03E+06				22.2	22.19	22.27	21.53	21.51	21.82				22.2	21.53		0.044042	0.172992	
111	A0A087W2	Splicing fac	SF3B2	16	19.1	3.54E+07	3.82E+07	3.32E+07	1.33E+07	2.08E+07	2.52E+07	6.11E+06	6.56E+06		25.05	25.38	25.16	23.69	24.48	24.75	22.52	22.4		25.16	24.48	22.46	0.1688	0.552695	0.087287
673	E9PB90	Hexokinas	HK2	14	19.8	7.81E+06	7.79E+06	8.76E+06	5.50E+06	4.70E+06	6.60E+06	3.17E+06	4.59E+06		22.82	23.07	23.33	22.4	22.37	22.9	21.56	21.91		23.07	22.4	21.74	0.25652	0.302317	0.252422
1566	P20042	Eukaryotic	EIF2S2	14	40.5	2.34E+07	3.47E+07	2.91E+07	1.47E+07	1.88E+07	2.48E+07	1.20E+07	1.06E+07		24.44	25.24	25.01	23.84	24.33	24.75	23.42	23.1		25.01	24.33	23.26	0.413719	0.452088	0.226033
1639	P28072	Proteasom	PSMB6	4	17.6	1.10E+07	2.00E+07	2.85E+07	1.35E+07	9.82E+06	3.13E+07		2.31E+07		23.38	24.39	24.97	23.71	23.38	25.17		24.27		24.39	23.71	24.27	0.804008	0.951372	
1121	O00629	Importin su	KPNA4	5	18.8	6.95E+06	9.35E+06	6.44E+06	4.82E+06	4.35E+06		4.02E+06			22.65	23.33	22.9	22.2	22.24		21.9			22.9	22.22	21.9	0.342223	0.028932	
1809	P48163	NADP-depe	ME1	7	17.8		7.47E+06	7.61E+06	4.55E+06		5.56E+06	3.11E+06				23.02	23.14	22.12		22.68	21.52			23.08	22.4	21.52	0.0865	0.394229	
606	D6RG15	Twinfilin-2	TWF2	6	44.5	4.50E+06	7.77E+06	6.72E+06	4.91E+06	4.50E+06	4.09E+06	8.20E+06	4.80E+06		22.04	23.07	22.94	22.23	22.31	22.25	22.9	21.98		22.94	22.25	22.44	0.561822	0.042232	0.651423
2707	Q96AY3	Peptidyl-pr	FKBP10	6	12.4	4.83E+06	8.94E+06	9.89E+06		4.73E+06	5.90E+06	5.48E+06			22.13	23.26	23.52		22.38	22.76	22.35			23.26	22.57	22.35	0.738906	0.270062	
1839	P49748-2	Very long-c	ACADVL	11	24	1.70E+07	1.66E+07	1.89E+07	1.15E+07	8.90E+06	1.38E+07	1.39E+07	1.16E+07	1.12E+07	23.98	24.14	24.39	23.45	23.23	23.94	23.69	23.24	22.99	24.14	23.45	23.24	0.205223	0.359281	0.352163
71	J3KRB3	60S riboso	RPL17	3	27.9	1.90E+07	6.12E+06	5.91E+06		2.80E+06	5.21E+06				24.16	22.73	22.81		21.66	22.58				22.81	22.12		0.802047	0.647836	
1425	P07814	Bifunctiona	EPRS	84	53	1.97E+09	1.20E+09	1.69E+09	1.39E+09	1.50E+09	2.18E+09	3.68E+08	8.95E+08	4.03E+08	31.68	30.85	31.6	30.52	30.91	31.72	28.33	29.54	27.83	31.6	30.91	28.33	0.461879	0.61477	0.880973
2072	Q01813	ATP-depen	PFKP	20	31.6	4.97E+07	7.05E+07	8.13E+07	4.21E+07	4.59E+07	7.20E+07	4.67E+07	3.01E+07	1.04E+07	25.54	26.31	26.63	25.44	25.62	26.37	25.44	24.68	22.87	26.31	25.62	24.68	0.56319	0.489965	1.320979
1321	O95373	Importin-7	IPO7	19	20.1	6.81E+07	9.45E+07	9.46E+07	5.88E+07	6.02E+07	7.15E+07	6.26E+07	5.92E+07	5.31E+07	25.97	26.75	26.88	25.91	26.05	26.35	25.85	25.64	25.18	26.75	26.05	25.64	0.489843	0.224624	0.34152
1930	P56192	Methionin	MARS	35	46.9	3.69E+08	3.38E+08	3.20E+08	2.36E+08	2.56E+08	3.63E+08	1.02E+08	2.92E+08	1.86E+08	28.56	28.79	28.88	27.9	28.09	28.78	26.6	27.79	26.72	28.79	28.09	26.72	0.163637	0.463098	0.657786
1614	P25685	DnaJ homo	DNAJB1	11	34.1	1.62E+07	2.68E+07	3.20E+07	1.29E+07	1.64E+07	1.59E+07	1.29E+07	1.74E+07	7.63E+06	23.91	24.85	25.11	23.65	24.14	24.15	23.55	23.85	22.38	24.85	24.14	23.55	0.631783	0.287191	0.774149
1636	P27824	Calnexin	CANX	12	19.9	6.45E+07	1.05E+08	1.20E+08	4.85E+07	6.76E+07	7.67E+07	9.44E+07	7.12E+07	3.00E+07	25.89	26.9	27.19	25.66	26.19	26.46	26.51	25.86	24.35	26.9	26.19	25.86	0.682982	0.406636	1.107786
2826	Q9BRA2	Thioredoxi	TXNDC17	4	38.2	5.41E+06	7.14E+06	9.79E+06	4.52E+06	4.37E+06	7.88E+06	4.05E+06			22.28	22.96	23.51	22.1	22.25	23.18	21.91			22.96	22.25	21.91	0.614904	0.581715	
1668	P30533	Alpha-2-ma	LRPAP1	9	28.3	1.52E+07	2.13E+07	2.09E+07	1.51E+07	1.29E+07	1.23E+07	2.00E+07	1.09E+07	4.90E+06	23.8	24.49	24.55	23.88	23.78	23.75	24.24	23.14	21.66	24.49	23.78	23.14	0.416596	0.068826	1.298037
1211	O60231	Putative pr	DHX16	4	5.9	1.61E+06	2.34E+06	2.50E+06	9.94E+05	1.43E+06	1.43E+06				20.52	21.42	21.55	20.08	20.71	20.75				21.42	20.71		0.558172	0.375538	
948	H7BXV5	Collagen al	COL18A1	3	5.4			2.05E+06	1.18E+06	1.39E+06	1.21E+06						21.3	20.25	20.67	20.59				21.3	20.59			0.220511	
2480	Q7L0Y3	Mitochond	TRMT10C	6	18.1	5.18E+06	5.96E+06	5.98E+06			3.32E+06	3.97E+06	4.13E+06		22.22	22.68	22.82			21.96	21.87	21.78		22.68	21.96	21.82	0.314414		0.062198
1893	P52788	Spermine s	SMS	4	11.7	4.55E+06	6.37E+06	5.90E+06		3.79E+06	3.65E+06	4.08E+06			22.04	22.79	22.81		22.04	22.1	21.93			22.79	22.07	21.93	0.43684	0.041144	
1567	P20073-2	Annexin A7	ANXA7	4	7.9		1.79E+06	3.58E+06			1.57E+06	1.38E+06		1.96E+06		21.07	22.09			20.86	20.26		20.07	21.58	20.86	20.16	0.722482		0.129512
2118	Q08945	FACT comp	SSRP1	16	27.8	3.07E+07	3.99E+07	4.35E+07	2.68E+07	2.43E+07	3.69E+07	3.80E+07	3.41E+07	2.01E+07	24.87	25.45	25.59	24.73	24.65	25.4	25.18	24.86	23.86	25.45	24.73	24.86	0.381709	0.412045	0.684377
1980	P62277	40S riboso	RPS13	9	40.4	3.92E+07	6.69E+07	8.87E+07	4.03E+07	4.35E+07	6.79E+07	3.41E+07	3.07E+07		25.21	26.25	26.75	25.36	25.53	26.27	25	24.73		26.25	25.53	24.87	0.78363	0.482726	0.192667
1430	P07954-2	Fumarate l	FH	13	39.8	3.45E+07	5.65E+07	4.46E+07	2.11E+07	2.85E+07	4.06E+07	2.90E+07	2.68E+07		25.03	26.01	25.66	24.4	24.94	25.53	24.78	24.49		25.66	24.94	24.63	0.498619	0.566598	0.205053
2241	Q14914-2	Prostaglan	PTGR1	5	25.9	3.73E+06	6.08E+06	4.46E+06	2.98E+06	2.83E+06	5.55E+06	3.96E+06		6.09E+06	21.8	22.71	22.4	21.53	21.68	22.67	21.86		22	22.4	21.68	21.93	0.463605	0.620446	0.096021
2405	Q5VTR2	E3 ubiquiti	RNF20	9	13.5	4.18E+06	5.37E+06	5.37E+06	2.84E+06	3.27E+06	4.31E+06	3.41E+06			21.95	22.57	22.69	21.47	21.84	22.31	21.65			22.57	21.84	21.65	0.393883	0.420359	
1775	P42704	Leucine-ric	LRPPRC	45	36.1	1.11E+08	1.39E+08	1.45E+08	7.08E+07	9.26E+07	1.02E+08	9.73E+07	1.06E+08	9.65E+06	26.82	27.41	27.52	26.15	26.69	26.9	26.55	26.41	22.73	27.41	26.69	26.41	0.377331	0.383048	2.16929
2821	Q9BQ52-4	Zinc phosp	ELAC2	4	5.5		2.87E+06	2.78E+06		1.36E+06	2.09E+06					21.69	21.7		20.63	21.32				21.7	20.97		0.008271	0.48479	
2223	Q14498-2	RNA-bindir	RBM39	14	35.7	9.72E+07	7.80E+07	8.32E+07	4.88E+07	5.80E+07	5.54E+07	6.69E+07	5.17E+07	2.43E+07	26.65	26.46	26.65	25.69	25.96	25.92	25.9	25.44	24.09	26.65	25.92	25.44	0.10927	0.149064	0.944428

3141	Q9UJW0	Dynactin s	DCTN4	7	24.6	8.71E+06	8.91E+06	9.57E+06	5.96E+06	4.30E+06	6.96E+06	5.43E+06	5.46E+06	5.18E+06	23.01	23.25	23.47	22.53	22.22	22.99	22.35	22.14	21.75	23.25	22.53	22.14	0.23115	0.387862	0.304316
1766	P42126-2	Enoyl-CoA	ECI1	5	23.9	5.35E+06	8.47E+06	9.75E+06	5.43E+06	5.01E+06	7.69E+06	7.69E+06			22.26	23.19	23.49	22.37	22.46	23.15	22.81			23.19	22.46	22.81	0.639427	0.423096	
3069	Q9NZL4	Hsp70-binc	HSPBP1	6	19.9	1.12E+07	1.34E+07	1.30E+07	8.30E+06	8.24E+06	9.59E+06	1.16E+07	1.12E+07		23.41	23.85	23.89	22.98	23.12	23.44	23.38	23.19		23.85	23.12	23.28	0.266216	0.234806	0.132602
2926	Q9H2H8	Peptidyl-pr	PPIL3	5	41.6	7.96E+06	1.24E+07	1.23E+07	7.73E+06	7.50E+06	8.76E+06	7.82E+06			22.87	23.76	23.79	22.9	23.03	23.29	22.85			23.76	23.03	22.85	0.525848	0.203192	
2641	Q8WVY7	Ubiquitin-l	UBLCP1	5	13.8	3.67E+06	5.40E+06	5.39E+06	3.69E+06	3.29E+06	3.07E+06	3.80E+06	3.66E+06		21.77	22.58	22.69	21.82	21.85	21.85	21.81	21.61		22.58	21.85	21.71	0.501118	0.018263	0.138976
1643	Q5VZU9	Tripeptidyl	TPP2	18	20.7	1.11E+07	1.32E+07	1.55E+07	9.00E+06	7.68E+06	1.09E+07	1.66E+07	1.01E+07	3.96E+06	23.39	23.83	24.11	23.1	23.05	23.58	23.95	23.04	21.26	23.83	23.1	23.04	0.360053	0.290106	1.367984
2367	Q5JTZ9	Alanine--tF	AARS2	5	5.8		2.16E+06		1.63E+06	1.02E+06	1.61E+06	1.50E+06				21.34		20.61	20.27	20.88	20.42			21.34	20.61	20.42		0.306044	
3010	Q9NR45	Sialic acid	NANS	3	14.8	4.02E+06	6.00E+06	5.57E+06		3.49E+06	3.36E+06	3.65E+06			21.89	22.69	22.74		21.93	21.98	21.73			22.69	21.95	21.73	0.476025	0.037564	
2124	Q09666	Neuroblast	AHNAK	31	15	1.05E+07	2.45E+07	1.84E+07	1.58E+07	1.00E+07	1.15E+07	1.19E+07	9.10E+06	6.42E+06	23.3	24.69	24.37	23.96	23.42	23.64	23.41	22.88	22.11	24.37	23.64	22.88	0.728415	0.26987	0.650268
1942	P60866	40S riboso	RPS20	4	28.6	1.44E+07	1.94E+07	2.11E+07	1.28E+07	1.11E+07	2.60E+07	1.55E+07	1.76E+07	2.02E+07	23.72	24.37	24.57	23.63	23.54	24.81	23.84	23.86	23.88	24.37	23.63	23.86	0.440315	0.711735	0.017724
109	A0A087WZ	Inositol he	PPIP5K2	7	6.9	3.72E+06	4.93E+06	4.47E+06	3.21E+06	2.81E+06	3.39E+06	5.00E+06	3.69E+06	3.44E+06	21.79	22.47	22.4	21.64	21.67	21.99	22.2	21.62	21.11	22.4	21.67	21.62	0.373359	0.19552	0.550231
480	C9JW69	Regulator	RCC1	6	23.7	4.79E+06	8.29E+06	8.45E+06	3.84E+06	4.90E+06	7.70E+06	4.96E+06	5.79E+06		22.11	23.16	23.28	21.88	22.42	23.15	22.19	22.24		23.16	22.42	22.21	0.644181	0.639206	0.033023
1512	P13674-2	Prolyl 4-hy	P4HA1	10	22.8	1.81E+07	1.39E+07	1.78E+07	1.06E+07	9.01E+06	1.41E+07	1.07E+07	9.39E+06	4.90E+06	24.08	23.9	24.33	23.34	23.24	23.96	23.27	22.93	21.66	24.08	23.34	22.93	0.214399	0.388502	0.850902
2104	Q07021	Compleme	C1QBP	8	33.7	1.78E+08	2.21E+08	2.69E+08	1.67E+08	2.71E+08	1.23E+08	7.36E+07	1.16E+08	2.86E+07	27.5	28.15	28.63	27.41	28.17	27.24	26.07	26.57	24.3	28.15	27.41	26.07	0.57113	0.492977	1.19275
1093	O00151	PDZ and LI	PDLIM1	4	16.4	1.76E+06	1.99E+06	2.73E+06		1.24E+06	1.14E+06	1.95E+06			20.65	21.23	21.67		20.47	20.51	20.79			21.23	20.49	20.79	0.510739	0.03246	
2880	Q9BY44-3	Eukaryotic	EIF2A	6	16.4	3.36E+06	3.95E+06	4.36E+06	2.77E+06	2.48E+06	2.58E+06	3.11E+06	2.52E+06		21.65	22.21	22.37	21.43	21.47	21.62	21.52	21.08		22.21	21.47	21.3	0.381031	0.100089	0.305319
411	B7Z9I1	Medium-cl	ACADM	8	21.6	1.58E+07	1.92E+07	1.74E+07	1.10E+07	1.11E+07	1.31E+07	1.63E+07	1.50E+07		23.86	24.35	24.28	23.38	23.54	23.86	23.93	23.63		24.28	23.54	23.78	0.265531	0.247095	0.206839
1949	P61158	Actin-relat	ACTR3	14	45.7	2.21E+07	4.35E+07	3.47E+07	2.25E+07	2.14E+07	2.85E+07	2.93E+07	3.70E+07	1.79E+07	24.34	25.58	25.25	24.5	24.5	25.01	24.79	24.95	23.7	25.25	24.5	24.79	0.645527	0.29097	0.682434
1906	P53621	Coatomer	COPA	31	28.6	4.16E+07	6.78E+07	6.39E+07	3.34E+07	4.17E+07	4.63E+07	3.83E+07	5.51E+07	2.16E+07	25.29	26.28	26.21	25.11	25.47	25.71	25.18	25.53	23.97	26.21	25.47	25.18	0.551216	0.305141	0.817523
1213	O60256	Phosphoril	PRPSAP2	12	45.8	4.06E+07	5.41E+07	4.04E+07	2.25E+07	4.36E+07	2.55E+07	3.13E+07	3.03E+07	9.94E+06	25.26	25.91	25.52	24.5	25.54	24.78	24.87	24.71	22.78	25.52	24.78	24.71	0.326527	0.536397	1.159187
1631	P27348	14-3-3 pro	YWHAQ	9	49.8	3.64E+07	4.04E+07	4.94E+07	2.64E+07	1.95E+07	3.99E+07	1.88E+07	2.39E+07	1.46E+07	25.08	25.46	25.86	24.71	24.38	25.51	24.15	24.34	23.41	25.46	24.71	24.15	0.392729	0.578843	0.490647
1336	O95757	Heat shock	HSPA4L	12	25	1.50E+07	1.67E+07	1.53E+07	8.48E+06	9.50E+06	1.41E+07	1.39E+07	6.76E+06	2.62E+06	23.79	24.16	24.09	23.01	23.34	23.96	23.69	22.44	20.77	24.09	23.34	22.44	0.19499	0.482098	1.467013
1222	O60493-2	Sorting ne	SNX3	4	30	3.59E+06	6.89E+06	6.99E+06	4.18E+06	4.15E+06	5.23E+06	3.98E+06			21.74	22.9	23	22	22.15	22.59	21.87			22.9	22.15	21.87	0.701507	0.305234	
2906	Q9H074	Polyadenyl	PAIP1	7	16.1	1.51E+07	1.35E+07	1.33E+07	9.00E+06	6.78E+06	1.25E+07	1.16E+07	9.74E+06		23.8	23.86	23.91	23.1	22.9	23.79	23.38	22.99		23.86	23.1	23.18	0.059325	0.46691	0.272187
1658	P30084	Enoyl-CoA	ECHS1	5	22.1	4.72E+06	7.87E+06	8.01E+06	3.99E+06	4.55E+06	7.47E+06	4.61E+06	4.43E+06		22.09	23.09	23.22	21.94	22.33	23.1	22.1	21.86		23.09	22.33	21.98	0.618287	0.591166	0.170177
2211	Q14152	Eukaryotic	EIF3A	42	31.8	1.91E+08	3.51E+08	3.13E+08	2.22E+08	2.52E+08	2.27E+08	2.69E+08	2.88E+08	9.45E+06	27.64	28.83	28.82	27.77	28.06	28.12	27.94	27.78	22.7	28.82	28.06	27.78	0.684533	0.189694	2.979248
2674	Q92797	Symplekin	SYMPK	3	5.2		2.04E+06	2.15E+06	1.29E+06	1.28E+06	1.72E+06					21.26	21.36	20.33	20.55	20.99				21.31	20.55		0.068523	0.333515	
834	G3V1C3	Apoptosis	API5	7	15.5	9.18E+06	5.48E+06	9.04E+06	4.63E+06	4.60E+06	7.57E+06	3.98E+06	4.19E+06	4.55E+06	23.1	22.59	23.37	22.15	22.34	23.12	21.87	21.81	21.51	23.1	22.34	21.81	0.396274	0.514955	0.191604
1424	P07741	Adenine pl	APRT	8	58.9	1.69E+07	2.88E+07	2.37E+07	1.60E+07	1.39E+07	1.60E+07	1.36E+07	1.30E+07	1.24E+07	23.97	24.94	24.75	23.99	23.88	24.16	23.64	23.42	23.14	24.75	23.99	23.42	0.514478	0.140348	0.249176
295	A2A2D0	Stathmin	STMN1	4	51.2	1.54E+07	2.33E+07	2.15E+07	1.16E+07	1.34E+07	2.24E+07	1.74E+07	1.33E+07	1.01E+07	23.82	24.62	24.59	23.46	23.83	24.63	24.04	23.45	22.81	24.59	23.83	23.45	0.454023	0.597415	0.613392
2008	P62937	Peptidyl-pr	PPIA	14	75.2	5.96E+08	8.87E+08	1.01E+09	5.04E+08	7.52E+08	8.15E+08	4.70E+08	5.26E+08	3.57E+08	29.23	30.4	30.68	28.88	29.63	29.85	28.78	28.53	27.61	30.4	29.63	28.53	0.767885	0.509273	0.616558
1975	P62249	40S riboso	RPS16	10	57.5	1.58E+08	1.92E+08	2.21E+08	1.05E+08	1.36E+08	1.86E+08	9.78E+07	1.18E+08	5.88E+07	27.27	27.95	28.27	26.75	27.19	27.92	26.56	26.62	25.27	27.95	27.19	26.56	0.506425	0.589581	0.7655
711	H7BXH2	Serine/thr	PPP6R3	5	7	1.49E+06	3.52E+06	3.84E+06	1.84E+06	2.16E+06	2.63E+06	2.12E+06	1.90E+06		20.44	22.02	22.21	20.82	21.25	21.64	20.88	20.71		22.02	21.25	20.8	0.971668	0.412489	0.119149
2610	Q8TCF1	AN1-type z	ZFAND1	4	16	5.66E+06	7.88E+06	8.06E+06	4.83E+06	4.55E+06	4.89E+06				22.35	23.09	23.24	22.2	22.33	22.48				23.09	22.33		0.476737	0.140696	
1413	P06753-5	Tropomyo	TPM3	13	42.7	2.85E+07	5.13E+07	8.62E+07	3.25E+07	2.36E+07	4.16E+07	7.39E+07	3.40E+07	4.75E+07	24.71	25.81	26.67	25.05	24.62	25.56	26.08	24.85	25.03	25.81	25.05	25.03	0.986053	0.473934	0.665249
1549	P18077	60S riboso	RPL35A	9	47.3	2.07E+08	1.76E+08	7.95E+07	1.31E+08	1.18E+08	9.61E+07	1.17E+08	1.86E+08	1.55E+08	27.77	27.81	26.6	27.15	27	26.77	26.81	27.26	26.5	27.77	27	26.81	0.688198	0.189077	0.384364
1309	O95071-2	E3 ubiquiti	UBR5	16	8.9	1.30E+07	1.74E+07	1.70E+07	9.86E+06	1.04E+07	1.28E+07	1.02E+07	1.03E+07	3.44E+06	23.6	24.24	24.25	23.25	23.47	23.82	23.22	23.07	21.11	24.24	23.47	23.07	0.372791	0.289342	1.180538
2192	Q13724-2	Mannosyl-	MOGS	4	7		5.25E+06	7.81E+06		3.18E+06	4.45E+06	4.18E+06				22.53	23.18		21.81	22.36	21.97			22.85	22.08	21.97	0.458156	0.38788	
2701	Q96A72	Protein ma	MAGOHB	6	48	3.43E+07	4.07E+07	3.89E+07	3.00E+07	2.48E+07	2.12E+07	2.79E+07	2.74E+07		25.02	25.48	25.48	24.86	24.71	24.55	24.72	24.51		25.48	24.71	24.61	0.26578	0.152137	0.145072
2856	Q9BUP3	Oxidoredu	HTATIP2	5	22.3	2.07E+06	4.29E+06	4.87E+06	2.54E+06	2.60E+06	3.58E+06	2.74E+06			20.91	22.3	22.52	21.33	21.52	22.06	21.31			22.3	21.52	21.31	0.875987	0.377915	
1483	P11216	Glycogen p	PYGB	9	17.7	6.63E+06	8.77E+06	6.01E+06	3.39E+06	3.80E+06	5.10E+06	7.54E+06			22.59	23.23	22.83	21.7	22.05	22.55	22.77			22.83	22.05	22.77	0.324494	0.426973	
1451	P09382	Galectin-1	LGALS1	9	74.8	1.47E+08	1.77E+08	1.69E+08	1.21E+08	1.13E+08	1.64E+08	1.84E+08	1.78E+08	1.40E+08	27.19	27.82	27.76	26.98	26.95	27.73	27.48	27.21	26.39	27.76	26.98	27.21	0.350204	0.442792	0.566473
1006	J3KQ06	Transmem	TMEM45A	2	6.5	8.14E+06		5.28E+06		2.71E+06	4.60E+06	2.86E+06			22.91		22.66		21.61	22.4	21.4			22.79	22.01	21.4	0.183503	0.556181	
2313	Q16531	DNA dama	DDB1	30	31.1	6.85E+07	7.92E+07	7.26E+07	4.72E+07	4.82E+07	6.34E+07	5.27E+07	4.13E+07	1.15E+07	25.98	26.48	26.48	25.62	25.7	26.17	25.61	25.09	23.04	26.48	25.7	25.09	0.289876	0.29569	1.360178
3264	Q9Y6H1	Coiled-coil	CHCHD2	4	33.8	3.31E+06	7.54E+06	3.96E+06		1.70E+06	3.27E+06	3.54E+06			21.63	23.03	22.24		20.97	21.94	21.69			22.24	21.46	21.69	0.701088	0.68655	
1175	O15397	Importin-8	IPO8	51	48.5	3.27E+09	3.81E+09	3.57E+09	4.46E+09	4.65E+09	4.78E+09	8.13E+09	1.20E+10	4.83E+09	32.19	32.8	32.9	32.02	31.99	32.79	32.85	33.2	30.73	32.8	32.02	32.85	0.384515	0.457324	1.337713
2168	Q13347	Eukaryotic	EIF3I	16	67.4	9.48E+07	1.58E+08	1.37E+08	9.67E+07	7.49E+07	9.30E+07	1.23E+08	1.05E+08	1.96E+07	26.59	27.64	27.44	26.65	26.35	26.71	26.88	26.41	23.81	27.44	26.65	26.41	0.55383	0.191045	1.651581
69	A0A087W\	Kanadaptir	SLC4A1AP	2	6.2			1.44E+06		8.14E+05	8.21E+05						20.8		19.9	20.14				20.8	20.02			0.166727	
2269	Q15181	Inorganic p	PPA1	12	49.8	4.65E+07	5.42E+07	5.45E+07	3.38E+07	2.76E+07	4.47E+07	3.80E+07	4.15E+07	3.04E+07	25.46	25.91	25.98	25.12	24.9	25.67	25.18	25.1	24.38	25.91	25.12	25.1	0.284362	0.395797	0.441581
1584	P22234	Multifuncti	PAICS	19	40.2	9.32E+07	1.55E+08	1.53E+08	9.04E+07	9.70E+07	1.33E+08	1.25E+08	1.36E+08	8.58E+07	26.58	27.6	27.56	26.59	26.77	27.41	26.93	26.79	25.85	27.56	26.77	26.79	0.577853	0.430964	0.583302
1648	P29692	Elongation	EEF1D	10	46.3	5.69E+08	6.03E+08	5.38E+08	4.35E+08	3.67E+08	4.89E+08	5.27E+08	6.95E+08	3.19E+08	29.11	29.48	29.51	28.69	28.56	29.24	28.99	28.96	27.41	29.48	28.69	28.96	0.226314	0.36525	0.900459
352	B0S8I6	Protein FA	FAM50A	4	19.2		4.91E+06	4.91E+06			2.74E+06					22.46	22.54			21.71				22.5	21.71		0.059325		
1710	P35573	Glycogen c	AGL	8	7	2.05E+06	2.96E+06	3.22E+06	1.94E+06	1.69E+06	2.02E+06		1.61E+06		20.89	21.75	21.92	20.91	20.95	21.28		20.38		21.75	20.95	20.38	0.553149	0.19912	
1187	O43252	Bifunctiona	PAPSS1	6	11.7	2.61E+06	5.39E+06	6.83E+06	3.03E+06	3.09E+06	3.51E+06	3.64E+06	2.95E+06		21.28	22.57	22.97	21.55	21.78	22.04	21.73	21.34		22.57	21.78	21.54	0.885522	0.244522	0.271901
2705	Q96AG4	Leucine-ric	LRRC59	7	31.6	3.21E+07	5.26E+07	4.71E+07	1.81E+07	2.92E+07	3.83E+07	2.57E+07	2.67E+07	1.32E+07	24.95	25.87	25.76	24.17	24.97	25.44	24.63	24.48	23.26	25.76	24.97	24.48	0.502209	0.643986	0.755542
306	A6NDG6	Phosphogl	PGP	5	19.3	5.72E+06	7.42E+06	7.00E+06	3.59E+06	4.27E+06	5.49E+06	4.54E+06	4.21E+06	3.46E+06	22.37	23.01	23	21.76	22.21	22.65	22.08	21.81	21.12	23	22.21	21.81	0.370812	0.447571	0.49915
354	B1AHD1	NHP2-like	NHP2L1	2	18.2	4.45E+06	4.57E+06	2.22E+06	2.39E+06	2.08E+06	2.95E+06				22.03	22.38	21.42	21.23	21.21	21.8				22.03	21.23		0.487497	0.335462	
864	Q2L6G8	Corneodes	CDSN	3	9.6	2.54E+06		1.56E+07	3.84E+06				2.68E+06	5.68E+06	21.24		24.12	21.88				21.2	21.88	22.68	21.88	21.54	2.03623		0.486219
2714	Q96C19	EF-hand dc	EFHD2	4	21.7		4.65E+06	4.56E+06	3.14E+06							22.39	22.43	21.61						22.41	21.61		0.030108		
1736	P37802	Transgelin	TAGLN2	14	75.9	1.56E+08	2.52E+08	2.46E+08	1.29E+08	1.73E+08	2.01E+08	1.81E+08	1.56E+08	8.66E+07	27.27	28.32	28.48	27.11	27.52	28.03	27.46	26.99	25.88	28.32	27.52	26.99	0.661622	0.458138	0.809302
1731	P36915	Guanine nu	GNL1	4	7.6	3.70E+06	4.03E+06	5.09E+06	2.76E+06	1.96E+06	3.74E+06	2.50E+06	2.00E+06		21.78	22.23	22.58	21.42	21.12	22.13	21.16	20.78		22.23	21.42	20.97	0.39957	0.517163	0.271731
1179	O15511	Actin-relat	ARPC5	2	21.9	9.04E+06	1.45E+07	5.42E+06		4.40E+06		7.75E+06	7.75E+06		23.07	23.95	22.7		22.26		22.83	22.66		23.07	22.26	22.75	0.642151		0.12008
1919	P55060-3	Exportin-2	CSE1L	29	33.7	8.36E+07	1.70E+08	1.25E+08	7.75E+07	8.09E+07	9.59E+07	7.94E+07	6.44E+07	2.12E+07	26.33	27.74	27.26	26.32	26.45	26.77	26.2	25.76	23.94	27.26	26.45	25.76	0.716728	0.231459	1.197306
894	H0YEN5	40S riboso	RPS2	13	63.6	1.26E+08	1.25E+08	9.19E+07	5.63E+07	6.37E+07	8.58E+07	5.03E+07	6.19E+07	1.89E+07	26.95	27.26	26.82	25.85	26.14	26.61	25.57	25.69	23.76	26.95	26.14	25.57	0.225495	0.379325	1.081883
1541	P17174	Aspartate	GOT1	10	33.4	1.27E+07	1.74E+07	1.61E+07	7.02E+06	9.63E+06	1.41E+07	1.05E+07	1.04E+07	1.18E+07	23.57	24.24	24.16	22.77	23.35	23.96	23.25	23.08	23.08	24.16	23.35	23.08	0.364586	0.593419	0.097814
1070	M0QXF7	Myeloid-de	MYDGF	2	32.1	5.80E+06	9.07E+06	8.00E+06	5.63E+06	3.61E+06	4.63E+06	5.15E+06			22.39	23.28	23.22	22.44	21.97	22.4	22.25			23.22	22.4	22.25	0.498815	0.258883	
895	H0YFA4	Cysteine-ri	CRIP2	2	26.6		4.16E+06	4.79E+06	2.57E+06	2.66E+06	2.50E+06					22.27	22.51	21.34	21.58	21.57				22.39	21.57		0.170126	0.133581	
2287	Q15459	Splicing fac	SF3A1	14	21.9	1.96E+07	2.38E+07	3.28E+07	1.41E+07	1.34E+07	1.36E+07	6.50E+06	4.44E+06		24.19	24.65	25.14	23.78	23.83	23.91	22.6	21.87		24.65	23.83	22.24	0.471174	0.066488	0.520533
1154	O15067	Phosphoril	PFAS	15	17	1.77E+07	2.24E+07	2.47E+07	1.10E+07	1.27E+07	1.72E+07	1.42E+07	1.10E+07	4.86E+06	24.05	24.58	24.79	23.38	23.75	24.28	23.72	23.15	21.62	24.58	23.75	23.15	0.384285	0.454905	1.083891
1513	P13797	Plastin-3	PLS3	19	41.1	3.64E+07	4.48E+07	5.08E+07	2.80E+07	2.26E+07	3.83E+07	2.79E+07	2.03E+07	2.35E+07	25.08	25.62	25.9	24.8	24.57	25.44	24.72	24.11	24.04	25.62	24.8	24.11	0.418145	0.453726	0.371769
1416	P07099	Epoxide hy	EPHX1	4	9			2.53E+06			1.43E+06	1.28E+06					21.57			20.75	20.13			21.57	20.75	20.13			
1492	P11717	Cation-ind	IGF2R	15	6.9	1.77E+07	2.47E+07	2.32E+07	1.38E+07	1.38E+07	1.49E+07	1.45E+07	1.15E+07		24.05	24.71	24.7	23.74	23.87	24.05	23.76	23.22		24.7	23.87	23.49	0.380571	0.155248	0.376859
3059	Q9NYU2-2	UDP-gluco	UGGT1	13	11.8	1.08E+07	1.28E+07	1.27E+07	8.00E+06	7.21E+06	9.01E+06	1.14E+07	5.13E+06		23.34	23.8	23.85	22.93	22.97	23.34	23.35	22.05		23.8	22.97	22.7	0.281194	0.22788	0.918667
1823	P49321	Nuclear au	NASP	14	27.5	2.51E+07	4.28E+07	4.48E+07	2.52E+07	2.51E+07	3.24E+07	4.40E+07	1.84E+07	1.52E+07	24.53	25.57	25.66	24.65	24.73	25.23	25.34	23.94	23.46	25.57	24.73	23.94	0.625843	0.314214	0.97351
1043	K7EK06	Phenylalan	FARSA	3	32	9.41E+06	3.24E+06	7.70E+06	5.08E+06	4.49E+06	3.92E+06	5.16E+06			23.13	21.89	23.16	22.29	22.3	22.19	22.25			23.13	22.29	22.25	0.725747	0.062127	
1613	P25398	40S riboso	RPS12	7	65.9	1.08E+08	1.06E+08	1.03E+08	4.08E+07	7.49E+07	6.10E+07	8.81E+07	6.43E+07		26.8	26.92	26.99	25.39	26.35	26.09	26.38	25.75		26.92	26.09	26.07	0.099327	0.500767	0.44194
3105	U3KQ56	Glyoxylate	GRHPR	3	14.5	4.80E+06	1.12E+07	9.51E+06	4.17E+06		8.35E+06	5.74E+06	5.40E+06		22.12	23.61	23.46	22		23.25	22.42	22.12		23.46	22.62	22.27	0.821003	0.883399	0.21392

3191	Q9Y2A7	Nck-associ	NCKAP1	8	8.7	4.89E+06	7.28E+06	6.86E+06	3.86E+06	4.09E+06	5.47E+06	4.97E+06	3.77E+06		22.15	22.99	22.98	21.88	22.14	22.65	22.2	21.66		22.98	22.14	21.93	0.481791	0.39	0.382397
1474	P10809	60 kDa hea	HSPD1	41	77.1	1.06E+09	1.13E+09	1.37E+09	7.88E+08	8.74E+08	1.21E+09	8.26E+08	8.36E+08	3.19E+08	30.39	30.73	31.22	29.59	29.89	30.55	29.5	29.37	27.41	30.73	29.89	29.37	0.420623	0.49406	1.167274
1749	P40227	T-complex	CCT6A	26	52.7	7.41E+08	7.65E+08	7.21E+08	5.46E+08	4.69E+08	5.20E+08	5.54E+08	5.31E+08	3.67E+08	29.5	30.02	29.93	29.09	28.98	29.32	29.01	28.56	27.68	29.93	29.09	28.56	0.281803	0.176098	0.678371
1744	P39023	60S riboso	RPL3	17	40.7	1.43E+08	2.28E+08	1.85E+08	9.83E+07	1.24E+08	1.37E+08	1.95E+08	1.10E+08	7.44E+07	27.17	28.19	27.92	26.68	27.08	27.47	27.56	26.46	25.62	27.92	27.08	26.46	0.532204	0.39419	0.971813
2232	Q14684-2	Ribosomal	RRP1B	4	6.5			7.78E+06	5.21E+06	5.17E+06	2.18E+06						23.17	22.33	22.51	21.39				23.17	22.33			0.604186	
2134	Q12792	Twinfilin-1	TWF1	10	32.6	1.71E+07	2.73E+07	2.58E+07	1.48E+07	1.51E+07	2.16E+07	1.12E+07	9.51E+06	7.84E+06	23.99	24.87	24.84	23.85	24	24.58	23.32	22.96	22.43	24.84	24	22.96	0.498184	0.383424	0.449585
2505	Q86U42-2	Polyadenyl	PABPN1	2	9.1	5.95E+06	7.53E+06	7.15E+06	4.10E+06	4.21E+06	6.58E+06	4.11E+06			22.43	23.03	23.04	21.97	22.18	22.9	21.94			23.03	22.18	21.94	0.346433	0.488179	
847	G3XAI2	Laminin su	LAMB1	11	7.7	8.60E+06	8.97E+06	7.88E+06	4.90E+06	4.62E+06	6.22E+06	5.06E+06	5.00E+06	3.40E+06	22.99	23.27	23.19	22.22	22.35	22.83	22.23	22.03	21.08	23.19	22.35	22.03	0.144026	0.320249	0.611189
1864	P50990	T-complex	CCT8	26	51.5	2.74E+08	1.73E+08	2.04E+08	1.37E+08	8.94E+07	1.27E+08	1.05E+08	8.07E+07	1.88E+08	28.08	27.76	28.14	27.23	26.63	27.32	26.68	26.06	26.77	28.08	27.23	26.68	0.199837	0.373802	0.384968
1394	P05023-3	Sodium/pc	ATP1A1	21	27	4.99E+07	4.77E+07	3.72E+07	2.43E+07	3.22E+07	2.37E+07	4.13E+07	1.92E+07	3.23E+07	25.55	25.66	25.41	24.59	25.1	24.71	25.28	24.01	24.5	25.55	24.71	24.5	0.126353	0.26748	0.641678
2290	Q15582	Transformi	TGFBI	7	10.1	9.08E+06	9.47E+06	1.04E+07	5.45E+06	5.17E+06	8.94E+06	5.49E+06	4.97E+06	4.57E+06	23.08	23.36	23.57	22.38	22.51	23.33	22.36	22.02	21.53	23.36	22.51	22.02	0.249158	0.511904	0.41851
1627	P26640	Valine--tRN	VARS	32	31.4	1.64E+08	2.15E+08	1.91E+08	8.61E+07	1.33E+08	1.47E+08	8.62E+07	8.67E+07	2.90E+07	27.38	28.11	27.98	26.51	27.13	27.59	26.35	26.2	24.31	27.98	27.13	26.2	0.393111	0.543458	1.134975
317	A6NJA2	Ubiquitin c	USP14	9	25.7	1.53E+07	1.71E+07	2.14E+07	1.09E+07	9.03E+06	1.29E+07	9.19E+06	7.60E+06	4.54E+06	23.81	24.22	24.59	23.36	23.25	23.84	23.08	22.64	21.5	24.22	23.36	22.64	0.387305	0.31061	0.816436
2483	Q7L2H7	Eukaryotic	EIF3M	16	52.1	1.32E+08	1.07E+08	9.99E+07	6.71E+07	6.90E+07	5.51E+07	7.60E+07	9.35E+07	4.76E+07	27	26.95	26.94	26.09	26.21	25.92	26.1	26.28	25.04	26.95	26.09	26.1	0.033056	0.145131	0.667444
1826	P49368	T-complex	CCT3	32	58	6.05E+08	7.32E+08	6.85E+08	5.16E+08	4.82E+08	5.90E+08	4.34E+08	5.16E+08	1.24E+08	29.25	29.92	29.91	28.91	29.06	29.4	28.63	28.47	26.26	29.91	29.06	28.47	0.384901	0.254842	1.324701
1587	Q32Q12	Nucleoside	NME1-NM	18	73.3	5.37E+08	6.85E+08	5.61E+08	4.04E+08	4.17E+08	6.01E+08	3.12E+08	4.08E+08	3.22E+08	29	29.69	29.63	28.61	28.78	29.47	28.11	28.19	27.44	29.63	28.78	28.11	0.38241	0.455675	0.41393
699	E9PHS0	LanC-like p	LANCL1	4	22.4	3.00E+07	2.15E+07	2.21E+07	2.44E+07	9.58E+06	1.24E+07	2.25E+07	2.16E+07		24.83	24.51	24.64	24.6	23.35	23.78	24.43	24.18		24.64	23.78	24.3	0.159638	0.636615	0.180281
627	E7EM64	COP9 signa	COPS6	6	26.1	8.15E+06	1.44E+07	1.34E+07	8.75E+06	7.49E+06	1.03E+07	7.93E+06	8.81E+06	5.08E+06	22.92	23.94	23.92	23.06	23.03	23.52	22.86	22.85	21.72	23.92	23.06	22.85	0.581997	0.276053	0.654566
1116	O00469	Procollage	PLOD2	8	11	1.92E+07	1.61E+07	1.78E+07	9.46E+06	1.32E+07	8.81E+06	1.63E+07	1.49E+07	8.62E+06	24.17	24.1	24.33	23.18	23.82	23.31	23.93	23.63	22.55	24.17	23.31	23.63	0.115954	0.334984	0.723818
425	F8WAU4	Elongation	GFM1	5	11.3	4.58E+06	7.78E+06	8.93E+06	4.41E+06	4.27E+06	6.01E+06	4.19E+06	4.37E+06		22.06	23.07	23.35	22.08	22.21	22.78	21.97	21.84		23.07	22.21	21.91	0.677544	0.373273	0.090501
409	B7Z7P8	Eukaryotic	ETF1	10	24.3	2.85E+07	3.51E+07	3.70E+07	1.46E+07	1.98E+07	2.92E+07	1.91E+07	1.71E+07	9.93E+06	24.71	25.26	25.4	23.83	24.4	25.04	24.18	23.81	22.78	25.26	24.4	23.81	0.369749	0.604323	0.725968
1895	P52888	Thimet olig	THOP1	9	14.7	1.14E+07	1.85E+07	2.16E+07	1.14E+07	1.01E+07	1.75E+07	1.66E+07	1.71E+07	1.08E+07	23.42	24.3	24.6	23.43	23.43	24.3	23.95	23.81	22.94	24.3	23.43	23.81	0.610225	0.499337	0.544952
1374	P02786	Transferrin	TFRC	22	35	5.09E+07	5.99E+07	7.66E+07	2.89E+07	3.45E+07	5.75E+07	3.50E+07	1.94E+07	1.02E+07	25.58	26.07	26.54	24.83	25.2	25.98	25.06	24.02	22.83	26.07	25.2	24.02	0.48352	0.586709	1.117268
2927	Q9H2J4	Phosducin-	PDCL3	6	27.6	5.76E+06	7.65E+06	7.40E+06	4.77E+06	3.24E+06	4.49E+06				22.38	23.05	23.1	22.18	21.84	22.37				23.05	22.18		0.406198	0.269862	
3004	Q9NQR4	Omega-am	NIT2	5	23.6	3.39E+06	4.80E+06	4.13E+06	2.76E+06	2.19E+06	3.24E+06	2.45E+06	2.35E+06		21.66	22.44	22.29	21.42	21.28	21.92	21.14	21.01		22.29	21.42	21.08	0.415946	0.339652	0.093413
1762	P41250	Glycine--tR	GARS	14	24.6	2.38E+07	3.80E+07	4.07E+07	2.08E+07	2.11E+07	2.78E+07	2.43E+07	2.47E+07	9.15E+06	24.47	25.37	25.54	24.37	24.49	24.97	24.53	24.39	22.64	25.37	24.49	24.39	0.575034	0.318848	1.052164
1832	P49588	Alanine--tR	AARS	21	31.2	2.93E+07	3.33E+07	5.03E+07	1.98E+07	1.80E+07	1.97E+07	2.07E+07	1.79E+07	4.24E+06	24.78	25.17	25.89	24.29	24.26	24.47	24.32	23.88	21.35	25.17	24.29	23.88	0.562834	0.110382	1.599833
2195	Q13838	Spliceosom	DDX39B	4	43.7	8.85E+06	1.36E+07	1.32E+07	4.03E+06	7.29E+06	1.21E+07				23.04	23.87	23.91	21.95	22.99	23.71				23.87	22.99		0.491326	0.886208	
2709	Q96BD8	Spindle an	SKA1	4	21.2	4.45E+07	3.37E+07	2.10E+07	1.99E+07	1.87E+07	2.94E+07	5.82E+06	3.72E+07		25.41	25.19	24.56	24.3	24.32	25.06	22.44	24.96		25.19	24.32	23.7	0.440097	0.435891	1.783698
3057	Q9NYL9	Tropomod	TMOD3	10	39.8	2.40E+07	3.83E+07	3.18E+07	1.71E+07	1.72E+07	2.14E+07	2.82E+07	2.62E+07	8.37E+06	24.48	25.4	25.1	24.07	24.21	24.56	24.75	24.47	22.52	25.1	24.21	24.47	0.466799	0.252927	1.214271
80	A0A087W	3-hydroxyi	HIBCH	5	13.4	2.38E+06	3.28E+06	3.56E+06		1.61E+06	1.90E+06	2.18E+06	1.85E+06		21.12	21.91	22.08		20.86	21.19	20.93	20.65		21.91	21.02	20.79	0.512753	0.230929	0.201237
2098	Q06203	Amidopho	PPAT	6	13.2	5.59E+06	7.06E+06	7.99E+06	4.19E+06	3.81E+06	6.49E+06	3.68E+06	3.14E+06		22.32	22.95	23.22	22.01	22.06	22.88	21.75	21.43		22.95	22.06	21.59	0.456992	0.488182	0.23061
1260	O75369-2	Filamin-B	FLNB	72	44.1	1.49E+08	1.83E+08	1.78E+08	1.01E+08	1.11E+08	1.26E+08	9.75E+07	8.71E+07	3.23E+07	27.22	27.91	27.83	26.71	26.94	27.3	26.55	26.22	24.5	27.83	26.94	26.22	0.378915	0.295473	1.099175
1138	O14818	Proteasom	PSMA7	12	51.2	8.67E+07	1.09E+08	7.27E+07	4.55E+07	4.05E+07	6.93E+07	2.52E+07	3.43E+07	6.20E+06	26.41	26.99	26.48	25.59	25.43	26.29	24.59	24.87	22.06	26.48	25.59	24.59	0.313561	0.459067	1.546107
2484	Q7L576	Cytoplasmi	CYFIP1	16	14.8	1.58E+07	2.06E+07	2.21E+07	9.77E+06	1.11E+07	1.85E+07	1.27E+07	1.19E+07	2.45E+06	23.86	24.43	24.64	23.23	23.54	24.41	23.52	23.31	20.64	24.43	23.54	23.31	0.402013	0.613437	1.606168
676	E9PC90	G2/mitotic	CCNB1	3	8		7.20E+06	1.09E+07			4.66E+06					22.98	23.64			22.41				23.31	22.41		0.471204		
1420	P07355	Annexin A2	ANXA2	24	64	9.08E+07	1.05E+08	1.30E+08	6.35E+07	5.22E+07	8.20E+07	5.42E+07	5.23E+07	7.65E+07	26.51	26.9	27.35	26.01	25.82	26.56	25.67	25.46	25.66	26.9	26.01	25.66	0.417421	0.38491	0.115644
475	C9JRD2	DnaJ homo	DNAJB2	5	24.6		4.06E+06	6.03E+06		2.77E+06						22.24	22.83		21.64					22.53	21.64		0.416139		
3002	Q9NQC3-2	Reticulon-4	RTN4	6	28.7	1.59E+07	2.85E+07	2.58E+07	1.55E+07	1.46E+07	2.20E+07	1.88E+07	2.12E+07		23.87	24.92	24.84	23.91	23.94	24.6	24.15	24.16		24.84	23.94	24.15	0.582781	0.388525	0.008744
1945	P61019	Ras-relatec	RAB2A	6	34.9	9.62E+06	8.05E+06	3.76E+06	3.95E+06	6.11E+06	4.00E+06	3.86E+06	4.12E+06		23.17	23.11	22.18	21.92	22.75	22.21	21.83	21.77		23.11	22.21	21.8	0.555451	0.420158	0.038386
2261	Q15056-2	Eukaryotic	EIF4H	7	32.9	2.33E+07	3.37E+07	3.68E+07	1.97E+07	1.85E+07	2.56E+07	1.68E+07	1.51E+07		24.42	25.19	25.38	24.28	24.29	24.79	23.97	23.64		25.19	24.29	23.81	0.508205	0.288247	0.227349
1193	O43396	Thioredoxi	TXNL1	6	22.8	6.83E+06	1.01E+07	9.54E+06	5.98E+06	4.34E+06	7.38E+06	4.27E+06	5.92E+06		22.64	23.45	23.46	22.55	22.23	23.08	22	22.26		23.45	22.55	22.13	0.474112	0.426509	0.188626
1712	P35606-2	Coatomer	COPB2	16	26.1	1.83E+07	3.43E+07	2.09E+07	1.20E+07	1.19E+07	1.67E+07	1.94E+07	1.06E+07		24.11	25.23	24.55	23.52	23.64	24.23	24.2	23.1		24.55	23.64	23.65	0.559832	0.380063	0.777071
1493	P11766	Alcohol de	ADH5	8	25.7	1.25E+07	1.54E+07	2.11E+07	5.97E+06	8.27E+06	1.20E+07	9.40E+06	5.72E+06		23.55	24.04	24.57	22.54	23.13	23.69	23.11	22.22		24.04	23.13	22.66	0.509266	0.577385	0.626042
2141	Q12906-4	Interleukin	ILF3	13	28.2	1.07E+07	3.23E+07	3.57E+07	1.30E+07	1.69E+07	2.28E+07	1.70E+07	7.52E+06	4.78E+06	23.33	25.09	25.32	23.66	24.19	24.65	24	22.63	21.6	25.09	24.19	22.63	1.093031	0.496386	1.205101
1203	O43776	Asparagine	NARS	13	23.4	1.17E+07	1.88E+07	2.04E+07	1.12E+07	9.07E+06	1.47E+07	7.63E+06	8.49E+06	2.85E+06	23.46	24.32	24.51	23.41	23.26	24.03	22.79	22.8	20.88	24.32	23.41	22.79	0.55663	0.411606	1.102585
2110	Q07960	Rho GTPas	ARHGAP1	5	15.3	6.28E+06	8.65E+06	9.14E+06	3.59E+06	4.50E+06	7.45E+06	5.91E+06	4.77E+06		22.51	23.22	23.39	21.76	22.31	23.09	22.47	21.97		23.22	22.31	22.22	0.466816	0.667869	0.349078
2475	Q70UQ0-4	Inhibitor of	IKBIP	7	22.5	9.83E+06	1.06E+07	9.00E+06	5.69E+06	4.71E+06	8.22E+06	7.53E+06	9.88E+06		23.2	23.52	23.37	22.46	22.37	23.22	22.76	23.01		23.37	22.46	22.89	0.159845	0.468785	0.176893
1878	P51665	26S protea	PSMD7	9	32.4	2.33E+07	5.47E+07	4.83E+07	3.08E+07	3.27E+07	2.46E+07	2.99E+07	3.70E+07	7.87E+06	24.42	25.95	25.83	24.92	25.12	24.74	24.82	24.95	22.43	25.83	24.92	24.82	0.846677	0.193439	1.418616
806	F8VZQ9	SAP domai	SARNP	5	22.1		9.95E+06	7.20E+06	5.23E+06	3.69E+06	6.16E+06	6.69E+06	4.31E+06			23.43	23.06	22.33	22.01	22.82	22.64	21.83		23.24	22.33	22.24	0.258594	0.407648	0.570351
831	G3V1D3	Dipeptidyl	DPP3	7	14.9	7.00E+06	9.82E+06	1.11E+07	5.17E+06	5.12E+06	6.23E+06	5.88E+06	5.34E+06		22.67	23.41	23.68	22.31	22.49	22.83	22.46	22.1		23.41	22.49	22.28	0.521698	0.265266	0.258726
621	E5RI99	60S riboso	RPL30	7	58.8	1.69E+08	1.31E+08	7.83E+07	8.19E+07	7.20E+07	8.31E+07	8.17E+07	8.52E+07	6.74E+07	27.44	27.33	26.56	26.42	26.27	26.57	26.23	26.16	25.46	27.33	26.42	26.16	0.478126	0.151574	0.427099
1988	P62424	60S riboso	RPL7A	16	44.7	3.04E+08	3.30E+08	2.73E+08	2.06E+08	2.02E+08	2.41E+08	2.04E+08	1.85E+08	7.97E+07	28.17	28.76	28.65	27.69	27.73	28.29	27.66	27.25	25.71	28.65	27.73	27.25	0.311072	0.331175	1.028007
672	H0YHX9	Nascent pc	NACA	7	40.8	4.92E+07	7.37E+07	6.91E+07	3.78E+07	4.14E+07	5.67E+07	3.97E+07	3.39E+07	3.46E+07	25.52	26.38	26.39	25.28	25.46	25.96	25.24	24.83	24.62	26.38	25.46	24.83	0.498478	0.354056	0.314704
3228	Q9Y490	Talin-1	TLN1	54	32.1	8.86E+07	1.22E+08	1.16E+08	6.65E+07	6.49E+07	8.74E+07	1.07E+08	1.11E+08	1.61E+07	26.48	27.24	27.1	26.07	26.17	26.65	26.71	26.47	23.57	27.1	26.17	26.47	0.404657	0.306153	1.748112
1444	P08708	40S riboso	RPS17	8	60	2.11E+07	5.36E+07	4.79E+07	3.05E+07	2.74E+07	5.37E+07	3.57E+07	2.98E+07		24.28	25.89	25.82	24.89	24.89	25.91	25.11	24.66		25.82	24.89	24.88	0.908261	0.585668	0.315273
1547	P17987	T-complex	TCP1	29	57.6	8.01E+08	9.06E+08	8.45E+08	7.43E+08	6.98E+08	7.03E+08	7.13E+08	7.88E+08	3.66E+08	29.64	30.48	30.43	29.45	29.5	29.67	29.27	29.3	27.66	30.43	29.5	29.27	0.466698	0.117011	0.938616
1834	Q5T5C7	Serine--tRN	SARS	4	14	3.66E+06	6.87E+06	8.72E+06	3.61E+06	3.59E+06	5.13E+06	3.48E+06	3.09E+06		21.76	22.89	23.32	21.77	21.97	22.56	21.67	21.4		22.89	21.97	21.53	0.805968	0.410747	0.186066
2193	Q13813	Spectrin al	SPTAN1	164	64.3	1.84E+09	2.10E+09	1.95E+09	2.10E+09	1.81E+09	1.92E+09	1.37E+09	1.62E+09	8.69E+08	31.44	32.08	32.16	31.15	31.03	31.63	30.61	30.64	29.02	32.08	31.15	30.61	0.392296	0.315186	0.925437
1504	P12956	X-ray repai	XRCC6	28	52.7	1.12E+08	1.55E+08	1.80E+08	5.84E+07	9.13E+07	1.47E+08	1.17E+08	9.18E+07	6.77E+06	26.83	27.6	27.87	25.89	26.66	27.59	26.81	26.26	22.18	27.6	26.66	26.26	0.537525	0.850388	2.531198
2126	Q10567-4	AP-1 comp	AP1B1	3	18.2	2.49E+06	3.73E+06	2.98E+06	1.90E+06	2.18E+06	1.53E+06				21.21	22.11	21.82	20.88	21.27	20.83				21.82	20.88		0.456874	0.240629	
2683	Q92973-2	Transporti	TNPO1	12	18.5	1.92E+07	3.95E+07	3.43E+07	1.09E+07	1.84E+07	1.83E+07	2.46E+07	1.15E+07	6.87E+06	24.17	25.43	25.22	23.36	24.29	24.39	24.55	23.22	22.21	25.22	24.29	23.22	0.677299	0.565326	1.171452
1481	P11177-2	Pyruvate d	PDHB	10	38.1	1.66E+08	1.81E+08	1.89E+08	1.20E+08	8.45E+07	1.04E+08	1.01E+08	1.58E+08	9.65E+07	27.42	27.88	27.96	26.95	26.54	26.94	26.59	27.02	26.01	27.88	26.94	26.59	0.291982	0.236054	0.504787
1033	J3QT28	Mitotic che	BUB3	9	38.1	2.73E+07	3.72E+07	3.43E+07	2.74E+07	1.35E+07	1.72E+07	2.11E+07	2.54E+07	1.32E+07	24.64	25.34	25.22	24.76	23.84	24.28	24.34	24.42	23.26	25.22	24.28	24.34	0.373058	0.462727	0.649382
1051	K7ENJ0	UV excisio	RAD23A	4	20.9	3.77E+06	5.97E+06	6.91E+06			2.79E+06	2.24E+06	2.52E+06		21.82	22.68	22.99			21.73	21.01	21.08		22.68	21.73	21.05	0.606891		0.049378
2297	Q15691	Microtubu	MAPRE1	9	47.8	1.23E+07	2.88E+07	3.43E+07	1.57E+07	1.55E+07	1.44E+07	1.27E+07	1.14E+07	2.15E+06	23.52	24.94	25.22	23.94	24.03	24	23.52	23.21	20.36	24.94	24	23.21	0.911784	0.045255	1.740694
973	I3L0A0	Ubiquitin-c	TMEM189-	7	14.1	1.21E+07	2.20E+07	2.11E+07	1.27E+07	7.43E+06	1.90E+07	1.44E+07	1.21E+07	1.14E+07	23.5	24.55	24.57	23.61	23.01	24.43	23.74	23.34	23.03	24.55	23.61	23.34	0.611425	0.712189	0.358469
1001	J3QL05	Serine/argi	SRSF2	4	36.9	4.16E+07	3.37E+07	3.76E+07	2.05E+07	9.35E+06	1.97E+07	1.32E+07		8.99E+06	25.29	25.19	25.42	24.34	23.31	24.47	23.57		22.62	25.29	24.34	23.09	0.115355	0.635373	0.678245
2522	Q86XR7-2	TIR domair	TICAM2	3	7.7		3.35E+06	6.56E+06	2.43E+06	2.76E+06	2.36E+06					21.94	22.92	21.26	21.63	21.48				22.43	21.48		0.688323	0.184669	
1814	P48643	T-complex	CCT5	44	71.9	1.03E+09	1.21E+09	1.27E+09	8.67E+08	9.58E+08	1.14E+09	7.11E+08	8.35E+08	1.69E+08	30.33	30.96	31.03	29.8	30.01	30.43	29.25	29.33	26.6	30.96	30.01	29.25	0.3894	0.323794	1.555993
2146	Q13011	Delta(3,5)-	ECH1	8	30.8	2.25E+07	3.13E+07	2.91E+07	1.68E+07	1.35E+07	2.03E+07	1.62E+07	1.54E+07	1.41E+07	24.36	25.04	25.01	24.05	23.84	24.5	23.91	23.67	23.36	25.01	24.05	23.67	0.383393	0.338859	0.275434
1683	P31930	Cytochrom	UQCRC1	9	27.9	1.68E+07	2.40E+07	2.04E+07	9.42E+06	1.12E+07	1.87E+07	1.11E+07	9.51E+06	5.04E+06	23.96	24.66	24.51	23.18	23.55	24.42	23.31	22.96	21.7	24.51	23.55	22.96	0.366216	0.635842	0.843944
2165	Q13263	Transcripti	TRIM28	20	33.1	4.31E+07	8.37E+07	6.77E+07	3.37E+07	4.01E+07	4.80E+07	3.43E+07	2.96E+07	1.02E+07	25.37	26.55	26.36	25.12	25.4	25.75	25.02	24.65	22.83	26.36	25.4	24.65	0.634545	0.315596	1.172118
3093	Q9P2R7-2	Succinyl-Cc	SUCLA2	2	5	1.83E+06	3.50E+06		1.40E+06			1.57E+06	1.70E+06		20.74	22.01		20.42			20.54	20.48		21.38	20.42	20.51	0.897985		0.043733
1702	P34932	Heat shock	HSPA4	32	41.5	8.72E+07	1.42E+08	1.31E+08	6.59E+07	7.93E+07	1.11E+08	7.12E+07	6.88E+07	3.08E+07	26.44	27.44	27.38	26.06	26.42	27.07	26	25.84	24.4	27.38	26.42	25.84	0.560712	0.511832	0.882232
2224	Q14558	Phosphoril	PRPSAP1	9	36.8	2.81E+07	3.87E+07	2.93E+07	1.48E+07	2.41E+07	1.49E+07	3.14E+07	2.25E+07	1.11E+07	24.69	25.42	25.02	23.85	24.64	24.05	24.89	24.24	22.98	25.02	24.05	24.24	0.364276	0.408805	0.970558
2881	Q9BY76-2	Angiopoiet	ANGPTL4	3	9.8	3.30E+06		2.79E+06			1.36E+06				21.63		21.71			20.7				21.67	20.7		0.058117		

1521	P14550	Alcohol de	AKR1A1	6	19.1	4.83E+06	7.50E+06	7.18E+06	3.48E+06		4.54E+06	3.98E+06	3.94E+06	4.29E+06	22.13	23.03	23.05	21.72		22.39	21.87	21.72	21.38	23.03	22.06	21.72	0.524721	0.470053	0.249847
1857	P50502	Hsc70-inte	ST13	8	22	3.22E+07	3.84E+07	4.78E+07	2.15E+07	1.76E+07	3.55E+07	2.80E+07	1.62E+07	9.06E+06	24.96	25.4	25.8	24.43	24.23	25.33	24.73	23.75	22.63	25.4	24.43	23.75	0.417664	0.584778	1.050169
1642	P28482	Mitogen-a	MAPK1	9	31.4	1.17E+07	1.66E+07	1.60E+07	9.36E+06	8.44E+06	9.02E+06	5.85E+06	8.22E+06		23.46	24.14	24.15	23.17	23.17	23.35	22.45	22.75		24.14	23.17	22.6	0.394023	0.102737	0.213419
1537	P16435	NADPH--cy	POR	11	18.9	9.53E+06	1.29E+07	1.52E+07	5.53E+06	6.57E+06	1.13E+07	8.74E+06	5.48E+06	6.09E+06	23.15	23.82	24.08	22.4	22.84	23.61	23	22.15	22	23.82	22.84	22.15	0.479989	0.61286	0.540674
1505	P13010	X-ray repai	XRCC5	26	39.1	9.39E+07	1.44E+08	1.64E+08	6.68E+07	8.28E+07	1.17E+08	8.78E+07	7.49E+07	3.20E+07	26.59	27.46	27.65	26.09	26.49	27.13	26.37	25.96	24.46	27.46	26.49	25.96	0.568526	0.525997	1.004479
294	A2A274	Aconitate h	ACO2	9	16.4	9.30E+06	9.44E+06	1.05E+07	5.45E+06	4.69E+06	7.63E+06	6.58E+06	5.37E+06		23.12	23.35	23.58	22.38	22.36	23.14	22.62	22.11		23.35	22.38	22.36	0.233769	0.445151	0.363787
1082	M0R0Y2	Alpha-solu	NAPA	8	39.5	9.99E+06	1.21E+07	1.36E+07	5.57E+06	6.12E+06	1.10E+07	6.64E+06	5.88E+06	3.04E+06	23.23	23.73	23.93	22.42	22.75	23.59	22.63	22.25	20.97	23.73	22.75	22.25	0.358093	0.601974	0.869056
2715	Q96C86	m7GpppX	DCPS	4	15.4	1.89E+06	3.20E+06	2.81E+06		1.38E+06	1.54E+06		1.92E+06		20.78	21.87	21.73		20.66	20.84		20.72		21.73	20.75	20.72	0.593821	0.127493	
3258	Q9Y696	Chloride in	CLIC4	3	20.2		1.85E+06	3.15E+06	1.44E+06	1.27E+06	1.65E+06					21.12	21.9	20.44	20.52	20.94				21.51	20.52		0.549032	0.265654	
1577	P21796	Voltage-de	VDAC1	10	49.1	1.14E+07	1.61E+07	1.63E+07	8.38E+06	8.15E+06	1.30E+07	7.88E+06	8.03E+06	2.74E+06	23.42	24.1	24.18	22.99	23.11	23.85	22.85	22.72	20.84	24.1	23.11	22.72	0.415419	0.464208	1.12487
2877	Q9BXW7-2	Cat eye syr	CECR5	3	11.5	3.47E+06	3.33E+06	4.23E+06			1.67E+06				21.69	21.94	22.33			20.95				21.94	20.95		0.324261		
2013	P63092-3	Guanine nu	GNAS	3	12.1		6.14E+06	5.28E+06		1.61E+06	5.10E+06	4.67E+06	7.62E+06			22.73	22.66		20.86	22.55	22.11	22.64		22.69	21.7	22.38	0.053393	1.192056	0.374901
1585	P22307-6	Non-specif	SCP2	3	22.1	2.03E+06	4.56E+06	4.88E+06	2.63E+06	1.30E+06	5.76E+06	2.65E+06	2.10E+06		20.87	22.37	22.53	21.38	20.59	22.73	21.25	20.87		22.37	21.38	21.06	0.918621	1.078812	0.264497
2299	Q15738	Sterol-4-alp	NSDHL	3	10.5	2.61E+06	3.48E+06	4.32E+06			1.74E+06	3.51E+06			21.28	22	22.36			21.01	21.67			22	21.01	21.67	0.554281		
1948	P61106	Ras-relatec	RAB14	6	39.5	1.48E+07	9.48E+06	4.77E+06	5.35E+06	9.71E+06	4.49E+06	5.79E+06	4.13E+06	8.82E+06	23.77	23.37	22.5	22.35	23.36	22.37	22.43	21.78	22.59	23.37	22.37	22.43	0.649047	0.575354	0.429484
885	H0Y8C6	Importin-5	IPO5	26	33.5	7.06E+07	1.19E+08	1.14E+08	4.73E+07	6.16E+07	8.93E+07	7.81E+07	8.42E+07	2.13E+07	26.07	27.14	27.07	25.63	26.07	26.68	26.14	26.14	23.95	27.07	26.07	26.14	0.601922	0.528466	1.26752
1728	P36578	60S riboso	RPL4	23	44	3.06E+08	4.22E+08	3.46E+08	1.26E+08	2.38E+08	2.36E+08	2.44E+08	2.02E+08	2.35E+07	28.19	29.08	29	27.07	28	28.22	27.86	27.36	24.04	29	28	27.36	0.489496	0.613487	2.076031
2011	P63000	Ras-relatec	RAC1	5	30.2	1.56E+07	1.46E+07	1.49E+07	6.40E+06	7.13E+06	1.94E+07	9.39E+06	7.48E+06	1.31E+07	23.84	23.96	24.04	22.65	22.96	24.45	23.1	22.62	23.24	23.96	22.96	23.1	0.101578	0.963876	0.326979
1452	P09429	High mobil	HMGB1	9	35.8	5.12E+07	5.46E+07	7.91E+07	3.09E+07	1.94E+07	8.40E+07	3.53E+07	1.79E+07		25.58	25.93	26.58	24.93	24.37	26.59	25.08	23.88		25.93	24.93	24.48	0.507569	1.152294	0.845622
3125	Q9UHD1	Cysteine ar	CHORDC1	8	32.5	1.01E+07	2.33E+07	1.87E+07	9.87E+06	9.79E+06	1.76E+07	9.22E+06	1.01E+07		23.25	24.62	24.38	23.25	23.37	24.31	23.09	23.04		24.38	23.37	23.06	0.733921	0.578364	0.034108
1022	J3KTF8	Rho GDP-d	ARHGDIA	6	40.2	3.68E+07	6.00E+07	4.65E+07	2.70E+07	2.50E+07	2.75E+07	1.26E+07	1.30E+07	1.40E+07	25.11	26.08	25.75	24.74	24.72	24.95	23.5	23.42	23.34	25.75	24.74	23.42	0.495633	0.128823	0.081549
1709	P35270	Sepiapterir	SPR	5	24.9	4.88E+06	1.06E+07	1.09E+07	4.95E+06	5.14E+06	7.04E+06				22.15	23.52	23.64	22.24	22.5	23.01				23.52	22.5		0.831197	0.392674	
707	E9PK47	Alpha-1,4 g	PYGL	17	22.7	2.25E+07	2.57E+07	2.17E+07	7.19E+06	1.14E+07	1.44E+07	1.46E+07	6.94E+06	7.44E+06	24.36	24.8	24.61	22.81	23.59	24	23.77	22.49	22.33	24.61	23.59	22.49	0.220539	0.606011	0.790584
2482	Q7L1Q6-2	Basic leuci	BZW1	15	38.2	9.60E+07	1.14E+08	1.23E+08	6.42E+07	5.98E+07	8.35E+07	8.89E+07	7.52E+07	6.34E+07	26.63	27.04	27.24	26.02	26.02	26.58	26.39	25.97	25.38	27.04	26.02	25.97	0.312257	0.32254	0.504264
1002	J3KPP4	Luc7-like p	LUC7L3	2	5.1	2.16E+06	3.29E+06	4.32E+06	1.58E+06	1.63E+06	3.51E+06	3.10E+06			20.98	21.91	22.36	20.54	20.89	22.04	21.51			21.91	20.89	21.51	0.707093	0.786113	
1399	P05198	Eukaryotic	EIF2S1	13	52.7	2.60E+07	3.44E+07	4.23E+07	1.87E+07	1.50E+07	2.61E+07	1.97E+07	2.04E+07		24.58	25.23	25.56	24.2	23.99	24.82	24.22	24.12		25.23	24.2	24.17	0.498338	0.434897	0.069567
1999	P62851	40S riboso	RPS25	4	24	4.98E+07	7.05E+07	6.31E+07	3.18E+07	3.31E+07	4.53E+07	3.05E+07	3.25E+07	3.15E+07	25.55	26.31	26.18	25.02	25.15	25.68	24.85	24.78	24.43	26.18	25.15	24.78	0.408957	0.349532	0.22414
381	B4DLR8	NAD(P)H d	NQO1	7	34.2	1.61E+07	3.25E+07	3.52E+07	1.74E+07	1.54E+07	2.28E+07				23.9	25.11	25.3	24.09	24.02	24.65				25.11	24.09		0.758979	0.348008	
1970	P62136	Serine/thre	PPP1CA	11	35.5	2.20E+07	4.08E+07	3.83E+07	1.74E+07	1.99E+07	3.28E+07	1.39E+07	1.69E+07	3.78E+06	24.33	25.49	25.44	24.09	24.41	25.25	23.69	23.8	21.19	25.44	24.41	23.69	0.657769	0.601752	1.474937
1007	J3KQ32	Obg-like AT	OLA1	3	8.9		7.55E+06	8.51E+06	4.18E+06	4.05E+06	5.53E+06	5.28E+06				23.04	23.29	22	22.13	22.66	22.29			23.16	22.13	22.29	0.180845	0.346925	
189	A0A0A0MT	Isovaleryl-(	IVD	4	9.6		1.72E+06	4.11E+06			1.23E+06					20.99	22.29			20.61				21.64	20.61		0.920107		
2093	Q5T760	Serine/argi	SRSF11	3	11.1		3.03E+06	3.77E+06		1.52E+06	1.84E+06	2.53E+06				21.78	22.19		20.77	21.12	21.19			21.98	20.95	21.19	0.289876	0.24909	
2727	Q96EK6	Glucosamii	GNPNAT1	3	23.4	6.01E+06	1.12E+07	1.43E+07	2.88E+06	5.39E+06	9.61E+06		3.44E+06		22.45	23.61	23.99	21.49	22.57	23.44		21.53		23.61	22.57	21.53	0.804727	0.976172	
1685	P31939	Bifunctiona	ATIC	20	46.3	3.39E+07	3.86E+07	4.31E+07	1.45E+07	1.94E+07	2.58E+07	2.52E+07	1.38E+07	9.39E+06	25	25.41	25.58	23.82	24.37	24.8	24.59	23.5	22.69	25.41	24.37	23.5	0.298872	0.489654	0.950799
2061	Q00796	Sorbitol de	SORD	6	19.6	6.09E+06	1.01E+07	9.37E+06	5.21E+06	4.76E+06	6.41E+06	5.94E+06	5.64E+06	5.29E+06	22.46	23.45	23.43	22.33	22.39	22.87	22.47	22.2	21.78	23.43	22.39	22.2	0.565468	0.297041	0.350154
2904	Q9GZT8-2	NIF3-like p	NIF3L1	6	26	5.88E+06	1.33E+07	1.15E+07	6.62E+06	5.70E+06	1.06E+07	8.69E+06	9.77E+06		22.41	23.84	23.74	22.69	22.65	23.55	22.99	23		23.74	22.69	22.99	0.79908	0.509005	0.005113
1617	P25788-2	Proteasom	PSMA3	6	25.8	1.10E+07	1.82E+07	1.83E+07	9.35E+06	8.86E+06	1.67E+07				23.38	24.28	24.36	23.16	23.23	24.23				24.28	23.23		0.54551	0.601462	
978	I3L0U2	Testisin	PRSS21	3	14.2		6.73E+06	6.14E+06		3.83E+06	2.47E+06					22.86	22.85		22.07	21.54				22.85	21.8		0.006147	0.372074	
1381	P04181	Ornithine a	OAT	23	56.3	1.54E+08	1.51E+08	1.19E+08	7.17E+07	7.87E+07	6.59E+07	1.77E+08	1.57E+08	9.90E+07	27.25	27.54	27.16	26.2	26.4	26.19	27.4	27	26.03	27.25	26.2	27	0.197954	0.120042	0.705405
1690	P31948	Stress-indu	STIP1	25	38.5	7.03E+07	7.94E+07	7.73E+07	3.75E+07	4.11E+07	7.26E+07	4.78E+07	3.12E+07	2.02E+07	26.06	26.5	26.56	25.27	25.44	26.38	25.47	24.75	23.88	26.5	25.44	24.75	0.271342	0.59643	0.797996
1423	P07737	Profilin-1	PFN1	11	85	4.22E+08	7.32E+08	5.57E+08	3.39E+08	3.64E+08	6.27E+08	4.86E+08	3.91E+08	1.68E+08	28.69	29.92	29.58	28.31	28.52	29.51	28.81	28.11	26.59	29.58	28.52	28.11	0.632767	0.637156	1.138202
230	F5GWX5	Chromodo	CHD4	12	7.6	1.17E+07	2.07E+07	1.92E+07	8.31E+06	9.66E+06	1.26E+07	9.22E+06	5.28E+06		23.46	24.44	24.41	22.98	23.35	23.81	23.09	22.08		24.41	23.35	22.58	0.556986	0.413374	0.710839
1880	P51858	Hepatoma-	HDGF	9	51.7	1.02E+07	1.57E+07	1.87E+07	5.87E+06	7.34E+06	1.22E+07	7.35E+06	8.07E+06	6.94E+06	23.27	24.07	24.38	22.5	23	23.73	22.75	22.73	22.22	24.07	23	22.73	0.573909	0.618246	0.298604
1865	P50991	T-complex	CCT4	30	64.2	1.17E+09	1.16E+09	1.08E+09	8.08E+08	6.86E+08	8.58E+08	8.00E+08	6.73E+08	4.61E+08	30.5	30.78	30.75	29.68	29.47	29.94	29.45	28.89	28.16	30.75	29.68	28.89	0.155653	0.23131	0.64725
1527	P15121	Aldose red	AKR1B1	8	36.1	2.83E+07	5.03E+07	3.65E+07	1.15E+07	1.84E+07	3.24E+07	1.15E+07	1.47E+07		24.7	25.77	25.36	23.45	24.29	25.23	23.37	23.62		25.36	24.29	23.49	0.539771	0.891067	0.176666
121	A0A087X0	Acyl-coenz	ACOT2	3	7.8	2.91E+06	3.74E+06	3.51E+06	2.02E+06	1.63E+06	2.00E+06	2.21E+06	2.06E+06		21.45	22.12	22.04	20.96	20.89	21.26	20.98	20.83		22.04	20.96	20.91	0.366958	0.194562	0.102965
1711	P35579	Myosin-9	MYH9	151	62.8	2.83E+09	4.14E+09	3.35E+09	3.37E+09	2.85E+09	3.03E+09	2.35E+09	2.59E+09	1.85E+09	31.99	33.05	32.67	31.59	31.46	31.89	31.11	31.01	29.79	32.67	31.59	31.01	0.536507	0.219848	0.73376
785	F6TLX2	Glyoxalase	GLOD4	4	8.8	4.73E+06	6.93E+06	7.18E+06	3.61E+06		3.23E+06				22.09	22.93	23.05	21.77		21.92				22.93	21.85		0.520666	0.107545	
1270	Q5JRI1	Serine/argi	SRSF10	6	33.7	8.15E+06	1.03E+07	1.04E+07	5.51E+06	4.14E+06	6.91E+06		3.63E+06		22.92	23.48	23.57	22.4	22.15	22.98		21.59		23.48	22.4	21.59	0.352628	0.42639	
1968	P61981	14-3-3 prot	YWHAG	4	33.6	1.02E+08	1.22E+08	1.05E+08	4.12E+07	1.50E+08	5.61E+07	4.95E+07	1.34E+08		26.72	27.24	27.03	25.4	27.36	25.95	25.55	26.77		27.03	25.95	26.16	0.262711	1.00852	0.860706
839	G3V4F7	Signal reco	SRP54	5	11.4			1.84E+07		6.10E+06	1.27E+07						24.37		22.74	23.81				24.37	23.28			0.758819	
1559	P19338	Nucleolin	NCL	24	32.1	3.12E+08	5.16E+08	4.10E+08	2.30E+08	2.57E+08	2.95E+08	2.85E+08	1.87E+08	4.26E+07	28.24	29.26	29.19	27.82	28.1	28.58	28	27.28	24.89	29.19	28.1	27.28	0.570308	0.386197	1.624492
677	E9PCB6	Neurolysin	NLN	4	8.3	2.90E+06	4.50E+06	5.20E+06	2.42E+06	1.88E+06	4.65E+06	2.62E+06	1.89E+06	1.97E+06	21.43	22.35	22.62	21.26	21.08	22.41	21.23	20.7	20.13	22.35	21.26	20.7	0.620505	0.72097	0.545667
2021	P67936	Tropomyos	TPM4	17	48.8	4.54E+07	9.11E+07	1.29E+08	4.58E+07	2.85E+07	4.55E+07	5.53E+07	5.37E+07	2.25E+07	25.43	26.69	27.32	25.59	24.94	25.68	25.7	25.51	23.99	26.69	25.59	25.51	0.961375	0.40534	0.934981
631	E7ENJ6	AP-1 comp	AP1M1	8	22.7	5.50E+06	1.14E+07	1.04E+07	4.25E+06	5.02E+06	7.93E+06	7.70E+06	6.54E+06		22.29	23.63	23.57	22.03	22.47	23.19	22.82	22.39		23.57	22.47	22.6	0.756913	0.585528	0.303538
2185	Q13595	Transform	TRA2A	8	30.1	3.66E+07	2.20E+07	2.02E+07	9.54E+06	1.02E+07	1.35E+07	5.43E+06	1.48E+07		25.1	24.55	24.48	23.19	23.45	23.9	22.35	23.63		24.55	23.45	22.99	0.337275	0.356728	0.901961
1688	P31946	14-3-3 prot	YWHAB	8	58.5	4.21E+07	6.24E+07	5.08E+07	2.79E+07	2.25E+07	2.69E+07	2.01E+07	1.35E+07	1.13E+07	25.32	26.14	25.9	24.79	24.56	24.89	24.26	23.46	23.01	25.9	24.79	23.46	0.420989	0.16796	0.632264
2474	Q70UQ0	Inhibitor of	IKBIP	3	16.3	2.26E+06	3.35E+06	3.37E+06	1.43E+06	1.59E+06	1.71E+06	1.24E+06		2.23E+06	21.04	21.94	21.98	20.43	20.83	20.98	20.09		20.49	21.94	20.83	20.29	0.533472	0.28139	0.282604
1626	P26639	Threonine-	TARS	28	41.1	6.89E+07	9.05E+07	9.61E+07	3.68E+07	4.45E+07	8.25E+07	5.60E+07	4.83E+07	8.73E+06	25.99	26.68	26.88	25.26	25.56	26.57	25.71	25.35	22.57	26.68	25.56	25.35	0.470776	0.684538	1.71411
1557	P19022-2	Cadherin-2	CDH2	5	9.4	1.23E+06	2.77E+06	2.56E+06	1.03E+06	1.24E+06	1.37E+06				20.14	21.63	21.59	20.14	20.47	20.72				21.59	20.47		0.847836	0.287627	
1646	P29401	Transketol	TKT	24	44.9	2.50E+08	3.67E+08	4.04E+08	1.97E+08	2.08E+08	3.09E+08	3.56E+08	2.70E+08	1.18E+08	27.96	28.88	29.12	27.65	27.76	28.64	28.25	27.66	26.21	28.88	27.76	27.66	0.616404	0.546669	1.052887
751	F5GXM3	IST1 homo	IST1	2	15.6		3.03E+06	3.54E+06	1.96E+06	1.38E+06			2.01E+06			21.78	22.06	20.94	20.66			20.8		21.92	20.8	20.8	0.203054	0.199371	
3132	Q9UHW5	GPN-loop (	GPN3	2	9.9			1.92E+06			7.42E+05						21.23			20.11				21.23	20.11				
290	A0AVT1	Ubiquitin-l	UBA6	7	6.9	1.58E+07	2.54E+07	2.56E+07	1.12E+07	1.19E+07	1.24E+07		8.98E+06		23.86	24.77	24.83	23.41	23.64	23.78		22.87		24.77	23.64	22.87	0.543453	0.187139	
2999	Q9NQ29-2	Putative RI	LUC7L	2	8.6		2.88E+06	4.55E+06	9.85E+05	1.66E+06	2.24E+06	2.21E+06				21.7	22.43	20.03	20.93	21.43	20.98			22.06	20.93	20.98	0.51698	0.710051	
1570	Q9NRW1	Ras-relatec	RAB6B	3	16.3	2.06E+07	1.00E+07		5.22E+06	5.98E+06	6.09E+06				24.25	23.44		22.33	22.71	22.8				23.84	22.71		0.573107	0.24908	
139	A0A087X2	Serine/argi	SRSF3	3	49.5	5.39E+07	3.29E+07	1.37E+07	1.61E+07	2.14E+07	1.03E+07	6.49E+06	1.36E+07		25.66	25.13	23.93	24	24.5	23.52	22.6	23.48		25.13	24	23.04	0.884866	0.492957	0.624341
1616	P25786	Proteasom	PSMA1	12	43.7	2.57E+07	4.56E+07	4.07E+07	2.10E+07	1.98E+07	4.33E+07			1.12E+07	24.56	25.63	25.54	24.39	24.4	25.63			22.99	25.54	24.4	22.99	0.593417	0.713477	
1045	K7EQ02	DAZ-associ	DAZAP1	6	30.3	3.68E+06	1.04E+07	8.01E+06	3.61E+06	3.88E+06	8.34E+06	4.02E+06	3.84E+06		21.78	23.49	23.22	21.77	22.09	23.24	21.9	21.69		23.22	22.09	21.8	0.922032	0.775756	0.148665
1901	P53396-2	ATP-citrate	ACLY	38	41.3	1.13E+08	1.68E+08	1.68E+08	7.65E+07	8.68E+07	1.21E+08	1.04E+08	8.66E+07	4.20E+07	26.84	27.73	27.73	26.29	26.59	27.21	26.64	26.19	24.88	27.73	26.59	26.19	0.509904	0.468721	0.913958
1883	P52209-2	6-phospho	PGD	13	35.7	5.66E+07	7.93E+07	8.78E+07	3.98E+07	3.67E+07	5.98E+07	4.34E+07	4.09E+07	2.73E+07	25.74	26.49	26.71	25.35	25.3	26.05	25.33	25.08	24.23	26.49	25.35	25.08	0.512767	0.42024	0.574363
2284	Q15427	Splicing fac	SF3B4	2	5.2	3.76E+06	6.74E+06	5.59E+06	2.74E+06	2.70E+06	4.53E+06		3.70E+06		21.81	22.86	22.75	21.42	21.6	22.38		21.63		22.75	21.6	21.63	0.579208	0.513799	
3070	Q9NZL9	Methionin	MAT2B	6	23.4	1.60E+07	2.05E+07	1.99E+07	9.24E+06	9.17E+06	1.43E+07	1.26E+07	1.18E+07	7.52E+06	23.89	24.43	24.46	23.15	23.27	23.98	23.5	23.28	22.36	24.43	23.27	23.28	0.318298	0.450406	0.608269
1575	P21333-2	Filamin-A	FLNA	147	66.9	8.44E+09	9.48E+09	8.65E+09	7.67E+09	7.68E+09	7.27E+09	6.58E+09	7.62E+09	4.54E+09	33.42	34.26	34.31	33.09	33.07	33.37	32.43	32.23	30.61	34.26	33.09	32.23	0.49904	0.165959	0.995794
97	A0A087WY	Tumor pro	TPD52L2	3	17.1		4.08E+06	3.10E+06	1.93E+06	1.57E+06	3.16E+06					22.25	21.88	20.9	20.8	21.9				22.07	20.9		0.260771	0.608363	
1534	P16152	Carbonyl re	CBR1	10	56	1.26E+07	2.32E+07	2.35E+07	1.02E+07	1.02E+07	1.39E+07	2.82E+07	2.12E+07		23.56	24.61	24.73	23.29	23.45	23.95	24.75	24.16		24.61	23.45	24.45	0.646331	0.342291	0.414002
1523	P14625	Endoplasm	HSP90B1	32	43.5	3.48E+08	4.46E+08	2.94E+08	1.77E+08	1.76E+08	2.40E+08	3.10E+08	1.15E+08	4.18E+07	28.43	29.15	28.73	27.47	27.56	28.26	28.1	26.53	24.87	28.73	27.56	26.53	0.361998	0.432984	1.614162

1862	P50750	Cyclin-dep	CDK9	3	9.9		3.18E+06	3.52E+06		1.53E+06						21.87	22.05		20.78					21.96	20.78		0.127766		
236	A0A0C4DG	Zinc finger	ZRANB2	7	23.1	1.82E+07	1.86E+07	1.75E+07	8.85E+06	8.16E+06	1.10E+07	1.13E+07	1.21E+07		24.1	24.3	24.29	23.07	23.12	23.59	23.34	23.34		24.29	23.12	23.34	0.11443	0.286904	0.000966
1293	O76003	Glutaredo	GLRX3	9	34	8.85E+06	1.36E+07	1.38E+07	7.19E+06	5.68E+06	5.66E+06	2.91E+06	6.71E+06	6.41E+06	23.04	23.87	23.94	22.81	22.64	22.69	21.42	22.43	22.11	23.87	22.69	22.11	0.502549	0.08481	0.513879
1599	P23526	Adenosylh	AHCY	19	39.1	1.01E+08	1.63E+08	1.68E+08	8.37E+07	8.30E+07	1.14E+08	1.26E+08	1.15E+08	6.25E+07	26.7	27.68	27.73	26.48	26.5	27.11	26.94	26.53	25.36	27.68	26.5	26.53	0.581294	0.361033	0.818688
376	B4DJV2	Citrate syn	CS	9	29.1	3.70E+07	4.49E+07	4.44E+07	1.76E+07	2.02E+07	3.07E+07	2.67E+07	2.26E+07	1.51E+07	25.11	25.63	25.65	24.1	24.44	25.13	24.66	24.25	23.46	25.63	24.44	24.25	0.304493	0.521894	0.613767
311	A6NFX8	ADP-sugar	NUDT5	9	40.1	1.23E+07	2.24E+07	2.16E+07	1.03E+07	9.87E+06	1.20E+07	1.32E+07	1.15E+07	4.25E+06	23.52	24.58	24.6	23.32	23.39	23.69	23.57	23.22	21.37	24.58	23.39	23.22	0.614548	0.199643	1.183239
131	A0A087X1	Nuclease-s	YBX1	8	34.1	2.05E+07	3.69E+07	5.92E+07	1.32E+07	1.64E+07	2.28E+07	5.11E+06	1.75E+07		24.24	25.33	26.09	23.68	24.14	24.65	22.24	23.86		25.33	24.14	23.05	0.927739	0.486309	1.143954
1597	P23396	40S riboso	RPS3	18	66.7	1.27E+08	1.74E+08	2.15E+08	9.08E+07	7.62E+07	1.80E+08	7.26E+07	9.98E+07	6.89E+07	26.97	27.78	28.2	26.59	26.39	27.86	26.04	26.36	25.5	27.78	26.59	26.04	0.626604	0.795978	0.439481
1853	P50395	Rab GDP d	GDI2	26	66.5	2.30E+08	3.09E+08	2.94E+08	1.78E+08	1.70E+08	2.28E+08	2.38E+08	2.19E+08	1.13E+08	27.89	28.69	28.73	27.49	27.5	28.15	27.83	27.46	26.16	28.69	27.5	27.46	0.472908	0.381281	0.873324
1628	P26641	Elongation	EEF1G	22	43.7	8.95E+08	1.29E+09	9.95E+08	6.75E+08	6.81E+08	8.81E+08	9.30E+08	9.88E+08	5.09E+08	29.86	31.21	30.66	29.25	29.46	29.99	29.71	29.74	28.34	30.66	29.46	29.71	0.679658	0.379685	0.798217
2670	Q92688-2	Acidic leuc	ANP32B	5	41	7.09E+06	1.63E+07	1.96E+07	5.07E+06	6.80E+06	1.08E+07	6.38E+06			22.69	24.11	24.44	22.29	22.91	23.57	22.57			24.11	22.91	22.57	0.92917	0.641031	
787	F8VNT9	Tetraspani	CD63	2	9		6.93E+06	8.31E+06	3.89E+06	3.11E+06	5.62E+06	3.70E+06	2.62E+06			22.93	23.27	21.89	21.78	22.69	21.78	21.15		23.1	21.89	21.46	0.242038	0.495434	0.444632
2176	Q13442	28 kDa hea	PDAP1	3	21.5	5.34E+06	9.96E+06	1.26E+07	4.70E+06	4.31E+06	4.95E+06	5.26E+06	4.17E+06		22.26	23.43	23.84	22.17	22.22	22.5	22.29	21.8		23.43	22.22	22.05	0.819055	0.174809	0.344082
2159	Q13177	Serine/thre	PAK2	9	26.7	7.21E+06	9.33E+06	1.14E+07	4.54E+06	3.53E+06	5.29E+06		1.85E+06		22.71	23.33	23.72	22.11	21.95	22.6		20.65		23.33	22.11	20.65	0.507798	0.341002	
1979	P62269	40S riboso	RPS18	10	49.3	4.18E+07	6.47E+07	7.84E+07	2.21E+07	2.96E+07	6.28E+07	1.97E+07	1.87E+07	1.14E+07	25.31	26.21	26.57	24.47	24.99	26.14	24.22	23.96	23.03	26.21	24.99	23.96	0.649124	0.857868	0.626793
2125	Q10471	Polypeptid	GALNT2	10	22.8	1.07E+07	1.76E+07	1.69E+07	7.04E+06	7.38E+06	1.15E+07	7.60E+06	6.35E+06	8.92E+06	23.33	24.25	24.24	22.78	23.01	23.64	22.78	22.36	22.6	24.24	23.01	22.6	0.529381	0.444143	0.212197
643	E7EQR4	Ezrin	EZR	10	31.9	1.68E+07	3.10E+07	3.48E+07	1.04E+07	1.30E+07	2.07E+07	2.70E+07	1.23E+07	5.87E+06	23.96	25.02	25.27	23.33	23.79	24.53	24.68	23.36	21.93	25.02	23.79	23.36	0.692315	0.608163	1.372529
1556	P18669	Phosphogl	PGAM1	15	67.3	5.14E+07	1.04E+08	1.65E+08	3.49E+07	4.64E+07	1.30E+08	1.36E+07	2.04E+07		25.59	26.88	27.67	25.18	25.64	27.37	23.64	24.12		26.88	25.64	23.88	1.050483	1.152981	0.337972
1447	P08758	Annexin A	ANXA5	16	45.6	3.79E+07	5.77E+07	7.99E+07	2.33E+07	2.60E+07	3.94E+07	2.28E+07	1.14E+07	1.46E+07	25.14	26.03	26.61	24.53	24.79	25.49	24.45	23.21	23.41	26.03	24.79	23.41	0.738586	0.494462	0.666348
808	F8W031	Protein car	CNPY2	4	21.3	4.07E+06	7.37E+06	7.17E+06	3.46E+06	3.05E+06	5.79E+06	3.54E+06			21.92	23	23.04	21.71	21.76	22.74	21.69			23	21.76	21.69	0.638828	0.579887	
1231	O60664-4	Perilipin-3	PLIN3	10	38.4	1.49E+07	2.51E+07	2.07E+07	1.02E+07	8.88E+06	1.58E+07	1.40E+07	1.31E+07	5.27E+06	23.78	24.74	24.53	23.29	23.23	24.13	23.7	23.43	21.77	24.53	23.29	23.43	0.505481	0.502486	1.044925
1552	P18206-2	Vinculin	VCL	35	38.6	4.84E+08	5.61E+08	4.96E+08	2.71E+08	2.72E+08	3.49E+08	3.60E+08	2.86E+08	7.11E+08	28.88	29.44	29.42	28.12	28.18	28.7	28.28	27.77	28.76	29.42	28.18	28.28	0.319534	0.320599	0.493613
342	A9Z1X7	Serine/argi	SRRM1	5	9.5	2.27E+06	3.08E+06	2.68E+06	1.37E+06	1.15E+06	1.55E+06				21.06	21.81	21.65	20.41	20.37	20.85				21.65	20.41		0.394383	0.265535	
1460	P09960	Leukotrien	LTA4H	9	20.5	8.63E+06	1.15E+07	1.46E+07	4.89E+06	4.80E+06	9.78E+06	8.81E+06	5.10E+06		23	23.64	24.02	22.22	22.4	23.46	23.02	22.05		23.64	22.4	22.53	0.515391	0.671751	0.683294
208	A0A0B4J2	3-ketoacyl-	ACAA2	8	27.9	5.29E+06	9.20E+06	9.29E+06	4.33E+06	3.63E+06	5.43E+06	5.62E+06	5.43E+06		22.25	23.31	23.42	22.06	21.99	22.64	22.4	22.13		23.31	22.06	22.26	0.647455	0.355253	0.194388
1494	P11908	Ribose-pho	PRPS2	14	53.1	4.61E+07	1.17E+08	1.01E+08	2.83E+07	4.82E+07	7.05E+07	5.44E+07	2.37E+07	6.40E+06	25.44	27.11	26.96	24.81	25.7	26.32	25.68	24.32	22.11	26.96	25.7	24.32	0.921862	0.761516	1.802821
1417	P07195	L-lactate d	LDHB	14	46.7	3.17E+08	5.27E+08	5.40E+08	2.44E+08	2.44E+08	3.64E+08	3.27E+08	2.31E+08	1.55E+08	28.3	29.29	29.53	27.98	28.02	28.79	28.18	27.55	26.5	29.29	28.02	27.55	0.650245	0.456562	0.851227
2117	Q08752	Peptidyl-pr	PPID	6	15.1	2.71E+06	9.33E+06	6.99E+06	3.51E+06	2.78E+06	4.30E+06	3.56E+06			21.32	23.33	23	21.73	21.64	22.31	21.7			23	21.73	21.7	1.074244	0.360077	
2240	Q14847	LIM and SH	LASP1	4	21.5	8.22E+06	1.50E+07	1.45E+07	6.83E+06	5.69E+06	7.49E+06	6.25E+06	6.31E+06		22.93	24.01	24.01	22.74	22.65	23.1	22.54	22.35		24.01	22.74	22.44	0.621514	0.240098	0.13314
1778	P43034	Platelet-ac	PAFAH1B1	9	24.6	5.08E+06	1.10E+07	9.79E+06	4.92E+06	3.67E+06	4.87E+06	3.78E+06	4.42E+06		22.19	23.58	23.51	22.23	22	22.47	21.8	21.85		23.51	22.23	21.83	0.779926	0.237327	0.039173
1754	P40925	Malate deh	MDH1	9	35.9	6.33E+07	8.87E+07	9.82E+07	3.00E+07	3.89E+07	5.73E+07	3.76E+07	4.26E+07	1.37E+07	25.86	26.64	26.9	24.86	25.36	25.98	25.16	25.14	23.31	26.64	25.36	25.14	0.542474	0.562126	1.062221
120	A0A087X0	Sorting ne	SNX12	3	18		4.17E+06	4.28E+06	2.07E+06	1.78E+06	3.47E+06					22.27	22.35	20.98	21.03	22.03				22.31	21.03		0.054119	0.593874	
924	H3BV80	RNA-bindi	RNPS1	3	16.1	1.02E+07	8.45E+06	8.32E+06	2.84E+06	3.63E+06	4.70E+06				23.27	23.18	23.27	21.47	21.99	22.43				23.27	21.99		0.050174	0.476604	
2302	Q15785	Mitochond	TOMM34	7	30.7	5.74E+06	1.01E+07	9.17E+06	4.55E+06	3.87E+06	9.06E+06		5.95E+06		22.37	23.45	23.4	22.12	22.08	23.36		22.27		23.4	22.12	22.27	0.611044	0.72615	
1441	P08621-2	U1 small n	SNRNP70	7	19.2	7.93E+06	1.14E+07	1.48E+07	3.95E+06	4.63E+06	7.85E+06	4.38E+06			22.86	23.63	24.03	21.92	22.35	23.17	22.03			23.63	22.35	22.03	0.599292	0.632644	
1859	P50552	Vasodilato	VASP	4	12.6		5.32E+06	6.17E+06		1.65E+06	3.21E+06	2.96E+06	2.47E+06			22.55	22.86		20.92	21.92	21.46	21.06		22.7	21.42	21.26	0.222481	0.704261	0.277525
1511	P13667	Protein dis	PDIA4	24	37.5	4.21E+07	5.48E+07	6.73E+07	2.51E+07	2.45E+07	4.58E+07	3.18E+07	1.87E+07	5.50E+06	25.32	25.95	26.34	24.64	24.67	25.7	24.92	23.96	21.84	25.95	24.67	23.96	0.51126	0.603804	1.576832
625	E5RIW3	Tubulin-sp	TBCA	6	53.6	6.53E+06	1.36E+07	1.79E+07	5.37E+06	5.44E+06	8.91E+06	2.94E+06			22.56	23.87	24.35	22.36	22.58	23.32	21.44			23.87	22.58	21.44	0.92232	0.501403	
1960	P61604	10 kDa hea	HSPE1	8	67.6	4.88E+07	5.03E+07	7.39E+06	4.52E+06	1.73E+07	1.25E+08	2.39E+07	1.91E+07	3.91E+07	25.51	25.77	23.1	22.1	24.22	27.26	24.5	23.99	24.75	25.51	24.22	24.5	1.471585	2.593563	0.387707
205	A0A0B4J1	Serine/argi	SRSF7	9	55.5	1.64E+08	1.29E+08	1.03E+08	5.78E+07	5.94E+07	7.97E+07	2.27E+07	4.91E+07		27.38	27.3	26.99	25.88	26.01	26.53	24.44	25.37		27.3	26.01	24.9	0.203985	0.34136	0.653236
1576	P21399	Cytoplasmi	ACO1	5	8.3		6.41E+06	3.64E+06		1.21E+06	3.13E+06	6.48E+06	1.99E+06			22.8	22.13		20.44	21.88	22.59	20.77		22.46	21.16	21.68	0.474655	1.016083	1.290595
1755	P40926	Malate deh	MDH2	15	59.5	9.48E+07	1.45E+08	1.41E+08	4.82E+07	6.43E+07	1.02E+08	8.20E+07	6.54E+07	2.90E+07	26.59	27.47	27.47	25.65	26.15	26.9	26.25	25.79	24.31	27.47	26.15	25.79	0.50648	0.626137	1.01256
1466	P10155-3	60 kDa SS-	TROVE2	6	14.5	1.34E+07	1.45E+07	1.55E+07	6.35E+06	5.40E+06	1.03E+07	6.76E+06	7.47E+06	7.90E+06	23.63	23.95	24.11	22.63	22.58	23.52	22.65	22.61	22.44	23.95	22.63	22.61	0.246416	0.529487	0.109197
1414	P06756-3	Integrin al	ITGAV	10	13.7	5.08E+06	7.76E+06	1.06E+07	3.36E+06	3.00E+06	4.51E+06	2.20E+06	2.70E+06	1.50E+06	22.19	23.06	23.59	21.69	21.74	22.38	20.96	21.22	19.33	23.06	21.74	20.96	0.706823	0.385677	1.023048
1105	O00273-2	DNA fragm	DFFA	7	32.8	2.94E+06	6.92E+06	7.04E+06	3.10E+06	2.29E+06	3.10E+06	3.52E+06			21.47	22.92	23.01	21.59	21.35	21.86	21.68			22.92	21.59	21.68	0.862435	0.25795	
1592	P23246	Splicing fa	SFPQ	16	22.9	4.42E+08	2.96E+08	3.42E+08	1.75E+08	3.90E+08	1.20E+08	1.90E+08	9.42E+07	1.88E+08	28.8	28.59	28.96	27.46	28.63	27.18	27.53	26.29	26.77	28.8	27.46	26.77	0.187887	0.766104	0.627486
1404	P05556-2	Integrin be	ITGB1	7	10.9	8.04E+06	1.70E+07	1.69E+07	4.81E+06	6.70E+06	9.47E+06	1.31E+07	6.77E+06	2.61E+06	22.89	24.21	24.24	22.19	22.88	23.42	23.56	22.44	20.73	24.21	22.88	22.44	0.77249	0.613059	1.426026
3253	Q9Y617	Phosphose	PSAT1	14	46.2	2.92E+07	4.81E+07	4.59E+07	2.04E+07	1.78E+07	2.21E+07	3.13E+07	2.45E+07	1.93E+07	24.77	25.67	25.7	24.33	24.24	24.61	24.87	24.37	23.79	25.67	24.33	24.37	0.530558	0.190745	0.537952
915	H0YN26	Acidic leuc	ANP32A	9	39	2.40E+07	3.30E+07	3.86E+07	5.62E+06	1.31E+07	1.79E+07	1.00E+07			24.48	25.14	25.46	22.43	23.81	24.36	23.2			25.14	23.81	23.2	0.501958	0.991788	
1922	P55084-2	Trifunction	HADHB	11	32.5	1.17E+07	2.25E+07	2.03E+07	9.24E+06	7.03E+06	1.12E+07	1.15E+07	1.02E+07	6.86E+06	23.46	24.59	24.49	23.15	22.95	23.61	23.37	23.05	22.2	24.49	23.15	23.05	0.623465	0.337675	0.602163
719	E9PLL6	60S riboso	RPL27A	4	42.6	2.47E+07	3.67E+07	3.03E+07	1.27E+07	1.24E+07	4.25E+07	7.72E+06	2.80E+07	1.53E+07	24.51	25.32	25.06	23.61	23.71	25.59	22.83	24.55	23.48	25.06	23.71	23.48	0.416706	1.114459	0.872339
2049	P98082-2	Disabled h	DAB2	5	11.4	4.92E+06	6.25E+06	5.44E+06	1.61E+06	2.31E+06	2.46E+06	3.36E+06			22.16	22.76	22.71	20.58	21.36	21.53	21.63			22.71	21.36	21.63	0.336219	0.509848	
1435	P08238	Heat shock	HSP90AB1	30	58	9.83E+08	1.41E+09	1.63E+09	8.62E+08	1.02E+09	1.48E+09	1.05E+09	7.52E+08	4.34E+08	30.05	31.48	31.48	29.76	30.12	31	29.98	29.11	28.01	31.48	30.12	29.11	0.821723	0.640162	0.988014
2142	Q12907	Vesicular i	LMAN2	9	32.3	7.90E+06	1.61E+07	1.38E+07	5.91E+06	5.46E+06	1.00E+07	5.27E+06	5.91E+06	4.40E+06	22.84	24.1	23.94	22.51	22.59	23.49	22.29	22.26	21.42	23.94	22.59	22.26	0.684746	0.544001	0.497167
1594	P23284	Peptidyl-pr	PPIB	12	48.1	8.61E+07	1.22E+08	1.24E+08	5.71E+07	4.78E+07	8.96E+07	4.10E+07	4.39E+07	3.21E+07	26.39	27.24	27.25	25.87	25.68	26.69	25.27	25.17	24.47	27.24	25.87	25.17	0.496515	0.533752	0.435312
2849	Q9BTT0-3	Acidic leuc	ANP32E	3	22.3	3.71E+06	1.28E+07	1.27E+07	2.58E+06	4.92E+06	8.96E+06	3.35E+06			21.78	23.8	23.85	21.35	22.43	23.33	21.63			23.8	22.43	21.63	1.178645	0.989358	
1956	P61247	40S riboso	RPS3A	13	48.9	8.99E+07	1.16E+08	1.04E+08	4.77E+07	3.16E+07	6.27E+07	4.96E+07	2.82E+07	2.11E+07	26.49	27.09	27.01	25.63	25.07	26.13	25.56	24.57	23.93	27.01	25.63	24.57	0.3233	0.53208	0.818982
1189	O43290	U4/U6.U5	SART1	7	11.2	3.82E+06	8.61E+06	4.90E+06	2.20E+06	2.65E+06	1.86E+06	2.99E+06			21.84	23.21	22.54	21.08	21.57	21.16	21.48			22.54	21.16	21.48	0.685195	0.261145	
2808	Q99714-2	3-hydroxy	HSD17B10	7	52.4	2.50E+07	9.32E+06	1.51E+07	3.62E+06	5.91E+06	1.20E+07	6.07E+06	6.77E+06		24.52	23.32	24.07	21.78	22.69	23.69	22.51	22.44		24.07	22.69	22.48	0.604305	0.959594	0.049007
3179	Q9UQ80	Proliferati	PA2G4	17	45.2	5.85E+07	8.56E+07	8.96E+07	3.33E+07	3.39E+07	6.25E+07	4.54E+07	3.73E+07	2.48E+07	25.78	26.56	26.76	25.1	25.17	26.12	25.4	24.97	24.11	26.56	25.17	24.97	0.520646	0.571691	0.654401
319	A6NKB8	Aminopept	RNPEP	8	19.5	9.73E+06	1.04E+07	1.23E+07	3.20E+06	3.89E+06	7.42E+06	4.89E+06	3.40E+06		23.18	23.49	23.79	21.64	22.09	23.08	22.16	21.52		23.49	22.09	21.84	0.304842	0.738581	0.459279
1427	P07900	Heat shock	HSP90AA1	49	53.8	1.07E+09	1.87E+09	2.13E+09	9.00E+08	1.14E+09	1.57E+09	1.26E+09	8.91E+08	4.96E+08	30.43	31.83	32.25	29.93	30.42	31.26	30.43	29.52	28.3	31.83	30.42	29.52	0.95111	0.674174	1.069317
1629	P26885	Peptidyl-pr	FKBP2	3	28.9		7.95E+06	8.71E+06	2.82E+06	3.15E+06	3.61E+06	2.81E+06	2.94E+06			23.1	23.32	21.45	21.8	22.07	21.36	21.33		23.21	21.8	21.34	0.156649	0.313755	0.014855
3111	Q9UBV8	Peflin	PEF1	2	8.5			5.02E+06	1.80E+06	2.62E+06							22.57	20.76	21.55					22.57	21.15			0.556864	
891	H0YCU9	Transgelin	TAGLN	6	41.1	9.88E+06	1.56E+07	1.09E+07	4.62E+06	4.32E+06	1.19E+07	3.14E+06	4.44E+06		23.21	24.06	23.64	22.15	22.23	23.68	21.54	21.87		23.64	22.23	21.71	0.423448	0.864103	0.228242
219	C9J9K3	40S riboso	RPSA	13	65.9	1.53E+08	2.18E+08	2.04E+08	7.99E+07	9.36E+07	1.47E+08	1.12E+08	1.18E+08	5.77E+07	27.24	28.14	28.14	26.35	26.72	27.59	26.75	26.62	25.24	28.14	26.72	26.62	0.516979	0.637527	0.837343
1403	P05455	Lupus La p	SSB	9	27.9	8.84E+06	1.90E+07	1.75E+07	2.99E+06	6.69E+06	7.96E+06	9.57E+06	5.03E+06		23.04	24.34	24.29	21.53	22.87	23.19	23.13	22.04		24.29	22.87	22.58	0.738182	0.879249	0.774696
2869	Q9BWD1	Acetyl-CoA	ACAT2	11	42.3	8.43E+06	2.88E+07	3.00E+07	6.35E+06	1.09E+07	1.64E+07	1.06E+07	7.56E+06		22.96	24.94	25.04	22.63	23.52	24.19	23.26	22.63		24.94	23.52	22.94	1.174054	0.783872	0.443636
1920	P55072	Transitiona	VCP	46	68.4	5.27E+08	7.21E+08	5.08E+08	2.56E+08	1.96E+08	3.67E+08	8.03E+08	3.82E+08	1.43E+08	28.95	29.86	29.47	28.04	27.66	28.81	29.47	28.06	26.42	29.47	28.04	28.06	0.457357	0.58358	1.525297
1787	P45974-2	Ubiquitin c	USP5	12	21.7	4.76E+06	1.01E+07	9.93E+06	3.63E+06	3.74E+06	5.82E+06	3.13E+06	4.03E+06		22.1	23.45	23.52	21.78	22.03	22.75	21.54	21.75		23.45	22.03	21.64	0.80166	0.503985	0.148683
1158	O15144	Actin-relat	ARPC2	10	31.7	1.56E+07	3.49E+07	3.40E+07	1.41E+07	1.21E+07	1.76E+07	1.85E+07	1.83E+07	5.54E+06	23.84	25.26	25.21	23.78	23.68	24.31	24.11	23.93	21.85	25.21	23.78	23.93	0.80299	0.338044	1.256905
1378	P04075	Fructose-b	ALDOA	24	75	4.52E+08	6.02E+08	4.60E+08	2.36E+08	2.23E+08	3.56E+08	3.16E+08	2.40E+08	1.30E+08	28.82	29.47	29.34	27.9	27.89	28.73	28.13	27.57	26.32	29.34	27.9	27.57	0.345001	0.481274	0.926382
1290	O75937	DnaJ homo	DNAJC8	4	17.4	3.39E+06	8.06E+06	7.44E+06	2.58E+06	2.80E+06	5.33E+06	2.81E+06			21.66	23.12	23.12	21.35	21.66	22.62	21.36			23.12	21.66	21.36	0.843223	0.658167	
1459	P09874	Poly [ADP-	PARP1	20	22.9	2.28E+07	4.41E+07	4.39E+07	1.64E+07	1.64E+07	2.92E+07	1.08E+07	9.43E+06	2.12E+06	24.39	25.61	25.61	24.03	24.14	25.04	23.29	22.94	20.32	25.61	24.14	22.94	0.705247	0.554928	1.624123
1199	O43707	Alpha-acti	ACTN4	40	51.4	1.63E+08	2.53E+08	2.61E+08	9.32E+07	1.04E+08	1.82E+08	1.03E+08	1.17E+08	1.98E+07	27.34	28.35	28.59	26.62	26.88	27.88	26.62	26.59	23.84	28.35	26.88	26.59	0.666604	0.662725	1.59842
2081	Q02878	60S riboso	RPL6	15	44.4	1.60E+08	2.36E+08	2.12E+08	5.31E+07	9.35E+07	1.07E+08	1.12E+08	9.89E+07		27.29	28.22	28.18	25.78	26.71	27	26.75	26.35		28.18	26.71	26.55	0.526188	0.640238	0.279841
972	H9KV75	Alpha-acti	ACTN1	17	37.7	1.79E+07	4.35E+07	3.05E+07	9.59E+06	1.15E+07	1.99E+07	7.00E+06	1.97E+07		24.06	25.58	25.07	23.21	23.6	24.49	22.69	24.05		25.07	23.6	23.37	0.775784	0.656532	0.960555

1730	P36871	Phosphogl	PGM1	10	22.4	8.10E+06	1.55E+07	1.64E+07	5.21E+06	5.39E+06	1.11E+07	6.00E+06	4.44E+06		22.9	24.05	24.19	22.33	22.57	23.6	22.49	21.87		24.05	22.57	22.18	0.704756	0.674858	0.438476	
458	C9JFR7	Cytochrom	CYCS	5	27.7	1.49E+07	2.56E+07	2.71E+07	1.02E+07	9.36E+06	1.77E+07	1.21E+07	7.74E+06	7.24E+06	23.78	24.79	24.89	23.29	23.31	24.33	23.43	22.65	22.29	24.79	23.31	22.65	0.614937	0.591787	0.583737	
1509	P13639	Elongation	EEF2	46	49.4	1.03E+09	1.28E+09	8.43E+08	5.05E+08	4.37E+08	8.23E+08	9.91E+08	3.70E+08	4.26E+08	30.33	31.13	30.39	28.9	28.9	29.89	29.9	28.01	27.93	30.39	28.9	28.01	0.446868	0.575819	1.113501	
1346	P00338	L-lactate d	LDHA	17	47.9	8.17E+08	1.30E+09	1.15E+09	5.19E+08	6.44E+08	8.99E+08	7.39E+08	7.40E+08	3.11E+08	29.72	31.29	30.86	28.93	29.38	30.01	29.36	29.08	27.36	30.86	29.38	29.08	0.809694	0.542359	1.08266	
1607	P24752	Acetyl-CoA	ACAT1	8	21.8	1.11E+07	1.68E+07	1.90E+07	6.55E+06	5.71E+06	1.07E+07	9.03E+06	1.04E+07	4.72E+06	23.39	24.18	24.4	22.67	22.66	23.56	23.05	23.08	21.58	24.18	22.67	23.05	0.528154	0.515177	0.859041	
1338	O95816	BAG family	BAG2	6	28	5.58E+07	2.19E+07	3.63E+06	5.33E+06	8.72E+06	7.20E+06	4.85E+06	4.65E+06	1.20E+07	25.71	24.55	22.12	22.35	23.21	23.04	22.15	21.94	23.1	24.55	23.04	22.15	1.832435	0.456292	0.621262	
1586	P22314-2	Ubiquitin-l	UBA1	33	41.7	1.34E+08	2.12E+08	2.19E+08	6.88E+07	8.61E+07	1.37E+08	9.31E+07	6.50E+07	1.62E+07	27.02	28.08	28.25	26.12	26.56	27.47	26.49	25.78	23.58	28.08	26.56	25.78	0.664702	0.687056	1.518617	
3210	Q9Y383	Putative RI	LUC7L2	8	22.7	2.44E+07	4.37E+07	3.44E+07	9.79E+06	1.23E+07	2.23E+07	2.24E+07			24.5	25.59	25.24	23.23	23.7	24.62	24.42			25.24	23.7	24.42	0.557723	0.706351		
2236	Q14697	Neutral alp	GANAB	29	33.3	6.63E+07	1.67E+08	1.39E+08	3.87E+07	5.55E+07	1.13E+08	2.24E+07	4.62E+07	5.94E+06	25.92	27.71	27.45	25.3	25.9	27.09	24.42	25.27	21.94	27.45	25.9	24.42	0.970888	0.911537	1.727527	
1461	P09972	Fructose-b	ALDOC	7	32.7	1.23E+07	2.29E+07	2.32E+07	7.11E+06	7.61E+06	9.28E+06	8.35E+06			23.52	24.6	24.7	22.79	23.04	23.4	22.92			24.6	23.04	22.92	0.652616	0.303463		
330	A8K878	Mesencepl	MANF	6	31.9	7.64E+06	1.48E+07	1.48E+07	5.08E+06	4.85E+06	7.07E+06	6.50E+06	6.90E+06		22.79	23.99	24.03	22.29	22.41	23.01	22.6	22.48		23.99	22.41	22.54	0.70427	0.386276	0.090229	
2273	Q15293	Reticulocal	RCN1	9	37.8	1.45E+07	1.81E+07	1.97E+07	6.66E+06	5.80E+06	9.55E+06	5.39E+06	5.99E+06		23.74	24.27	24.44	22.69	22.67	23.43	22.33	22.28		24.27	22.69	22.3	0.365316	0.431793	0.040194	
1502	P12277	Creatine ki	CKB	14	52.8	1.83E+08	3.10E+08	2.78E+08	1.07E+08	1.29E+08	1.95E+08	1.45E+08	1.16E+08	7.53E+07	27.56	28.71	28.68	26.77	27.1	28	27.13	26.57	25.63	28.68	27.1	26.57	0.653103	0.638562	0.758793	
2735	Q96G03	Phosphogl	PGM2	4	8.2	2.66E+06	4.84E+06	5.61E+06	1.80E+06	1.61E+06	3.34E+06	3.86E+06	2.06E+06		21.31	22.44	22.76	20.76	20.86	21.97	21.83	20.83		22.44	20.86	21.33	0.764204	0.671525	0.704025	
1354	P00505	Aspartate	GOT2	16	38.6	4.59E+07	5.93E+07	6.62E+07	1.77E+07	2.06E+07	3.99E+07	3.47E+07	2.95E+07	1.05E+07	25.44	26.06	26.28	24.11	24.47	25.51	25.05	24.63	22.89	26.06	24.47	24.63	0.434259	0.725534	1.146779	
2019	P63261	Actin, cyto	ACTG1	29	76.5	1.08E+10	1.22E+10	1.05E+10	7.18E+09	8.24E+09	8.56E+09	1.04E+10	1.14E+10	8.97E+09	34.02	34.73	34.77	32.9	33.13	33.85	33.32	33.09	32.14	34.73	33.13	33.09	0.423705	0.491682	0.6226	
1638	P28070	Proteasom	PSMB4	4	23.1	1.60E+07	1.41E+07	3.48E+06		5.66E+06	3.30E+06	3.88E+06	1.04E+07	7.20E+06	23.89	23.92	22.01		22.63	21.96	21.84	23.08	22.28	23.89	22.29	22.28	1.09531	0.472857	0.6291	
2661	Q92597	Protein ND	NDRG1	5	21.1	9.45E+06	3.69E+06	1.09E+07	2.72E+06	2.60E+06	2.86E+06				23.14	22.08	23.64	21.41	21.52	21.77				23.14	21.52		0.796682	0.183701		
2018	P63244	Guanine nu	GNB2L1	17	71.9	9.41E+07	1.47E+08	1.60E+08	5.78E+07	5.49E+07	1.11E+08	7.72E+07	6.75E+07	1.93E+07	26.59	27.5	27.6	25.88	25.88	27.07	26.12	25.81	23.79	27.5	25.88	25.81	0.557639	0.686422	1.262729	
2243	Q14974	Importin su	KPNB1	20	27.7	1.49E+08	2.14E+08	1.97E+08	6.71E+07	1.33E+08	7.62E+07	1.44E+08	7.52E+07	7.76E+07	27.22	28.1	28.06	26.09	27.13	26.43	27.12	25.97	25.67	28.06	26.43	25.97	0.495585	0.529012	0.76627	
218	A0A0C4DF	Protein SE	SET	8	29.3	2.60E+07	1.29E+08	1.32E+08	3.33E+07	4.69E+07	1.05E+08	6.05E+07	2.68E+07	6.82E+06	24.58	27.3	27.39	25.1	25.67	26.96	25.82	24.49	22.19	27.3	25.67	24.49	1.599095	0.952927	1.838138	
1355	P00558	Phosphogl	PGK1	33	81.3	5.09E+08	7.28E+08	7.98E+08	2.41E+08	2.84E+08	4.66E+08	4.08E+08	3.01E+08	2.27E+08	28.92	29.89	30.13	27.94	28.25	29.11	28.54	27.82	27.01	29.89	28.25	27.82	0.639347	0.610225	0.767394	
1246	O75083	WD repeat	WDR1	13	36	2.87E+07	3.97E+07	4.81E+07	1.42E+07	1.25E+07	2.88E+07	1.67E+07	9.53E+06	5.01E+06	24.73	25.44	25.82	23.79	23.72	25.01	23.96	22.96	21.68	25.44	23.79	22.96	0.553153	0.726385	1.142559	
1206	O43852-2	Calumenin	CALU	8	28.3	8.02E+06	2.63E+07	2.23E+07	8.32E+06	4.99E+06	9.03E+06	8.55E+06	8.25E+06		22.88	24.82	24.65	22.99	22.46	23.35	22.97	22.76		24.65	22.99	22.86	1.077297	0.450273	0.14857	
1284	O75822-3	Eukaryotic	EIF3J	8	47.4	5.32E+06	1.43E+07	1.69E+07	3.63E+06	4.36E+06	1.23E+07	5.37E+06	5.75E+06		22.25	23.94	24.24	21.78	22.25	23.75	22.33	22.22		23.94	22.25	22.28	1.068362	1.030137	0.073231	
224	A0A0C4DG	Probable A	DDX46	22	23	2.54E+07	4.06E+07	3.88E+07	1.17E+07	1.28E+07	2.66E+07	9.72E+06	1.10E+06		24.55	25.47	25.47	23.48	23.77	24.87	23.16	19.87		25.47	23.77	21.52	0.534811	0.731035	2.32359	
1522	P14618	Pyruvate k	PKM	42	72.3	1.33E+09	1.37E+09	1.69E+09	7.51E+08	7.59E+08	1.20E+09	8.29E+08	7.83E+08	5.13E+08	30.64	31.36	31.6	29.5	29.65	30.52	29.52	29.25	28.41	31.36	29.65	29.25	0.503746	0.549653	0.579206	
657	E7ETZ4	Basic leuci	BZW2	11	26.7	2.40E+07	5.31E+07	4.45E+07	6.09E+06	1.46E+07	1.99E+07	1.97E+07	1.94E+07	3.58E+06	24.48	25.88	25.66	22.57	23.94	24.49	24.22	24.02	21.15	25.66	23.94	24.02	0.752706	0.988612	1.717976	
2937	Q9H3N1	Thioredoxi	TMX1	4	16.4		9.08E+06	8.94E+06	2.93E+06	2.69E+06	5.72E+06	3.40E+06	3.37E+06			23.29	23.35	21.51	21.6	22.71	21.64	21.5		23.32	21.6	21.57	0.045178	0.6682	0.100028	
845	G3V5Z7	Proteasom	PSMA6	7	28.6	3.17E+07	3.36E+07	3.53E+07	1.11E+07	1.03E+07	3.03E+07	7.80E+06	8.64E+06	1.03E+07	24.93	25.19	25.31	23.39	23.46	25.1	22.84	22.83	22.85	25.19	23.46	22.84	0.194895	0.964974	0.01005	
1106	O00299	Chloride in	CLIC1	15	68.5	1.01E+08	1.86E+08	1.93E+08	7.15E+07	4.67E+07	1.15E+08	6.73E+07	6.10E+07	2.39E+07	26.7	27.93	28.01	26.19	25.66	27.12	25.91	25.67	24.07	27.93	26.19	25.67	0.735962	0.739665	1.004329	
1715	P35613-2	Basigin	BSG	8	36.8	2.26E+07	2.82E+07	3.20E+07	9.29E+06	6.85E+06	2.52E+07	1.06E+07	1.02E+07	1.92E+07	24.37	24.91	25.11	23.16	22.92	24.75	23.26	23.05	23.78	24.91	23.16	23.26	0.38313	0.996799	0.373198	
1024	J3KTL2	Serine/argi	SRSF1	12	36.8	1.82E+08	1.39E+08	1.45E+08	4.80E+07	5.02E+07	6.34E+07	2.06E+07	3.38E+07		27.55	27.41	27.52	25.64	25.77	26.17	24.31	24.82		27.52	25.77	24.57	0.07193	0.273046	0.359775	
1520	P14324-2	Farnesyl py	FDPS	5	15	1.34E+07	2.98E+07	3.52E+07	8.56E+06	8.80E+06	1.63E+07	8.65E+06			23.63	24.97	25.3	23.02	23.21	24.18	22.99			24.97	23.21	22.99	0.887265	0.622197		
1620	P26038	Moesin	MSN	22	41.6	1.00E+08	1.29E+08	1.29E+08	3.26E+07	4.28E+07	8.67E+07	6.09E+07	4.24E+07	5.80E+06	26.67	27.3	27.32	25.06	25.5	26.61	25.83	25.13	21.92	27.3	25.5	25.13	0.368641	0.800802	2.088112	
3178	Q9UQ35	Serine/argi	SRRM2	56	28.8	5.54E+08	4.50E+08	3.55E+08	9.94E+07	1.42E+08	1.73E+08	9.41E+07	1.21E+08	7.22E+06	29.04	29.18	29.04	26.69	27.22	27.78	26.5	26.65	22.29	29.04	27.22	26.5	0.077602	0.5495	2.475991	
1411	P06733	Alpha-enol	ENO1	32	77.9	1.35E+09	2.04E+09	1.80E+09	6.15E+08	9.64E+08	1.36E+09	1.04E+09	8.70E+08	4.00E+08	30.66	31.97	31.9	29.18	30.04	30.71	29.95	29.44	27.78	31.9	30.04	29.44	0.735274	0.76898	1.135267	
2163	Q13247-3	Serine/argi	SRSF6	9	27.5	1.48E+08	1.21E+08	8.93E+07	3.60E+07	3.65E+07	6.08E+07	1.86E+07	2.32E+07	9.18E+06	27.2	27.21	26.76	25.21	25.29	26.08	24.12	24.28	22.66	27.2	25.29	24.12	0.258151	0.477798	0.893581	
1727	P36543	V-type pro	ATP6V1E1	4	19.9		7.87E+06	8.90E+06	2.46E+06	2.04E+06	4.12E+06	4.27E+06	3.24E+06			23.09	23.34	21.27	21.18	22.26	22	21.46		23.21	21.27	21.73	0.179643	0.601017	0.379213	
743	F2Z393	Transaldol	TALDO1	10	33	3.12E+07	4.34E+07	4.63E+07	1.27E+07	9.20E+06	2.17E+07	1.19E+07	8.44E+06	7.49E+06	24.91	25.58	25.74	23.61	23.28	24.58	23.41	22.79	22.35	25.58	23.61	22.79	0.440462	0.67741	0.53335	
2740	Q96HE7	ERO1-like	ERO1L	8	23.7	7.62E+06	9.29E+06	1.25E+07	2.33E+06	2.05E+06	5.12E+06	2.31E+06	2.65E+06		22.79	23.32	23.82	21.18	21.19	22.55	21.07	21.17		23.32	21.19	21.12	0.5185	0.790339	0.072704	
1385	P04406	Glyceralde	GAPDH	24	65.4	1.51E+09	2.20E+09	1.92E+09	7.50E+08	8.70E+08	1.40E+09	8.72E+08	9.12E+08	6.05E+08	30.89	32.15	32.01	29.48	29.86	30.82	29.63	29.58	28.53	32.01	29.86	29.58	0.688661	0.688556	0.621909	
1961	P61626	Lysozyme	LYZ	2	14.2	1.40E+08			3.14E+07						27.14			24.95						27.14	24.95					
1935	P60174-1	Triosephos	TPI1	15	79.1	1.99E+08	3.38E+08	3.59E+08	9.09E+07	7.41E+07	2.40E+08	6.83E+07	4.62E+07	1.81E+07	27.71	28.79	29.07	26.59	26.33	28.26	25.93	25.27	23.71	28.79	26.59	25.27	0.716261	1.048375	1.136659	
1080	M0R0F0	40S riboso	RPS5	6	27	6.60E+07	1.01E+08	9.86E+07	1.49E+07	2.32E+07	5.55E+07	1.89E+07	1.68E+07		25.91	26.82	26.93	23.87	24.59	25.93	24.16	23.79		26.82	24.59	23.98	0.561288	1.043532	0.262782	
1746	P40121	Macrophag	CAPG	6	27.3	2.82E+06	1.08E+07	1.25E+07	1.89E+06	2.74E+06	2.09E+06	2.98E+06	2.68E+06	3.11E+06	21.39	23.55	23.82	20.87	21.62	21.32	21.47	21.2	20.99	23.55	21.32	21.2	1.335265	0.381025	0.239597	
2	A0A024R4	40S riboso	RPS9	10	40.2	7.02E+07	4.10E+07	2.00E+07	3.67E+06	5.03E+06	2.91E+07	3.74E+06	5.70E+06	2.09E+06	26.05	25.5	24.46	21.8	22.47	25.03	21.79	22.21	20.27	25.5	22.47	21.79	0.806408	1.705585	1.021858	
2053	Q00403	Transcripti	GTF2B	2	6.6		6.59E+05									19.8								19.8						
1568	Q32MN6	TATA-box-l	TBP	2	5.9		5.54E+05	1.04E+06								19.33	20.33							19.83			0.705639			
484	H7C2W1	D-beta-hyc	BDH1	2	13.2		1.06E+06	7.53E+05								20.38	19.79							20.09			0.413621			
479	C9JVN9	L-2-hydrox	L2HGDH	2	5.4		9.35E+05									20.13								20.13						
2716	Q96CM3-2	RNA pseud	RPUSD4	3	6.9		9.46E+05									20.16								20.16						+
408	B7Z7F3	Ran-bindin	RANBP3	2	5.3		9.83E+05	8.82E+05								20.23	20.1							20.16			0.096379			
2851	Q9BU61-2	NADH deh	NDUFAF3	2	16.5			9.65E+05									20.23							20.23						
1008	J3KQ34	COP9 signa	COPS7B	2	10.4			9.77E+05									20.28							20.28						
3208	Q9Y316-2	Protein ME	MEMO1	3	13.5	1.30E+06	1.20E+06	1.01E+06					1.34E+06		20.21	20.52	20.3					20.18		20.3		20.18	0.160108			
455	H7C131	3-ketoacyl	ACAA1	2	14.8		1.31E+06	8.61E+05								20.62	20.02							20.32			0.420584			
2762	Q96P11-5	Probable 2	NSUN5	2	5.9			1.10E+06									20.43							20.43						
2002	P62877	E3 ubiquiti	RBX1	2	24.1			1.10E+06									20.43							20.43						
1322	O95391	Pre-mRNA	SLU7	2	5.3			1.10E+06									20.43							20.43						
63	A0A087WV	Endophilin	SH3GLB1	2	6.1			1.12E+06									20.47							20.47						
456	C9JEX3	Myotubula	MTMR2	3	6.1		1.17E+06									20.47								20.47						
460	C9JGI3	Thymidine	TYMP	2	5.4			1.14E+06									20.5							20.5						
1341	O95900	Probable tl	TRUB2	2	6.3		1.54E+06	9.28E+05								20.87	20.15							20.51			0.507602			
3063	V9GYX7	Actin-relat	ACTR10	2	7.6			1.15E+06									20.51							20.51						
390	B5MBZ8	Protein ph	PPP1R7	3	15			1.20E+06									20.57							20.57						
1185	O43237-2	Cytoplasm	DYNC1LI2	2	5.8		1.15E+06	1.32E+06								20.45	20.7							20.57			0.178197			
3046	Q9NWY4	UPF0609 p	C4orf27	2	5.8			1.24E+06									20.62							20.62						
31	A0A087WU	Vacuolar p	VPS45	2	5.1		1.49E+06	1.14E+06								20.79	20.5							20.64			0.208256			
887	H0Y9B6	Beta-hexos	HEXB	2	15.3		9.73E+05	1.89E+06								20.18	21.2							20.69			0.718422			
2905	Q9H061	Transmem	TMEM126A	2	14.9		1.01E+06	1.77E+06								20.28	21.11							20.7			0.590356			+
1197	O43615	Mitochond	TIMM44	3	6.9		1.73E+06	1.08E+06								21	20.39							20.7			0.428406			
2556	Q8N335	Glycerol-3	GPD1L	2	6		1.35E+06	1.43E+06								20.64	20.78							20.71			0.102105			
2843	Q9BT09	Protein car	CNPY3	2	13.3		1.38E+06	1.43E+06								20.66	20.78							20.72			0.087109			
337	A8MWX3	Putative W	WASH4P	3	6.7		1.40E+06	1.39E+06								20.7	20.75							20.73			0.038773			
2458	Q6UWP7-2	Lysocardio	LCLAT1	3	11		1.56E+06	1.24E+06								20.88	20.62							20.75			0.187725			
721	E9PQW0	Peroxisom	PEX16	2	36.7			1.42E+06									20.77							20.77						

2162	Q13243	Serine/argi	SRSF5	3	19.9	1.89E+06						20.78						20.78				
2750	Q96JB5-2	CDK5 regul	CDK5RAP3	3	5.5		1.45E+06	1.49E+06					20.74	20.83				20.79		0.066588		+
1825	P49366	Deoxyhypu	DHPS	2	6			1.45E+06						20.81				20.81				
473	G5E9W8	Glycogenir	GYG1	2	13.5		1.50E+06						20.82					20.82				
2314	Q16537-2	Serine/thre	PPP2R5E	2	5.8		1.69E+06	1.28E+06					20.96	20.69				20.83		0.190081		
122	Q2TAM5	Transcripti	RELA	2	6.1		1.74E+06	1.25E+06	1.10E+06				21.02	20.64	19.94			20.83	19.94	0.268483		
3247	Q9Y5P6	Mannose-1	GMPPB	2	8.9		1.43E+06	1.64E+06	1.71E+06				20.72	20.96	20.62			20.84	20.62	0.17013		
2876	Q9BXR0	Queuine tF	QTRT1	2	6.9	1.52E+06	1.52E+06	1.52E+06	1.57E+06			20.46	20.84	20.86	20.54			20.84	20.54	0.223106		
280	B0QYG7	Protein kin	PACSIN2	2	13.2		1.50E+06	1.68E+06					20.82	21.01				20.91		0.136289		
820	F8W8D4	NEDD8-act	UBA3	3	7.6		1.04E+06	2.42E+06					20.34	21.51				20.93		0.827043		
746	F5GWI9	WASH com	CCDC53	2	15.4		1.64E+06	1.64E+06					20.93	20.96				20.94		0.022568		
1085	M0R1T5	Charged m	CHMP2A	2	12.3		1.68E+06						20.96					20.96				
1037	K7EIN2	Protein syr	NUDT16L1	2	11.1	2.13E+06						20.96						20.96				
2111	Q08188	Protein-glu	TGM3	3	6.2			1.66E+06			5.95E+06			20.98			21.95	20.98	21.95			
616	E5RFZ8	Zinc transp	SLC39A14	2	28.1		1.73E+06	1.72E+06					21	21.07				21.04		0.05139		
1062	K7EQG9	Tyrosine-p	PTPN2	3	9.6			1.71E+06						21.06				21.06				
792	F8VS07	LIM domai	LIMA1	2	6.1			1.74E+06						21.09				21.09				
1453	Q6ZN40	Tropomyos	TPM1	2	14.7			1.74E+06						21.09				21.09				
351	B0QZK4	Heterochro	HP1BP3	2	7.9		1.65E+06	2.00E+06					20.94	21.26				21.1		0.229371		
2655	Q92522	Histone H1	H1FX	2	10.8		1.84E+06						21.11					21.11				
215	H0Y2P2	Peptide-N(	NGLY1	2	8.3		1.84E+06						21.11					21.11				
27	A0A087W\	E3 ubiquiti	UHRF1	4	6.3		1.99E+06	1.75E+06					21.23	21.1				21.17		0.095376		
2642	Q8WW12-	PEST prote	PCNP	2	20			1.86E+06						21.18				21.18				
2248	Q15006	ER membr	EMC2	3	14.1		1.76E+06	2.10E+06					21.04	21.33				21.18		0.204321		
183	A0A0A0MS	Aldo-keto r	AKR1C3	2	9.6	2.45E+06						21.19						21.19				
737	E9PS00	Fatty acid c	FADS3	2	6		1.77E+06	2.11E+06					21.06	21.34				21.2		0.19623		
2384	Q5T447	E3 ubiquiti	HECTD3	5	8		2.03E+06						21.25					21.25				
1963	P61769	Beta-2-mic	B2M	2	26.9			2.02E+06						21.28				21.28				
1232	O60671-2	Cell cycle c	RAD1	2	13.4		2.06E+06						21.28					21.28				
79	A0A087WX	Nucleolar p	ZCCHC17	2	15.8			2.07E+06						21.31				21.31				
996	J3KNK1	Mitogen-ac	MAPK9	3	8.3			2.11E+06						21.34				21.34				
2873	Q9BX68	Histidine tr	HINT2	2	22.1	1.39E+06	2.17E+06	3.46E+06	2.35E+06			20.32	21.34	22.01	21.09			21.34	21.09	0.848448		
26	A0A087W1	Pyrroline-5	PYCR2	2	17.1		2.18E+06						21.35					21.35				
1929	P55957	BH3-intera	BID	2	14.4			2.14E+06						21.35				21.35				
945	H3BUB4	Snurportin	SNUPN	2	8.5			2.16E+06						21.37				21.37				
2972	Q9H9G7	Protein arg	AGO3	2	12.8		2.25E+06						21.37					21.37				
2319	Q16643	Drebrin	DBN1	3	8.9	2.64E+06	3.80E+06	2.22E+06				21.29	22.15	21.42				21.42		0.464715		
2350	Q4U2R6	39S ribosol	MRPL51	2	22.7			2.22E+06						21.42				21.42				
2423	Q6DKI1	60S ribosol	RPL7L1	2	10.6	2.84E+06	2.56E+06	2.27E+06				21.4	21.54	21.43				21.43		0.074583		
18	I3L159	Heme oxyg	HMOX2	3	16.2		2.91E+06	1.87E+06					21.72	21.19				21.45		0.376487		
344	H0Y645	Casein kina	CSNK1E	2	8.5		2.45E+06						21.49					21.49				
592	D6RAN8	39S ribosol	MRPL27	2	22.6		2.58E+06	2.34E+06					21.55	21.46				21.5		0.059101		
2916	X6RM59	5-nucleotic	NT5C3A	2	7.6		2.51E+06						21.52					21.52				
2698	Q96A35	39S ribosol	MRPL24	2	10.6	3.02E+06	2.92E+06	2.17E+06				21.52	21.73	21.37				21.52		0.177375		
611	E5RFU0	Phosphatic	SNAP91	2	17.2		1.95E+06	3.04E+06	1.30E+06	1.37E+06			21.2	21.85	20.15	20.22		21.52	20.18	0.45409	0.04333	
2920	Q9H1E3	Nuclear ub	NUCKS1	2	8.2		2.24E+06	2.80E+06					21.36	21.71				21.54		0.24758		
1227	O60547-2	GDP-mann	GMDS	2	5			2.52E+06						21.56				21.56				
3255	Q9Y676	28S ribosol	MRPS18B	3	16.3			2.52E+06						21.56				21.56				
1432	P08133-2	Annexin A6	ANXA6	4	8.7		2.39E+06	2.80E+06					21.45	21.71				21.58		0.18874		
1732	P36952	Serpin B5	SERPINB5	2	6.9	3.22E+06						21.6						21.6				
772	F5H608	ATP syntha	ATP5H	3	23.5	1.68E+06	3.65E+06	2.58E+06				20.56	22.06	21.6				21.6		0.770254		
782	F6S7Q7	Threonine-	TARS2	3	6.5		2.71E+06						21.6					21.6				
1426	P07858	Cathepsin	CTSB	2	8.3			2.62E+06						21.62				21.62				
271	E9PLH9	GDP-L-fuco	TSTA3	2	19.3		1.85E+06	3.65E+06					21.12	22.13				21.63		0.713262		
2230	Q14677	Clathrin int	CLINT1	3	6.1		2.82E+06						21.65					21.65				
2079	Q02790	Peptidyl-pr	FKBP4	2	7.4	3.39E+06						21.66						21.66				
341	A8MZF9	Developme	DRG2	3	12.2		2.53E+06	3.04E+06					21.54	21.85				21.69		0.218182		
2527	Q8IV48	3-5 exoribo	ERI1	2	6.3		2.89E+06			3.16E+06			21.71			21.44		21.71	21.44			
2997	Q9NPJ6	Mediator c	MED4	5	21.9		2.59E+06	3.19E+06		4.27E+06			21.56	21.91		21.82		21.73	21.82	0.250688		
2959	Q9H7Z7	Prostaglan	PTGES2	3	12.2			2.82E+06						21.73				21.73				
2497	Q7Z417	Nuclear fra	NUFIP2	4	7.3		2.96E+06						21.75					21.75				
2860	Q9BV57	1,2-dihydrc	ADI1	2	17.3			2.85E+06						21.75				21.75				
2567	Q8N6R0-3	Methyltrar	METTL13	2	5.7		3.00E+06						21.76					21.76				
942	H3BRV9	Nuclear tra	NUTF2	2	29	5.71E+06		1.90E+06				22.36		21.21				21.79		0.813432		
1032	J3QRS9	BUB3-inter	ZNF207	2	6.7		3.75E+06	2.37E+06					22.13	21.47				21.8		0.461819		
2577	Q8NBF2-2	NHL repea	NHLRC2	2	6.3	3.32E+06	3.51E+06		2.78E+06			21.64	22.02		21.34			21.83	21.34	0.269424		
1590	P22735	Protein-glu	TGM1	4	6.1			3.06E+06			1.37E+07			21.86			23.31	21.86	23.31			
825	K7ENK9	Vesicle-ass	VAMP2	2	35.3			3.08E+06						21.87				21.87				
454	C9JBI3	Phosphose	PSPH	3	18.2			3.09E+06						21.87				21.87				
2088	Q04760-2	Lactoylglut	GLO1	4	25.4	5.40E+06	3.21E+06	2.61E+06				22.27	21.88	21.62				21.88		0.330006		
1009	J3KQ48	Peptidyl-tR	PTRH2	2	18.9		3.21E+06						21.88					21.88				
2574	Q8NAV1	Pre-mRNA	PRPF38A	5	15.1	4.13E+06	3.05E+06	3.30E+06				21.93	21.79	21.96				21.93		0.091417		
2828	Q9BRK5-6	45 kDa calc	SDF4	4	14.7		3.84E+06	2.98E+06					22.16	21.82				21.99		0.245844		
141	A0A087X2I	C-Jun-amir	SPAG9	5	6.7	3.33E+06	3.84E+06	3.43E+06				21.64	22.16	21.99				21.99		0.267573		
922	H3BMH2	Ras-relatec	RAB11A	2	15.5	4.35E+06						22						22				
1221	O60488-2	Long-chain	ACSL4	2	10.3	4.57E+06						22.05						22.05				
9	A0A075B6I	Nitric oxide	NOSIP	3	13.8			3.55E+06						22.07				22.07				

1765	P42025	Beta-centr	ACTR1B	2	25.8	4.69E+06									22.08									22.08								
273	J3QT77	Serum para	PON2	3	11.4		4.07E+06	3.44E+06								22.24	22							22.12			0.174219					
1039	K7EIY1	Protein FA	FAM32A	2	23.4			3.66E+06									22.14							22.14								
3211	Q9Y399	28S riboso	MRPS2	3	8.8	3.81E+06		4.63E+06					3.87E+06		21.83		22.46					21.7		22.14		21.7	0.442302					
2040	P82675	28S riboso	MRPS5	3	8.8	2.49E+06	3.96E+06	3.75E+06				2.91E+06			21.21	22.22	22.17				21.42			22.17		21.42	0.564958					
2318	Q16610-2	Extracellul	ECM1	2	6.5			3.76E+06									22.18							22.18								
324	A6NNI4	Tetraspani	CD9	2	10.7			3.78E+06									22.19							22.19								
447	C9J719	3-beta-hyd	EBP	2	17.8		4.01E+06									22.22								22.22								
1517	P13929-3	Beta-enola	ENO3	3	23.8		3.93E+06	4.17E+06								22.2	22.31							22.25			0.080506					
768	F5H4B6	Aldehyde c	ALDH16A1	3	5.6		4.12E+06					6.19E+06				22.26					22.53			22.26		22.53						
2827	Q9BRF8-3	Serine/thre	CPPED1	2	18		3.96E+06	4.39E+06								22.22	22.38							22.3			0.116944					
2235	Q14696	LDLR chape	MESDC2	3	14.5			4.15E+06									22.3							22.3								
2960	Q9H814	Phosphory	PHAX	2	6.9			4.22E+06									22.33							22.33								
970	H8Y6P7	DNA-direct	GCOM1	3	6.8		4.54E+06					1.60E+07				22.36					23.88			22.36		23.88						
336	C9JN83	N-alpha-ac	NAA10	2	11.3			4.38E+06									22.38							22.38								
683	E9PDQ8	Succinyl-Co	SUCLG2	6	15.6		4.84E+06	4.77E+06								22.44	22.5							22.47			0.038705					
2078	Q02750	Dual specif	MAP2K1	4	22.9		4.53E+06	5.17E+06								22.36	22.6							22.48			0.172574					
3160	Q9UMS4	Pre-mRNA	PRPF19	2	10.9	6.15E+06									22.48									22.48								
169	A0A0A0MF	Probable g	SMARCA1	2	5.6			4.84E+06									22.52							22.52								
2327	Q16822	Phosphoer	PCK2	2	6.6	6.30E+06									22.52									22.52								
2711	Q96BR5	Cytochrom	COA7	2	10		5.37E+06	4.81E+06								22.57	22.51							22.54			0.037663					
1687	P31944	Caspase-14	CASP14	5	18.2			5.07E+06									22.57							22.57								
2333	Q2NL82	Pre-rRNA-p	TSR1	5	9.2		5.38E+06	5.22E+06								22.57	22.62							22.6			0.03899					
830	G3V0E4	Mitochond	PMPCB	4	8.2		5.39E+06	5.77E+06					3.79E+06			22.57	22.79					21.67		22.68		21.67	0.151664					
1052	K7EM18	Eukaryotic	EIF1	4	58.7	4.20E+06	6.06E+06	5.43E+06							21.96	22.7	22.71							22.7			0.428349					
2404	Q5VTL8-2	Pre-mRNA	PRPF38B	2	7.9			5.46E+06									22.72							22.72								
2307	Q16186	Proteasom	ADRM1	3	10.6	7.40E+06	3.38E+06	7.45E+06							22.74	21.95	23.12							22.74			0.595473					
639	E7EQ72	Transmem	TMED2	2	16.9	7.63E+06									22.79									22.79								
1069	M0QXD5	PIH1 doma	PIH1D1	2	10.2		6.67E+06									22.85								22.85								
310	A6NF51	3(2),5-bisp	BPNT1	3	11		6.68E+06	6.87E+06								22.85	22.98							22.91			0.091584					
149	A0A0A0MO	Protein RT	RTFDC1	3	9.5		6.59E+06	7.08E+06								22.83	23.01							22.92			0.130517					
147	A0A096LN	Ubiquitin-l	ISG15	2	15.3			7.01E+06									23.01							23.01								
1564	P19838	Nuclear fac	NFKB1	4	5.9		8.10E+06	6.87E+06				7.43E+06				23.13	22.98				22.76			23.05		22.76	0.104977					
2003	P62879	Guanine nu	GNB2	2	20.3		9.94E+06						1.01E+07			23.42						23.04		23.42		23.04						
1157	O15143	Actin-relat	ARPC1B	2	8.3			9.42E+06									23.44							23.44								
1239	O60831	PRA1 famil	PRAF2	2	16.3	1.01E+07	1.09E+07	1.06E+07					1.54E+07		23.25	23.57	23.59					23.67		23.57		23.67	0.192209					
1484	P11217	Glycogen p	PYGM	2	8.8	1.67E+07									23.96									23.96								
5	A0A024RA	Proteasom	PSMA2	2	17.1	2.17E+07									24.32									24.32								
2342	Q3ZCM7	Tubulin be	TUBB8	2	28.2	3.27E+07									24.97									24.97								
3044	Q9NWB6	Arginine ar	ARGLU1	3	8.4		3.19E+07	7.24E+07				1.04E+08	3.81E+07	6.64E+07		25.07	26.47				26.64	24.99	25.45	25.77		25.45	0.990234		0.854958			
591	D6RAN4	60S riboso	RPL9	4	34.6	9.32E+07									26.58									26.58								
312	A6NGJ7	Histone de	HDAC8	2	11.3								2.20E+07									24.2				24.2						
2800	Q99538-3	Legumain	LGMN	2	7.8								2.17E+07									24.18				24.18						
442	C9J4Z3	60S riboso	RPL37A	2	39.7								1.81E+07									23.91				23.91						
1274	O75607	Nucleoplas	NPM3	2	20.8									1.44E+07									23.39			23.39						+
1806	P47929	Galectin-7	LGALS7	2	14							8.61E+06									22.98					22.98						+
1506	P13073	Cytochrom	COX4I1	2	13								5.70E+06									22.21				22.21						
3188	Q9Y277	Voltage-de	VDAC3	2	6.7							3.62E+06									21.72					21.72						
705	E9PJM3	F-box only	FBXO3	2	6.7								1.93E+06									20.73				20.73						
2175	Q13426-3	DNA repair	XRCC4	2	8.7								1.71E+06									20.5				20.5						
481	C9JYM0	Ribonuclea	POP7	2	24.8								1.68E+06									20.45				20.45				+		
19	E9PPB5	Ester hydro	C11orf54	2	15.7									2.20E+06									20.43			20.43						
291	A0MZ66-8	Shootin-1	KIAA1598	3	8							1.37E+06									20.22					20.22						
1713	P35609-2	Alpha-actin	ACTN2	2	9.3								9.35E+05									19.63				19.63						
370	M0QZP4	Branched-c	BCAT2	2	10.9							9.36E+05									19.61					19.61						+
2393	Q5TA58	Protein arg	AGO1	2	10.1							7.44E+05									19.33					19.33						
1133	O14744	Protein arg	PRMT5	60	84.1	7.84E+10	5.95E+10	5.97E+10	1.21E+11	1.15E+11	9.00E+10	1.65E+11	1.93E+11	1.64E+11	36.76	36.76	36.76	36.76	36.76	36.76	36.76	36.76	36.76	36.76	36.76	36.76	0	0	0			
2445	Q6PDB4	Zinc finger	ZNF880	2	5.7																											
